# Copper-Catalyzed *gem*-Dichloroalkyl-Arylation of Unactivated Alkenes for the Synthesis of Dichloroalkyl-Azaheteropolycycles

**DOI:** 10.1021/acs.joc.5c02773

**Published:** 2025-12-23

**Authors:** Gustavo G. Flores-Bernal, Carlos S. Ulloa-Chacha, Marco T. Espinoza-Nicolás, Luis D. Miranda

**Affiliations:** Department of Organic Chemistry, Instituto de Química, Universidad Nacional Autónoma de México, Circuito Exterior, Ciudad Universitaria, 04510 Mexico City, Mexico

## Abstract

We report copper-catalyzed dichloroalkyl-arylation of terminal alkenes containing a pendant aryl group bearing trichloroalkyl compounds as radical precursors. Tandem radical cyclization proceeded with high regioselectivity, short reaction times, and tolerance for a broad array of functional groups and complex substrates, yielding fused azaheterocycles such as pyrrolizidines and indolizidines, which serve as important intermediates in total synthesis. This reaction sequence affords the installation of the *gem*-dichloroalkyl group, a potent pharmacophore and versatile building block in organic synthesis.

## Graphical abstract

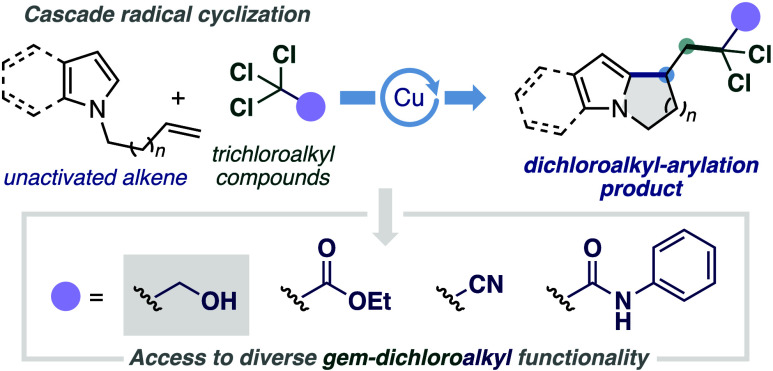

## Introduction

Installing polychloroalkyl groups within molecular architectures is a task in high demand in organic synthesis since these structural motifs occur in natural products, pharmaceuticals, and agrochemicals with relevant biological activity (Figure 1a).^1^ While nature’s pathway for incorporating chlorine atoms by chemoenzymatic processes is highly selective to afford bioactive compounds,^2^ conventional synthetic halogenation protocols often require toxic reagents, harsh reaction conditions, and the formation of unwanted byproducts.^3^ In recent years, several efficient synthetic methods have been reported for the functionalization of substrates with polychloroalkyl groups.^4^ Among these methods, the radical-mediated 1,2-difunctionalization of alkenes has proven to be a powerful and efficient transformation to obtain complex compounds in a single reaction step through the formation of two chemical bonds. The addition of polychloroalkyl radicals generated by selective C–Cl and C–H bond homocleavage of simple alkyl chlorides (e.g., CCl_4_, CHCl_3_, CH_2_Cl_2_) onto olefins can follow three different reaction paths (Figure 1b): (i) atom-transfer radical addition (ATRA, Kharasch-type reaction)^5^ and cyclization (ATRC);^6^ (ii) transition-metal-catalyzed or photoredox-catalyzed three-component difunctionalization;^7^ and (iii) radical cascade cyclization with pendant alkenes anchored on (hetero)­arenes for access to polychloroalkyl heterocycles.^4^

**Figure 1:**
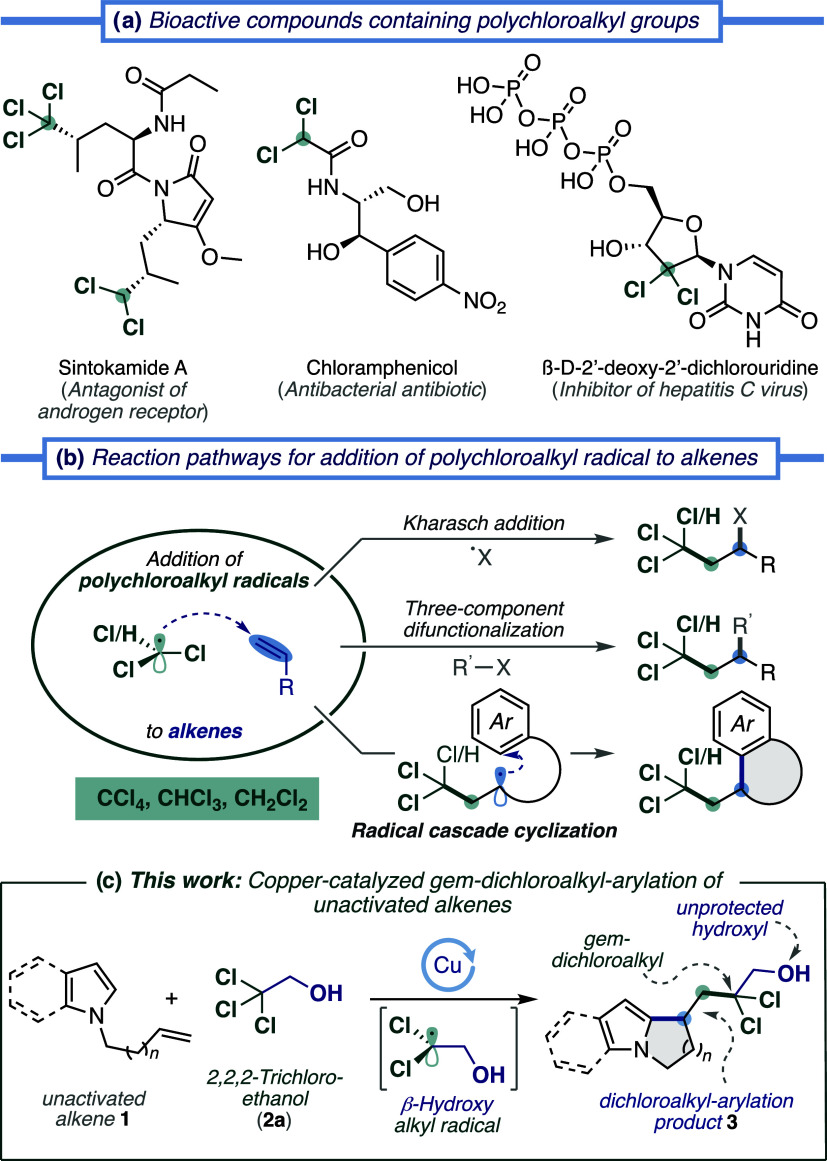
Significance of polychlorinated molecules and representative strategies for the polychloroalkylation of alkenes.

In recent years, significant efforts have been devoted to the development of polychloroalkyl/arylation methodologies of alkenes via radical cascade cyclization for the construction of heteropolycyclic architectures with polyhalomethanes as precursors of polychloromethyl radicals, such as oxindoles,^8^ indolines,^9^ dihydroisoquinolinones, pyrrolo-piperidino-quinazolinones, pyrrolo­[1,2-*a*]­indoles, and pyrido-pyrrolo-indolones.^10^ Despite the progress achieved so far, the abovementioned methods for synthesizing polychloroheterocycles are mainly limited to the use of stoichiometric amounts of difficult to handle and unsafe peroxides. Moreover, the polychloroalkyl precursors used have been limited to simple alkyl chlorides (CCl_4_, CHCl_3_, CH_2_Cl_2_, BrCCl_3_, and BrCHCl_2_).

Nowadays there is a high demand for the introduction of polyhalogenated fragments into valuable molecular scaffolds; however, recently published approaches focus on the introduction of simple polyfluoroalkyl groups,^11^ while the introduction of functionalized polyhaloalkyl groups remains as an unaddressed challenge. Advancement to the next level of research in this field will depend on the use of more complex polychloroalkyl radical precursors, especially those with functional groups that can facilitate subsequent transformations. Accordingly, during the construction of polycyclic compounds, these precursors might be incorporated as pendant synthons, which enable derivatization and access to intermediates to produce value-added molecules.

Furthermore, the incorporation of chlorine atoms into drugs and natural products not only influences bioactivity and specificity but also allows for the conversion of polychloroalkyl groups into a variety of functional groups including carbonyl derivatives, carbenes, alkynes, *gem*-diboronic esters, and cyclopropanes.^12^

Our laboratory has had an ongoing interest in radical cascade cyclization reactions for synthesizing polycyclic compounds present in biologically active natural products^13^ and the copper-mediated radical alkylation of heteroarenes.^14^ Herein, we describe our recent findings on the copper-catalyzed *gem*-dichloroalkyl/arylation of unactivated alkenes for constructing *gem*-dichloroalkyl heterocycles **3** via radical cascade cyclization with *N*-alkene-tethered heterocycles **1** (Figure 1c). We report the unprecedented copper-catalyzed generation of β-hydroxyalkyl radicals from readily available 2,2,2-trichloroethanol (**2a**, TCE), which is a reagent conventionally used as a protecting group for carboxylic acids^15^ and as a perhalogenated solvent in challenging C–H functionalization reactions.^14,16^ It is essential to note that single electron transfer (SET) reduction of the trichloromethyl group occurred with high selectivity despite the presence of an unprotected hydroxyl group. Thus, the alkylation of the unactivated alkene with a two-carbon fragment containing *gem*-dichloroalkyl and hydroxyl groups and the subsequent arylation were achieved. This strategy provides an efficient and feasible approach for several biologically important pyrrolizidine and indolizidine motifs in moderate to good yields.

## Results and Discussion

We began our studies optimizing the reaction conditions using 1-(4-penten-1-yl)-1*H*-pyrrole-2-carboxaldehyde (**1a**) as the model substrate and TCE (**2a**) as the dichloroalkylating reagent (Table 1). To our delight, we found that the reaction of **1a** (0.4 mmol) in TCE (0.4 M) occurred in the presence of a Cu­(OAc)_2_ (10 mol %)/TMEDA (10 mol %) catalyst system and K_2_CO_3_ (0.8 mmol) as a base, at 70 °C for 1.5 h to access the desired *gem*-dichloroalkyl/arylation product **3a** in 60% yield (Table 1, entry 1). Unreacted **1a** was recovered, and byproduct formation was negligible.

**Table 1:**
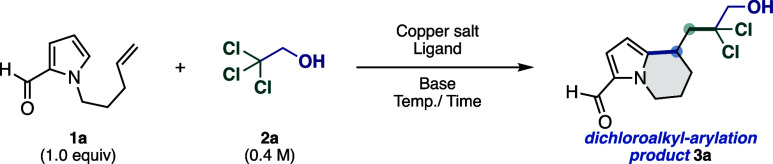
Optimization of the Reaction Conditions^a^,^d^

entry	copper salt (mol %)	ligand (mol %)	base (equiv)	Temp. (°C)	time (h)	**3a** (%)^b^
1	Cu(OAc)_2_ (10)	TMEDA (10)	K_2_CO_3_ (2)	70	1.5	60
2	Cu(EH)_2_ (10)	TMEDA (10)	K_2_CO_3_ (2)	70	1.5	50
3	CuSO_4_ (10)	TMEDA (10)	K_2_CO_3_ (2)	70	1.5	7
4	CuCl_2_ (10)	TMEDA (10)	K_2_CO_3_ (2)	70	1.5	24
5	CuBr_2_ (10)	TMEDA (10)	K_2_CO_3_ (2)	70	1.5	6
6	Cu(OAc)_2_ (10)	phen (10)	K_2_CO_3_ (2)	70	1.5	54
7	Cu(OAc)_2_ (10)	bpy (10)	K_2_CO_3_ (2)	70	1.5	56
8	Cu(OAc)_2_ (10)	TPMA (10)	K_2_CO_3_ (2)	70	1.5	60
9	Cu(OAc)_2_ (10)	4-MOTPA (10)	K_2_CO_3_ (2)	70	1.5	15
10	Cu(OAc)_2_ (10)	TMEDA (10)	Na_2_CO_3_ (2)	70	1.5	65
11	Cu(OAc)_2_ (10)	TMEDA (10)	Cs_2_CO_3_ (2)	70	1.5	22
12	Cu(OAc)_2_ (10)	TMEDA (10)	K_3_PO_4_ (2)	70	1.5	25
13	Cu(OAc)_2_ (10)	TMEDA (10)	2,6-lutidine (2)	70	1.5	9
14	Cu(OAc)_2_ (10)	TMEDA (10)	2,4,6-collidine (2)	70	1.5	11
15	Cu(OAc)_2_ (20)	TMEDA (20)	Na_2_CO_3_ (2)	70	1.5	63
16	Cu(OAc)_2_ (2.5)	TMEDA (5)	Na_2_CO_3_ (2)	70	1.5	62
17	Cu(OAc)_2_ (10)	TMEDA (10)	Na_2_CO_3_ (2)	110	0.25	62 (67)^c^

a Reactions were performed on a 0.4 mmol scale.

b Isolated yield.

c With 1 mmol **1a**.

d Abbreviations: TCE, 2,2,2-trichloroethanol; TMEDA, *N*,*N*,*N*′,*N*′-tetramethylethylenediamine; Cu­(EH)_2_, copper­(II) 2-ethylhexanoate; phen, 1,10-phenanthroline; bpy, 2,2′-bipyridyl; TPMA, tris­(2-pyridylmethyl)­amine; 4-MOTPA, 4-methoxytriphenylamine.

We then evaluated various copper­(II) salts, such as Cu­(EH)_2_, CuSO_4_, CuCl_2_, and CuBr_2_; however, only lower yields of product **3a** were obtained (Table 1, entries 2–5). Thus, Cu­(OAc)_2_ was established as the best choice. The requirement for using TMEDA as a ligand was demonstrated since the formation of product **3a** was not observed in its absence. In this sense, the efficiency of a set of representative tertiary amine ligands was evaluated; similar results were obtained with bidentate ligands 1,10-phenanthroline and 2,2′-bipyridyl (Table 1, entries 6 and 7), and tridentate ligand tris­(2-pyridylmethyl)­amine (Table 1, entry 8), while the use of the monodentate ligand 4-methoxytriphenylamine resulted in a significant decrease in yield (15%) (Table 1, entry 9). We next screened several inorganic and organic bases. By using Na_2_CO_3,_ a slight increase in the yield of product **3a** was achieved (65%) (Table 1, entry 10); however, with Cs_2_CO_3_ and K_3_PO_4,_ the yield of **3a** decreased (Table 1, entries 11 and 12) and was even lower when pyridine-type organic bases were used (Table 1, entries 13 and 14). Experiments performed with higher and lower loadings of catalyst and ligand led to slightly lower yields (Table 1, entries 15 and 16), and thus the use of 10 mol % of both reagents was preferable. Remarkably, TLC monitoring of an experiment performed using Cu­(OAc)_2_ (10 mol %), TMEDA (10 mol %), and Na_2_CO_3_ (2 equiv) at 110 °C revealed that after 15 min the consumption of substrate **1a** was complete, affording product **3a** in 62% yield (Table 1, entry 17). In this sense, it is possible to consider both pairs of temperature/reaction time values (70 °C, 1.5 h/110 °C, 15 min) as optimal for the copper-catalyzed radical cascade cyclization of *N*-alkene-tethered heterocycles **1** with trichloroalkyl compounds **2**.

Under optimal conditions, we evaluated the *gem*-dichloroalkyl-arylation of various *N*-alkene-tethered heterocycles **1** with trichloroalkyl compounds **2** (Scheme 1). First, we investigated the scope of the reaction with several *N*-alkene-tethered heterocycles **1** and TCE (**2a**) as the solvent and radical precursor. The effect of the *N*-alkyl tether chain length on the radical cascade cyclization over a homologous series of alkene tethers ranging from two to four bridging methylene groups (**1a**–**c**) showed that in addition to indolizidine **3a** it was possible to access pyrrolizidine **3b** and pyrrolo­[1,2-*a*]­azepine **3c** in 57% and 31% yields, respectively. Then, we investigated the scope of optimized reaction conditions with terminal alkenes tethered to indole and 7-azaindole, which were compatible to afford dichloroalkyl-heteroarylation products **3d**–**i**. Indolizidines **3d**,**e**,**h** were obtained in higher yields (41–65%) than pyrrolizidines **3f**,**g**,**i** (20–49%). Seeking to expand the scope of this strategy and accessing other high-value heterocyclic scaffolds, several alkene-tethered derivatives were evaluated. Thus, aniline **1j**, imidazole **1k**, and thiophen-2-ylmethanol **1l** derivatives reacted to afford indoline **3j**, imidazo­[2,1-*a*]­isoquinoline **3k**, and thieno­[2,3-*c*]­pyran **3l**. However, arylsulfonamide **1m**, pyrazole **1n**, and carbazole **1o**,**p** derivatives only led to atom-transfer (ATRA) products **4m**–**p**, respectively. Likewise, ATRA product **4c** was isolated as a byproduct when 1-(hex-5-en-1-yl)-1*H*-pyrrole-2-carbaldehyde (**1c**) was used. We hypothesize that ATRA products are formed by stereoelectronically disfavored intramolecular cyclization onto the aromatic ring in these substrates.

**Scheme 1:**
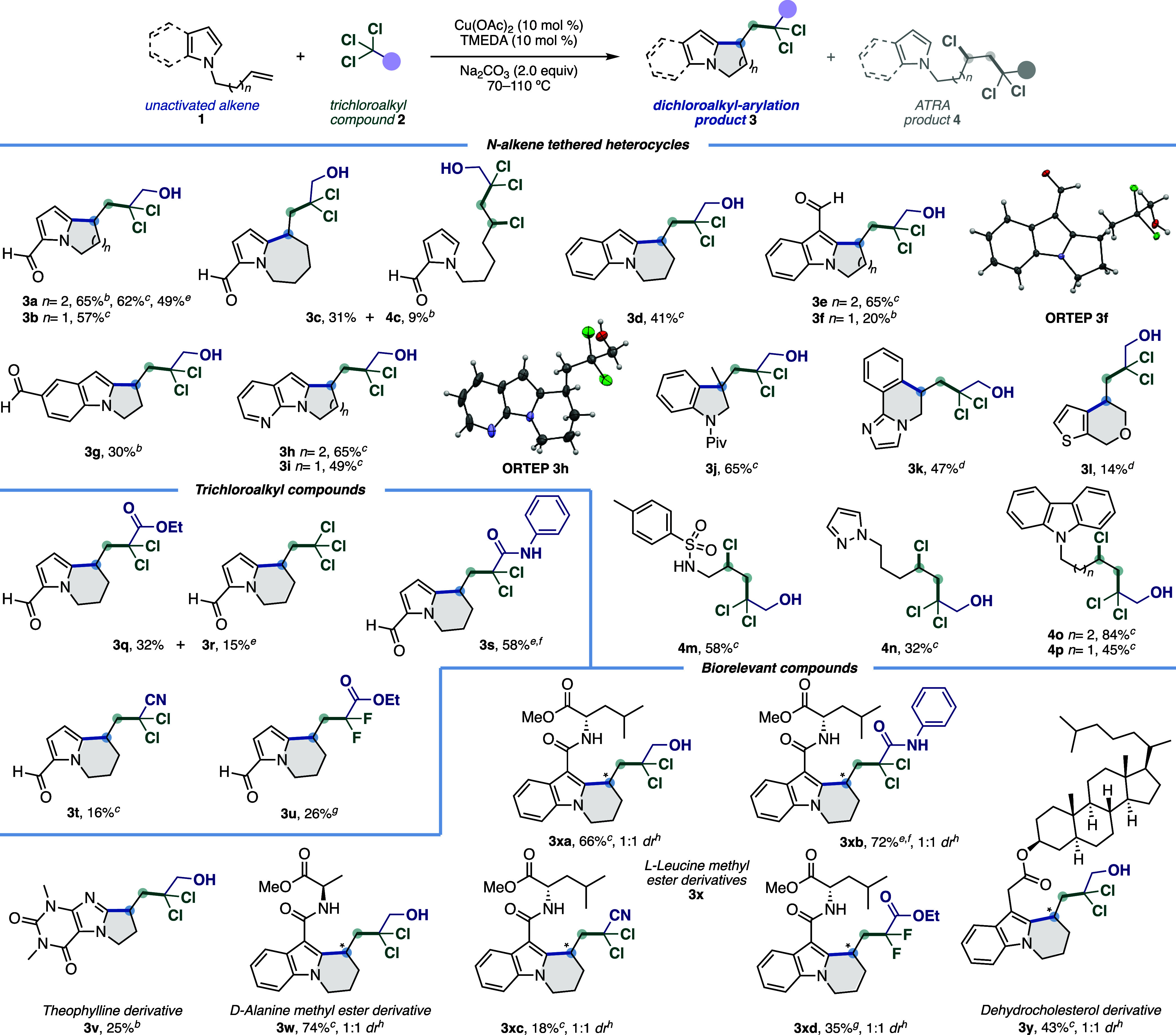
Scope of the Copper-Catalyzed Coupling between *N*-Alkene-Tethered Heterocycles 1 and Trichloroalkyl Compounds **2** ^h^

a Cited yields are of isolated material.
b As in entry 10 of Table 1.
c As in entry 17 of Table 1. Reaction conditions modified from entry 17 of Table 1.
d Run for 2 h.
e With 3.0 equiv of trichloroalkyl compound **2** in DMF (0.4 M) for 3 h.
f Run for 1 h.
g Run for 12 h.
h Measured by NMR of the crude reaction mixture.

After the approach with neat TCE was demonstrated, we evaluated the use of 3.0 equiv of TCE and DMF (0.4 M) as solvent with alkene **1a**, which still afforded the dichloroalkyl/arylation product **3a** in 49% yield. The neat approach is more efficient; however, the solvent approach allows the use of nonbulk trichloroalkyl compounds, such as ethyl 2,2,2-trichloroacetate (**2b**) and 2,2,2-trichloro-*N*-phenylacetamide (**2c**). Thus, we studied the scope of optimized reaction conditions with trichloralkyl compounds **2b**–**e** as precursors stabilized *gem*-dihaloalkyl radicals using alkene **1a** as the model substrate. Thus, an experiment performed using 3 equiv of **2b** in DMF as solvent afforded the mixture of indolizidines **3q** and **3r** in 32% and 15% yields, respectively. The latter product is formed from an interesting decarboxylative process. Similarly, using DMF as a solvent, treatment of **1a** with 3 equiv of **2c** led to the desired unprotected amide **3s** in 58% yield. On the other hand, under the neat approach, the use of trichloroacetonitrile (**2d**) and ethyl chlorodifluoroacetate (**2e**) led to the product **3t** and the *gem*-difluorinated derivative **3u**, respectively.

To demonstrate the applicability of copper-catalyzed radical cascade cyclization with trichloroalkyl compounds **2** for the late-stage modification of biorelevant molecules, we subjected the derivatives of theophylline (**1v**), d-alanine methyl ester (**1w**), l-leucine methyl ester (**1x**), and dehydrocholesterol (**1y**) to the optimized reaction conditions, delivering the radical cyclization products **3v**–**y** with high chemo- and regioselectivity. While indolizidines **3w**, **3xa**, **3xb**, and **3y** synthesized from TCE (**1a**) and 2,2,2-trichloro-*N*-phenylacetamide (**2c**) were obtained in moderate to good yields (43–74%), indolizidines **3xc** and **3xd** synthesized from trichloroacetonitrile (**2d**) and ethyl chlorodifluoroacetate (**2e**) were obtained in lower yields (18–35%).

It is noteworthy that the cyclization products (**3q**, **3r**, **3t**, **3u**, **3xc**, **3xd**) obtained with lower yields (15–35%) correspond to those prepared using radical precursors **2b** and **2c**. In this regard, precursors containing electron-withdrawing groups showed lower reactivity under reaction conditions than TCE (**2a**) (with the exception of the amide-derived precursor **2d**, which afforded **3s** and **3xb** in 58–72%). However, the radical cascade cyclization occurred with high selectivity, as NMR analysis of the crude reaction mixtures showed no presence of dichloroethanol, byproduct formation was negligible, and unreacted starting material was recovered. We hypothesize a mismatch of the redox potentials between the copper catalytic system and the radical precursors **2b** and **2c**.

The pyrrolizidine and indolizidine cores obtained in this work are prevalent structural motifs in *Aspidosperma* alkaloids,^17^ so their subsequent derivatization may pave the way for the development of new synthesis routes, and thus for this purpose, the conversion of the *gem*-dichloride group was studied (Scheme 2a). Treatment of product **3h** with NaOH in MeOH led to corresponding ketone **5a** in 57% yield. This outcome allows us to envision our protocol as a practical strategy to access elusive acetyl radicals for the functionalization of π systems using TCE as a masked precursor. On the other hand, treatment with *t*-BuOK of the TBDMS ether **5b** obtained from **3h** via an iodine-accelerated alcohol protection reaction^18^ resulted in an intramolecular cyclopropanation reaction leading to the spirocyclopropane derivative **5c** in 73% yield.

**Scheme 2:**
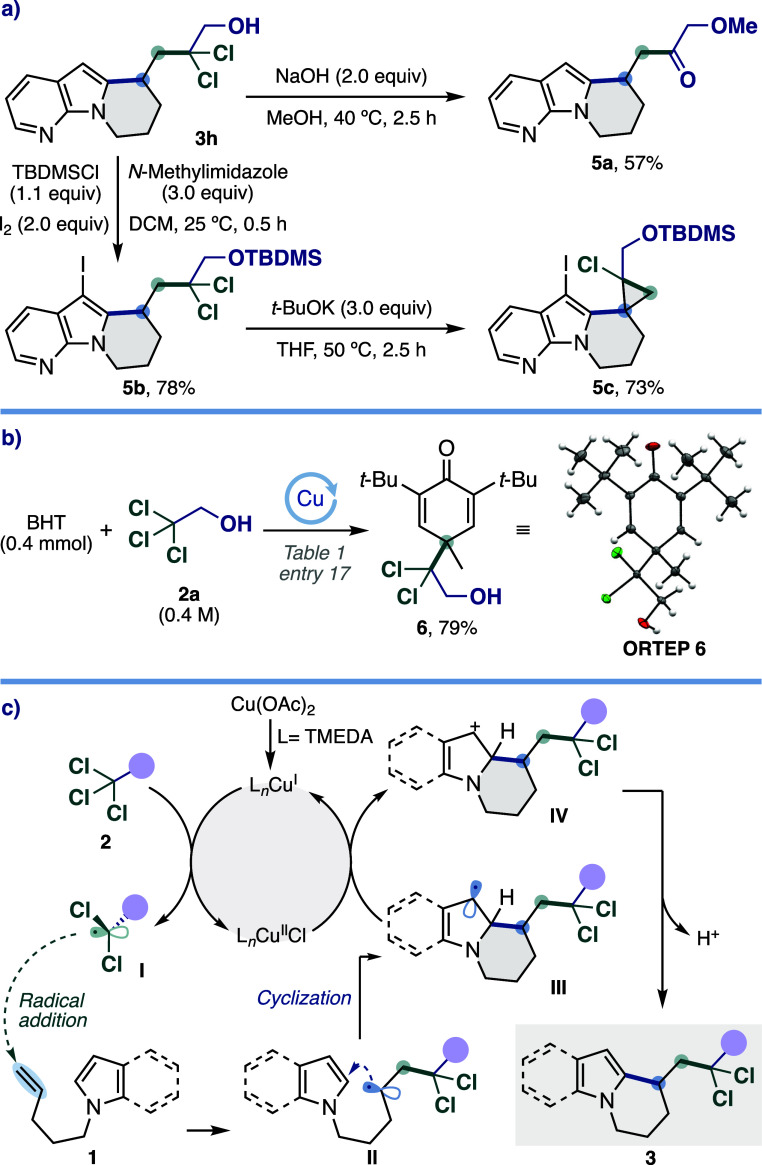
(a) Derivatization Reactions, (b) Radical Trapping Experiment, and (c) Proposed Reaction Mechanism

To gain insight into the reaction mechanism, a radical trapping experiment was performed with BHT (2,6-di-*tert*-butyl-4-methylphenol), obtaining the adduct **6** in 79% yield (Scheme 2b), suggesting that a radical pathway is involved under the copper catalytic system, with *gem*-dichloroalkyl radicals being generated by selective reduction of the trichloromethyl group in TCE (**2a**). Based on these experimental results, a possible mechanism for this dichloroalkyl-arylation reaction is proposed in Scheme 2c. First, the Cu­(I) complex formed *in situ* from Cu­(OAc)_2_ and TMEDA performs a single electron transfer (SET) to the trichloroalkyl compound **2** to generate the *gem*-dichloroalkyl radical **I** and the Cu­(II) species. Next, intermolecular radical addition of **I** on alkene **1** gives rise to secondary radical **II**, and after intramolecular cyclization on the heteroarene, intermediate radical **III** is formed. The SET oxidation of **III** by Cu­(II) species affords intermediate **IV**, regenerating the Cu­(I) species and thus completing the catalytic cycle. Finally, the deprotonation of **IV** promoted by the presence of Na_2_CO_3_ yields product **3**.

## Conclusion

In summary, we have disclosed a copper-catalyzed *gem*-dichloroalkyl radical cascade cyclization of terminal alkenes tethered to *N*-heteroarenes via the *gem*-dichloroalkyl-arylation reaction of unactivated alkenes with trichloroalkyl compounds. The unprecedented copper-catalyzed generation of β-hydroxyalkyl radicals from readily available TCE and their selective addition to unactivated alkenes for the construction of a series of highly functionalized azaheteropolycyclic scaffolds via a tandem process was particularly noteworthy. This reaction sequence affords indolizidine, pyrrolizidine, indoline, imidazo­[2,1-*a*]­isoquinoline, and thieno­[2,3-*c*]­pyran nuclei containing polychloroalkyl groups, which are key intermediates in the total synthesis of natural products. This protocol was also compatible with stabilized *gem*-dihaloalkyl radicals containing ester, amide, and nitrile groups and enabled late-stage diversification of biorelevant compounds, offering alternative access to novel polychlorinated derivatives of bioactive molecules. Furthermore, the practical conversion of the *gem*-dichloride moiety present in indolizidine products to the carbonyl group paves the way for the development of new protocols using TCE as a masked acetyl radical precursor for the functionalization of π systems. Further exploration of these pathways is underway in our laboratory.

## Experimental Section

### General Information

^1^H and ^13^C NMR spectra were obtained on JEOL Eclipse 300 MHz, Bruker Avance III 400 MHz, Bruker Avance III HD 500 and 700 MHz spectrometers. Chemical shifts (δ) are reported in parts per million (ppm) relative to the residual proton signal of CHCl_3_ (δ 7.26) for ^1^H NMR and CDCl_3_ (δ 77.16) for ^13^C NMR. Coupling constants (*J*) are reported in Hertz. Peak assignments of the ^1^H and ^13^C NMR spectra were confirmed using 2D NMR experiments (COSY, TOCSY, HSQC, and HMBC). HRMS was determined on a JEOL AccuTOF JMS-T100LC with an ionSense DART controller ionization source (HRMS-DART), an Agilent 6530 Accurate-Mass Q-TOF LC/MS spectrometer (HRMS-ESI), or a JEOL JMS-700 MStation at 70 eV (HRMS-EI), as specified. Infrared spectra were recorded on a Bruker Tensor 27 FT-IR spectrophotometer. X-ray crystallographic structures for **3f**, **3h**, and **6** were obtained on a Bruker D8 Venture diffractometer using Cu Kα radiation (λ = 1.5417 Å). Melting points were determined on a Fisher apparatus and are uncorrected. Thin layer chromatograms were performed on precoated TLC sheets of silica gel 60 F_254_ (E. Merck). Flash chromatography was carried out using silica gel (Merck 230–400 mesh). All reactions were carried out under an argon atmosphere in oven- or flame-dried glassware unless the reaction procedure states otherwise. Degassed solutions were obtained by freeze–pump–thaw cycles (3×) using liquid nitrogen. All reagents and solvents were purchased from Sigma-Aldrich and Tecsiquim and were used without further purification.

#### Synthesis of Methyl (1-(Pent-4-en-1-yl)-1*H*-indole-3-carbonyl)-d-alaninate (**1w**)

The procedure described by Ngai and co-workers was followed.^19^ An oven-dried 25 mL round-bottom flask charged with a stir bar, NaH (60% in mineral oil, 400 mg, 10.0 mmol, 5.0 equiv), and DMF (0.2 M) was cooled to 0 °C under a nitrogen atmosphere. Indole-3-carboxylic acid (322 mg, 2.0 mmol, 1.0 equiv) was added in one portion, and the resulting mixture was stirred at 0 °C for 30 min, followed by the addition of 5-bromo-1-pentene (471 mg, 3.0 mmol, 1.5 equiv). The reaction mixture was allowed to warm to room temperature. After being stirred for 16 h, the reaction mixture was quenched with water and washed with diethyl ether. The aqueous layer was acidified with 6.0 M HCl to pH 2.0, extracted with ethyl acetate, and the organic layer was washed with brine, dried over anhydrous Na_2_SO_4_, filtered, and concentrated under reduced pressure. The residue was used directly without further purification. A round-bottom flask was charged with *N*-alkylated indole-3-carboxylic acid (1.0 equiv), DCM (0.2 M), HATU (1.57 g, 4.0 mmol, 2.0 equiv), Et_3_N (1.11 mL, 8.0 mmol, 4.0 equiv), and DMAP (24 mg, 0.2 mmol, 10.0 mol %). The resulting mixture was cooled to 0 °C and stirred for 30 min. *D*-alanine ethyl ester hydrochloride (313 mg, 2.2 mmol, 1.1 equiv) was added subsequently. The reaction mixture was allowed to warm to room temperature while being stirred for 16 h. After completion, the reaction mixture was concentrated, and the residue was purified by flash column chromatography on silica gel with DCM/EtOAc (96:4) as eluent to afford the desired product **1w** (247 mg, 0.786 mmol, 39%) as a pale-yellow solid. mp: 100–104 °C. [∝]_D_^25^= −28.3 ° (*c* = 0.01, DCM). ^1^H NMR (400 MHz, CDCl_3_): δ 8.07–7.99 (m, 1H), 7.73 (s, 1H), 7.38–7.33 (m, 1H), 7.30–7.24 (m, 2H), 6.62 (bs, 1H), 5.78 (ddt, *J* = 17.0, 10.4, 6.5 Hz, 1H), 5.10–4.99 (m, 2H), 4.94–4.82 (m, 1H), 4.12 (td, *J* = 7.1, 2.1 Hz, 2H), 3.81 (s, 3H), 2.07 (q, *J* = 7.1, 6.5 Hz, 2H), 1.95 (p, *J* = 7.0 Hz, 2H), 1.56 (d, *J* = 7.2 Hz, 3H). ^13^C­{^1^H} NMR (100 MHz, CDCl_3_): δ 174.4, 164.6, 134.0, 136.7, 131.8, 125.6, 122.6, 121.7, 120.4, 116.1, 110.5, 110.4, 52.6, 48.2, 46.2, 30.8, 29.0, 19.1. FT-IR (ATR) ν_max_: 3310, 3101, 2982, 2936, 1729, 1617, 1535, 1457, 1388, 1337, 1288, 1258, 1235, 1184, 1162, 1049, 1013, 995, 920, 851, 781, 750, 740, 629, 558, 534, 429 cm^–1^. HRMS-(ESI) (*m*/*z*) calcd for C_18_H_23_N_2_O_3_ [M + H]^+^: 315.1709; found: 315.1710.

#### Procedure for the Synthesis of Dichloroalkyl-Arylation Products **3** and/or ATRA Products **4**

A microwave reaction vial equipped with a stir bar was charged with *N*-alkene-tethered heterocycle **1**, Cu­(OAc)_2_ (10 mol %), TMEDA (10 mol %), and Na_2_CO_3_ (2.0 equiv). The trichloroalkyl compound 2 was charged as the solvent (0.4 M) or using 3.0 equiv and DMF as the solvent (0.4 M), as specified. The vial was sealed with a PTFE-lined butyl rubber septum and aluminum crimp cap, and the solution was degassed by three consecutive freeze–pump–thaw cycles using liquid nitrogen and backfilled with pure argon. The mixture was stirred in an oil bath at 70 °C for 1.5 h or 110 °C for 15 min, as specified or unless the reaction procedure states otherwise. After cooling to room temperature, the crude reaction mixture was extracted with a saturated solution of NaHCO_3_ and EtOAc. The organic phase was dried over anhydrous Na_2_SO_4_ and evaporated under reduced pressure. The residue was purified by flash column chromatography on silica gel to afford the desired dichloroalkyl-arylation product **3** and/or ATRA product **4**.

#### 8-(2,2-Dichloro-3-hydroxypropyl)-5,6,7,8-tetrahydroindolizine-3-carbaldehyde (**3a**) (1 mmol Scale)

A microwave reaction vial equipped with a stir bar was charged with 1-(pent-4-en-1-yl)-1*H*-pyrrole-2-carbaldehyde (**1a**) (163 mg, 1.0 mmol), Cu­(OAc)_2_ (19 mg, 0.1 mmol), TMEDA (12 mg, 0.1 mmol), Na_2_CO_3_ (212 mg, 2.0 mmol), and TCE (**2a**) (2.4 mL). The vial was sealed with a PTFE-lined butyl rubber septum and aluminum crimp cap, and the solution was degassed by three consecutive freeze–pump–thaw cycles using liquid nitrogen and backfilled with pure argon. The mixture was stirred at 110 °C in an oil bath for 15 min. After being cooled to room temperature, the crude reaction mixture was extracted with a saturated solution of NaHCO_3_ (40 mL) and EtOAc (50 mL). The organic phase was dried over anhydrous Na_2_SO_4_ and evaporated under reduced pressure. The residue was purified by flash column chromatography on silica gel with hexane/EtOAc (8:2) as eluent to afford the dichloroalkyl-arylation product **3a** (185 mg, 0.67 mmol, 67%) as a violet oil (0.4 mmol scale). Following the general procedure above, we used 1-(pent-4-en-1-yl)-1*H*-pyrrole-2-carbaldehyde (**1a**) (67 mg, 0.41 mmol), Cu­(OAc)_2_ (8 mg, 0.041 mmol), TMEDA (5 mg, 0.041 mmol), Na_2_CO_3_ (87 mg, 0.82 mmol), and TCE (**2a**) (1.0 mL). The mixture was stirred at 110 °C in an oil bath for 15 min. The crude reaction mixture was purified by flash column chromatography on silica gel with hexane/EtOAc (8:2) as eluent to afford the dichloroalkyl-arylation product **3a** (70 mg, 0.253 mmol, 62%) as a violet oil. ^1^H NMR (400 MHz, CDCl_3_): δ 9.44 (s, 1H), 6.89 (d, *J* = 4.2 Hz, 1H), 6.18 (dd, *J* = 4.1, 0.9 Hz, 1H), 4.53 (dt, *J* = 13.9, 5.1 Hz, 1H), 4.21 (ddd, *J* = 14.3, 9.5, 5.3 Hz, 1H), 4.00, 3.95 (AB system, *J* = 12.9 Hz, 2H), 3.38–3.30 (m, 1H), 2.80 (dd, *J* = 15.3, 2.9 Hz, 1H), 2.47 (dd, *J* = 15.3, 7.2 Hz, 1H), 2.37–2.28 (m, 1H), 2.09 (dddt, *J* = 16.1, 7.1, 4.8, 2.4 Hz, 1H), 2.00–1.88 (m, 1H), 1.74 (dddd, *J* = 13.4, 10.7, 8.9, 2.8 Hz, 1H). ^13^C­{^1^H} NMR (100 MHz, CDCl_3_): δ 178.9, 143.6, 131.2, 124.6, 107.9, 93.4, 72.7, 49.1, 45.7, 32.4, 27.9, 21.8. FT-IR (ATR) ν_max_: 3320, 2927, 2865, 2791, 2718, 1723, 1634, 1490, 1468, 1437, 1398, 1319, 1265, 1202, 1163, 1129, 1070, 1039, 966, 938, 785, 734, 712, 681, 646, 626, 597, 566, 441, 409 cm^–1^. HRMS-(DART) (*m*/*z*) calcd for C_12_H_16_Cl_2_NO_2_ [M + H]^+^: 276.0558; found: 276.0545.

#### 1-(2,2-Dichloro-3-hydroxypropyl)-2,3-dihydro-1*H*-pyrrolizine-5-carbaldehyde (**3b**)

Following the general procedure above, we used 1-(but-3-en-1-yl)-1*H*-pyrrole-2-carbaldehyde (**1b**) (61 mg, 0.41 mmol), Cu­(OAc)_2_ (8 mg, 0.041 mmol), TMEDA (5 mg, 0.041 mmol), Na_2_CO_3_ (87 mg, 0.82 mmol), and TCE (**2a**) (1.0 mL). The mixture was stirred at 110 °C in an oil bath for 15 min. The crude reaction mixture was purified by flash column chromatography on silica gel with hexane/EtOAc (8:2) as eluent to afford the dichloroalkyl-arylation product **3b** (61 mg, 0.233 mmol, 57%) as a brown oil. ^1^H NMR (400 MHz, CDCl_3_): δ 9.38 (s, 1H), 6.94 (d, *J* = 4.0 Hz, 1H), 6.03 (dd, *J* = 3.9, 0.9 Hz, 1H), 4.46 (ddd, *J* = 11.9, 8.9, 3.0 Hz, 1H), 4.19–4.09 (m, 1H), 4.00, 3.96 (AB system, *J* = 12.3 Hz, 2H), 3.61 (qd, *J* = 8.3, 3.1 Hz, 1H), 3.00 (bs, 1H), 2.95 (dtd, *J* = 13.1, 7.7, 3.1 Hz, 1H), 2.84 (dd, *J* = 15.1, 3.2 Hz, 1H), 2.49–2.36 (m, 2H). ^13^C­{^1^H} NMR (100 MHz, CDCl_3_): δ 178.6, 149.8, 128.5, 126.5, 103.0, 92.9, 72.6, 47.7, 47.3, 36.2, 34.6. FT-IR (ATR) ν_max_: 3301, 2931, 2857, 2792, 2723, 1635, 1529, 1457, 1444, 1413, 1359, 1256, 1166, 1130, 1074, 1031, 803, 765, 721, 649, 597, 560 cm^–1^. HRMS-(EI) (*m*/*z*) calcd for C_11_H_13_Cl_2_NO_2_ [M]^+^: 261.0323; found: 261.0318.

#### 9-(2,2-Dichloro-3-hydroxypropyl)-6,7,8,9-tetrahydro-5*H*-pyrrolo­[1,2-*a*]­azepine-3-carbaldehyde (**3c**) and 1-(5,7,7-Trichloro-8-hydroxyoctyl)-1*H*-pyrrole-2-carbaldehyde (**4c**)

Following the general procedure above, we used 1-(hex-5-en-1-yl)-1*H*-pyrrole-2-carbaldehyde (**1c**) (59 mg, 0.332 mmol), Cu­(OAc)_2_ (6 mg, 0.033 mmol), TMEDA (4 mg, 0.033 mmol), Na_2_CO_3_ (70 mg, 0.67 mmol), and TCE (**2a**) (0.81 mL). The mixture was stirred at 70 °C in an oil bath for 1.5 h. The crude reaction mixture was purified by flash column chromatography on silica gel with hexane/EtOAc (8:2) as eluent to afford the dichloroalkyl-arylation product **3c** (30 mg, 0.103 mmol, 31%) as a brown oil, and the ATRA product **4c** (10 mg, 0.031 mmol, 9%) as a brown oil. **3c**. ^1^H NMR (400 MHz, CDCl_3_): δ 9.38 (s, 1H), 6.79 (d, *J* = 4.0 Hz, 1H), 6.09 (d, *J* = 4.0 Hz, 1H), 4.72 (bs, 1H), 3.89, 3.83 (AB system, *J* = 12.5 Hz, 2H), 3.42 (bs, 1H), 2.90 (dd, *J* = 15.0, 6.3 Hz, 1H), 2.68 (bs, 1H), 2.58 (bs, 1H), 2.52 (dd, *J* = 15.0, 5.3 Hz, 1H), 2.07–1.63 (m, 6H). ^13^C­{^1^H} NMR (100 MHz, CDCl_3_): δ 179.5, 148.9, 131.6, 125.3, 109.3, 93.8, 72.3, 45.9, 45.1, 35.3, 33.8, 28.3, 27.5. FT-IR (ATR) ν_max_: 3370, 2926, 2851, 2799, 2736, 1635, 1487, 1468, 1443, 1404, 1349, 1331, 1262, 1220, 1184, 1155, 1080, 1039, 974, 938, 892, 808, 773, 724, 705, 648, 628, 608, 541, 482, 464 cm^–1^. HRMS-(DART) (*m*/*z*) calcd for C_13_H_18_Cl_2_NO_2_ [M + H]^+^: 290.0715; found: 290.0701. **4c**. ^1^H NMR (400 MHz, CDCl_3_): δ 9.52 (d, *J* = 1.0 Hz, 1H), 6.96–6.92 (m, 2H), 6.23 (dd, *J* = 3.9, 2.5 Hz, 1H), 4.39–4.26 (m, 2H), 4.24–4.17 (m, 1H), 4.08, 3.96 (AB system, *J* = 12.6 Hz, 2H), 2.86 (dd, *J* = 15.8, 7.5 Hz, 1H), 2.66 (dd, *J* = 15.8, 3.2 Hz, 1H), 1.94–1.76 (m, 4H), 1.64–1.43 (m, 2H). ^13^C­{^1^H} NMR (100 MHz, CDCl_3_): δ 179.5, 131.5, 131.4, 125.3, 109.9, 91.6, 71.6, 58.1, 51.7, 49.0, 38.8, 30.7, 23.2. FT-IR (ATR) ν_max_: 3396, 2928, 2862, 2766, 2722, 1640, 1525, 1480, 1454, 1402, 1365, 1323, 1217, 1070, 1027, 952, 887, 764, 746, 606, 557, 503 cm^–1^. HRMS-(DART) (*m*/*z*) calcd for C_13_H_19_Cl_3_NO_2_ [M + H]^+^: 326.0481; found: 326.0475.

#### 2,2-Dichloro-3-(6,7,8,9-tetrahydropyrido­[1,2-*a*]­indol-9-yl)­propan-1-ol (**3d**)

Following the general procedure above, we used 1-(pent-4-en-1-yl)-1*H*-indole (**1d**) (76 mg, 0.41 mmol), Cu­(OAc)_2_ (8 mg, 0.041 mmol), TMEDA (5 mg, 0.041 mmol), Na_2_CO_3_ (87 mg, 0.82 mmol), and TCE (**2a**) (1.0 mL). The mixture was stirred at 110 °C in an oil bath for 15 min. The crude reaction mixture was purified by flash column chromatography on silica gel with DCM/hexane (8:2) as eluent to afford the dichloroalkyl-arylation product **3d** (50 mg, 0.168 mmol, 41%) as a violet oil. ^1^H NMR (300 MHz, CDCl_3_): δ 7.55 (d, *J* = 7.0 Hz, 1H), 7.27 (d, *J* = 7.1 Hz, 1H), 7.16 (dt, *J* = 6.9, 1.8, 1.2 Hz, 1H), 7.09 (ddd, *J* = 8.2, 7.0, 1.3 Hz, 1H), 6.38 (bs, 1H), 4.24–4.14 (m, 1H), 4.05–3.88 (m, 3H), 3.52–3.41 (m, 1H), 2.98 (dd, *J* = 15.3, 3.0 Hz, 1H), 2.53 (dd, *J* = 15.3, 7.0 Hz, 1H), 2.48–2.38 (m, 1H), 2.26–2.17 (m, 1H), 2.15–2.03 (m, 1H), 1.82–1.68 (m, 1H). ^13^C­{^1^H} NMR (100 MHz, CDCl_3_): δ 140.7, 136.5, 128.1, 120.9, 120.1, 120.0, 108.9, 97.9, 93.9, 72.8, 49.2, 42.3, 32.8, 29.2, 22.3. FT-IR (ATR) ν_max_: 3364, 3049, 2925, 2865, 1710, 1610, 1575, 1530, 1456, 1414, 1363, 1312, 1228, 1197, 1165, 1070, 1012, 956, 920, 808, 738, 714, 644, 595, 568, 423 cm^–1^. HRMS-(DART) (*m*/*z*) calcd for C_15_H_18_Cl_2_NO [M + H]^+^: 298.0765; found: 298.0754.

#### 9-(2,2-Dichloro-3-hydroxypropyl)-6,7,8,9-tetrahydropyrido­[1,2-*a*]­indole-10-carbaldehyde (**3e**)

Following the general procedure above, we used 1-(pent-4-en-1-yl)-1*H*-indole-3-carbaldehyde (**1e**) (59 mg, 0.277 mmol), Cu­(OAc)_2_ (5 mg, 0.028 mmol), TMEDA (3 mg, 0.028 mmol), Na_2_CO_3_ (59 mg, 0.533 mmol), and TCE (**2a**) (0.68 mL). The mixture was stirred at 110 °C in an oil bath for 15 min. The crude reaction mixture was purified by flash column chromatography on silica gel with hexane/EtOAc (7:3) as eluent to afford the dichloroalkyl-arylation product **3e** (59 mg, 0.181 mmol, 65%) as brown crystals. mp: 155–157 °C. ^1^H NMR (700 MHz, CDCl_3_): δ 10.25 (s, 1H), 8.07 (d, *J* = 7.4 Hz, 1H), 7.36–7.29 (m, 3H), 4.32 (ddd, *J* = 12.5, 6.2, 2.1 Hz, 1H), 4.24 (d, *J* = 12.5 Hz, 1H), 4.16–4.13 (m, 1H), 4.00 (d, *J* = 13.0 Hz, 1H), 3.98–3.93 (m, 1H), 2.84–2.73 (m, 3H), 2.38–2.30 (m, 1H), 2.18–2.13 (m, 1H), 1.93 (tdd, *J* = 13.7, 5.1, 3.0 Hz, 1H). ^13^C­{^1^H} NMR (175 MHz, CDCl_3_): δ 184.6, 149.5, 136.3, 127.1, 123.6, 123.3, 119.3, 111.6, 109.9, 92.0, 71.8, 45.8, 42.7, 30.7, 22.8, 17.4. FT-IR (film) ν_max_: 3307, 2950, 2931, 2866, 2852, 1625, 1610, 1579, 1509, 1476, 1455, 1435, 1400, 1380, 1339, 1314, 1250, 1203, 1167, 1142, 1113, 1083, 1062, 1023, 974, 912, 894, 873, 783, 759, 740, 713, 643, 593, 562, 546, 513, 436 cm^–1^. HRMS-(DART) (*m*/*z*) calcd for C_16_H_18_Cl_2_NO_2_ [M + H]^+^: 326.0714; found: 326.0705.

#### 1-(2,2-Dichloro-3-hydroxypropyl)-2,3-dihydro-1*H*-pyrrolo­[1,2-*a*]­indole-9-carbaldehyde (**3f**)

Following the general procedure above, we used 1-(but-3-en-1-yl)-1*H*-indole-3-carbaldehyde (**1f**) (90 mg, 0.452 mmol), Cu­(OAc)_2_ (8 mg, 0.045 mmol), TMEDA (5 mg, 0.045 mmol), Na_2_CO_3_ (96 mg, 0.903 mmol), and TCE (**2a**) (1.1 mL). The mixture was stirred at 70 °C in an oil bath for 1.5 h. The crude reaction mixture was purified by flash column chromatography on silica gel with hexane/EtOAc (7:3) as eluent to afford the dichloroalkyl-arylation product **3f** (28 mg, 0.09 mmol, 20%) as colorless crystals. mp: 155–158 °C. ^1^H NMR (400 MHz, CDCl_3_): δ 10.14 (s, 1H), 8.03 (d, *J* = 6.0 Hz, 1H), 7.34–7.28 (m, 3H), 4.30–4.22 (m, 2H), 4.18 (ddd, *J* = 10.8, 9.0, 4.6 Hz, 1H), 4.04 (d, *J* = 13.1 Hz, 1H), 3.95–3.88 (m, 1H), 3.18 (dd, *J* = 15.2, 1.3 Hz, 1H), 3.14–3.06 (m, 1H), 2.85 (ddd, *J* = 17.6, 8.2, 4.2 Hz, 1H), 2.54 (dd, *J* = 15.1, 9.5 Hz, 1H). ^13^C­{^1^H} NMR (100 MHz, CDCl_3_): δ 184.2, 155.4, 132.7, 131.4, 123.3, 123.2, 119.7, 110.8, 109.1, 91.9, 71.4, 47.3, 44.1, 36.0, 35.4. FT-IR (ATR) ν_max_: 3403, 3047, 2997, 2952, 2927, 2869, 2851, 2787, 2759, 1980, 1947, 1909, 1873, 1829, 1791, 1642, 1571, 1526, 1473, 1447, 1434, 1398, 1371, 1346, 1306, 1286, 1249, 1169, 1121, 1095, 1077, 1035, 940, 844, 753, 742, 710, 677, 578, 549, 483, 462, 415 cm^–1^. HRMS-(DART) (*m*/*z*) calcd for C_15_H_16_Cl_2_NO_2_ [M + H]^+^: 312.0558; found: 312.0552.

#### 1-(2,2-Dichloro-3-hydroxypropyl)-2,3-dihydro-1*H*-pyrrolo­[1,2-*a*]­indole-7-carbaldehyde (**3g**)

Following the general procedure above, we used 1-(but-3-en-1-yl)-1*H*-indole-5-carbaldehyde (**1g**) (82 mg, 0.412 mmol), Cu­(OAc)_2_ (8 mg, 0.041 mmol), TMEDA (5 mg, 0.041 mmol), Na_2_CO_3_ (87 mg, 0.823 mmol), and TCE (**2a**) (1.0 mL). The mixture was stirred at 70 °C in an oil bath for 1.5 h. The crude reaction mixture was purified by flash column chromatography on silica gel with DCM/hexane (6:4) as eluent to afford the dichloroalkyl-arylation product **3g** (38 mg, 0.122 mmol, 30%) as a violet oil. ^1^H NMR (400 MHz, CDCl_3_): δ 9.98 (s, 1H), 8.06 (s, 1H), 7.70 (dd, *J* = 8.5, 1.6 Hz, 1H), 7.29 (d, *J* = 8.5 Hz, 1H), 6.37 (s, 1H), 4.23 (ddd, *J* = 10.3, 8.7, 2.7 Hz, 1H), 4.08–3.99 (m, 3H), 3.85–3.76 (m, 1H), 3.07 (dtd, *J* = 12.8, 7.4, 2.8 Hz, 1H), 2.99 (dd, *J* = 15.0, 3.2 Hz, 1H), 2.57–2.48 (m, 2H). ^13^C­{^1^H} NMR (100 MHz, CDCl_3_): δ 192.9, 149.2, 136.2, 132.6, 129.3, 126.0, 121.6, 110.0, 94.9, 93.0, 72.7, 48.0, 43.8, 36.7, 34.8. FT-IR (ATR) ν_max_: 3379, 2923, 2873, 2852, 1667, 1602, 1565, 1472, 1449, 1393, 1347, 1291, 1227, 1195, 1123, 1071, 1016, 942, 890, 801, 716, 662, 582, 567, 425 cm^–1^. HRMS-(DART) (*m*/*z*) calcd for C_15_H_16_Cl_2_NO_2_ [M + H]^+^: 312.0558; found: 312.0554.

#### 2,2-Dichloro-3-(6,7,8,9-tetrahydropyrido­[3,2-*b*]­indolizin-6-yl)­propan-1-ol (**3h**)

Following the general procedure above, we used 1-(pent-4-en-1-yl)-1*H*-pyrrolo­[2,3-*b*]­pyridine (**1h**) (76 mg, 0.41 mmol), Cu­(OAc)_2_ (8 mg, 0.041 mmol), TMEDA (5 mg, 0.041 mmol), Na_2_CO_3_ (87 mg, 0.82 mmol), and TCE (**2a**) (1.0 mL). The mixture was stirred at 110 °C in an oil bath for 15 min. The crude reaction mixture was purified by flash column chromatography on silica gel with hexane/EtOAc (6:4) as eluent to afford the dichloroalkyl-arylation product **3h** (80 mg, 0.267 mmol, 65%) as a yellow solid. mp: 40–44 °C. ^1^H NMR (400 MHz, CDCl_3_): δ 8.25 (dd, *J* = 4.8, 1.5 Hz, 1H), 7.82 (dd, *J* = 7.7, 1.5 Hz, 1H), 7.04 (dd, *J* = 7.7, 4.8 Hz, 1H), 6.35 (d, *J* = 1.4 Hz, 1H), 4.50–4.42 (m, 1H), 4.09–3.97 (m, 3H), 3.51–3.43 (m, 1H), 2.96 (dd, *J* = 15.3, 3.0 Hz, 1H), 2.55 (dd, *J* = 15.3, 6.9 Hz, 1H), 2.49–2.41 (m, 1H), 2.28–2.19 (m, 1H), 2.11–1.99 (m, 1H), 1.83–1.72 (m, 1H). ^13^C­{^1^H} NMR (100 MHz, CDCl_3_): δ 147.7, 141.7, 141.7, 127.9, 120.9, 116.2, 96.1, 93.7, 72.7, 49.2, 41.4, 32.9, 29.2, 22.1. FT-IR (ATR) ν_max_: 3200, 2920, 2862, 1941, 1903, 1872, 1714, 1634, 1595, 1574, 1532, 1480, 1433, 1400, 1370, 1304, 1287, 1257, 1217, 1166, 1146, 1114, 1070, 1030, 1004, 955, 934, 831, 804, 765, 740, 713, 650, 594, 539, 494, 449, 428 cm^–1^. HRMS-(DART) (*m*/*z*) calcd for C_14_H_17_Cl_2_N_2_O [M + H]^+^: 299.0717; found: 299.0712.

#### 2,2-Dichloro-3-(7,8-dihydro-6*H*-pyrido­[3,2-*b*]­pyrrolizin-6-yl)­propan-1-ol (**3i**)

Following the general procedure above, we used 1-(but-3-en-1-yl)-1*H*-pyrrolo­[2,3-*b*]­pyridine (**1i**) (71 mg, 0.41 mmol), Cu­(OAc)_2_ (8 mg, 0.041 mmol), TMEDA (5 mg, 0.041 mmol), Na_2_CO_3_ (87 mg, 0.82 mmol), and TCE (**2a**) (1.0 mL). The mixture was stirred at 110 °C in an oil bath for 15 min. The crude reaction mixture was purified by flash column chromatography on silica gel with hexane/EtOAc (1:1) as eluent to afford the dichloroalkyl-arylation product **3i** (57 mg, 0.2 mmol, 49%) as a pale-yellow solid. mp: 150–152 °C. ^1^H NMR (400 MHz, CDCl_3_): δ 8.21 (dd, *J* = 4.9, 1.5 Hz, 1H), 7.83 (dd, *J* = 7.8, 1.5 Hz, 1H), 7.02 (dd, *J* = 7.8, 4.8 Hz, 1H), 6.19 (d, *J* = 1.4 Hz, 1H), 4.42 (ddd, *J* = 10.7, 8.7, 2.8 Hz, 1H), 4.13 (ddd, *J* = 10.7, 8.9, 7.1 Hz, 1H), 4.06, 4.02 (AB system, *J* = 12.3 Hz, 2H), 3.88–3.74 (m, 1H), 3.09–3.00 (m, 1H), 2.98 (dd, *J* = 15.0, 3.2 Hz, 1H), 2.57–2.43 (m, 2H). ^13^C­{^1^H} NMR (100 MHz, CDCl_3_): δ 148.1, 144.1, 141.4, 129.0, 125.8, 115.6, 93.2, 91.4, 72.7, 47.9, 43.1, 36.8, 35.1. FT-IR (ATR) ν_max_: 3129, 2957, 2918, 2897, 1937, 1899, 1861, 1732, 1594, 1571, 1539, 1490, 1442, 1407, 1362, 1297, 1275, 1208, 1151, 1127, 1096, 1078, 1038, 1014, 970, 949, 929, 896, 841, 803, 774, 755, 716, 693, 642, 595, 583, 564, 480, 464, 437 cm^–1^. HRMS-(DART) (*m*/*z*) calcd for C_13_H_15_Cl_2_N_2_O [M + H]^+^: 285.0561; found: 285.0555.

#### 1-(3-(2,2-Dichloro-3-hydroxypropyl)-3-methylindolin-1-yl)-2,2-dimethylpropan-1-one (**3j**)

Following the general procedure above, we used *N*-(2-methylallyl)-*N*-phenylpivalamide (**1j**) (95 mg, 0.41 mmol), Cu­(OAc)_2_ (8 mg, 0.041 mmol), TMEDA (5 mg, 0.041 mmol), Na_2_CO_3_ (87 mg, 0.82 mmol), and TCE (**2a**) (1.0 mL). The mixture was stirred at 110 °C in an oil bath for 15 min. The crude reaction mixture was purified by flash column chromatography on silica gel with hexane/EtOAc (8:2) as eluent to afford the dichloroalkyl-arylation product **3j** (91 mg, 0.264 mmol, 65%) as a brown solid. mp: 111–113 °C. ^1^H NMR (400 MHz, CDCl_3_): δ 8.21 (ddd, *J* = 8.2, 1.1, 0.6 Hz, 1H), 7.22 (ddd, *J* = 8.2, 7.3, 1.5 Hz, 1H), 7.15 (ddd, *J* = 7.5, 1.5, 0.6 Hz, 1H), 7.06 (td, *J* = 7.5, 1.1 Hz, 1H), 4.49 (d, *J* = 10.7 Hz, 1H), 4.19 (d, *J* = 10.7 Hz, 1H), 3.86 (s, 2H), 2.72 (s, 2H), 1.58 (s, 3H), 1.38 (s, 9H). ^13^C­{^1^H} NMR (100 MHz, CDCl_3_): δ 176.8, 143.1, 139.0, 128.4, 124.2, 122.1, 118.8, 91.9, 73.8, 62.5, 50.4, 45.0, 40.4, 27.9, 25.9. FT-IR (ATR) ν_max_: 3399, 3074, 2969, 2936, 2927, 2873, 1620, 1591, 1478, 1407, 1380, 1361, 1276, 1242, 1220, 1203, 1174, 1149, 1095, 1082, 1026, 1005, 994, 950, 922, 873, 851, 808, 757, 733, 702, 664, 628, 602, 554, 486, 449, 430 cm^–1^. HRMS-(DART) (*m*/*z*) calcd for C_17_H_24_Cl_2_NO_2_ [M + H]^+^: 344.1184; found: 344.1169.

#### 2,2-Dichloro-3-(5,6-dihydroimidazo­[2,1-*a*]­isoquinolin-6-yl)­propan-1-ol (**3k**)

Following the general procedure above, we used 1-allyl-2-phenyl-1*H*-imidazole (**1k**) (76 mg, 0.41 mmol), Cu­(OAc)_2_ (8 mg, 0.041 mmol), TMEDA (5 mg, 0.041 mmol), Na_2_CO_3_ (87 mg, 0.82 mmol), and TCE (**2a**) (1.0 mL). The mixture was stirred at 110 °C in an oil bath for 2 h. The crude reaction mixture was purified by flash column chromatography on silica gel with EtOAc/DCM (6:4) as eluent to afford the dichloroalkyl-arylation product **3k** (57 mg, 0.192 mmol, 47%) as an orange oil. ^1^H NMR (400 MHz, CDCl_3_): δ 7.93 (bs, 1H), 7.37–7.27 (m, 3H), 7.00 (bs, 1H), 6.88 (bs, 1H), 4.58 (dd, *J* = 13.1, 2.2 Hz, 1H), 4.17 (dt, *J* = 13.0, 3.6 Hz, 1H), 3.97, 3.92 (AB system, *J* = 12.3 Hz, 2H), 3.66 (dd, *J* = 8.1, 3.8 Hz, 1H), 2.38, 2.25 (ABX system, *J* = 15.4, 8.2, 2.9 Hz, 2H). ^13^C­{^1^H} NMR (100 MHz, CDCl_3_): δ 143.7, 136.9, 129.4, 128.7, 128.3, 127.7, 125.7, 124.0, 120.0, 93.7, 72.8, 47.5, 46.1, 36.2. FT-IR (ATR) ν_max_: 3138, 3111, 3055, 2920, 2849, 1747, 1675, 1608, 1581, 1539, 1502, 1474, 1453, 1424, 1364, 1323, 1279, 1264, 1218, 1178, 1138, 1089, 1074, 1049, 1023, 933, 838, 819, 734, 703, 595, 534, 475, 435 cm^–1^. HRMS-(DART) (*m*/*z*) calcd for C_14_H_15_Cl_2_N_2_O [M + H]^+^: 297.0561; found: 297.0548.

#### 2,2-Dichloro-3-(4,7-dihydro-5H-thieno­[2,3-*c*]­pyran-4-yl)­propan-1-ol (**3l**)

Following the general procedure above, we used 2-((allyloxy)­methyl)­thiophene (**1l**) (63 mg, 0.41 mmol), Cu­(OAc)_2_ (8 mg, 0.041 mmol), TMEDA (5 mg, 0.041 mmol), Na_2_CO_3_ (87 mg, 0.82 mmol), and TCE (**2a**) (1.0 mL). The mixture was stirred at 110 °C in an oil bath for 2 h. The crude reaction mixture was purified by flash column chromatography on silica gel with hexane/EtOAc (8:2) as eluent to afford the dichloroalkyl-arylation product **3l** (15 mg, 0.056 mmol, 14%) as a brown oil. ^1^H NMR (400 MHz, CDCl_3_): δ 7.16 (d, *J* = 5.2 Hz, 1H), 6.71 (d, *J* = 5.2 Hz, 1H), 4.75, 4.70 (ABX system, *J* = 14.5, 1.3, 1.7 Hz, 2H), 4.06 (dd, *J* = 7.1, 4.3 Hz, 2H), 3.96 (d, *J* = 4.6 Hz, 2H), 3.48–3.41 (m, 1H), 2.71, 2.60 (ABX system, *J* = 15.4, 7.2, 2.7 Hz, 2H). ^13^C­{^1^H} NMR (100 MHz, CDCl_3_): δ 137.2, 134.3, 123.9, 123.6, 93.4, 72.8, 70.0, 66.8, 48.2, 33.5. FT-IR (ATR) ν_max_: 3400, 2922, 2851, 1760, 1713, 1449, 1429, 1399, 1313, 1265, 1224, 1168, 1069, 917, 837, 812, 709, 639, 606, 566, 508, 452 cm^–1^. HRMS-(EI) (*m*/*z*) calcd for C_10_H_12_Cl_2_O_2_S [M]^+^: 265.9935; found: 265.9946.

#### 4-Methyl-*N*-(2,4,4-trichloro-5-hydroxypentyl)­benzenesulfonamide (**4m**)

Following the general procedure above, we used *N*-allyl-4-methylbenzenesulfonamide (**1m**) (108 mg, 0.51 mmol), Cu­(OAc)_2_ (9 mg, 0.051 mmol), TMEDA (6 mg, 0.051 mmol), Na_2_CO_3_ (108 mg, 1.02 mmol), and TCE (**2a**) (1.3 mL). The mixture was stirred at 110 °C in an oil bath for 15 min. The crude reaction mixture was purified by flash column chromatography on silica gel with hexane/EtOAc (7:3) as the eluent to afford the ATRA product **4m** (107 mg, 0.297 mmol, 58%) as a colorless oil. ^1^H NMR (300 MHz, CDCl_3_): δ 7.76, 7.33 (AA′BB′ system, *J* = 8.1 Hz, 4H), 5.25–5.07 (m, 1H), 4.33–4.22 (m, 1H), 3.98–3.90 (AB system, *J* = 12.6 Hz, 2H), 3.49–3.37 (m, 1H), 3.30–3.18 (m, 1H), 2.74 (d, *J* = 5.3 Hz, 2H), 2.43 (s, 3H). ^13^C­{^1^H} NMR (75 MHz, CDCl_3_): δ 144.2, 136.7, 130.1, 127.2, 90.8, 71.9, 56.6, 49.7, 48.5, 21.7. FT-IR (ATR) ν_max_: 3479, 3278, 2927, 2874, 1597, 1448, 1417, 1323, 1230, 1154, 1089, 1068, 1018, 942, 899, 812, 707, 660, 549 cm^–1^. HRMS-(DART) (*m*/*z*) calcd for C_12_H_17_Cl_3_NO_3_S [M + H]^+^: 359.9994; found: 359.9983.

#### 2,2,4-Trichloro-7-(1*H*-pyrazol-1-yl)­heptan-1-ol (**4n**)

Following the general procedure above, we used 1-(pent-4-en-1-yl)-1*H*-pyrazole (**1n**) (56 mg, 0.41 mmol), Cu­(OAc)_2_ (8 mg, 0.041 mmol), TMEDA (5 mg, 0.041 mmol), Na_2_CO_3_ (87 mg, 0.82 mmol), and TCE (**2a**) (1.0 mL). The mixture was stirred at 110 °C in an oil bath for 15 min. The crude reaction mixture was purified by flash column chromatography on silica gel with hexane/EtOAc (7:3) as the eluent to afford the ATRA product **4n** (37 mg, 0.130 mmol, 32%) as a yellow oil. ^1^H NMR (400 MHz, CDCl_3_): δ 7.50 (d, *J* = 1.3 Hz, 1H), 7.39 (d, *J* = 2.3 Hz, 1H), 6.26–6.24 (m, 1H), 4.25 (ddt, *J* = 8.3, 7.1, 4.0 Hz, 1H), 4.19 (t, *J* = 6.8 Hz, 2H), 4.04, 3.94 (AB system, *J* = 12.5 Hz, 2H), 3.22 (bs, 1H), 2.85, 2.64 (ABX system, *J* = 15.7, 7.1, 3.8 Hz, 2H), 2.22–1.99 (m, 2H), 1.91–1.72 (m, 2H). ^13^C­{^1^H} NMR (100 MHz, CDCl_3_): δ 139.5, 129.3, 105.8, 91.5, 71.7, 57.6, 51.5, 51.2, 36.2, 27.1. FT-IR (ATR) ν_max_: 3218, 2929, 2865, 1718, 1514, 1442, 1398, 1368, 1278, 1207, 1090, 1075, 1055, 949, 918, 881, 752, 718, 652, 617, 600, 558, 479 cm^–1^. HRMS-(DART) (*m*/*z*) calcd for C_10_H_16_Cl_3_N_2_O [M + H]^+^: 285.0328; found: 285.0323.

#### 7-(9*H*-Carbazol-9-yl)-2,2,4-trichloroheptan-1-ol (**4o**)

Following the general procedure above, we used 9-(pent-4-en-1-yl)-9*H*-carbazole (**1o**) (96 mg, 0.41 mmol), Cu­(OAc)_2_ (8 mg, 0.041 mmol), TMEDA (5 mg, 0.041 mmol), Na_2_CO_3_ (87 mg, 0.82 mmol), and TCE (**2a**) (1.0 mL). The mixture was stirred at 110 °C in an oil bath for 15 min. The crude reaction mixture was purified by flash column chromatography on silica gel with hexane/DCM (6:4) as eluent to afford ATRA product **4o** (133 mg, 0.346 mmol, 84%) as a dark green oil. ^1^H NMR (400 MHz, CDCl_3_): δ 8.11 (dd, *J* = 7.8, 0.7 Hz, 2H), 7.48 (ddt, *J* = 8.1, 7.0, 1.1 Hz, 2H), 7.41 (dd, *J* = 8.2, 1.0 Hz, 2H), 7.27–7.22 (m, 2H), 4.38 (t, *J* = 6.9 Hz, 2H), 4.23 (dp, *J* = 11.3, 3.9 Hz, 1H), 4.03, 3.89 (AB system, *J* = 12.6 Hz, 2H), 2.81 (ddd, *J* = 15.8, 7.6, 1.0 Hz, 1H), 2.56 (dd, *J* = 15.6, 3.2 Hz, 1H), 2.25–2.06 (m, 2H), 1.99–1.84 (m, 2H). ^13^C­{^1^H} NMR (100 MHz, CDCl_3_): δ 140.4, 125.9, 123.1, 120.6, 119.2, 108.7, 91.4, 71.7, 57.9, 51.5, 42.4, 36.8, 25.7. FT-IR (ATR) ν_max_: 3410, 3050, 2923, 2850, 1889, 1720, 1626, 1593, 1483, 1451, 1422, 1381, 1325, 1230, 1152, 1120, 1066, 1020, 946, 907, 830, 798, 748, 722, 599, 558, 528, 423 cm^–1^. HRMS-(DART) (*m*/*z*) calcd for C_19_H_21_Cl_3_NO [M + H]^+^: 384.0688; found: 384.0676.

#### 6-(9H-Carbazol-9-yl)-2,2,4-trichlorohexan-1-ol (**4p**)

Following the general procedure above, we used 9-(but-3-en-1-yl)-9*H*-carbazole (**1p**) (75 mg, 0.339 mmol), Cu­(OAc)_2_ (6 mg, 0.034 mmol), TMEDA (4 mg, 0.034 mmol), Na_2_CO_3_ (72 mg, 0.678 mmol), and TCE (**2a**) (0.83 mL). The mixture was stirred at 110 °C in an oil bath for 15 min. The crude reaction mixture was purified by flash column chromatography on silica gel with hexane/DCM (6:4) as eluent to afford ATRA product **4p** (57 mg, 0.154 mmol, 45%) as a dark green oil. ^1^H NMR (400 MHz, CDCl_3_): δ 8.11 (dt, *J* = 7.8, 1.1 Hz, 2H), 7.52–7.45 (m, 4H), 7.28–7.23 (m, 2H), 4.64–4.50 (m, 2H), 4.37–4.29 (m, 1H), 3.99, 3.87 (AB system, *J* = 12.5, 7.0 Hz, 2H), 2.93, 2.65 (ABX system, *J* = 15.7, 6.8, 4.4 Hz, 2H), 2.60–2.50 (m, 1H), 2.38 (t, *J* = 7.7 Hz, 1H), 2.33–2.22 (m, 1H). ^13^C­{^1^H} NMR (100 MHz, CDCl_3_): δ 140.3, 126.0, 123.2, 120.6, 119.3, 108.6, 91.0, 71.9, 56.1, 51.4, 40.1, 38.1. FT-IR (ATR) ν_max_: 3548, 3431, 3051, 3022, 2924, 2852, 1927, 1888, 1853, 1805, 1769, 1705, 1659, 1626, 1595, 1483, 1452, 1380, 1324, 1232, 1184, 1152, 1120, 1064, 1001, 942, 908, 852, 748, 722, 598, 562, 528, 422 cm^–1^. HRMS-(DART) (*m*/*z*) calcd for C_18_H_19_Cl_3_NO [M + H]^+^: 370.0532; found: 370.0519.

#### Ethyl 2,2-Dichloro-3-(3-formyl-5,6,7,8-tetrahydroindolizin-8-yl)­propanoate (**3q**) and 8-(2,2,2-Trichloroethyl)-5,6,7,8-tetrahydroindolizine-3-carbaldehyde (**3r**)

Following the general procedure above, we used 1-(pent-4-en-1-yl)-1*H*-pyrrole-2-carbaldehyde (**1a**) (67 mg, 0.41 mmol), Cu­(OAc)_2_ (8 mg, 0.041 mmol), TMEDA (5 mg, 0.041 mmol), Na_2_CO_3_ (87 mg, 0.82 mmol), ethyl 2,2,2-trichloroacetate (**2b**) (0.235, 1.23 mmol, 3.0 equiv), and DMF (1.0 mL). The mixture was stirred at 110 °C in an oil bath for 3 h. The crude reaction mixture was purified by flash column chromatography on silica gel with hexane/EtOAc (8:2) as eluent to afford polychloroalkyl-arylation products **3q** (42 mg, 0.132 mmol, 32%) and **3r** (17 mg, 0.061 mmol, 15%) as purple oils. **3q**. ^1^H NMR (400 MHz, CDCl_3_): δ 9.44 (s, 1H), 6.89 (d, *J* = 4.2 Hz, 1H), 6.18 (dd, *J* = 4.2, 0.9 Hz, 1H), 4.51 (dt, *J* = 14.0, 5.3 Hz, 1H), 4.32 (q, *J* = 7.1 Hz, 2H), 4.22 (ddd, *J* = 14.1, 9.1, 5.1 Hz, 1H), 3.34–3.25 (m, 1H), 3.01, 2.74 (ABX system, *J* = 15.1, 3.7, 7.6 Hz, 2H), 2.22–2.14 (m, 1H), 2.13–2.03 (m, 1H), 1.97–1.85 (m, 1H), 1.66 (dddd, *J* = 12.9, 10.8, 8.5, 2.6 Hz, 1H), 1.36 (t, *J* = 7.2 Hz, 3H). ^13^C­{^1^H} NMR (100 MHz, CDCl_3_): δ 178.9, 165.9, 142.6, 131.4, 124.5, 108.1, 83.9, 64.3, 50.2, 45.6, 32.6, 27.0, 21.6, 13.9. FT-IR (ATR) ν_max_: 3101, 2921, 2850, 2540, 2391, 1715, 1582, 1465, 1397, 1366, 1327, 1268, 1193, 1172, 1142, 1087, 1036, 895, 867, 773, 661, 627, 579, 440 cm^–1^. HRMS-(DART) (*m*/*z*) calcd for C_14_H_18_Cl_2_NO_3_ [M + H]^+^: 318.0663; found: 318.0674. **3r**. ^1^H NMR (400 MHz, CDCl_3_): δ 9.46 (s, 1H), 6.91 (d, *J* = 4.1 Hz, 1H), 6.21 (dd, *J* = 4.2, 0.8 Hz, 1H), 4.54 (dt, *J* = 14.0, 5.1 Hz, 1H), 4.23 (ddd, *J* = 14.2, 9.3, 5.1 Hz, 1H), 3.42–3.35 (m, 1H), 3.26, 2.99 (ABX system, *J* = 15.3, 2.8, 6.9 Hz, 2H), 2.42–2.33 (m, 1H), 2.10 (ddtd, *J* = 14.7, 7.5, 5.0, 2.8 Hz, 1H), 2.02–1.90 (m, 1H), 1.85–1.75 (m, 1H). ^13^C­{^1^H} NMR (100 MHz, CDCl_3_): δ 179.0, 142.3, 131.4, 124.5, 108.0, 98.8, 60.8, 45.7, 33.8, 27.6, 21.8. FT-IR (ATR) ν_max_: 3043, 2917, 2848, 2439, 1730, 1655, 1580, 1463, 1397, 1322, 1264, 1193, 1140, 1068, 1032, 964, 936, 896, 868, 780, 691, 622, 577 cm^–1^. HRMS-(DART) (*m*/*z*) calcd for C_11_H_13_Cl_3_NO [M + H]^+^: 280.0062; found: 280.0064.

#### 2,2-Dichloro-3-(3-formyl-5,6,7,8-tetrahydroindolizin-8-yl)-*N*-phenylpropanamide (**3s**)

Following the general procedure above, we used 1-(pent-4-en-1-yl)-1*H*-pyrrole-2-carbaldehyde (**1a**) (67 mg, 0.41 mmol), Cu­(OAc)_2_ (8 mg, 0.041 mmol), TMEDA (5 mg, 0.041 mmol), Na_2_CO_3_ (87 mg, 0.82 mmol), 2,2,2-trichloro-*N*-phenylacetamide (**2c**) (0.293, 1.23 mmol, 3.0 equiv), and DMF (1.0 mL). The mixture was stirred at 110 °C in an oil bath for 1 h. The crude reaction mixture was purified by flash column chromatography on silica gel with hexane/EtOAc (8:2) as eluent to afford the dichloroalkyl-arylation product **3s** (87 mg, 0.238 mmol, 58%) as a brown oil. ^1^H NMR (400 MHz, CDCl_3_): δ 9.43 (s, 1H), 8.62 (bs, 1H), 7.57 (dd, *J* = 8.6, 1.2 Hz, 2H), 7.38 (t, *J* = 8.1 Hz, 2H), 7.20 (t, *J* = 7.4 Hz, 1H), 6.89 (d, *J* = 4.2 Hz, 1H), 6.24 (d, *J* = 4.2 Hz, 1H), 4.51 (dt, *J* = 14.1, 5.2 Hz, 1H), 4.18 (ddd, *J* = 14.7, 9.6, 5.4 Hz, 1H), 3.32–3.24 (m, 1H), 3.11, 2.91 (ABX system, *J* = 15.2, 3.3, 7.6 Hz, 2H), 2.24–2.15 (m, 1H), 2.12–2.02 (m, 1H), 1.95–1.82 (m, 1H), 1.71 (qd, *J* = 11.7, 2.5 Hz, 1H). ^13^C­{^1^H} NMR (100 MHz, CDCl_3_): δ 178.9, 163.3, 142.9, 136.6, 131.2, 129.3, 125.8, 124.6, 120.4, 108.2, 86.3, 49.6, 45.6, 32.8, 27.0, 21.7. FT-IR (ATR) ν_max_: 3396, 3305, 3130, 3058, 2926, 2861, 2788, 2723, 1693, 1642, 1597, 1528, 1489, 1441, 1400, 1317, 1298, 1238, 1203, 1178, 1164, 1100, 1038, 983, 904, 859, 821, 785, 753, 689, 636, 567, 504, 455 cm^–1^. HRMS-(ESI) (*m*/*z*) calcd for C_18_H_19_Cl_2_N_2_O_2_ [M + H]^+^: 365.0824; found: 365.0830.

#### 2,2-Dichloro-3-(3-formyl-5,6,7,8-tetrahydroindolizin-8-yl)­propanenitrile (**3t**)

Following the general procedure above, we used 1-(pent-4-en-1-yl)-1*H*-pyrrole-2-carbaldehyde (**1a**) (121 mg, 0.74 mmol), Cu­(OAc)_2_ (14 mg, 0.074 mmol), TMEDA (9 mg, 0.074 mmol), Na_2_CO_3_ (157 mg, 1.48 mmol), and trichloroacetonitrile (**2d**) (1.0 mL). The mixture was stirred at 110 °C in an oil bath for 15 min. The crude reaction mixture was purified by flash column chromatography on silica gel with DCM as eluent to afford the dichloroalkyl-arylation product **3t** (33 mg, 0.122 mmol, 16%) as a dark solid. mp: 68–71 °C. ^1^H NMR (400 MHz, CDCl_3_): δ 9.48 (s, 1H), 6.92 (d, *J* = 4.2 Hz, 1H), 6.16 (d, *J* = 4.2 Hz, 1H), 4.56 (dt, *J* = 14.0, 5.1 Hz, 1H), 4.22 (ddd, *J* = 14.2, 9.4, 5.1 Hz, 1H), 3.42–3.33 (m, 1H), 3.05, 2.79 (ABX system, *J* = 15.2, 3.1, 8.1 Hz, 2H), 2.42–2.33 (m, 1H), 2.12 (ddtd, *J* = 14.6, 7.4, 5.0, 2.7 Hz, 1H), 2.02–1.90 (m, 1H), 1.79 (dddd, *J* = 13.4, 11.2, 8.8, 2.8 Hz, 1H). ^13^C­{^1^H} NMR (100 MHz, CDCl_3_): δ 179.2, 140.7, 131.6, 124.4, 115.8, 107.8, 67.6, 53.5, 45.6, 32.9, 26.7, 21.6. FT-IR (ATR) ν_max_: 3116, 3045, 2968, 2918, 2849, 2218, 1715, 1641, 1488, 1465, 1438, 1402, 1350, 1321, 1265, 1246, 1207, 1169, 1123, 1089, 1035, 976, 939, 821, 777, 724, 684, 644, 548, 498, 454, 406 cm^–1^. HRMS-(DART) (*m*/*z*) calcd for C_12_H_13_Cl_2_N_2_O [M + H]^+^: 271.0405; found: 271.0393.

#### Ethyl 2,2-Difluoro-3-(3-formyl-5,6,7,8-tetrahydroindolizin-8-yl)­propanoate (**3u**)

Following the general procedure above, we used 1-(pent-4-en-1-yl)-1*H*-pyrrole-2-carbaldehyde (**1a**) (67 mg, 0.41 mmol), Cu­(OAc)_2_ (8 mg, 0.041 mmol), TMEDA (5 mg, 0.041 mmol), Na_2_CO_3_ (87 mg, 0.82 mmol), and ethyl chlorodifluoroacetate (**2e**) (1.0 mL). The mixture was stirred at 110 °C in an oil bath for 12 h. The crude reaction mixture was purified by flash column chromatography on silica gel with hexane/EtOAc (9:1) as eluent to afford the dichloroalkyl-arylation product **3u** (30 mg, 0.105 mmol, 26%) as a purple solid. mp: 167–170 °C. ^1^H NMR (400 MHz, CDCl_3_): δ 9.44 (s, 1H), 6.89 (d, *J* = 4.1 Hz, 1H), 6.08 (d, *J* = 4.2 Hz, 1H), 4.55 (dt, *J* = 13.9, 4.9 Hz, 1H), 4.33 (q, *J* = 7.1 Hz, 2H), 4.16 (ddd, *J* = 14.3, 10.0, 5.0 Hz, 1H), 3.21 (dp, *J* = 9.4, 4.6 Hz, 1H), 2.64 (dtd, *J* = 22.1, 15.4, 3.7 Hz, 1H), 2.35–2.24 (m, 1H), 2.22–2.14 (m, 1H), 2.08 (ddtd, *J* = 14.1, 7.1, 4.7, 2.7 Hz, 1H), 1.95–1.83 (m, 1H), 1.61 (dddd, *J* = 13.3, 11.4, 9.3, 2.6 Hz, 1H), 1.36 (t, *J* = 7.1 Hz, 3H). ^13^C­{^1^H} NMR (100 MHz, CDCl_3_): δ 179.0, 164.1 (t, *J*_C–F_ = 32.5 Hz), 142.0, 131.4, 124.4, 115.9 (t, *J*_C–F_ = 251.8 Hz), 107.6, 63.3, 45.6, 39.7 (t, *J*_C–F_ = 22.6 Hz), 29.3, 26.6, 21.7, 14.1. FT-IR (ATR) ν_max_: 3124, 2919, 2850, 2463, 1916, 1745, 1609, 1500, 1468, 1451, 1434, 1401, 1370, 1355, 1317, 1304, 1285, 1252, 1188, 1167, 1129, 1102, 1066, 1048, 1034, 945, 814, 794, 780, 766, 745, 698, 676, 638, 566, 496, 442, 414 cm^–1^. HRMS-(ESI) (*m*/*z*) calcd for C_14_H_18_F_2_NO_3_ [M + H]^+^: 286.1255; found: 286.1250.

#### 8-(2,2-Dichloro-3-hydroxypropyl)-1,3-dimethyl-7,8-dihydro-1*H*-pyrrolo­[2,1-*f*]­purine-2,4­(3*H*,6*H*)-dione (**3v**)

Following the general procedure above, we used 7-(but-3-en-1-yl)-1,3-dimethyl-3,7-dihydro-1*H*-purine-2,6-dione (**1v**) (80 mg, 0.342 mmol), Cu­(OAc)_2_ (6 mg, 0.034 mmol), TMEDA (4 mg, 0.034 mmol), Na_2_CO_3_ (72 mg, 0.683 mmol), and TCE (**2a**) (1.0 mL). The mixture was stirred at 70 °C in an oil bath for 1.5 h. The crude reaction mixture was purified by flash column chromatography on silica gel with EtOAc/DCM (6:4) as eluent to afford the dichloroalkyl-arylation product **3v** (29 mg, 0.084 mmol, 25%) as a pale-yellow solid. mp: 185–189 °C. ^1^H NMR (400 MHz, CDCl_3_): δ 4.40 (ddd, *J* = 11.2, 9.1, 1.4 Hz, 1H), 4.20–4.10 (m, 2H), 3.99 (dd, *J* = 13.0, 1.2 Hz, 1H), 3.51 (s, 3H), 3.46 (qd, *J* = 8.0, 2.1 Hz, 1H), 3.38 (s, 3H), 2.99 (dd, *J* = 15.2, 7.9 Hz, 2H), 2.54 (ddd, *J* = 15.2, 2.9, 1.2 Hz, 1H), 2.42 (dq, *J* = 13.0, 9.5 Hz, 1H). ^13^C­{^1^H} NMR (100 MHz, CDCl_3_): δ 159.5, 154.7, 151.6, 151.3, 106.2, 92.0, 70.3, 46.6, 45.1, 35.1, 33.7, 30.3, 28.2. FT-IR (ATR) ν_max_: 3391, 2920, 2850, 1697, 1652, 1544, 1472, 1425, 1407, 1336, 1297, 1200, 1087, 1050, 973, 948, 831, 746, 724, 700, 652, 603, 559, 527, 510, 492, 441, 418 cm^–1^. HRMS-(DART) (*m*/*z*) calcd for C_13_H_17_Cl_2_N_4_O_3_ [M + H]^+^: 347.0677; found: 347.0675.

#### Methyl (9-(2,2-Dichloro-3-hydroxypropyl)-6,7,8,9-tetrahydropyrido­[1,2-*a*]­indole-10-carbonyl)-d-alaninate (**3w**)

Following the general procedure above, we used methyl (1-(pent-4-en-1-yl)-1*H*-indole-3-carbonyl)-d-alaninate (**1w**) (129 mg, 0.41 mmol), Cu­(OAc)_2_ (8 mg, 0.041 mmol), TMEDA (5 mg, 0.041 mmol), Na_2_CO_3_ (87 mg, 0.82 mmol), and TCE (**2a**) (1.0 mL). The mixture was stirred at 110 °C in an oil bath for 15 min. The crude reaction mixture was purified by flash column chromatography on silica gel with hexane/EtOAc (8:2) as eluent to afford the dichloroalkyl-arylation product **3w** as a 1:1 mixture of diastereomers (129 mg, 0.302 mmol, 74%) as a yellow solid. mp: 132–134 °C. ^1^H NMR (400 MHz, CDCl_3_): δ 7.83 (d, *J* = 7.1 Hz, 1H), 7.80 (d, *J* = 7.1 Hz, 1H), 7.40–7.24 (m, 6H), 6.84 (d, *J* = 7.5 Hz, 2H), 4.85 (p, *J* = 7.2 Hz, 2H), 4.42 (dd, *J* = 12.8, 2.8 Hz, 2H), 4.31 (dd, *J* = 12.1, 6.1 Hz, 2H), 4.19–4.09 (m, 2H), 3.96–3.85 (m, 4H), 3.83 (s, 3H), 3.81 (s, 3H), 2.90–2.72 (m, 6H), 2.44–2.30 (m, 2H), 2.14–2.05 (m, 2H), 1.81 (tq, *J* = 11.1, 3.3 Hz, 2H), 1.57 (d, *J* = 7.1 Hz, 3H), 1.54 (d, *J* = 7.1 Hz, 3H). ^13^C­{^1^H} NMR (100 MHz, CDCl_3_): δ 174.1, 174.1, 165.9, 165.8, 147.3, 147.3, 136.3, 124.7, 124.7, 122.7, 122.6, 122.4, 118.6, 110.2, 103.8, 103.8, 92.4, 70.1, 70.0, 52.8, 52.7, 48.4, 48.3, 45.5, 42.6, 30.6, 30.6, 22.8, 19.2, 19.1, 17.1. FT-IR (ATR) ν_max_: 3380, 2983, 2951, 2921, 2868, 2851, 1741, 1637, 1607, 1520, 1483, 1473, 1452, 1428, 1380, 1360, 1309, 1287, 1206, 1196, 1163, 1126, 1081, 1028, 998, 982, 962, 919, 876, 857, 829, 786, 741, 728, 709, 693, 673, 637, 589, 558, 524, 502, 468, 432, 408 cm^–1^. HRMS-(ESI) (*m*/*z*) calcd for C_20_H_25_Cl_2_N_2_O_4_ [M + H]^+^: 427.1191; found: 427.1195.

#### Methyl (9-(2,2-Dichloro-3-hydroxypropyl)-6,7,8,9-tetrahydropyrido­[1,2-*a*]­indole-10-carbonyl)-l-leucinate (**3xa**)

Following the general procedure above, we used methyl (1-(pent-4-en-1-yl)-1*H*-indole-3-carbonyl)-l-leucinate (**1x**) (146 mg, 0.41 mmol), Cu­(OAc)_2_ (8 mg, 0.041 mmol), TMEDA (5 mg, 0.041 mmol), Na_2_CO_3_ (87 mg, 0.82 mmol), and TCE (**2a**) (1.0 mL). The mixture was stirred at 110 °C in an oil bath for 15 min. The crude reaction mixture was purified by flash column chromatography on silica gel with hexane/EtOAc (8:2) as eluent to afford the dichloroalkyl-arylation product **3xa** as a 1:1 mixture of diastereomers (127 mg, 0.271 mmol, 66%) as a white solid. mp: 145–148 °C. ^1^H NMR (400 MHz, CDCl_3_): δ 7.81 (d, *J* = 7.0 Hz, 1H), 7.77 (d, *J* = 6.8 Hz, 1H), 7.39–7.23 (m, 6H), 6.62 (d, *J* = 8.5 Hz, 1H), 6.59 (d, *J* = 8.6 Hz, 1H), 4.90 (qd, *J* = 8.3, 5.0 Hz, 2H), 4.40 (dd, *J* = 12.8, 2.0 Hz, 2H), 4.35–4.27 (m, 2H), 4.18–4.05 (m, 2H), 3.97–3.84 (m, 4H), 3.81 (s, 3H), 3.78 (s, 3H), 2.91–2.70 (m, 6H), 2.44–2.28 (m, 2H), 2.15–2.03 (m, 2H), 1.89–1.64 (m, 8H), 1.06–0.95 (m, 12H). ^13^C­{^1^H} NMR (100 MHz, CDCl_3_): δ 174.0, 174.0, 166.2, 166.0, 147.2, 147.1, 136.3, 136.2, 124.7, 124.7, 124.7, 122.6, 122.6, 122.3, 122.3, 118.5, 118.5, 110.2, 110.2, 103.9, 103.9, 92.4, 92.4, 70.2, 70.0, 52.6, 52.5, 51.1, 50.9, 45.5, 45.5, 42.6, 42.6, 42.2, 42.1, 30.6, 25.2, 25.2, 23.0, 23.0, 22.8, 22.8, 22.3, 22.3, 17.2, 17.1. FT-IR (ATR) ν_max_: 3519, 3440, 3334, 2954, 2921, 2870, 1723, 1643, 1606, 1525, 1473, 1454, 1427, 1364, 1321, 1259, 1204, 1165, 1108, 1075, 1002, 979, 918, 895, 860, 830, 784, 751, 734, 701, 674, 592, 531, 485, 433 cm^–1^. HRMS-(DART) (*m*/*z*) calcd for C_23_H_31_Cl_2_N_2_O_4_ [M + H]^+^: 469.1660; found: 469.1672.

#### Methyl (9-(2,2-Dichloro-3-oxo-3-(phenylamino)­propyl)-6,7,8,9-tetrahydropyrido­[1,2-*a*]­indole-10-carbonyl)-l-leucinate (**3xb**)

Following the general procedure above, we used methyl (1-(pent-4-en-1-yl)-1*H*-indole-3-carbonyl)-l-leucinate (**1x**) (181 mg, 0.508 mmol), Cu­(OAc)_2_ (9 mg, 0.051 mmol), TMEDA (6 mg, 0.051 mmol), Na_2_CO_3_ (108 mg, 1.016 mmol), 2,2,2-trichloro-*N*-phenylacetamide (**2c**) (363 mg, 1.523 mmol, 3.0 equiv), and DMF (1.0 mL). The mixture was stirred at 110 °C in an oil bath for 1 h. The crude reaction mixture was purified by flash column chromatography on silica gel with hexane/EtOAc (9:1) as eluent to afford the dichloroalkyl-arylation product **3xb** as a 1:1 mixture of diastereomers (203 mg, 0.363 mmol, 72%) as an orange oil. ^1^H NMR (400 MHz, CDCl_3_): δ 9.25 (bs, 2H), 7.86–7.66 (m, 6H), 7.42–7.32 (m, 6H), 7.32–7.26 (m, 4H), 7.18 (q, *J* = 7.3 Hz, 2H), 6.50 (d, *J* = 8.9 Hz, 1H), 6.37 (d, *J* = 8.2 Hz, 1H), 5.01 (td, *J* = 9.0, 4.7 Hz, 1H), 4.90 (td, *J* = 8.3, 5.2 Hz, 1H), 4.38–4.25 (m, 4H), 3.89 (qd, *J* = 11.2, 5.7 Hz, 2H), 3.79 (s, 3H), 3.77 (s, 3H), 3.41 (dd, *J* = 15.2, 4.1 Hz, 2H), 3.12 (ddd, *J* = 24.2, 15.2, 11.2 Hz, 2H), 2.42–2.26 (m, 4H), 2.09–1.99 (m, 2H), 1.90–1.66 (m, 8H), 1.09–0.96 (m, 12H). ^13^C­{^1^H} NMR (100 MHz, CDCl_3_): δ 174.2, 174.1, 165.8, 165.5, 163.1, 145.0, 144.9, 137.5, 137.5, 136.1, 136.1, 129.1, 129.0, 125.2, 125.1, 124.9, 124.9, 122.3, 122.2, 122.2, 122.2, 120.6, 120.6, 118.6, 118.5, 110.1, 105.3, 105.2, 86.7, 86.6, 52.5, 51.1, 50.8, 46.7, 46.5, 42.6, 42.4, 42.3, 41.7, 31.5, 31.2, 25.2, 25.2, 23.1, 22.9, 22.6, 22.3, 22.2, 17.8, 17.7. FT-IR (ATR) ν_max_: 3447, 3357, 3289, 3135, 3053, 2952, 2924, 2870, 2949, 1735, 1686, 1635, 1599, 1526, 1499, 1441, 1365, 1317, 1267, 1237, 1201, 1164, 1011, 977, 901, 829, 783, 750, 734, 689, 639, 594, 568, 557, 505 cm^–1^. HRMS-(ESI) (*m*/*z*) calcd for C_29_H_34_Cl_2_N_3_O_4_ [M + H]^+^: 558.1926; found: 558.1920.

#### Methyl (9-(2,2-Dichloro-2-cyanoethyl)-6,7,8,9-tetrahydropyrido­[1,2-*a*]­indole-10-carbonyl)-l-leucinate (**3xc**)

Following the general procedure above, we used methyl (1-(pent-4-en-1-yl)-1*H*-indole-3-carbonyl)-l-leucinate (**1x**) (146 mg, 0.41 mmol), Cu­(OAc)_2_ (8 mg, 0.041 mmol), TMEDA (5 mg, 0.041 mmol), Na_2_CO_3_ (87 mg, 0.82 mmol), and trichloroacetonitrile (**2d**) (1.0 mL). The mixture was stirred at 110 °C in an oil bath for 15 min. The crude reaction mixture was purified by flash column chromatography on silica gel with hexane/EtOAc (9:1) as eluent to afford the dichloroalkyl-arylation product **3xc** as a 1:1 mixture of diastereomers (35 mg, 0.075 mmol, 18%) as a yellow solid. mp: 115–119 °C. ^1^H NMR (400 MHz, CDCl_3_): δ 7.81 (d, *J* = 7.6 Hz, 2H), 7.38–7.26 (m, 6H), 6.39 (d, *J* = 7.8 Hz, 1H), 6.33 (d, *J* = 8.0 Hz, 1H), 5.01–4.84 (m, 2H), 4.47–4.37 (m, 2H), 4.31 (dd, *J* = 12.4, 5.2 Hz, 2H), 3.92 (td, *J* = 11.7, 5.7 Hz, 2H), 3.78 (s, 3H), 3.77 (s, 3H), 3.39 (dd, *J* = 14.8, 2.9 Hz, 2H), 2.84–2.73 (m, 2H), 2.57–2.47 (m, 2H), 2.33–2.20 (m, 2H), 2.19–2.09 (m, 2H), 2.03–1.91 (m, 2H), 1.85–1.74 (m, 4H), 1.69 (q, *J* = 8.6 Hz, 2H), 1.06–0.97 (m, 12H). ^13^C­{^1^H} NMR (100 MHz, CDCl_3_): δ 174.4, 165.1, 143.4, 136.2, 124.9, 122.4, 122.4, 118.9, 116.3, 110.1, 106.1, 67.1, 52.5, 50.7, 48.6, 42.7, 42.3, 31.5, 25.3, 23.2, 22.3, 22.0, 18.1. FT-IR (ATR) ν_max_: 3436, 3175, 3050, 2954, 2925, 2870, 2851, 1736, 1633, 1535, 1485, 1454, 1433, 1367, 1341, 1321, 1267, 1235, 1210, 1197, 1166, 1120, 1066, 1023, 981, 925, 825, 779, 748, 735, 677, 615, 583, 532, 497, 472, 441 cm^–1^. HRMS-(DART) (*m*/*z*) calcd for C_23_H_28_Cl_2_N_3_O_3_ [M + H]^+^: 464.1508; found: 464.1510.

#### Methyl (9-(3-Ethoxy-2,2-difluoro-3-oxopropyl)-6,7,8,9-tetrahydropyrido­[1,2-*a*]­indole-10-carbonyl)-l-leucinate (**3xd**)

Following the general procedure above, we used methyl (1-(pent-4-en-1-yl)-1*H*-indole-3-carbonyl)-l-leucinate (**1x**) (95 mg, 0.267 mmol), Cu­(OAc)_2_ (5 mg, 0.027 mmol), TMEDA (3 mg, 0.027 mmol), Na_2_CO_3_ (56 mg, 0.533 mmol), and ethyl chlorodifluoroacetate (**2e**) (1.0 mL). The mixture was stirred at 110 °C in an oil bath for 12 h. The crude reaction mixture was purified by flash column chromatography on silica gel with hexane/EtOAc (9:1) as eluent to afford the dichloroalkyl-arylation product **3xd** as a 1:1 mixture of diastereomers (44 mg, 0.092 mmol, 35%) as a yellow oil. *Isomer I*: ^1^H NMR (400 MHz, CDCl_3_): δ 7.83–7.79 (m, 1H), 7.36–7.32 (m, 1H), 7.27 (ddd, *J* = 6.6, 4.3, 1.7 Hz, 2H), 6.30 (d, *J* = 8.5 Hz, 1H), 4.89 (td, *J* = 8.7, 5.1 Hz, 1H), 4.39 (q, *J* = 7.1 Hz, 2H), 4.32–4.16 (m, 2H), 3.89 (td, *J* = 11.5, 5.5 Hz, 1H), 3.78 (s, 3H), 2.86–2.69 (m, 1H), 2.40 (ddt, *J* = 17.5, 15.0, 11.3 Hz, 1H), 2.33–2.17 (m, 2H), 2.13–2.03 (m, 1H), 1.97–1.86 (m, 1H), 1.84–1.74 (m, 2H), 1.73–1.63 (m, 1H), 1.39 (t, *J* = 7.2 Hz, 3H), 1.00 (t, *J* = 6.2 Hz, 6H). ^13^C­{^1^H} NMR (100 MHz, CDCl_3_): δ 174.4, 165.3, 164.2 (t, *J*_C–F_ = 31.6 Hz), 145.1, 136.1, 125.0, 122.1, 122.0, 118.7, 116.3 (t, *J*_C–F_ = 251.6 Hz), 109.9, 105.6, 63.1, 52.4, 50.9, 42.7, 42.1, 37.6 (t, *J*_C–F_ = 22.0 Hz), 27.8, 25.2, 23.5, 23.0, 22.3, 18.0, 14.1. *Isomer II*: ^1^H NMR (400 MHz, CDCl_3_): δ 7.82–7.78 (m, 1H), 7.36–7.32 (m, 1H), 7.29–7.24 (m, 2H), 6.29 (d, *J* = 8.6 Hz, 1H), 4.90 (td, *J* = 8.8, 5.1 Hz, 1H), 4.37 (q, *J* = 7.2 Hz, 2H), 4.28 (ddd, *J* = 12.0, 6.2, 2.2 Hz, 1H), 4.20–4.12 (m, 1H), 3.89 (td, *J* = 11.2, 5.6 Hz, 1H), 3.77 (s, 3H), 2.83 (tdd, *J* = 20.2, 15.0, 3.0 Hz, 1H), 2.41 (ddt, *J* = 17.9, 15.0, 11.3 Hz, 1H), 2.32–2.17 (m, 2H), 2.12–2.03 (m, 1H), 1.96–1.85 (m, 1H), 1.85–1.66 (m, 3H), 1.38 (t, *J* = 7.2 Hz, 3H), 1.02 (d, *J* = 6.1 Hz, 3H), 1.00 (d, *J* = 6.4 Hz, 3H). ^13^C­{^1^H} NMR (100 MHz, CDCl_3_): δ 174.4, 165.2, 164.2 (t, *J*_C–F_ = 32.3 Hz), 145.1, 136.1, 125.0, 122.1, 122.0, 118.8, 116.3 (t, *J*_C–F_ = 251.6 Hz), 109.9, 105.7, 63.1, 52.3, 50.7, 42.7, 42.1, 37.6 (t, *J*_C–F_ = 21.9 Hz), 27.8, 25.3, 23.5, 23.1, 22.2, 17.9, 14.1. FT-IR (ATR) ν_max_: 3443, 2955, 2871, 1761, 1739, 1643, 1531, 1457, 1431, 1368, 1312, 1273, 1232, 1205, 1164, 1122, 1103, 1061, 1019, 982, 955, 923, 852, 792, 776, 739, 688, 652, 629, 561, 510, 474, 439 cm^–1^. HRMS-(ESI) (*m*/*z*) calcd for C_25_H_33_F_2_N_2_O_5_ [M + H]^+^: 479.2358; found: 479.2360.

#### (3*S*,5*S*,8*R*,9*S*,10*S*,13*R*,14*S*,17*R*)-10,13-Dimethyl-17-((*S*)-6-methylheptan-2-yl)­hexadecahydro-1*H*-cyclopenta­[*a*]­phenanthren-3-yl 2-(9-(2,2-dichloro-3-hydroxypropyl)-6,7,8,9-tetrahydropyrido­[1,2-*a*]­indol-10-yl)­acetate (**3y**)

Following the general procedure above, we used methyl (3*S*,5*S*,8*R*,9*S*,10*S*,13*R*,14*S*,17*R*)-10,13-dimethyl-17-((*S*)-6-methylheptan-2-yl)­hexadecahydro-1*H*-cyclopenta­[*a*]­phenanthren-3-yl 2-(1-(pent-4-en-1-yl)-1*H*-indol-3-yl)­acetate (**1y**) (108 mg, 0.176 mmol), Cu­(OAc)_2_ (3 mg, 0.018 mmol), TMEDA (2 mg, 0.018 mmol), Na_2_CO_3_ (38 mg, 0.352 mmol), and TCE (**2a**) (1.0 mL). The mixture was stirred at 110 °C in an oil bath for 15 min. The crude reaction mixture was purified by flash column chromatography on silica gel with hexane/EtOAc (8:2) as eluent to afford the dichloroalkyl-arylation product **3y** as a 1:1 mixture of diastereomers (55 mg, 0.076 mmol, 43%) as a brown oil. ^1^H NMR (400 MHz, CDCl_3_): δ 7.59 (d, *J* = 7.8 Hz, 2H), 7.25 (d, *J* = 7.2 Hz, 2H), 7.17 (td, *J* = 8.1, 7.6, 1.3 Hz, 2H), 7.11 (t, *J* = 7.4 Hz, 2H), 4.68 (tt, *J* = 11.1, 4.9 Hz, 2H), 4.26 (ddd, *J* = 10.5, 5.2, 2.0 Hz, 2H), 4.00 (dd, *J* = 12.3, 1.3 Hz, 2H), 3.95 (d, *J* = 12.9 Hz, 2H), 3.85–3.71 (m, 8H), 2.83 (ddd, *J* = 15.6, 4.5, 2.0 Hz, 2H), 2.69 (dd, *J* = 15.6, 10.2 Hz, 2H), 2.54 (d, *J* = 13.4 Hz, 2H), 1.98–1.93 (m, 2H), 1.83–1.76 (m, 4H), 1.70–1.65 (m, 2H), 1.56–1.49 (m, 8H), 1.38–1.21 (m, 20H), 1.15–1.08 (m, 12H), 1.03–0.94 (m, 10H), 0.92–0.89 (m, 10H), 0.88–0.85 (m, 18H), 0.81 (s, 6H), 0.65–0.63 (m, 8H). ^13^C­{^1^H} NMR (100 MHz, CDCl_3_): δ 172.1, 137.5, 136.1, 128.0, 121.1, 119.9, 118.7, 118.7, 108.9, 103.0, 93.3, 74.6, 72.8, 56.6, 56.4, 54.3, 46.4, 44.8, 44.8, 42.7, 42.4, 40.1, 39.7, 36.9, 36.3, 35.9, 35.6, 35.6, 34.1, 32.1, 30.9, 30.9, 29.4, 28.7, 28.4, 28.2, 27.6, 24.8, 24.3, 24.0, 23.0, 22.7, 21.3, 18.8, 18.6, 12.4, 12.2. FT-IR (ATR) ν_max_: 3442, 3049, 2929, 2865, 1724, 1613, 1565, 1461, 1363, 1332, 1266, 1243, 1164, 1147, 1131, 1075, 1014, 957, 928, 863, 843, 805, 737, 677, 597, 572, 541, 465, 433 cm^–1^. HRMS-(ESI) (*m*/*z*) calcd for C_44_H_66_Cl_2_NO_3_ [M + H]^+^: 726.4420; found: 726.4402.

#### Derivatization of 2,2-Dichloro-3-(6,7,8,9-tetrahydropyrido­[3,2-*b*]­indolizin-6-yl)­propan-1-ol (**3h**)

##### 1-Methoxy-3-(6,7,8,9-tetrahydropyrido­[3,2-*b*]­indolizin-6-yl)­propan-2-one (**5a**)

Following the procedure described by Bao and co-workers.^20^ A round-bottom flask was charged with a mixture of 2,2-dichloro-3-(6,7,8,9-tetrahydropyrido­[3,2-*b*]­indolizin-6-yl)­propan-1-ol (**3h**) (100 mg, 0.334 mmol), NaOH (27 mg, 0.668 mmol), and methanol (2.2 mL). The reaction mixture was stirred at 40 °C for 2.5 h and then cooled to room temperature. The solvent was evaporated and the residue dissolved in EtOAc and washed with water. The organic phase was dried over anhydrous Na_2_SO_4_ and evaporated under reduced pressure. The residue obtained was purified by flash column chromatography on silica gel with hexane/EtOAc (6:4) as eluent to afford ketone **5a** (49 mg, 0.190 mmol, 57%) as a yellow oil. ^1^H NMR (400 MHz, CDCl_3_): δ 8.24 (d, *J* = 4.9 Hz, 1H), 7.80 (d, *J* = 7.8 Hz, 1H), 7.03 (dd, *J* = 7.7, 4.9 Hz, 1H), 6.15 (s, 1H), 4.45 (dt, *J* = 12.3, 4.9 Hz, 1H), 4.09–4.00 (m, 1H), 4.05 (s, 2H), 3.69–3.60 (m, 1H), 3.45 (s, 3H), 3.06 (dd, *J* = 17.6, 5.5 Hz, 1H), 2.79 (dd, *J* = 17.6, 7.8 Hz, 1H), 2.24–2.11 (m, 2H), 2.09–1.98 (m, 1H), 1.60–1.49 (m, 1H). ^13^C­{^1^H} NMR (100 MHz, CDCl_3_): δ 207.2, 141.3, 141.1, 128.0, 121.0, 116.1, 95.6, 78.3, 59.6, 44.4, 41.5, 30.2, 29.8, 27.5, 21.9. FT-IR (ATR) ν_max_: 3045, 2995, 2950, 2925, 2881, 2856, 1910, 1875, 1844, 1723, 1593, 1565, 1525, 1478, 1431, 1399, 1360, 1349, 1306, 1290, 1252, 1191, 1144, 1109, 1037, 1004, 975, 941, 899, 833, 813, 768, 717, 700, 670, 582, 565, 533, 455, 437 cm^–1^. HRMS-(ESI) (*m*/*z*) calcd for C_15_H_19_N_2_O_2_ [M + H]^+^: 259.1447; found: 259.1454.

##### 6-(3-((*tert*-Butyldimethylsilyl)­oxy)-2,2-dichloropropyl)-5-iodo-6,7,8,9-tetrahydropyrido­[3,2-*b*]­indolizine (**5b**)

Following the procedure described by Stawinski and co-workers.^21^ 2,2-dichloro-3-(6,7,8,9-tetrahydropyrido­[3,2-*b*]­indolizin-6-yl)­propan-1-ol (**3h**) (273 mg, 0.912 mmol), *N*-methylimidazole (227 mg, 2.737 mmol), and iodine (463 mg, 1.825 mmol) were dissolved in DCM (2.73 mL). TBDMSCl (156 mg, 1.004 mmol) was added, and the reaction mixture was stirred at rt for 30 min. The solvent was evaporated, the residue dissolved in EtOAc and washed with a saturated solution of Na_2_S_2_O_3_. The organic phase was dried over anhydrous Na_2_SO_4_ and evaporated under reduced pressure. The residue obtained was purified by flash column chromatography on silica gel with hexane/EtOAc (9:1) as eluent to afford the TBDMS ether **5b** (382 mg, 0.708 mmol, 78%) as a yellow solid. mp: 75–78 °C. ^1^H NMR (400 MHz, CDCl_3_): δ 8.28 (dt, *J* = 4.8, 1.4 Hz, 1H), 7.67 (ddd, *J* = 7.9, 3.3, 1.5 Hz, 1H), 7.12 (ddd, *J* = 8.2, 4.8, 2.4 Hz, 1H), 4.60–4.53 (m, 1H), 4.02, 3.99 (AB system, *J* = 10.9 Hz, 2H), 3.97–3.90 (m, 1H), 3.77 (ddt, *J* = 10.9, 5.3, 2.7 Hz, 1H), 2.77, 2.52 (ABX system, *J* = 15.2, 2.9, 11.1 Hz, 2H), 2.74–2.67 (m, 1H), 2.28–2.17 (m, 1H), 2.12–2.03 (m, 1H), 1.91 (ddt, *J* = 17.3, 13.8, 3.4 Hz, 1H), 0.92 (s, 9H), 0.13 (d, *J* = 2.5 Hz, 6H). ^13^C­{^1^H} NMR (100 MHz, CDCl_3_): δ 147.8, 143.0, 141.1, 128.3, 123.6, 116.9, 91.6, 73.7, 53.7, 44.2, 42.0, 31.8, 25.9, 23.5, 18.5, 18.3, −5.1, −5.2. FT-IR (ATR) ν_max_: 2995, 2950, 2925, 2882, 2855, 1910, 1875, 1844, 1739, 1711, 1592, 1564, 1524, 1477, 1431, 1399, 1360, 1350, 1306, 1289, 1251, 1190, 1144, 1113, 1037, 1004, 974, 954, 940, 898, 832, 814, 783, 767, 717, 699, 671, 593, 582, 564, 532, 468, 455, 438 cm^–1^. HRMS-(ESI) (*m*/*z*) calcd for C_20_H_30_Cl_2_IN_2_OSi [M + H]^+^: 539.0549; found: 539.0560.

##### 2-(((*tert*-Butyldimethylsilyl)­oxy)­methyl)-2-chloro-5′-iodo-8′,9′-dihydro-7′*H*-spiro­[cyclopropane-1,6′-pyrido­[3,2-*b*]­indolizine] (**5c**)

Following the procedure described by Rong and co-workers.^22^ The mixture of 6-(3-((*tert*-butyldimethylsilyl)­oxy)-2,2-dichloropropyl)-5-iodo-6,7,8,9-tetrahydropyrido­[3,2-*b*]­indolizine (**5b**) (50 mg, 0.093 mmol), *t*-BuOK (32 mg, 0.278 mmol), and THF (1.9 mL) was added into a round-bottom flask and stirred at 50 °C for 2.5 h under a nitrogen atmosphere. After the reaction was completed, the solution was concentrated under reduced pressure, and the residue obtained was purified by flash column chromatography on silica gel with hexane/EtOAc (9:1) as eluent to afford spirocyclopropane **5c** (34 mg, 0.068 mmol, 73%) as a yellow solid. mp: 100–103 °C. ^1^H NMR (400 MHz, CDCl_3_): δ 8.28 (dd, *J* = 4.8, 1.5 Hz, 1H), 7.64 (dd, *J* = 7.8, 1.5 Hz, 1H), 7.10 (dd, *J* = 7.8, 4.8 Hz, 1H), 4.52–4.44 (m, 1H), 4.37–4.28 (m, 1H), 3.96 (d, *J* = 11.3 Hz, 1H), 3.58 (d, *J* = 11.5 Hz, 1H), 3.13 (d, *J* = 7.5 Hz, 1H), 2.48–2.36 (m, 1H), 2.23–2.04 (m, 3H), 1.30 (d, *J* = 7.6 Hz, 1H), 0.60 (s, 9H), −0.19 (s, 3H), −0.49 (s, 3H). ^13^C­{^1^H} NMR (100 MHz, CDCl_3_): δ 147.9, 143.6, 135.4, 128.9, 124.1, 116.8, 67.6, 56.3, 50.4, 41.2, 30.1, 29.3, 25.5, 23.6, 21.4, 18.0, −5.7, −6.0. FT-IR (ATR) ν_max_: 3045, 2952, 2925, 2888, 2851, 1910, 1875, 1844, 1718, 1589, 1567, 1517, 1479, 1459, 1429, 1396, 1370, 1329, 1304, 1284, 1249, 1195, 1159, 1102, 1061, 1030, 1005, 959, 941, 835, 774, 746, 664, 631, 595, 566, 541, 496, 459, 431 cm^–1^. HRMS-(ESI) (*m*/*z*) calcd for C_20_H_29_ClIN_2_OSi [M + H]^+^: 503.0782; found: 503.0790.

##### Radical Trapping Experiment: 2,6-Di-*tert*-butyl-4-(1,1-dichloro-2-hydroxyethyl)-4-methylcyclohexa-2,5-dien-1-one (**6**)

Following the general procedure above, we used dibutylhydroxytoluene (90 mg, 0.41 mmol), Cu­(OAc)_2_ (8 mg, 0.041 mmol), TMEDA (5 mg, 0.041 mmol), Na_2_CO_3_ (87 mg, 0.82 mmol), and TCE (**2a**) (1.0 mL). The mixture was stirred at 110 °C in an oil bath for 15 min. The crude reaction mixture was purified by flash column chromatography on silica gel with hexane/DCM (1:1) as eluent to afford product **6** (108 mg, 0.324 mmol, 79%) as brown crystals. mp: 125–130 °C. ^1^H NMR (400 MHz, CDCl_3_): δ 6.76 (s, 2H), 3.81 (s, 2H), 1.57 (s, 3H), 1.23 (s, 18H). ^13^C­{^1^H} NMR (100 MHz, CDCl_3_): δ 185.6, 148.5, 140.3, 100.4, 70.5, 48.5, 35.3, 29.5, 23.4. FT-IR (ATR) ν_max_: 3499, 2996, 2952, 2867, 1755, 1735, 1654, 1632, 1482, 1456, 1388, 1371, 1361, 1298, 1245, 1233, 1200, 1173, 1140, 1100, 1013, 932, 878, 809, 731, 718, 654, 580, 550, 510, 440 cm^–1^. HRMS-(DART) (*m*/*z*) calcd for C_17_H_27_Cl_2_O_2_ [M + H]^+^: 333.1388; found: 333.1380.

## Supplementary Material



## Data Availability

The data underlying this study are available in the published article and its Supporting Information.

## Notes

Experimental procedures; ^1^H and ^13^C­{^1^H} NMR spectra of products; and X-ray crystallographic information (PDF)
