# Supplementary material for: Copper-Catalyzed gem-Dichloroalkyl-Arylation of Unactivated Alkenes for the Synthesis of Dichloroalkyl-Azaheteropolycycles
Source: J Org Chem. 2025 Dec 23;91(1):676–88. doi: 10.1021/acs.joc.5c02773 (PMC12797284; doi:10.1021/acs.joc.5c02773)
Supplement: Supplementary file 1 [file jo5c02773_si_001.pdf]

Supporting Information for

Copper-Catalyzed *gem*-Dichloroalkyl-Arylation of Unactivated Alkenes for the Synthesis of Dichloroalkyl-Azaheteropolycycles

Gustavo G. Flores-Bernal, Carlos S. Ulloa-Chacha, Marco T. Espinoza-Nicolás, and Luis D. Miranda*

Instituto de Química, Universidad Nacional Autónoma de México, Circuito Exterior, Ciudad Universitaria, 04510 Mexico City, Mexico.

*E-mail: lmiranda@unam.mx; Tel: +52-55-5622-4420

Table of Contents

Part I Experimental section S2
General information S2
1.1 Procedure for the synthesis of substrates S2
1.2 Procedure for the synthesis of dichloroalkyl-arylation products **3** S3
1.3 Derivatization of 2,2-dichloro-3-(6,7,8,9-tetrahydropyrido[3,2-*b*]indolizin-6-yl)propan-1-ol (**3h**) S10
1.4 Radical trapping experiment S11
Part II X-ray crystallographic information S12
Part III ^{1}H and ^{13}C{^{1}H} NMR spectra S14
References S49

Part I Experimental section

General information

^{1}H and ^{13}C NMR spectra were obtained on JEOL Eclipse 300 MHz, Bruker Avance III 400 MHz, Bruker Avance III HD 500 MHz and 700 MHz spectrometers. Chemical shifts (δ) are reported in parts per million (ppm) relative to residual proton signal of $CHCl_3$ (δ 7.26) for ^{1}H NMR, and $CDCl_3$ (δ 77.16) for ^{13}C NMR. Coupling constants (J) are reported in Hertz. Peak assignments of the ^{1}H and ^{13}C NMR spectra were confirmed by using 2D NMR experiments (COSY, TOCSY, HSQC, and HMBC). HRMS were determined on a JEOL AccuTOF JMS-T100LC with an ionSense DART controller ionization source (HRMS-DART), an Agilent 6530 Accurate-Mass Q-TOF LC/MS spectrometer (HRMS-ESI), or a JEOL JMS-700 MStation at 70 eV (HRMS-EI), as specified. Infrared spectra were recorded on a Bruker Tensor 27 FT-IR spectrophotometer. X-ray crystallographic structures for **3f**, **3h** and **6** were obtained on a Bruker D8 Venture diffractometer using CuKα radiation (λ= 1.5417 Å). Melting points were determined on a Fisher apparatus and are uncorrected. Thin layer chromatograms were performed on precoated TLC sheets of silica gel 60 F_{254} (E. Merck). Flash chromatography was carried out using silica gel (Merck 230–400 mesh). All reactions were carried out under an argon atmosphere in oven- or flame-dried glassware unless the reaction procedure states otherwise. Degassed solutions were obtained by freeze-pump-thaw cycles (3x) using liquid nitrogen. All reagents and solvents were purchased from Sigma-Aldrich and Tecsiquim and were used without further purification.

1.1 Procedure for the synthesis of substrates

N-alkene tethered heterocycles **1** were prepared by *N*- or *O*-alkylation of *N*-heterocycles, amines, or alcohol with alkyl halides according to the corresponding literature methods: **1a**[1], **1b**[2], **1c**[3], (**1d**,**h**,**j**,**l**,**m**,**x**,**y**)[4], **1e**[5], **1f**[6], **1g**[7], **1i**[8], **1k**[9], **1n**[10], **1o**[11], **1p**[12], **1v**[13]. NMR spectra matched previously reported data.

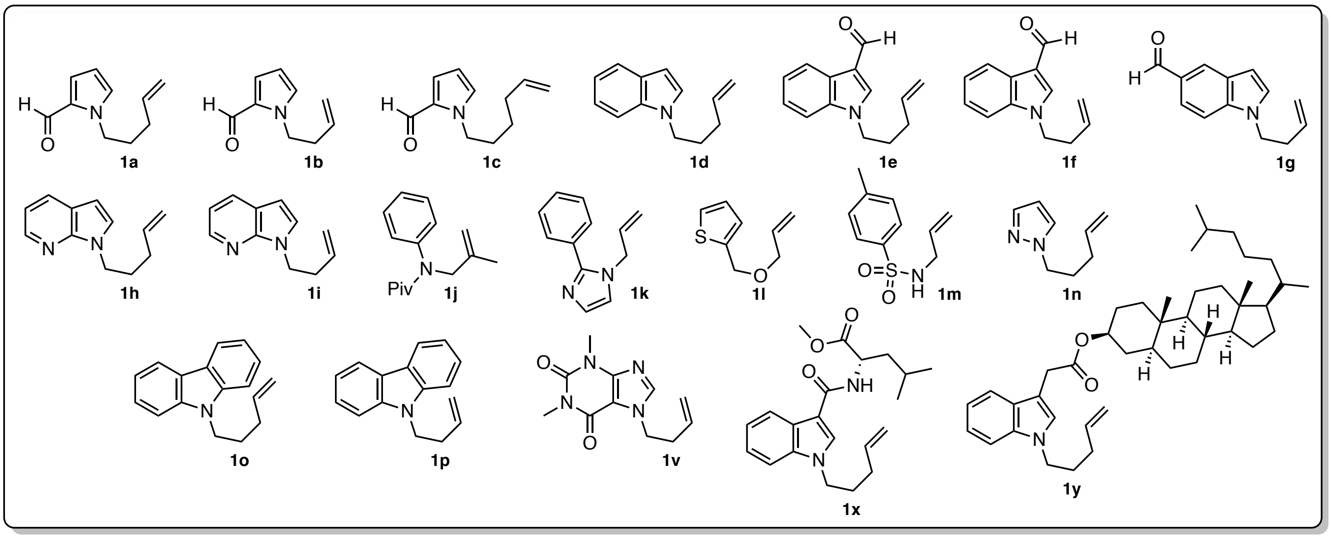

Synthesis of methyl (1-(pent-4-en-1-yl)-1*H*-indole-3-carbonyl)-*D*-alaninate (1w)

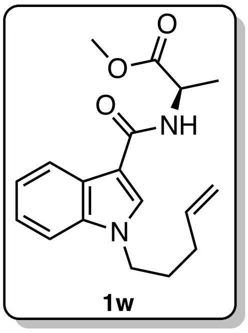

Following the procedure described by Ngai and co-workers.[4] An oven-dried 25 mL round bottom flask charged with a stir bar, NaH (60% in mineral oil, 400 mg, 10.0 mmol, 5.0 equiv.), and DMF (0.2 M) was cooled to 0 ºC under a nitrogen atmosphere. Indole-3-carboxylic acid (322 mg, 2.0 mmol, 1.0 equiv.) was added in one portion, and the resulting mixture was stirred at 0 ºC for 30 min, followed by the addition of 5-bromo-1-pentene (471 mg, 3.0 mmol, 1.5 equiv.). The reaction mixture was allowed to warm to room temperature. After stirring for 16 h, the reaction mixture was quenched with water and washed with diethyl ether. The aqueous layer was acidified with 6.0 M HCl to pH 2.0, extracted with ethyl acetate, and the organic layer was washed with brine, dried over anhydrous Na_2SO_4, filtered, and concentrated under reduced pressure. The residue was used directly without further purification. A round bottom flask was charged with the *N*-alkylated indole-3-carboxylic acid (1.0 equiv.), DCM (0.2 M), HATU (1.57 g, 4.0 mmol, 2.0 equiv.), Et_3N (1.11 mL, 8.0 mmol, 4.0 equiv.), and DMAP (24 mg, 0.2 mmol, 10.0 mol%). The resulting mixture was cooled to 0 °C and stirred for 30 min. *D*-alanine ethyl ester hydrochloride (313 mg, 2.2 mmol, 1.1 equiv.) was added subsequently. The reaction mixture was allowed to warm to room temperature while being stirred for 16 h. After completion, the reaction mixture was concentrated and the residue was purified by flash column chromatography on silica gel with DCM/EtOAc (96:4) as eluent to afford the desired product **1w** (247 mg, 0.786 mmol, 39%) as a pale-yellow solid. mp: 100-104 ºC. $[\alpha]_D^{25}$= –28.3 º (c= 0.01, DCM). **^{1}H NMR** (400 MHz, $CDCl_3$): δ 8.07 – 7.99 (m, 1H), 7.73 (s, 1H), 7.38 – 7.33 (m, 1H), 7.30 – 7.24 (m, 2H), 6.62 (bs, 1H), 5.78 (ddt, J = 17.0, 10.4, 6.5 Hz, 1H), 5.10 – 4.99 (m, 2H), 4.94 – 4.82 (m, 1H), 4.12 (td, J = 7.1, 2.1 Hz, 2H), 3.81 (s, 3H), 2.07 (q, J = 7.1, 6.5 Hz, 2H), 1.95 (p, J = 7.0 Hz, 2H), 1.56 (d, J = 7.2 Hz, 3H). **^{13}C{^{1}H} NMR** (100 MHz, $CDCl_3$): δ 174.4, 164.6, 134.0, 136.7, 131.8, 125.6, 122.6, 121.7, 120.4, 116.1, 110.5, 110.4, 52.6, 48.2, 46.2, 30.8, 29.0, 19.1. **FT-IR** (ATR) ν_{max}: 3310, 3101, 2982, 2936, 1729, 1617, 1535, 1457, 1388, 1337, 1288, 1258, 1235, 1184, 1162, 1049, 1013, 995, 920, 851, 781, 750, 740, 629, 558, 534, 429 cm^{-1}. **HRMS**-(ESI) (m/z) calcd for $C_{18}H_{23}N_2O_3$ $[M+H]^+$: 315.1709; found: 315.1710.

2,2,2-Trichloroethanol (**2a**), trichloroacetonitrile (**2d**), ethyl chlorodifluoroacetate (**2e**) were purchased from Sigma-Aldrich and were used without further purification. Ethyl 2,2,2-trichloroacetate (**2b**) was prepared by esterification of trichloroacetic acid with ethanol[14] and 2,2,2-trichloro-*N*-phenylacetamide (**2c**) was prepared from trichloroacetic anhydride and aniline.[15] NMR spectra matched previously reported data.

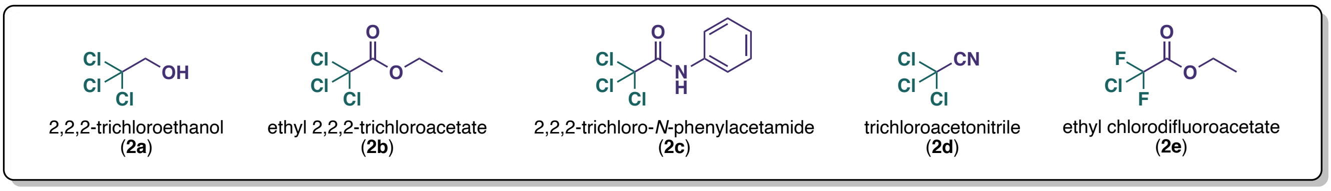

1.2 Procedure for the synthesis of dichloroalkyl-arylation products 3

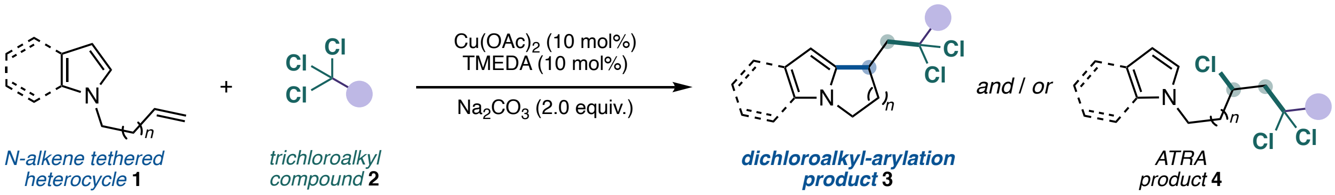

In a microwave reaction vial equipped with a stir bar was charged with *N*-alkene tethered heterocycle **1**, $Cu(OAc)_2$ (10 mol%), TMEDA (10 mol%), and Na_2CO_3 (2.0 equiv.). The trichloroalkyl compound 2 was charged as solvent (0.4 M) or using 3.0 equiv. and DMF as solvent (0.4 M), as specified. The vial was sealed with a PTFE lined butyl rubber septum and aluminum crimp cap, and the solution was degassed by three consecutive freeze−pump−thaw cycles using liquid nitrogen and backfilled with pure argon. The mixture was stirred in an oil bath at 70 ºC for 1.5 h or 110 ºC for 15 min, as specified or unless the reaction procedure states otherwise. After cooling to room temperature, the crude reaction mixture was extracted with a saturated solution of $NaHCO_3$ and EtOAc. The organic phase was dried over anhydrous Na_2SO_4 and evaporated under reduced pressure. The residue was purified by flash column chromatography on silica gel to afford the desired dichloroalkyl-arylation product **3** and/or ATRA product **4**.

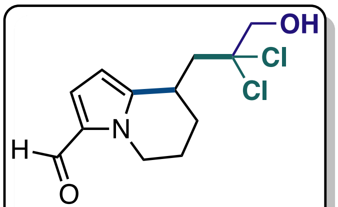
3a

8-(2,2-Dichloro-3-hydroxypropyl)-5,6,7,8-tetrahydroindolizine-3-carbaldehyde (3a) (1 mmol scale). In a microwave reaction vial equipped with a stir bar was charged with 1-(pent-4-en-1-yl)-1*H*-pyrrole-2-carbaldehyde (**1a**) (163 mg, 1.0 mmol), $Cu(OAc)_2$ (19 mg, 0.1 mmol), TMEDA (12 mg, 0.1 mmol), Na_2CO_3 (212 mg, 2.0 mmol), and 2,2,2-trichloroethanol (**2a**) (2.4 mL). The vial was sealed with a PTFE lined butyl rubber septum and aluminum crimp cap, and the solution was degassed by three consecutive freeze−pump−thaw cycles using liquid nitrogen and backfilled with pure argon. The mixture was stirred at 110 ºC in an oil bath for 15 min. After cooling to room temperature, the crude reaction mixture was extracted with a saturated solution of $NaHCO_3$ (40 mL) and EtOAc (50 mL). The organic phase was dried over anhydrous Na_2SO_4 and evaporated under reduced pressure. The residue was purified by flash column chromatography on silica gel with hexane/EtOAc (8:2) as eluent to afford the dichloroalkyl-arylation product **3a** (185 mg, 0.67 mmol, 67%) as a violet oil.

8-(2,2-Dichloro-3-hydroxypropyl)-5,6,7,8-tetrahydroindolizine-3-carbaldehyde (3a). Following the general procedure above, using 1-(pent-4-en-1-yl)-1*H*-pyrrole-2-carbaldehyde (**1a**) (67 mg, 0.41 mmol), $Cu(OAc)_2$ (8 mg, 0.041 mmol), TMEDA (5 mg, 0.041 mmol), Na_2CO_3 (87 mg, 0.82 mmol), and 2,2,2-trichloroethanol (**2a**) (1.0 mL). The mixture was stirred at 110 ºC in an oil bath for 15 min. The crude reaction mixture was purified by flash column chromatography on silica gel with hexane/EtOAc (8:2) as eluent to afford the dichloroalkyl-arylation product **3a** (70 mg, 0.253 mmol, 62%) as a violet oil. **^{1}H NMR** (400 MHz, $CDCl_3$): δ 9.44 (s, 1H), 6.89 (d, *J* = 4.2 Hz, 1H), 6.18 (dd, *J* = 4.1, 0.9 Hz, 1H), 4.53 (dt, *J* = 13.9, 5.1 Hz, 1H), 4.21 (ddd, *J* = 14.3, 9.5, 5.3 Hz, 1H), 4.00, 3.95 (AB system, *J* = 12.9 Hz, 2H), 3.38 – 3.30 (m, 1H), 2.80 (dd, *J* = 15.3, 2.9 Hz, 1H), 2.47 (dd, *J* = 15.3, 7.2 Hz, 1H), 2.37 – 2.28 (m, 1H), 2.09 (dddt, *J* = 16.1, 7.1, 4.8, 2.4 Hz, 1H), 2.00 – 1.88 (m, 1H), 1.74 (dddd, *J* = 13.4, 10.7, 8.9, 2.8 Hz, 1H). **^{13}C{^{1}H} NMR** (100 MHz, $CDCl_3$): δ 178.9, 143.6, 131.2, 124.6, 107.9, 93.4, 72.7, 49.1, 45.7, 32.4, 27.9, 21.8. **FT-IR** (ATR) ν_{max}: 3320, 2927, 2865, 2791, 2718, 1723, 1634, 1490, 1468, 1437, 1398, 1319, 1265, 1202, 1163, 1129, 1070, 1039, 966, 938, 785, 734, 712, 681, 646, 626, 597, 566, 441, 409 cm^{-1}. **HRMS**-(DART) (*m*/*z*) calcd for $C_{12}H_{16}Cl_2NO_2$ $[M+H]^+$: 276.0558; found: 276.0545.

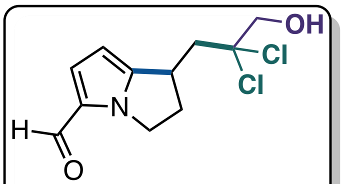
3b

1-(2,2-Dichloro-3-hydroxypropyl)-2,3-dihydro-1*H*-pyrrolizine-5-carbaldehyde (3b). Following the general procedure above, using 1-(but-3-en-1-yl)-1*H*-pyrrole-2-carbaldehyde (**1b**) (61 mg, 0.41 mmol), $Cu(OAc)_2$ (8 mg, 0.041 mmol), TMEDA (5 mg, 0.041 mmol), Na_2CO_3 (87 mg, 0.82 mmol), and 2,2,2-trichloroethanol (**2a**) (1.0 mL). The mixture was stirred at 110 ºC in an oil bath for 15 min. The crude reaction mixture was purified by flash column chromatography on silica gel with hexane/EtOAc (8:2) as eluent to afford the dichloroalkyl-arylation product **3b** (61 mg, 0.233 mmol, 57%) as a brown oil. **^{1}H NMR** (400 MHz, $CDCl_3$): δ 9.38 (s, 1H), 6.94 (d, *J* = 4.0 Hz, 1H), 6.03 (dd, *J* = 3.9, 0.9 Hz, 1H), 4.46 (ddd, *J* = 11.9, 8.9, 3.0 Hz, 1H), 4.19 – 4.09 (m, 1H), 4.00, 3.96 (AB system, *J* = 12.3 Hz, 2H), 3.61 (qd, *J* = 8.3, 3.1 Hz, 1H), 3.00 (bs, 1H), 2.95 (dtd, *J* = 13.1, 7.7, 3.1 Hz, 1H), 2.84 (dd, *J* = 15.1, 3.2 Hz, 1H), 2.49 – 2.36 (m, 2H). **^{13}C{^{1}H} NMR** (100 MHz, $CDCl_3$): δ 178.6, 149.8, 128.5, 126.5, 103.0, 92.9, 72.6,

47.7, 47.3, 36.2, 34.6. **FT-IR** (ATR) ν_{max}: 3301, 2931, 2857, 2792, 2723, 1635, 1529, 1457, 1444, 1413, 1359, 1256, 1166, 1130, 1074, 1031, 803, 765, 721, 649, 597, 560 cm^{-1}. **HRMS**-(EI) (*m/z*) calcd for $C_{11}H_{13}Cl_2NO_2$ [M]$^+$: 261.0323; found: 261.0318.

Following the general procedure above, using 1-(hex-5-en-1-yl)-1*H*-pyrrole-2-carbaldehyde (**1c**) (59 mg, 0.332 mmol), Cu(OAc)$_2$ (6 mg, 0.033 mmol), TMEDA (4 mg, 0.033 mmol), Na_2CO_3 (70 mg, 0.67 mmol), and 2,2,2-trichloroethanol (**2a**) (0.81 mL). The mixture was stirred at 70 ºC in an oil bath for 1.5 h. The crude reaction mixture was purified by flash column chromatography on silica gel with hexane/EtOAc (8:2) as eluent to afford the dichloroalkyl-arylation product **3c** (30 mg, 0.103 mmol, 31%) as a brown oil, and the ATRA product **4c** (10 mg, 0.031 mmol, 9%) as a brown oil.

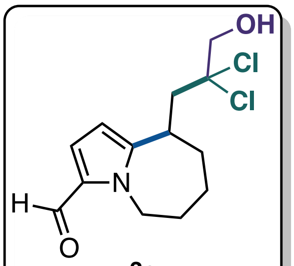

3c

9-(2,2-Dichloro-3-hydroxypropyl)-6,7,8,9-tetrahydro-5*H*-pyrrolo[1,2-*a*]azepine-3-carbaldehyde (3c). **^{1}H NMR** (400 MHz, $CDCl_3$): δ 9.38 (s, 1H), 6.79 (d, *J* = 4.0 Hz, 1H), 6.09 (d, *J* = 4.0 Hz, 1H), 4.72 (bs, 1H), 3.89, 3.83 (AB system, *J* = 12.5 Hz, 2H), 3.42 (bs, 1H), 2.90 (dd, *J* = 15.0, 6.3 Hz, 1H), 2.68 (bs, 1H), 2.58 (bs, 1H), 2.52 (dd, *J* = 15.0, 5.3 Hz, 1H), 2.07 – 1.63 (m, 6H). **^{13}C{^{1}H} NMR** (100 MHz, $CDCl_3$): δ 179.5, 148.9, 131.6, 125.3, 109.3, 93.8, 72.3, 45.9, 45.1, 35.3, 33.8, 28.3, 27.5. **FT-IR** (ATR) ν_{max}: 3370, 2926, 2851, 2799, 2736, 1635, 1487, 1468, 1443, 1404, 1349, 1331, 1262, 1220, 1184, 1155, 1080, 1039, 974, 938, 892, 808, 773, 724, 705, 648, 628, 608, 541, 482, 464 cm^{-1}. **HRMS**-(DART) (*m/z*) calcd for $C_{13}H_{18}Cl_2NO_2$ [M+H]$^+$: 290.0715; found: 290.0701.

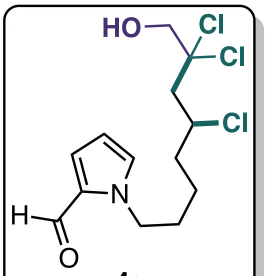

4c

1-(5,7,7-Trichloro-8-hydroxyoctyl)-1*H*-pyrrole-2-carbaldehyde (4c). **^{1}H NMR** (400 MHz, $CDCl_3$): δ 9.52 (d, *J* = 1.0 Hz, 1H), 6.96 – 6.92 (m, 2H), 6.23 (dd, *J* = 3.9, 2.5 Hz, 1H), 4.39 – 4.26 (m, 2H), 4.24 – 4.17 (m, 1H), 4.08, 3.96 (AB system, *J* = 12.6 Hz, 2H), 2.86 (dd, *J* = 15.8, 7.5 Hz, 1H), 2.66 (dd, *J* = 15.8, 3.2 Hz, 1H), 1.94 – 1.76 (m, 4H), 1.64 – 1.43 (m, 2H). **^{13}C{^{1}H} NMR** (100 MHz, $CDCl_3$): δ 179.5, 131.5, 131.4, 125.3, 109.9, 91.6, 71.6, 58.1, 51.7, 49.0, 38.8, 30.7, 23.2. **FT-IR** (ATR) ν_{max}: 3396, 2928, 2862, 2766, 2722, 1640, 1525, 1480, 1454, 1402, 1365, 1323, 1217, 1070, 1027, 952, 887, 764, 746, 606, 557, 503 cm^{-1}. **HRMS**-(DART) (*m/z*) calcd for $C_{13}H_{19}Cl_3NO_2$ [M+H]$^+$: 326.0481; found: 326.0475.

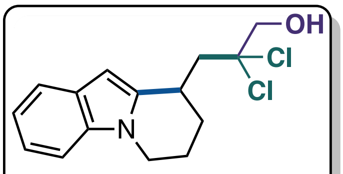

3d

2,2-Dichloro-3-(6,7,8,9-tetrahydropyrido[1,2-*a*]indol-9-yl)propan-1-ol (3d). Following the general procedure above, using 1-(pent-4-en-1-yl)-1*H*-indole (**1d**) (76 mg, 0.41 mmol), Cu(OAc)$_2$ (8 mg, 0.041 mmol), TMEDA (5 mg, 0.041 mmol), Na_2CO_3 (87 mg, 0.82 mmol), and 2,2,2-trichloroethanol (**2a**) (1.0 mL). The mixture was stirred at 110 ºC in an oil bath for 15 min. The crude reaction mixture was purified by flash column chromatography on silica gel with DCM/hexane (8:2) as eluent to afford the dichloroalkyl-arylation product **3d** (50 mg, 0.168 mmol, 41%) as a violet oil. **^{1}H NMR** (300 MHz, $CDCl_3$): δ 7.55 (d, *J* = 7.0 Hz, 1H), 7.27 (d, *J* = 7.1 Hz, 1H), 7.16 (dt, *J* = 6.9, 1.8, 1.2 Hz, 1H), 7.09 (ddd, *J* = 8.2, 7.0, 1.3 Hz, 1H), 6.38 (bs, 1H), 4.24 – 4.14 (m, 1H), 4.05 – 3.88 (m, 3H), 3.52 – 3.41 (m, 1H), 2.98 (dd, *J* = 15.3, 3.0 Hz, 1H), 2.53 (dd, *J* = 15.3, 7.0 Hz, 1H), 2.48 – 2.38 (m, 1H), 2.26 – 2.17 (m, 1H), 2.15 – 2.03 (m, 1H), 1.82 – 1.68 (m, 1H). **^{13}C{^{1}H} NMR** (100 MHz, $CDCl_3$): δ 140.7, 136.5, 128.1, 120.9, 120.1, 120.0, 108.9, 97.9, 93.9, 72.8, 49.2, 42.3, 32.8, 29.2, 22.3. **FT-IR** (ATR) ν_{max}: 3364, 3049, 2925, 2865, 1710, 1610, 1575, 1530, 1456, 1414, 1363, 1312, 1228, 1197, 1165, 1070, 1012, 956, 920, 808, 738, 714, 644, 595, 568, 423 cm^{-1}. **HRMS**-(DART) (*m/z*) calcd for $C_{15}H_{18}Cl_2NO$ [M+H]$^+$: 298.0765; found: 298.0754.

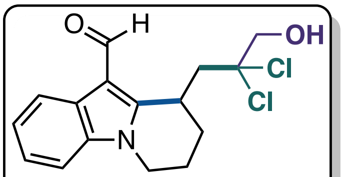

3e

9-(2,2-Dichloro-3-hydroxypropyl)-6,7,8,9-tetrahydropyrido[1,2-*a*]indole-10-carbaldehyde (3e). Following the general procedure above, using 1-(pent-4-en-1-yl)-1*H*-indole-3-carbaldehyde (**1e**) (59 mg, 0.277 mmol), Cu(OAc)$_2$ (5 mg, 0.028 mmol), TMEDA (3 mg, 0.028 mmol), Na_2CO_3 (59 mg, 0.533 mmol), and 2,2,2-trichloroethanol (**2a**) (0.68 mL). The mixture was stirred at 110 ºC in an oil bath for 15 min. The crude reaction mixture was purified by flash column chromatography on silica gel with hexane/EtOAc (7:3) as eluent to afford the dichloroalkyl-arylation product **3e** (59 mg, 0.181 mmol, 65%) as brown crystals. mp: 155-157 ºC. **^{1}H NMR** (700 MHz, $CDCl_3$): δ 10.25 (s, 1H), 8.07 (d, *J* = 7.4 Hz, 1H), 7.36 – 7.29 (m, 3H), 4.32 (ddd, *J* = 12.5, 6.2, 2.1 Hz, 1H), 4.24 (d, *J* = 12.5 Hz, 1H), 4.16 – 4.13 (m, 1H), 4.00 (d, *J* = 13.0 Hz, 1H), 3.98 – 3.93 (m, 1H), 2.84 – 2.73 (m, 3H), 2.38 – 2.30 (m, 1H), 2.18 – 2.13 (m, 1H), 1.93 (tdd, *J* = 13.7, 5.1, 3.0 Hz, 1H). **^{13}C{^{1}H} NMR** (175 MHz, $CDCl_3$): δ 184.6, 149.5, 136.3, 127.1, 123.6, 123.3, 119.3, 111.6, 109.9, 92.0, 71.8, 45.8, 42.7, 30.7, 22.8, 17.4. **FT-IR** (film) ν_{max}: 3307, 2950, 2931, 2866, 2852, 1625, 1610, 1579, 1509, 1476, 1455, 1435, 1400, 1380, 1339, 1314, 1250, 1203, 1167, 1142, 1113, 1083, 1062, 1023, 974, 912, 894, 873, 783, 759, 740, 713, 643, 593, 562, 546, 513, 436 cm^{-1}. **HRMS**-(DART) (*m/z*) calcd for $C_{16}H_{18}Cl_2NO_2$ [M+H]$^+$: 326.0714; found: 326.0705.

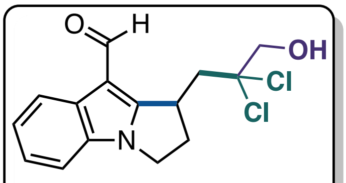

3f

1-(2,2-Dichloro-3-hydroxypropyl)-2,3-dihydro-1*H*-pyrrolo[1,2-*a*]indole-9-carbaldehyde (3f). Following the general procedure above, using 1-(but-3-en-1-yl)-1*H*-indole-3-carbaldehyde (**1f**) (90 mg, 0.452 mmol), Cu(OAc)$_2$ (8 mg, 0.045 mmol), TMEDA (5 mg, 0.045 mmol), Na_2CO_3 (96 mg, 0.903 mmol), and 2,2,2-trichloroethanol (**2a**) (1.1 mL). The mixture was stirred at 70 ºC in an oil bath for 1.5 h. The crude reaction mixture was purified by flash column chromatography on silica gel with hexane/EtOAc (7:3) as eluent to afford the dichloroalkyl-arylation product **3f** (28 mg, 0.09 mmol, 20%) as colorless crystals. mp: 155-158 ºC. **^{1}H NMR** (400 MHz, $CDCl_3$): δ 10.14 (s, 1H), 8.03 (d, *J* = 6.0 Hz, 1H), 7.34 – 7.28 (m, 3H), 4.30 – 4.22 (m, 2H), 4.18 (ddd, *J* = 10.8, 9.0, 4.6 Hz, 1H), 4.04 (d, *J* = 13.1 Hz, 1H), 3.95 – 3.88 (m, 1H), 3.18 (dd, *J* = 15.2, 1.3 Hz, 1H), 3.14 – 3.06 (m, 1H), 2.85 (ddd, *J* = 17.6, 8.2, 4.2 Hz, 1H), 2.54 (dd, *J* = 15.1, 9.5 Hz, 1H). **^{13}C{^{1}H} NMR** (175 MHz, $CDCl_3$): δ

184.2, 155.4, 132.7, 131.4, 123.3, 123.2, 119.7, 110.8, 109.1, 91.9, 71.4, 47.3, 44.1, 36.0, 35.4. **FT-IR** (ATR) ν_{max}: 3403, 3047, 2997, 2952, 2927, 2869, 2851, 2787, 2759, 1980, 1947, 1909, 1873, 1829, 1791, 1642, 1571, 1526, 1473, 1447, 1434, 1398, 1371, 1346, 1306, 1286, 1249, 1169, 1121, 1095, 1077, 1035, 940, 844, 753, 742, 710, 677, 578, 549, 483, 462, 415 cm^{-1}. **HRMS**-(DART) (*m*/*z*) calcd for $C_{15}H_{16}Cl_2NO_2$ [M+H]$^+$: 312.0558; found: 312.0552.

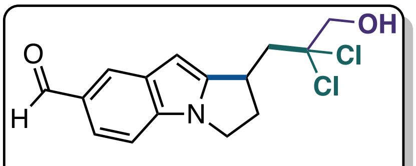

3g

1-(2,2-Dichloro-3-hydroxypropyl)-2,3-dihydro-1*H*-pyrrolo[1,2-*a*]indole-7-carbaldehyde (3g). Following the general procedure above, using 1-(but-3-en-1-yl)-1*H*-indole-5-carbaldehyde (**1g**) (82 mg, 0.412 mmol), $Cu(OAc)_2$ (8 mg, 0.041 mmol), TMEDA (5 mg, 0.041 mmol), Na_2CO_3 (87 mg, 0.823 mmol), and 2,2,2-trichloroethanol (**2a**) (1.0 mL). The mixture was stirred at 70 ºC in an oil bath for 1.5 h. The crude reaction mixture was purified by flash column chromatography on silica gel with DCM/hexane (6:4) as eluent to afford the dichloroalkyl-arylation product **3g** (38 mg, 0.122 mmol, 30%) as a violet oil. **^{1}H NMR** (400 MHz, $CDCl_3$): δ 9.98 (s, 1H), 8.06 (s, 1H), 7.70 (dd, *J* = 8.5, 1.6 Hz, 1H), 7.29 (d, *J* = 8.5 Hz, 1H), 6.37 (s, 1H), 4.23 (ddd, *J* = 10.3, 8.7, 2.7 Hz, 1H), 4.08 – 3.99 (m, 3H), 3.85 – 3.76 (m, 1H), 3.07 (dtd, *J* = 12.8, 7.4, 2.8 Hz, 1H), 2.99 (dd, *J* = 15.0, 3.2 Hz, 1H), 2.57 – 2.48 (m, 2H). **^{13}C{^{1}H} NMR** (100 MHz, $CDCl_3$): δ 192.9, 149.2, 136.2, 132.6, 129.3, 126.0, 121.6, 110.0, 94.9, 93.0, 72.7, 48.0, 43.8, 36.7, 34.8. **FT-IR** (ATR) ν_{max}: 3379, 2923, 2873, 2852, 1667, 1602, 1565, 1472, 1449, 1393, 1347, 1291, 1227, 1195, 1123, 1071, 1016, 942, 890, 801, 716, 662, 582, 567, 425 cm^{-1}. **HRMS**-(DART) (*m*/*z*) calcd for $C_{15}H_{16}Cl_2NO_2$ [M+H]$^+$: 312.0558; found: 312.0554.

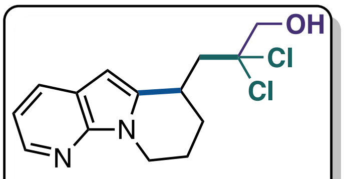

3h

2,2-Dichloro-3-(6,7,8,9-tetrahydropyrido[3,2-*b*]indolizin-6-yl)propan-1-ol (3h). Following the general procedure above, using 1-(pent-4-en-1-yl)-1*H*-pyrrolo[2,3-*b*]pyridine (**1h**) (76 mg, 0.41 mmol), $Cu(OAc)_2$ (8 mg, 0.041 mmol), TMEDA (5 mg, 0.041 mmol), Na_2CO_3 (87 mg, 0.82 mmol), and 2,2,2-trichloroethanol (**2a**) (1.0 mL). The mixture was stirred at 110 ºC in an oil bath for 15 min. The crude reaction mixture was purified by flash column chromatography on silica gel with hexane/EtOAc (6:4) as eluent to afford the dichloroalkyl-arylation product **3h** (80 mg, 0.267 mmol, 65%) as a yellow solid. mp: 40-44 ºC. **^{1}H NMR** (400 MHz, $CDCl_3$): δ 8.25 (dd, *J* = 4.8, 1.5 Hz, 1H), 7.82 (dd, *J* = 7.7, 1.5 Hz, 1H), 7.04 (dd, *J* = 7.7, 4.8 Hz, 1H), 6.35 (d, *J* = 1.4 Hz, 1H), 4.50 – 4.42 (m, 1H), 4.09 – 3.97 (m, 3H), 3.51 – 3.43 (m, 1H), 2.96 (dd, *J* = 15.3, 3.0 Hz, 1H), 2.55 (dd, *J* = 15.3, 6.9 Hz, 1H), 2.49 – 2.41 (m, 1H), 2.28 – 2.19 (m, 1H), 2.11 – 1.99 (m, 1H), 1.83 – 1.72 (m, 1H). **^{13}C{^{1}H} NMR** (100 MHz, $CDCl_3$): δ 147.7, 141.7, 141.7, 127.9, 120.9, 116.2, 96.1, 93.7, 72.7, 49.2, 41.4, 32.9, 29.2, 22.1. **FT-IR** (ATR) ν_{max}: 3200, 2920, 2862, 1941, 1903, 1872, 1714, 1634, 1595, 1574, 1532, 1480, 1433, 1400, 1370, 1304, 1287, 1257, 1217, 1166, 1146, 1114, 1070, 1030, 1004, 955, 934, 831, 804, 765, 740, 713, 650, 594, 539, 494, 449, 428 cm^{-1}. **HRMS**-(DART) (*m*/*z*) calcd for $C_{14}H_{17}Cl_2N_2O$ [M+H]$^+$: 299.0717; found: 299.0712.

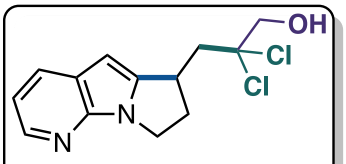

3i

2,2-Dichloro-3-(7,8-dihydro-6*H*-pyrido[3,2-*b*]pyrrolizin-6-yl)propan-1-ol (3i). Following the general procedure above, using 1-(but-3-en-1-yl)-1*H*-pyrrolo[2,3-*b*]pyridine (**1i**) (71 mg, 0.41 mmol), $Cu(OAc)_2$ (8 mg, 0.041 mmol), TMEDA (5 mg, 0.041 mmol), Na_2CO_3 (87 mg, 0.82 mmol), and 2,2,2-trichloroethanol (**2a**) (1.0 mL). The mixture was stirred at 110 ºC in an oil bath for 15 min. The crude reaction mixture was purified by flash column chromatography on silica gel with hexane/EtOAc (1:1) as eluent to afford the dichloroalkyl-arylation product **3i** (57 mg, 0.2 mmol, 49%) as a pale-yellow solid. mp: 150-152 ºC. **^{1}H NMR** (400 MHz, $CDCl_3$): δ 8.21 (dd, *J* = 4.9, 1.5 Hz, 1H), 7.83 (dd, *J* = 7.8, 1.5 Hz, 1H), 7.02 (dd, *J* = 7.8, 4.8 Hz, 1H), 6.19 (d, *J* = 1.4 Hz, 1H), 4.42 (ddd, *J* = 10.7, 8.7, 2.8 Hz, 1H), 4.13 (ddd, *J* = 10.7, 8.9, 7.1 Hz, 1H), 4.06, 4.02 (AB system, *J* = 12.3 Hz, 2H), 3.88 – 3.74 (m, 1H), 3.09 – 3.00 (m, 1H), 2.98 (dd, *J* = 15.0, 3.2 Hz, 1H), 2.57 – 2.43 (m, 2H). **^{13}C{^{1}H} NMR** (100 MHz, $CDCl_3$): δ 148.1, 144.1, 141.4, 129.0, 125.8, 115.6, 93.2, 91.4, 72.7, 47.9, 43.1, 36.8, 35.1. **FT-IR** (ATR) ν_{max}: 3129, 2957, 2918, 2897, 1937, 1899, 1861, 1732, 1594, 1571, 1539, 1490, 1442, 1407, 1362, 1297, 1275, 1208, 1151, 1127, 1096, 1078, 1038, 1014, 970, 949, 929, 896, 841, 803, 774, 755, 716, 693, 642, 595, 583, 564, 480, 464, 437 cm^{-1}. **HRMS**-(DART) (*m*/*z*) calcd for $C_{13}H_{15}Cl_2N_2O$ [M+H]$^+$: 285.0561; found: 285.0555.

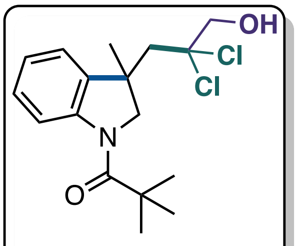

3j

1-(3-(2,2-Dichloro-3-hydroxypropyl)-3-methylindolin-1-yl)-2,2-dimethylpropan-1-one (3j). Following the general procedure above, using *N*-(2-methylallyl)-*N*-phenylpivalamide (**1j**) (95 mg, 0.41 mmol), $Cu(OAc)_2$ (8 mg, 0.041 mmol), TMEDA (5 mg, 0.041 mmol), Na_2CO_3 (87 mg, 0.82 mmol), and 2,2,2-trichloroethanol (**2a**) (1.0 mL). The mixture was stirred at 110 ºC in an oil bath for 15 min. The crude reaction mixture was purified by flash column chromatography on silica gel with hexane/EtOAc (8:2) as eluent to afford the dichloroalkyl-arylation product **3j** (91 mg, 0.264 mmol, 65%) as a brown solid. mp: 111-113 ºC. **^{1}H NMR** (400 MHz, $CDCl_3$): δ 8.21 (ddd, *J* = 8.2, 1.1, 0.6 Hz, 1H), 7.22 (ddd, *J* = 8.2, 7.3, 1.5 Hz, 1H), 7.15 (ddd, *J* = 7.5, 1.5, 0.6 Hz, 1H), 7.06 (td, *J* = 7.5, 1.1 Hz, 1H), 4.49 (d, *J* = 10.7 Hz, 1H), 4.19 (d, *J* = 10.7 Hz, 1H), 3.86 (s, 2H), 2.72 (s, 2H), 1.58 (s, 3H), 1.38 (s, 9H). **^{13}C{^{1}H} NMR** (100 MHz, $CDCl_3$): δ 176.8, 143.1, 139.0, 128.4, 124.2, 122.1, 118.8, 91.9, 73.8, 62.5, 50.4, 45.0, 40.4, 27.9, 25.9. **FT-IR** (ATR) ν_{max}: 3399, 3074, 2969, 2936, 2927, 2873, 1620, 1591, 1478, 1407, 1380, 1361, 1276, 1242, 1220, 1203, 1174, 1149, 1095, 1082, 1026, 1005, 994, 950, 922, 873, 851, 808, 757, 733, 702, 664, 628, 602, 554, 486, 449, 430 cm^{-1}. **HRMS**-(DART) (*m*/*z*) calcd for $C_{17}H_{24}Cl_2NO_2$ [M+H]$^+$: 344.1184; found: 344.1169.

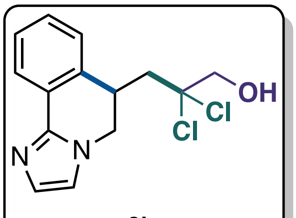

3k

2,2-Dichloro-3-(5,6-dihydroimidazo[2,1-*a*]isoquinolin-6-yl)propan-1-ol (3k). Following the general procedure above, using 1-allyl-2-phenyl-1*H*-imidazole (**1k**) (76 mg, 0.41 mmol), $Cu(OAc)_2$ (8 mg, 0.041 mmol), TMEDA (5 mg, 0.041 mmol), Na_2CO_3 (87 mg, 0.82 mmol), and 2,2,2-trichloroethanol (**2a**) (1.0 mL). The mixture was stirred at 110 ºC in an oil bath for 2 h. The crude reaction mixture was purified by flash column chromatography on silica gel with EtOAc/DCM (6:4) as eluent to afford the dichloroalkyl-arylation product **3k** (57 mg, 0.192 mmol, 47%) as an orange oil. **^{1}H NMR** (400 MHz, $CDCl_3$): δ 7.93 (bs, 1H), 7.37 – 7.27 (m, 3H), 7.00 (bs, 1H), 6.88 (bs, 1H), 4.58 (dd, *J* = 13.1, 2.2 Hz, 1H), 4.17 (dt, *J* = 13.0, 3.6 Hz, 1H), 3.97, 3.92 (AB system, *J* = 12.3Hz, 2H), 3.66 (dd, *J* = 8.1, 3.8 Hz, 1H), 2.38, 2.25 (ABX system, *J* = 15.4, 8.2, 2.9 Hz, 2H). **^{13}C{^{1}H} NMR** (100 MHz, $CDCl_3$): δ 143.7, 136.9, 129.4, 128.7, 128.3, 127.7, 125.7, 124.0, 120.0, 93.7, 72.8, 47.5, 46.1, 36.2. **FT-IR** (ATR) ν_{max}: 3138, 3111, 3055, 2920, 2849, 1747, 1675, 1608, 1581, 1539, 1502, 1474, 1453, 1424, 1364, 1323, 1279, 1264, 1218, 1178, 1138, 1089, 1074, 1049, 1023, 933, 838, 819, 734, 703, 595, 534, 475, 435 cm^{-1}. **HRMS**-(DART) (*m*/*z*) calcd for $C_{14}H_{15}Cl_2N_2O$ $[M+H]^+$: 297.0561; found: 297.0548.

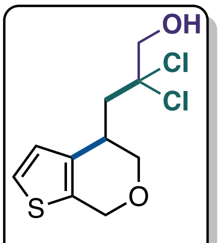

3l

2,2-Dichloro-3-(4,7-dihydro-5*H*-thieno[2,3-*c*]pyran-4-yl)propan-1-ol (3l). Following the general procedure above, using 2-((allyloxy)methyl)thiophene (**1l**) (63 mg, 0.41 mmol), $Cu(OAc)_2$ (8 mg, 0.041 mmol), TMEDA (5 mg, 0.041 mmol), Na_2CO_3 (87 mg, 0.82 mmol), and 2,2,2-trichloroethanol (**2a**) (1.0 mL). The mixture was stirred at 110 ºC in an oil bath for 2 h. The crude reaction mixture was purified by flash column chromatography on silica gel with hexane/EtOAc (8:2) as eluent to afford the dichloroalkyl-arylation product **3l** (15 mg, 0.056 mmol, 14%) as a brown oil. **^{1}H NMR** (400 MHz, $CDCl_3$): δ 7.16 (d, *J* = 5.2 Hz, 1H), 6.71 (d, *J* = 5.2 Hz, 1H), 4.75, 4.70 (ABX system, *J* = 14.5, 1.3, 1.7 Hz, 2H), 4.06 (dd, *J* = 7.1, 4.3 Hz, 2H), 3.96 (d, *J* = 4.6 Hz, 2H), 3.48 – 3.41 (m, 1H), 2.71, 2.60 (ABX system, *J* = 15.4, 7.2, 2.7 Hz, 2H). **^{13}C{^{1}H} NMR** (100 MHz, $CDCl_3$): δ 137.2, 134.3, 123.9, 123.6, 93.4, 72.8, 70.0, 66.8, 48.2, 33.5. **FT-IR** (ATR) ν_{max}: 3400, 2922, 2851, 1760, 1713, 1449, 1429, 1399, 1313, 1265, 1224, 1168, 1069, 917, 837, 812, 709, 639, 606, 566, 508, 452 cm^{-1}. **HRMS**-(EI) (*m*/*z*) calcd for $C_{10}H_{12}Cl_2O_2S$ $[M]^+$: 265.9935; found: 265.9946.

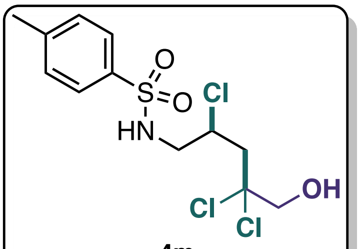

4m

4-Methyl-*N*-(2,4,4-trichloro-5-hydroxypentyl)benzenesulfonamide (4m). Following the general procedure above, using *N*-allyl-4-methylbenzenesulfonamide (**1m**) (108 mg, 0.51 mmol), $Cu(OAc)_2$ (9 mg, 0.051 mmol), TMEDA (6 mg, 0.051 mmol), Na_2CO_3 (108 mg, 1.02 mmol), and 2,2,2-trichloroethanol (**2a**) (1.3 mL). The mixture was stirred at 110 ºC in an oil bath for 15 min. The crude reaction mixture was purified by flash column chromatography on silica gel with hexane/EtOAc (7:3) as eluent to afford the ATRA product **4m** (107 mg, 0.297 mmol, 58%) as a colorless oil. **^{1}H NMR** (300 MHz, $CDCl_3$): δ 7.76, 7.33 (AA'BB' system, *J* = 8.1 Hz, 4H), 5.25 – 5.07 (m, 1H), 4.33 – 4.22 (m, 1H), 3.98 – 3.90 (AB system, *J* = 12.6 Hz, 2H), 3.49 – 3.37 (m, 1H), 3.30 – 3.18 (m, 1H), 2.74 (d, *J* = 5.3 Hz, 2H), 2.43 (s, 3H). **^{13}C{^{1}H} NMR** (75 MHz, $CDCl_3$): δ 144.2, 136.7, 130.1, 127.2, 90.8, 71.9, 56.6, 49.7, 48.5, 21.7. **FT-IR** (ATR) ν_{max}: 3479, 3278, 2927, 2874, 1597, 1448, 1417, 1323, 1230, 1154, 1089, 1068, 1018, 942, 899, 812, 707, 660, 549 cm^{-1}. **HRMS**-(DART) (*m*/*z*) calcd for $C_{12}H_{17}Cl_3NO_3S$ $[M+H]^+$: 359.9994; found: 359.9983.

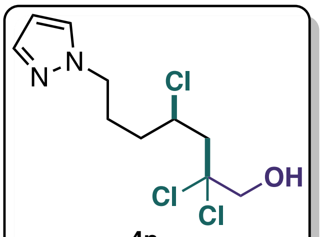

4n

2,2,4-Trichloro-7-(1*H*-pyrazol-1-yl)heptan-1-ol (4n). Following the general procedure above, using 1-(pent-4-en-1-yl)-1*H*-pyrazole (**1n**) (56 mg, 0.41 mmol), $Cu(OAc)_2$ (8 mg, 0.041 mmol), TMEDA (5 mg, 0.041 mmol), Na_2CO_3 (87 mg, 0.82 mmol), and 2,2,2-trichloroethanol (**2a**) (1.0 mL). The mixture was stirred at 110 ºC in an oil bath for 15 min. The crude reaction mixture was purified by flash column chromatography on silica gel with hexane/EtOAc (7:3) as eluent to afford the ATRA product **4n** (37 mg, 0.130 mmol, 32%) as a yellow oil. **^{1}H NMR** (400 MHz, $CDCl_3$): δ 7.50 (d, *J* = 1.3 Hz, 1H), 7.39 (d, *J* = 2.3 Hz, 1H), 6.26 – 6.24 (m, 1H), 4.25 (ddt, *J* = 8.3, 7.1, 4.0 Hz, 1H), 4.19 (t, *J* = 6.8 Hz, 2H), 4.04, 3.94 (AB system, *J* = 12.5 Hz, 2H), 3.22 (bs, 1H), 2.85, 2.64 (ABX system, *J* = 15.7, 7.1, 3.8 Hz, 2H), 2.22 – 1.99 (m, 2H), 1.91 – 1.72 (m, 2H). **^{13}C{^{1}H} NMR** (100 MHz, $CDCl_3$): δ 139.5, 129.3, 105.8, 91.5, 71.7, 57.6, 51.5, 51.2, 36.2, 27.1. **FT-IR** (ATR) ν_{max}: 3218, 2929, 2865, 1718, 1514, 1442, 1398, 1368, 1278, 1207, 1090, 1075, 1055, 949, 918, 881, 752, 718, 652, 617, 600, 558, 479 cm^{-1}. **HRMS**-(DART) (*m*/*z*) calcd for $C_{10}H_{16}Cl_3N_2O$ $[M+H]^+$: 285.0328; found: 285.0323.

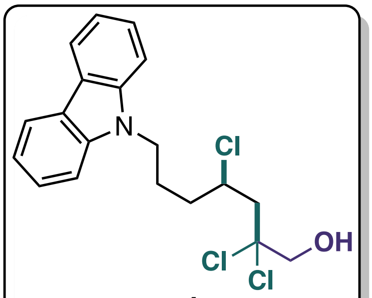

4o

7-(9*H*-Carbazol-9-yl)-2,2,4-trichloroheptan-1-ol (4o). Following the general procedure above, using 9-(pent-4-en-1-yl)-9*H*-carbazole (**1o**) (96 mg, 0.41 mmol), $Cu(OAc)_2$ (8 mg, 0.041 mmol), TMEDA (5 mg, 0.041 mmol), Na_2CO_3 (87 mg, 0.82 mmol), and 2,2,2-trichloroethanol (**2a**) (1.0 mL). The mixture was stirred at 110 ºC in an oil bath for 15 min. The crude reaction mixture was purified by flash column chromatography on silica gel with hexane/DCM (6:4) as eluent to afford the ATRA product **4o** (133 mg, 0.346 mmol, 84%) as a dark green oil. **^{1}H NMR** (400 MHz, $CDCl_3$): δ 8.11 (dd, *J* = 7.8, 0.7 Hz, 2H), 7.48 (ddt, *J* = 8.1, 7.0, 1.1 Hz, 2H), 7.41 (dd, *J* = 8.2, 1.0 Hz, 2H), 7.27 – 7.22 (m, 2H), 4.38 (t, *J* = 6.9 Hz, 2H), 4.23 (dp, *J* = 11.3, 3.9 Hz, 1H), 4.03, 3.89 (AB system, *J* = 12.6 Hz, 2H), 2.81 (ddd, *J* = 15.8, 7.6, 1.0 Hz, 1H), 2.56 (dd, *J* = 15.6, 3.2 Hz, 1H), 2.25 – 2.06 (m, 2H), 1.99 – 1.84 (m, 2H). **^{13}C{^{1}H} NMR** (100 MHz, $CDCl_3$): δ 140.4, 125.9, 123.1, 120.6, 119.2, 108.7, 91.4, 71.7, 57.9, 51.5, 42.4, 36.8, 25.7. **FT-IR** (ATR) ν_{max}: 3410, 3050, 2923, 2850, 1889, 1720, 1626, 1593, 1483, 1451, 1422, 1381, 1325, 1230, 1152, 1120, 1066, 1020, 946, 907, 830, 798, 748, 722, 599, 558, 528, 423 cm^{-1}. **HRMS**-(DART) (*m*/*z*) calcd for $C_{19}H_{21}Cl_3NO$ $[M+H]^+$: 384.0688; found: 384.0676.

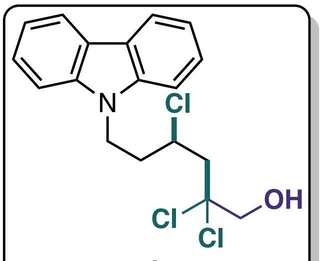

4p

6-(9*H*-Carbazol-9-yl)-2,2,4-trichlorohexan-1-ol (4p). Following the general procedure above, using 9-(but-3-en-1-yl)-9*H*-carbazole (**1p**) (75 mg, 0.339 mmol), $Cu(OAc)_2$ (6 mg, 0.034 mmol), TMEDA (4 mg, 0.034 mmol), Na_2CO_3 (72 mg, 0.678 mmol), and 2,2,2-trichloroethanol (**2a**) (0.83 mL). The mixture was stirred at 110 ºC in an oil bath for 15 min. The crude reaction mixture was purified by flash column chromatography on silica gel with hexane/DCM (6:4) as eluent to afford the ATRA product **4p** (57 mg, 0.154 mmol, 45%) as a dark green oil. **^{1}H NMR** (400 MHz, $CDCl_3$): δ 8.11 (dt, *J* = 7.8, 1.1 Hz, 2H), 7.52 – 7.45 (m, 4H), 7.28 – 7.23 (m, 2H), 4.64 – 4.50 (m, 2H), 4.37 – 4.29 (m, 1H), 3.99, 3.87 (AB system, *J* = 12.5, 7.0 Hz, 2H), 2.93, 2.65 (ABX system, *J* = 15.7, 6.8, 4.4 Hz, 2H), 2.60 – 2.50 (m, 1H), 2.38 (t, *J* = 7.7 Hz, 1H), 2.33 – 2.22 (m, 1H). **^{13}C{^{1}H} NMR** (100 MHz, $CDCl_3$): δ 140.3, 126.0, 123.2, 120.6, 119.3, 108.6, 91.0, 71.9, 56.1, 51.4, 40.1, 38.1. **FT-IR** (ATR) ν_{max}: 3548, 3431, 3051, 3022, 2924, 2852, 1927, 1888, 1853, 1805, 1769, 1705, 1659, 1626, 1595, 1483, 1452, 1380, 1324, 1232, 1184, 1152, 1120, 1064, 1001, 942, 908, 852, 748, 722, 598, 562, 528, 422 cm^{-1}. **HRMS**-(DART) (*m*/*z*) calcd for $C_{18}H_{19}Cl_3NO$ $[M+H]^+$: 370.0532; found: 370.0519.

Following the general procedure above, using 1-(pent-4-en-1-yl)-1*H*-pyrrole-2-carbaldehyde (**1a**) (67 mg, 0.41 mmol), $Cu(OAc)_2$ (8 mg, 0.041 mmol), TMEDA (5 mg, 0.041 mmol), Na_2CO_3 (87 mg, 0.82 mmol), ethyl 2,2,2-trichloroacetate (**2b**) (235 mg, 1.23 mmol, 3.0 equiv.), and DMF (1.0 mL). The mixture was stirred at 110 ºC in an oil bath for 3 h. The crude reaction mixture was purified by flash column chromatography on silica gel with hexane/EtOAc (8:2) as eluent to afford the polychloroalkyl-arylation products **3q** (42 mg, 0.132 mmol, 32%) and **3r** (17 mg, 0.061 mmol, 15%) as purple oils.

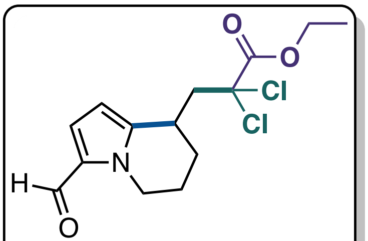

3q

Ethyl 2,2-dichloro-3-(3-formyl-5,6,7,8-tetrahydroindolizin-8-yl)propanoate (3q). ^{1}H NMR (400 MHz, $CDCl_3$): δ 9.44 (s, 1H), 6.89 (d, *J* = 4.2 Hz, 1H), 6.18 (dd, *J* = 4.2, 0.9 Hz, 1H), 4.51 (dt, *J* = 14.0, 5.3 Hz, 1H), 4.32 (q, *J* = 7.1 Hz, 2H), 4.22 (ddd, *J* = 14.1, 9.1, 5.1 Hz, 1H), 3.34 – 3.25 (m, 1H), 3.01, 2.74 (ABX system, *J* = 15.1, 3.7, 7.6 Hz, 2H), 2.22 – 2.14 (m, 1H), 2.13 – 2.03 (m, 1H), 1.97 – 1.85 (m, 1H), 1.66 (dddd, *J* = 12.9, 10.8, 8.5, 2.6 Hz, 1H), 1.36 (t, *J* = 7.2 Hz, 3H). **^{13}C{^{1}H} NMR** (100 MHz, $CDCl_3$): δ 178.9, 165.9, 142.6, 131.4, 124.5, 108.1, 83.9, 64.3, 50.2, 45.6, 32.6, 27.0, 21.6, 13.9. **FT-IR** (ATR) ν_{max}: 3101, 2921, 2850, 2540, 2391, 1715, 1582, 1465, 1397, 1366, 1327, 1268, 1193, 1172, 1142, 1087, 1036, 895, 867, 773, 661, 627, 579, 440 cm^{-1}. **HRMS**-(DART) (*m*/*z*) calcd for $C_{14}H_{18}Cl_2NO_3$ $[M+H]^+$: 318.0663; found: 318.0674.

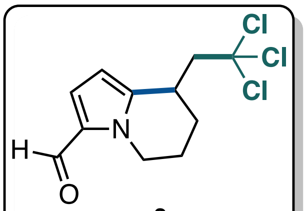

3r

8-(2,2,2-Trichloroethyl)-5,6,7,8-tetrahydroindolizine-3-carbaldehyde (3r). ^{1}H NMR (400 MHz, $CDCl_3$): δ 9.46 (s, 1H), 6.91 (d, *J* = 4.1 Hz, 1H), 6.21 (dd, *J* = 4.2, 0.8 Hz, 1H), 4.54 (dt, *J* = 14.0, 5.1 Hz, 1H), 4.23 (ddd, *J* = 14.2, 9.3, 5.1 Hz, 1H), 3.42 – 3.35 (m, 1H), 3.26, 2.99 (ABX system, *J* = 15.3, 2.8, 6.9 Hz, 2H), 2.42 – 2.33 (m, 1H), 2.10 (ddtd, *J* = 14.7, 7.5, 5.0, 2.8 Hz, 1H), 2.02 – 1.90 (m, 1H), 1.85 – 1.75 (m, 1H). **^{13}C{^{1}H} NMR** (100 MHz, $CDCl_3$): δ 179.0, 142.3, 131.4, 124.5, 108.0, 98.8, 60.8, 45.7, 33.8, 27.6, 21.8. **FT-IR** (ATR) ν_{max}: 3043, 2917, 2848, 2439, 1730, 1655, 1580, 1463, 1397, 1322, 1264, 1193, 1140, 1068, 1032, 964, 936, 896, 868, 780, 691, 622, 577 cm^{-1}. **HRMS**-(DART) (*m*/*z*) calcd for $C_{11}H_{13}Cl_3NO$ $[M+H]^+$: 280.0062; found: 280.0064.

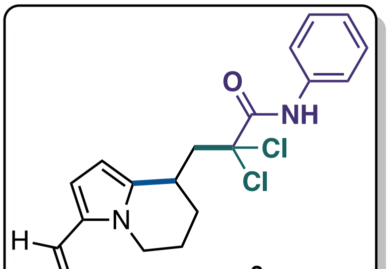

3s

2,2-Dichloro-3-(3-formyl-5,6,7,8-tetrahydroindolizin-8-yl)-*N*-phenylpropanamide (3s). Following the general procedure above, using 1-(pent-4-en-1-yl)-1*H*-pyrrole-2-carbaldehyde (**1a**) (67 mg, 0.41 mmol), $Cu(OAc)_2$ (8 mg, 0.041 mmol), TMEDA (5 mg, 0.041 mmol), Na_2CO_3 (87 mg, 0.82 mmol), 2,2,2-trichloro-*N*-phenylacetamide (**2c**) (293 mg, 1.23 mmol, 3.0 equiv.), and DMF (1.0 mL). The mixture was stirred at 110 ºC in an oil bath for 1 h. The crude reaction mixture was purified by flash column chromatography on silica gel with hexane/EtOAc (8:2) as eluent to afford the dichloroalkyl-arylation product **3s** (87 mg, 0.238 mmol, 58%) as a brown oil. **^{1}H NMR** (400 MHz, $CDCl_3$): δ 9.43 (s, 1H), 8.62 (bs, 1H), 7.57 (dd, *J* = 8.6, 1.2 Hz, 2H), 7.38 (t, *J* = 8.1 Hz, 2H), 7.20 (t, *J* = 7.4 Hz, 1H), 6.89 (d, *J* = 4.2 Hz, 1H), 6.24 (d, *J* = 4.2 Hz, 1H), 4.51 (dt, *J* = 14.1, 5.2 Hz, 1H), 4.18 (ddd, *J* = 14.7, 9.6, 5.4 Hz, 1H), 3.32 – 3.24 (m, 1H), 3.11, 2.91 (ABX system, *J* = 15.2, 3.3, 7.6 Hz, 2H), 2.24 – 2.15 (m, 1H), 2.12 – 2.02 (m, 1H), 1.95 – 1.82 (m, 1H), 1.71 (qd, *J* = 11.7, 2.5 Hz, 1H). **^{13}C{^{1}H} NMR** (100 MHz, $CDCl_3$): δ 178.9, 163.3, 142.9, 136.6, 131.2, 129.3, 125.8, 124.6, 120.4, 108.2, 86.3, 49.6, 45.6, 32.8, 27.0, 21.7. **FT-IR** (ATR) ν_{max}: 3396, 3305, 3130, 3058, 2926, 2861, 2788, 2723, 1693, 1642, 1597, 1528, 1489, 1441, 1400, 1317, 1298, 1238, 1203, 1178, 1164, 1100, 1038, 983, 904, 859, 821, 785, 753, 689, 636, 567, 504, 455 cm^{-1}. **HRMS**-(ESI) (*m*/*z*) calcd for $C_{18}H_{19}Cl_2N_2O_2$ $[M+H]^+$: 365.0824; found: 365.0830.

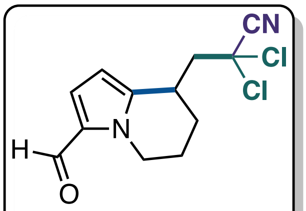

3t

2,2-Dichloro-3-(3-formyl-5,6,7,8-tetrahydroindolizin-8-yl)propanenitrile (3t). Following the general procedure above, using 1-(pent-4-en-1-yl)-1*H*-pyrrole-2-carbaldehyde (**1a**) (121 mg, 0.74 mmol), $Cu(OAc)_2$ (14 mg, 0.074 mmol), TMEDA (9 mg, 0.074 mmol), Na_2CO_3 (157 mg, 1.48 mmol), and trichloroacetonitrile (**2d**) (1.0 mL). The mixture was stirred at 110 ºC in an oil bath for 15 min. The crude reaction mixture was purified by flash column chromatography on silica gel with DCM as eluent to afford the dichloroalkyl-arylation product **3t** (33 mg, 0.122 mmol, 16%) as a dark solid. mp: 68-71 ºC. **^{1}H NMR** (400 MHz, $CDCl_3$): δ 9.48 (s, 1H), 6.92 (d, *J* = 4.2 Hz, 1H), 6.16 (d, *J* = 4.2 Hz, 1H), 4.56 (dt, *J* = 14.0, 5.1 Hz, 1H), 4.22 (ddd, *J* = 14.2, 9.4, 5.1 Hz, 1H), 3.42 – 3.33 (m, 1H), 3.05, 2.79 (ABX system, *J* = 15.2, 3.1, 8.1 Hz, 2H), 2.42 – 2.33 (m, 1H), 2.12 (ddtd, *J* = 14.6, 7.4, 5.0, 2.7

Hz, 1H), 2.02 – 1.90 (m, 1H), 1.79 (dddd, J = 13.4, 11.2, 8.8, 2.8 Hz, 1H). **^{13}C{^{1}H} NMR** (100 MHz, $CDCl_3$): δ 179.2, 140.7, 131.6, 124.4, 115.8, 107.8, 67.6, 53.5, 45.6, 32.9, 26.7, 21.6. **FT-IR** (ATR) ν_{max}: 3116, 3045, 2968, 2918, 2849, 2218, 1715, 1641, 1488, 1465, 1438, 1402, 1350, 1321, 1265, 1246, 1207, 1169, 1123, 1089, 1035, 976, 939, 821, 777, 724, 684, 644, 548, 498, 454, 406 cm^{-1}. **HRMS**-(DART) (m/z) calcd for $C_{12}H_{13}Cl_2N_2O$ [M+H]$^+$: 271.0405; found: 271.0393.

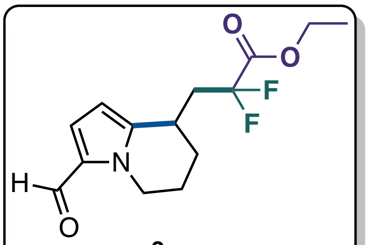

3u

Ethyl 2,2-difluoro-3-(3-formyl-5,6,7,8-tetrahydroindolizin-8-yl)propanoate (3u). Following the general procedure above, using 1-(pent-4-en-1-yl)-1H-pyrrole-2-carbaldehyde (**1a**) (67 mg, 0.41 mmol), $Cu(OAc)_2$ (8 mg, 0.041 mmol), TMEDA (5 mg, 0.041 mmol), Na_2CO_3 (87 mg, 0.82 mmol), and ethyl chlorodifluoroacetate (**2e**) (1.0 mL). The mixture was stirred at 110 ºC in an oil bath for 12 h. The crude reaction mixture was purified by flash column chromatography on silica gel with hexane/EtOAc (9:1) as eluent to afford the dichloroalkyl-arylation product **3u** (30 mg, 0.105 mmol, 26%) as a purple solid. mp: 167-170 ºC. **^{1}H NMR** (400 MHz, $CDCl_3$): δ 9.44 (s, 1H), 6.89 (d, J = 4.1 Hz, 1H), 6.08 (d, J = 4.2 Hz, 1H), 4.55 (dt, J = 13.9, 4.9 Hz, 1H), 4.33 (q, J = 7.1 Hz, 2H), 4.16 (ddd, J = 14.3, 10.0, 5.0 Hz, 1H), 3.21 (dp, J = 9.4, 4.6 Hz, 1H), 2.64 (dtd, J = 22.1, 15.4, 3.7 Hz, 1H), 2.35 – 2.24 (m, 1H), 2.22 – 2.14 (m, 1H), 2.08 (ddtd, J = 14.1, 7.1, 4.7, 2.7 Hz, 1H), 1.95 – 1.83 (m, 1H), 1.61 (dddd, J = 13.3, 11.4, 9.3, 2.6 Hz, 1H), 1.36 (t, J = 7.1 Hz, 3H). **^{13}C{^{1}H} NMR** (100 MHz, $CDCl_3$): δ 179.0, 164.1 (t, J_{C-F} = 32.5 Hz), 142.0, 131.4, 124.4, 115.9 (t, J_{C-F} = 251.8 Hz), 107.6, 63.3, 45.6, 39.7 (t, J_{C-F} = 22.6 Hz), 29.3, 26.6, 21.7, 14.1. **FT-IR** (ATR) ν_{max}: 3124, 2919, 2850, 2463, 1916, 1745, 1609, 1500, 1468, 1451, 1434, 1401, 1370, 1355, 1317, 1304, 1285, 1252, 1188, 1167, 1129, 1102, 1066, 1048, 1034, 945, 814, 794, 780, 766, 745, 698, 676, 638, 566, 496, 442, 414 cm^{-1}. **HRMS**-(ESI) (m/z) calcd for $C_{14}H_{18}F_2NO_3$ [M+H]$^+$: 286.1255; found: 286.1250.

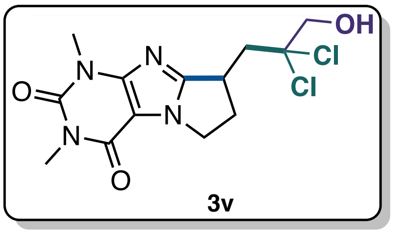

3v

8-(2,2-Dichloro-3-hydroxypropyl)-1,3-dimethyl-7,8-dihydro-1*H*-pyrrolo[2,1-*f*]purine-2,4(3*H*,6*H*)-dione (3v). Following the general procedure above, using 7-(but-3-en-1-yl)-1,3-dimethyl-3,7-dihydro-1H-purine-2,6-dione (**1v**) (80 mg, 0.342 mmol), $Cu(OAc)_2$ (6 mg, 0.034 mmol), TMEDA (4 mg, 0.034 mmol), Na_2CO_3 (72 mg, 0.683 mmol), and 2,2,2-trichloroethanol (**2a**) (1.0 mL). The mixture was stirred at 70 ºC in an oil bath for 1.5 h. The crude reaction mixture was purified by flash column chromatography on silica gel with EtOAc/DCM (6:4) as eluent to afford the dichloroalkyl-arylation product **3v** (29 mg, 0.084 mmol, 25%) as a pale-yellow solid. mp: 185-189 ºC. **^{1}H NMR** (400 MHz, $CDCl_3$): δ 4.40 (ddd, J = 11.2, 9.1, 1.4 Hz, 1H), 4.20 – 4.10 (m, 2H), 3.99 (dd, J = 13.0, 1.2 Hz, 1H), 3.51 (s, 3H), 3.46 (qd, J = 8.0, 2.1 Hz, 1H), 3.38 (s, 3H), 2.99 (dd, J = 15.2, 7.9 Hz, 2H), 2.54 (ddd, J = 15.2, 2.9, 1.2 Hz, 1H), 2.42 (dq, J = 13.0, 9.5 Hz, 1H). **^{13}C{^{1}H} NMR** (100 MHz, $CDCl_3$): δ 159.5, 154.7, 151.6, 151.3, 106.2, 92.0, 70.3, 46.6, 45.1, 35.1, 33.7, 30.3, 28.2. **FT-IR** (ATR) ν_{max}: 3391, 2920, 2850, 1697, 1652, 1544, 1472, 1425, 1407, 1336, 1297, 1200, 1087, 1050, 973, 948, 831, 746, 724, 700, 652, 603, 559, 527, 510, 492, 441, 418 cm^{-1}. **HRMS**-(DART) (m/z) calcd for $C_{13}H_{17}Cl_2N_4O_3$ [M+H]$^+$: 347.0677; found: 347.0675.

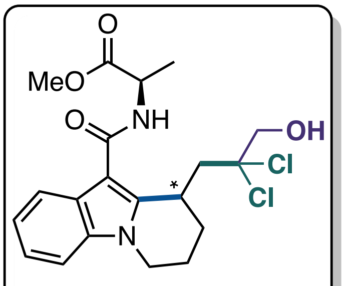

3w

Methyl (9-(2,2-dichloro-3-hydroxypropyl)-6,7,8,9-tetrahydropyrido[1,2-*a*]indole-10-carbonyl)-*D*-alaninate (3w). Following the general procedure above, using methyl (1-(pent-4-en-1-yl)-1H-indole-3-carbonyl)-D-alaninate (**1w**) (129 mg, 0.41 mmol), $Cu(OAc)_2$ (8 mg, 0.041 mmol), TMEDA (5 mg, 0.041 mmol), Na_2CO_3 (87 mg, 0.82 mmol), and 2,2,2-trichloroethanol (**2a**) (1.0 mL). The mixture was stirred at 110 ºC in an oil bath for 15 min. The crude reaction mixture was purified by flash column chromatography on silica gel with hexane/EtOAc (8:2) as eluent to afford the dichloroalkyl-arylation product **3w** as a 1:1 mixture of diastereomers (129 mg, 0.302 mmol, 74%) as a yellow solid. mp: 132-134 ºC. **^{1}H NMR** (400 MHz, $CDCl_3$): δ 7.83 (d, J = 7.1 Hz, 1H), 7.80 (d, J = 7.1 Hz, 1H), 7.40 – 7.24 (m, 6H), 6.84 (d, J = 7.5 Hz, 2H), 4.85 (p, J = 7.2 Hz, 2H), 4.42 (dd, J = 12.8, 2.8 Hz, 2H), 4.31 (dd, J = 12.1, 6.1 Hz, 2H), 4.19 – 4.09 (m, 2H), 3.96 – 3.85 (m, 4H), 3.83 (s, 3H), 3.81 (s, 3H), 2.90 – 2.72 (m, 6H), 2.44 – 2.30 (m, 2H), 2.14 – 2.05 (m, 2H), 1.81 (tq, J = 11.1, 3.3 Hz, 2H), 1.57 (d, J = 7.1 Hz, 3H), 1.54 (d, J = 7.1 Hz, 3H). **^{13}C{^{1}H} NMR** (100 MHz, $CDCl_3$): δ 174.1, 174.1, 165.9, 165.8, 147.3, 147.3, 136.3, 124.7, 124.7, 122.7, 122.6, 122.4, 118.6, 110.2, 103.8, 103.8, 92.4, 70.1, 70.0, 52.8, 52.7, 48.4, 48.3, 45.5, 42.6, 30.6, 30.6, 22.8, 19.2, 19.1, 17.1. **FT-IR** (ATR) ν_{max}: 3380, 2983, 2951, 2921, 2868, 2851, 1741, 1637, 1607, 1520, 1483, 1473, 1452, 1428, 1380, 1360, 1309, 1287, 1206, 1196, 1163, 1126, 1081, 1028, 998, 982, 962, 919, 876, 857, 829, 786, 741, 728, 709, 693, 673, 637, 589, 558, 524, 502, 468, 432, 408 cm^{-1}. **HRMS**-(ESI) (m/z) calcd for $C_{20}H_{25}Cl_2N_2O_4$ [M+H]$^+$: 427.1191; found: 427.1195.

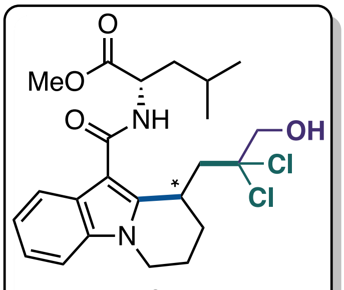

3xa

Methyl (9-(2,2-dichloro-3-hydroxypropyl)-6,7,8,9-tetrahydropyrido[1,2-*a*]indole-10-carbonyl)-*L*-leucinate (3xa). Following the general procedure above, using methyl (1-(pent-4-en-1-yl)-1H-indole-3-carbonyl)-L-leucinate (**1x**) (146 mg, 0.41 mmol), $Cu(OAc)_2$ (8 mg, 0.041 mmol), TMEDA (5 mg, 0.041 mmol), Na_2CO_3 (87 mg, 0.82 mmol), and 2,2,2-trichloroethanol (**2a**) (1.0 mL). The mixture was stirred at 110 ºC in an oil bath for 15 min. The crude reaction mixture was purified by flash column chromatography on silica gel with hexane/EtOAc (8:2) as eluent to afford the dichloroalkyl-arylation product **3xa** as a 1:1 mixture of diastereomers (127 mg, 0.271 mmol, 66%) as a white solid. mp: 145-148 ºC. **^{1}H NMR** (400 MHz, $CDCl_3$): δ 7.81 (d, J = 7.0 Hz, 1H), 7.77 (d, J = 6.8 Hz, 1H), 7.39 – 7.23 (m, 6H), 6.62 (d, J = 8.5 Hz, 1H), 6.59 (d, J = 8.6 Hz, 1H), 4.90 (qd, J = 8.3, 5.0 Hz, 2H), 4.40 (dd, J = 12.8, 2.0 Hz, 2H), 4.35 – 4.27 (m, 2H), 4.18 – 4.05 (m, 2H), 3.97 – 3.84 (m, 4H), 3.81 (s, 3H), 3.78 (s, 3H), 2.91 – 2.70 (m, 6H), 2.44 – 2.28 (m, 2H), 2.15 – 2.03 (m, 2H), 1.89 – 1.64 (m, 8H), 1.06 – 0.95 (m, 12H). **^{13}C{^{1}H} NMR** (100 MHz, $CDCl_3$): δ 174.0, 174.0, 166.2, 166.0, 147.2, 147.1, 136.3, 136.2, 124.7, 124.7, 122.6, 122.6, 122.3, 122.3, 118.5,

118.5, 110.2, 110.2, 103.9, 103.9, 92.4, 92.4, 70.2, 70.0, 52.6, 52.5, 51.1, 50.9, 45.5, 45.5, 42.6, 42.6, 42.2, 42.1, 30.6, 25.2, 25.2, 23.0, 23.0, 22.8, 22.8, 22.3, 22.3, 17.2, 17.1. **FT-IR** (ATR) ν_{max}: 3519, 3440, 3334, 2954, 2921, 2870, 1723, 1643, 1606, 1525, 1473, 1454, 1427, 1364, 1321, 1259, 1204, 1165, 1108, 1075, 1002, 979, 918, 895, 860, 830, 784, 751, 734, 701, 674, 592, 531, 485, 433 cm^{-1}. **HRMS**-(DART) (*m/z*) calcd for $C_{23}H_{31}Cl_2N_2O_4$ $[M+H]^+$: 469.1660; found: 469.1672.

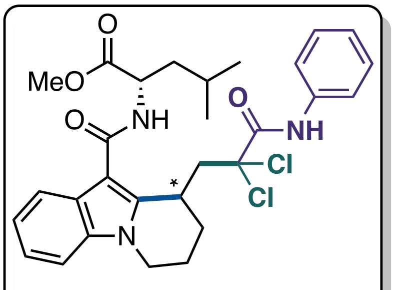

3xb

Methyl (9-(2,2-dichloro-3-oxo-3-(phenylamino)propyl)-6,7,8,9-tetrahydropyrido[1,2-*a*]indole-10-carbonyl)-*L*-leucinate (3xb). Following the general procedure above, using methyl (1-(pent-4-en-1-yl)-1*H*-indole-3-carbonyl)-*L*-leucinate (**1x**) (181 mg, 0.508 mmol), $Cu(OAc)_2$ (9 mg, 0.051 mmol), TMEDA (6 mg, 0.051 mmol), Na_2CO_3 (108 mg, 1.016 mmol), 2,2,2-trichloro-*N*-phenylacetamide (**2c**) (363 mg, 1.523 mmol, 3.0 equiv.), and DMF (1.0 mL). The mixture was stirred at 110 ºC in an oil bath for 1 h. The crude reaction mixture was purified by flash column chromatography on silica gel with hexane/EtOAc (9:1) as eluent to afford the dichloroalkyl-arylation product **3xb** as a 1:1 mixture of diastereomers (203 mg, 0.363 mmol, 72%) as an orange oil. ^{1}**H NMR** (400 MHz, $CDCl_3$): δ 9.25 (bs, 2H), 7.86 – 7.66 (m, 6H), 7.42 – 7.32 (m, 6H), 7.32 – 7.26 (m, 4H), 7.18 (q, *J* = 7.3 Hz, 2H), 6.50 (d, *J* = 8.9 Hz, 1H), 6.37 (d, *J* = 8.2 Hz, 1H), 5.01 (td, *J* = 9.0, 4.7 Hz, 1H), 4.90 (td, *J* = 8.3, 5.2 Hz, 1H), 4.38 – 4.25 (m, 4H), 3.89 (qd, *J* = 11.2, 5.7 Hz, 2H), 3.79 (s, 3H), 3.77 (s, 3H), 3.41 (dd, *J* = 15.2, 4.1 Hz, 2H), 3.12 (ddd, *J* = 24.2, 15.2, 11.2 Hz, 2H), 2.42 – 2.26 (m, 4H), 2.09 – 1.99 (m, 2H), 1.90 – 1.66 (m, 8H), 1.09 – 0.96 (m, 12H). ^{13}**C{^{1}H} NMR** (100 MHz, $CDCl_3$): δ 174.2, 174.1, 165.8, 165.5, 163.1, 145.0, 144.9, 137.5, 137.5, 136.1, 136.1, 129.1, 129.0, 125.2, 125.1, 124.9, 124.9, 122.3, 122.2, 122.2, 122.2, 120.6, 120.6, 118.6, 118.5, 110.1, 105.3, 105.2, 86.7, 86.6, 52.5, 51.1, 50.8, 46.7, 46.5, 42.6, 42.4, 42.3, 41.7, 31.5, 31.2, 25.2, 25.2, 23.1, 22.9, 22.6, 22.3, 22.2, 17.8, 17.7. **FT-IR** (ATR) ν_{max}: 3447, 3357, 3289, 3135, 3053, 2952, 2924, 2870, 2949, 1735, 1686, 1635, 1599, 1526, 1499, 1441, 1365, 1317, 1267, 1237, 1201, 1164, 1011, 977, 901, 829, 783, 750, 734, 689, 639, 594, 568, 557, 505 cm^{-1}. **HRMS**-(ESI) (*m/z*) calcd for $C_{29}H_{34}Cl_2N_3O_4$ $[M+H]^+$: 558.1926; found: 558.1920.

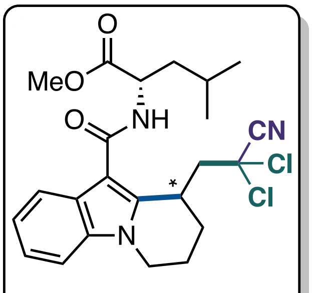

3xc

Methyl (9-(2,2-dichloro-2-cyanoethyl)-6,7,8,9-tetrahydropyrido[1,2-*a*]indole-10-carbonyl)-*L*-leucinate (3xc). Following the general procedure above, using methyl (1-(pent-4-en-1-yl)-1*H*-indole-3-carbonyl)-*L*-leucinate (**1x**) (146 mg, 0.41 mmol), $Cu(OAc)_2$ (8 mg, 0.041 mmol), TMEDA (5 mg, 0.041 mmol), Na_2CO_3 (87 mg, 0.82 mmol), and trichloroacetonitrile (**2d**) (1.0 mL). The mixture was stirred at 110 ºC in an oil bath for 15 min. The crude reaction mixture was purified by flash column chromatography on silica gel with hexane/EtOAc (9:1) as eluent to afford the dichloroalkyl-arylation product **3xc** as a 1:1 mixture of diastereomers (35 mg, 0.075 mmol, 18%) as a yellow solid. mp: 115-119 ºC. ^{1}**H NMR** (400 MHz, $CDCl_3$): δ 7.81 (d, *J* = 7.6 Hz, 2H), 7.38 – 7.26 (m, 6H), 6.39 (d, *J* = 7.8 Hz, 1H), 6.33 (d, *J* = 8.0 Hz, 1H), 5.01 – 4.84 (m, 2H), 4.47 – 4.37 (m, 2H), 4.31 (dd, *J* = 12.4, 5.2 Hz, 2H), 3.92 (td, *J* = 11.7, 5.7 Hz, 2H), 3.78 (s, 3H), 3.77 (s, 3H), 3.39 (dd, *J* = 14.8, 2.9 Hz, 2H), 2.84 – 2.73 (m, 2H), 2.57 – 2.47 (m, 2H), 2.33 – 2.20 (m, 2H), 2.19 – 2.09 (m, 2H), 2.03 – 1.91 (m, 2H), 1.85 – 1.74 (m, 4H), 1.69 (q, *J* = 8.6 Hz, 2H), 1.06 – 0.97 (m, 12H). ^{13}**C{^{1}H} NMR** (100 MHz, $CDCl_3$): δ 174.4, 165.1, 143.4, 136.2, 124.9, 122.4, 122.4, 118.9, 116.3, 110.1, 106.1, 67.1, 52.5, 50.7, 48.6, 42.7, 42.3, 31.5, 25.3, 23.2, 22.3, 22.0, 18.1. **FT-IR** (ATR) ν_{max}: 3436, 3175, 3050, 2954, 2925, 2870, 2851, 1736, 1633, 1535, 1485, 1454, 1433, 1367, 1341, 1321, 1267, 1235, 1210, 1197, 1166, 1120, 1066, 1023, 981, 925, 825, 779, 748, 735, 677, 615, 583, 532, 497, 472, 441 cm^{-1}. **HRMS**-(DART) (*m/z*) calcd for $C_{23}H_{28}Cl_2N_3O_3$ $[M+H]^+$: 464.1508; found: 464.1510.

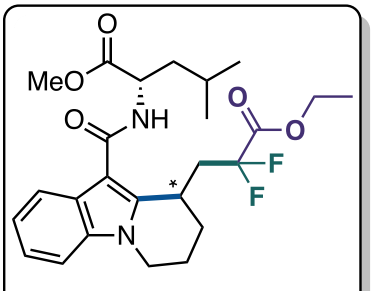

3xd

Methyl (9-(3-ethoxy-2,2-difluoro-3-oxopropyl)-6,7,8,9-tetrahydropyrido[1,2-*a*]indole-10-carbonyl)-*L*-leucinate (3xd). Following the general procedure above, using methyl (1-(pent-4-en-1-yl)-1*H*-indole-3-carbonyl)-*L*-leucinate (**1x**) (95 mg, 0.267 mmol), $Cu(OAc)_2$ (5 mg, 0.027 mmol), TMEDA (3 mg, 0.027 mmol), Na_2CO_3 (56 mg, 0.533 mmol), and ethyl chlorodifluoroacetate (**2e**) (1.0 mL). The mixture was stirred at 110 ºC in an oil bath for 12 h. The crude reaction mixture was purified by flash column chromatography on silica gel with hexane/EtOAc (9:1) as eluent to afford the dichloroalkyl-arylation product **3xd** as a 1:1 mixture of diastereomers (44 mg, 0.092 mmol, 35%) as a yellow oil. *Isomer I*: ^{1}**H NMR** (400 MHz, $CDCl_3$): δ 7.83 – 7.79 (m, 1H), 7.36 – 7.32 (m, 1H), 7.27 (ddd, *J* = 6.6, 4.3, 1.7 Hz, 2H), 6.30 (d, *J* = 8.5 Hz, 1H), 4.89 (td, *J* = 8.7, 5.1 Hz, 1H), 4.39 (q, *J* = 7.1 Hz, 2H), 4.32 – 4.16 (m, 2H), 3.89 (td, *J* = 11.5, 5.5 Hz, 1H), 3.78 (s, 3H), 2.86 – 2.69 (m, 1H), 2.40 (ddt, *J* = 17.5, 15.0, 11.3 Hz, 1H), 2.33 – 2.17 (m, 2H), 2.13 – 2.03 (m, 1H), 1.97 – 1.86 (m, 1H), 1.84 – 1.74 (m, 2H), 1.73 – 1.63 (m, 1H), 1.39 (t, *J* = 7.2 Hz, 3H), 1.00 (t, *J* = 6.2 Hz, 6H). ^{13}**C{^{1}H} NMR** (100 MHz, $CDCl_3$): δ 174.4, 165.3, 164.2 (t, $J_{C\text{-}F}$ = 31.6 Hz), 145.1, 136.1, 125.0, 122.1, 122.0, 118.7, 116.3 (t, $J_{C\text{-}F}$ = 251.6 Hz), 109.9, 105.6, 63.1, 52.4, 50.9, 42.7, 42.1, 37.6 (t, $J_{C\text{-}F}$ = 22.0 Hz), 27.8, 25.2, 23.5, 23.0, 22.3, 18.0, 14.1. *Isomer II*: ^{1}**H NMR** (400 MHz, $CDCl_3$): δ 7.82 – 7.78 (m, 1H), 7.36 – 7.32 (m, 1H), 7.29 – 7.24 (m, 2H), 6.29 (d, *J* = 8.6 Hz, 1H), 4.90 (td, *J* = 8.8, 5.1 Hz, 1H), 4.37 (q, *J* = 7.2 Hz, 2H), 4.28 (ddd, *J* = 12.0, 6.2, 2.2 Hz, 1H), 4.20 – 4.12 (m, 1H), 3.89 (td, *J* = 11.2, 5.6 Hz, 1H), 3.77 (s, 3H), 2.83 (tdd, *J* = 20.2, 15.0, 3.0 Hz, 1H), 2.41 (ddt, *J* = 17.9, 15.0, 11.3 Hz, 1H), 2.32 – 2.17 (m, 2H), 2.12 – 2.03 (m, 1H), 1.96 – 1.85 (m, 1H), 1.85 – 1.66 (m, 3H), 1.38 (t, *J* = 7.2 Hz, 3H), 1.02 (d, *J* = 6.1 Hz, 3H), 1.00 (d, *J* = 6.4 Hz, 3H). ^{13}**C{^{1}H} NMR** (100 MHz, $CDCl_3$): δ 174.4, 165.2, 164.2 (t, $J_{C\text{-}F}$ = 32.3 Hz), 145.1, 136.1, 125.0, 122.1, 122.0, 118.8, 116.3 (t, $J_{C\text{-}F}$ = 251.6 Hz), 109.9, 105.7, 63.1, 52.3, 50.7, 42.7, 42.1, 37.6 (t, $J_{C\text{-}F}$ = 21.9 Hz), 27.8, 25.3, 23.5, 23.1, 22.2, 17.9, 14.1. **FT-IR** (ATR) ν_{max}: 3443, 2955, 2871, 1761, 1739, 1643, 1531, 1457, 1431, 1368, 1312, 1273, 1232, 1205, 1164, 1122, 1103, 1061, 1019, 982, 955, 923, 852, 792, 776, 739, 688, 652, 629, 561, 510, 474, 439 cm^{-1}. **HRMS**-(ESI) (*m/z*) calcd for $C_{25}H_{33}F_2N_2O_5$ $[M+H]^+$: 479.2358; found: 479.2360.

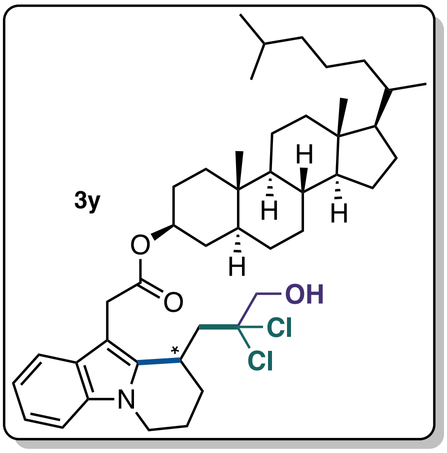

(3*S*,5*S*,8*R*,9*S*,10*S*,13*R*,14*S*,17*R*)-10,13-Dimethyl-17-((*S*)-6-methylheptan-2-yl)hexadecahydro-1*H*-cyclopenta[*a*]phenanthren-3-yl 2-(9-(2,2-dichloro-3-hydroxypropyl)-6,7,8,9-tetrahydropyrido[1,2-*a*]indol-10-yl)acetate (3Y). Following the general procedure above, using methyl (3*S*,5*S*,8*R*,9*S*,10*S*,13*R*,14*S*,17*R*)-10,13-dimethyl-17-((*S*)-6-methylheptan-2-yl)hexadecahydro-1*H*-cyclopenta[*a*]phenanthren-3-yl 2-(1-(pent-4-en-1-yl)-1*H*-indol-3-yl)acetate (**1y**) (108 mg, 0.176 mmol), $Cu(OAc)_2$ (3 mg, 0.018 mmol), TMEDA (2 mg, 0.018 mmol), Na_2CO_3 (38 mg, 0.352 mmol), and 2,2,2-trichloroethanol (**2a**) (1.0 mL). The mixture was stirred at 110 ºC in an oil bath for 15 min. The crude reaction mixture was purified by flash column chromatography on silica gel with hexane/EtOAc (8:2) as eluent to afford the dichloroalkyl-arylation product **3y** as a 1:1 mixture of diastereomers (55 mg, 0.076 mmol, 43%) as a brown oil. **^{1}H NMR** (400 MHz, $CDCl_3$): δ 7.59 (d, *J* = 7.8 Hz, 2H), 7.25 (d, *J* = 7.2 Hz, 2H), 7.17 (td, *J* = 8.1, 7.6, 1.3 Hz, 2H), 7.11 (t, *J* = 7.4 Hz, 2H), 4.68 (tt, *J* = 11.1, 4.9 Hz, 2H), 4.26 (ddd, *J* = 10.5, 5.2, 2.0 Hz, 2H), 4.00 (dd, *J* = 12.3, 1.3 Hz, 2H), 3.95 (d, *J* = 12.9 Hz, 2H), 3.85 – 3.71 (m, 8H), 2.83 (ddd, *J* = 15.6, 4.5, 2.0 Hz, 2H), 2.69 (dd, *J* = 15.6, 10.2 Hz, 2H), 2.54 (d, *J* = 13.4 Hz, 2H), 1.98 – 1.93 (m, 2H), 1.83 – 1.76 (m, 4H), 1.70 – 1.65 (m, 2H), 1.56 – 1.49 (m, 8H), 1.38 – 1.21 (m, 20H), 1.15 – 1.08 (m, 12H), 1.03 – 0.94 (m, 10H), 0.92 – 0.89 (m, 10H), 0.88 – 0.85 (m, 18H), 0.81 (s, 6H), 0.65 – 0.63 (m, 8H). **^{13}C{^{1}H} NMR** (100 MHz, $CDCl_3$): δ 172.1, 137.5, 136.1, 128.0, 121.1, 119.9, 118.7, 118.7, 108.9, 103.0, 93.3, 74.6, 72.8, 56.6, 56.4, 54.3, 46.4, 44.8, 44.8, 42.7, 42.4, 40.1, 39.7, 36.9, 36.3, 35.9, 35.6, 35.6, 34.1, 32.1, 30.9, 30.9, 29.4, 28.7, 28.4, 28.2, 27.6, 24.8, 24.3, 24.0, 23.0, 22.7, 21.3, 18.8, 18.6, 12.4, 12.2. **FT-IR** (ATR) ν_{max}: 3442, 3049, 2929, 2865, 1724, 1613, 1565, 1461, 1363, 1332, 1266, 1243, 1164, 1147, 1131, 1075, 1014, 957, 928, 863, 843, 805, 737, 677, 597, 572, 541, 465, 433 cm^{-1}. **HRMS**-(ESI) (*m*/*z*) calcd for $C_{44}H_{66}Cl_2NO_3$ $[M+H]^+$: 726.4420; found: 726.4402.

1.3 Derivatization of 2,2-dichloro-3-(6,7,8,9-tetrahydropyrido[3,2-*b*]indolizin-6-yl)propan-1-ol (3h)

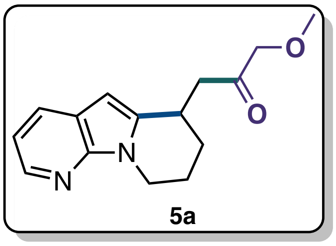

1-Methoxy-3-(6,7,8,9-tetrahydropyrido[3,2-*b*]indolizin-6-yl)propan-2-one (5a). Following the procedure described by Bao and co-workers.[15] A round-bottom flask was charged with a mixture of 2,2-dichloro-3-(6,7,8,9-tetrahydropyrido[3,2-*b*]indolizin-6-yl)propan-1-ol (**3h**) (100 mg, 0.334 mmol), NaOH (27 mg, 0.668 mmol), and methanol (2.2 mL). The reaction mixture was stirred at 40 °C for 2.5 h, and then was cooled to room temperature. The solvent was evaporated, the residue dissolved in EtOAc and washed with water. The organic phase was dried over anhydrous Na_2SO_4 and evaporated under reduced pressure. The residue obtained was purified by flash column chromatography on silica gel with hexane/EtOAc (6:4) as eluent to afford the ketone **5a** (49 mg, 0.190 mmol, 57%) as a yellow oil. **^{1}H NMR** (400 MHz, $CDCl_3$): δ 8.24 (d, *J* = 4.9 Hz, 1H), 7.80 (d, *J* = 7.8 Hz, 1H), 7.03 (dd, *J* = 7.7, 4.9 Hz, 1H), 6.15 (s, 1H), 4.45 (dt, *J* = 12.3, 4.9 Hz, 1H), 4.09 – 4.00 (m, 1H), 4.05 (s, 2H), 3.69 – 3.60 (m, 1H), 3.45 (s, 3H), 3.06 (dd, *J* = 17.6, 5.5 Hz, 1H), 2.79 (dd, *J* = 17.6, 7.8 Hz, 1H), 2.24 – 2.11 (m, 2H), 2.09 – 1.98 (m, 1H), 1.60 – 1.49 (m, 1H). **^{13}C{^{1}H} NMR** (100 MHz, $CDCl_3$): δ 207.2, 141.3, 141.1, 128.0, 121.0, 116.1, 95.6, 78.3, 59.6, 44.4, 41.5, 30.2, 29.8, 27.5, 21.9. **FT-IR** (ATR) ν_{max}: 3045, 2995, 2950, 2925, 2881, 2856, 1910, 1875, 1844, 1723, 1593, 1565, 1525, 1478, 1431, 1399, 1360, 1349, 1306, 1290, 1252, 1191, 1144, 1109, 1037, 1004, 975, 941, 899, 833, 813, 768, 717, 700, 670, 582, 565, 533, 455, 437 cm^{-1}. **HRMS**-(ESI) (*m*/*z*) calcd for $C_{15}H_{19}N_2O_2$ $[M+H]^+$: 259.1447; found: 259.1454.

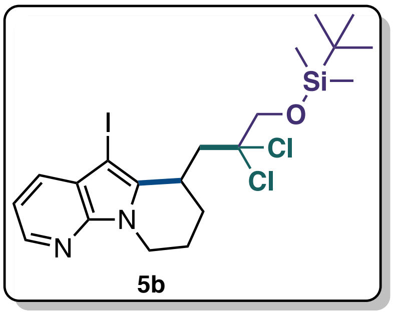

6-(3-((*Tert*-butyldimethylsilyl)oxy)-2,2-dichloropropyl)-5-iodo-6,7,8,9-tetrahydropyrido[3,2-*b*]indolizine (5b). Following the procedure described by Stawinski and co-workers.[16] 2,2-dichloro-3-(6,7,8,9-tetrahydropyrido[3,2-*b*]indolizin-6-yl)propan-1-ol (**3h**) (273 mg, 0.912 mmol), *N*-methylimidazole (227 mg, 2.737 mmol) and iodine (463 mg, 1.825 mmol) were dissolved in DCM (2.73 mL). TBDMSCl (156 mg, 1.004 mmol) was added, and the reaction mixture was stirred at r.t. for 30 min. The solvent was evaporated, the residue dissolved in EtOAc and washed with a saturated solution of $Na_2S_2O_3$. The organic phase was dried over anhydrous Na_2SO_4 and evaporated under reduced pressure. The residue obtained was purified by flash column chromatography on silica gel with hexane/EtOAc (9:1) as eluent to afford the TBDMS ether **5b** (382 mg, 0.708 mmol, 78%) as a yellow solid. mp: 75-78 ºC. **^{1}H NMR** (400 MHz, $CDCl_3$): δ 8.28 (dt, *J* = 4.8, 1.4 Hz, 1H), 7.67 (ddd, *J* = 7.9, 3.3, 1.5 Hz, 1H), 7.12 (ddd, *J* = 8.2, 4.8, 2.4 Hz, 1H), 4.60 – 4.53 (m, 1H), 4.02, 3.99 (AB system, *J* = 10.9 Hz, 2H), 3.97 – 3.90 (m, 1H), 3.77 (ddt, *J* = 10.9, 5.3, 2.7 Hz, 1H), 2.77, 2.52 (ABX system, *J* = 15.2, 2.9, 11.1 Hz, 2H), 2.74 – 2.67 (m, 1H), 2.28 – 2.17 (m, 1H), 2.12 – 2.03 (m, 1H), 1.91 (ddt, *J* = 17.3, 13.8, 3.4 Hz, 1H), 0.92 (s, 9H), 0.13 (d, *J* = 2.5 Hz, 6H). **^{13}C{^{1}H} NMR** (100 MHz, $CDCl_3$): δ 147.8, 143.0, 141.1, 128.3, 123.6, 116.9, 91.6, 73.7, 53.7, 44.2, 42.0, 31.8, 25.9, 23.5, 18.5, 18.3, -5.1, -5.2. **FT-IR** (ATR) ν_{max}: 2995, 2950, 2925, 2882, 2855, 1910, 1875, 1844, 1739, 1711, 1592, 1564, 1524, 1477, 1431, 1399, 1360, 1350, 1306, 1289, 1251, 1190, 1144, 1113, 1037, 1004, 974, 954, 940, 898, 832, 814, 783, 767, 717, 699, 671, 593, 582, 564, 532, 468, 455, 438 cm^{-1}. **HRMS**-(ESI) (*m*/*z*) calcd for $C_{20}H_{30}Cl_2IN_2OSi$ $[M+H]^+$: 539.0549; found: 539.0560.

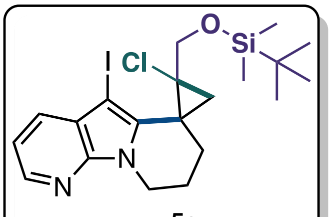

2-(((*Tert*-butyldimethylsilyl)oxy)methyl)-2-chloro-5'-iodo-8',9'-dihydro-7'*H*-spiro[cyclopropane-1,6'-pyrido[3,2-*b*]indolizine] (5c). Following the procedure described by Rong and co-workers.[17] The mixture of 6-(3-((*Tert*-butyldimethylsilyl)oxy)-2,2-dichloropropyl)-5-iodo-6,7,8,9-tetrahydropyrido[3,2-*b*]indolizine (**5b**) (50 mg, 0.093 mmol), *t*-BuOK (32 mg, 0.278 mmol) and THF (1.9 mL) was added into a round-bottom flask and stirred at 50 ℃ for 2.5 h under nitrogen atmosphere. After the reaction was completed, the solution was concentrated under reduced pressure and the residue obtained was purified by flash column chromatography on silica gel with hexane/EtOAc (9:1) as eluent to afford the spiro-cyclopropane **5c** (34 mg, 0.068 mmol, 73%) as a yellow solid. mp: 100-103 ºC. **^{1}H NMR** (400 MHz, $CDCl_3$): δ 8.28 (dd, *J* = 4.8, 1.5 Hz, 1H), 7.64 (dd, *J* = 7.8, 1.5 Hz, 1H), 7.10 (dd, *J* = 7.8, 4.8 Hz, 1H), 4.52 – 4.44 (m, 1H), 4.37 – 4.28 (m, 1H), 3.96 (d, *J* = 11.3 Hz, 1H), 3.58 (d, *J* = 11.5 Hz, 1H), 3.13 (d, *J* = 7.5 Hz, 1H), 2.48 – 2.36 (m, 1H), 2.23 – 2.04 (m, 3H), 1.30 (d, *J* = 7.6 Hz, 1H), 0.60 (s, 9H), -0.19 (s, 3H), -0.49 (s, 3H). **^{13}C{^{1}H} NMR** (100 MHz, $CDCl_3$): δ 147.9, 143.6, 135.4, 128.9, 124.1, 116.8, 67.6, 56.3, 50.4, 41.2, 30.1, 29.3, 25.5, 23.6, 21.4, 18.0, -5.7, -6.0. **FT-IR** (ATR) ν_{max}: 3045, 2952, 2925, 2888, 2851, 1910, 1875, 1844, 1718, 1589, 1567, 1517, 1479, 1459, 1429, 1396, 1370, 1329, 1304, 1284, 1249, 1195, 1159, 1102, 1061, 1030, 1005, 959, 941, 835, 774, 746, 664, 631, 595, 566, 541, 496, 459, 431 cm^{-1}. **HRMS**-(ESI) (*m*/*z*) calcd for $C_{20}H_{29}ClIN_2OSi$ [M+H]$^+$: 503.0782; found: 503.0790.

1.4 Radical trapping experiment

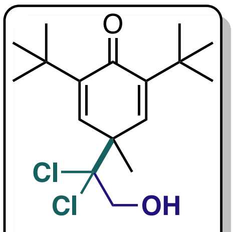

2,6-di-*tert*-butyl-4-(1,1-dichloro-2-hydroxyethyl)-4-methylcyclohexa-2,5-dien-1-one (6). Following the general procedure 1.2, using dibutylhydroxytoluene (90 mg, 0.41 mmol), $Cu(OAc)_2$ (8 mg, 0.041 mmol), TMEDA (5 mg, 0.041 mmol), Na_2CO_3 (87 mg, 0.82 mmol), and 2,2,2-trichloroethanol (**2a**) (1.0 mL). The mixture was stirred at 110 ºC in an oil bath for 15 min. The crude reaction mixture was purified by flash column chromatography on silica gel with hexane/DCM (1:1) as eluent to afford the product **6** (108 mg, 0.324 mmol, 79%) as brown crystals. mp: 125–130 ºC. **^{1}H NMR** (400 MHz, $CDCl_3$): δ 6.76 (s, 2H), 3.81 (s, 2H), 1.57 (s, 3H), 1.23 (s, 18H). **^{13}C{^{1}H} NMR** (100 MHz, $CDCl_3$): δ 185.6, 148.5, 140.3, 100.4, 70.5, 48.5, 35.3, 29.5, 23.4. **FT-IR** (ATR) ν_{max}: 3499, 2996, 2952, 2867, 1755, 1735, 1654, 1632, 1482, 1456, 1388, 1371, 1361, 1298, 1245, 1233, 1200, 1173, 1140, 1100, 1013, 932, 878, 809, 731, 718, 654, 580, 550, 510, 440 cm^{-1}. **HRMS**-(DART) (*m*/*z*) calcd for $C_{17}H_{27}Cl_2O_2$ [M+H]$^+$: 333.1388; found: 333.1380.

Part II X-ray crystallographic information

The structure of **3f**, **3h** and **6** were determined by X-ray diffraction on a Bruker D8 Venture diffractometer using CuKα radiation (λ= 1.5417 Å). Proper crystals were obtained in hexane/dichloromethane (30/1) by slow evaporation of the solvent at room temperature. The obtained data sets were processed with APEX3 software.[18] The phase problem was solved by direct methods using SHELXS-2014.[19] Parameters of obtained models were refined by full-matrix least-squares on F^2 using SHELXL-2016/4.[20] Non-hydrogen atoms were refined anisotropically, and hydrogen atoms were allowed to ride on the respective atoms. Calculations were performed using SHELXL-2014/7.[21] Figures were prepared with ORTEP-3 (ver. 2014.1).[22] Molecular geometry of compounds **3f**, **3h** and **6** observed in the crystal structure are shown in Figure S1, and crystal data and structure refinement results are shown in Table S1. Crystallographic data for the structures presented in this paper have been deposited with the Cambridge Crystallographic Data Centre as a supplementary publication number. CCDC 2491320 (**3f**), 2491321 (**3h**), and 2491322 (**6**). Copies of the data can be obtained, free of charge, on application to CCDC, 12 Union Road, Cambridge CB2 1EZ, UK, (fax: +44-(0)1223-336033 or e-mail: deposit@ccdc.cam.ac.uk/data_request/cif).

Table S1. Crystal data and structure refinement results for compounds **3f**, **3h** and **6**.

Compound	1-(2,2-Dichloro-3-hydroxypropyl)-2,3-dihydro-1*H*-pyrrolo[1,2-*a*]indole-9-carbaldehyde (**3f**)	2,2-Dichloro-3-(6,7,8,9-tetrahydropyrido[3,2-*b*]indolizin-6-yl)propan-1-ol (**3h**)	2,6-di-*tert*-butyl-4-(1,1-dichloro-2-hydroxyethyl)-4-methylcyclohexa-2,5-dien-1-one (**6**)
Empirical moiety formula	$C_{15}H_{15}Cl_2NO_2$	$C_{14}H_{16}Cl_2N_2O$	$C_{17}H_{26}Cl_2O_2$
Formula weight (g/mol)	312.19	299.20	333.29
Temperature (K)	150	150	150
Wavelength (Å)	0.71073	0.71073	0.71073
Crystal system	Monoclinic	Monoclinic	Monoclinic
Space group	$P2_1/c$	$P2_1/c$	$P2_1$
Unite cell dimensions	a= 9.2875(4) Å<br>b= 15.4275(6) Å<br>c= 9.6273(4) Å<br>α= 90º<br>ß= 93.781(1)º<br>γ= 90º	a= 10.0670(5) Å<br>b= 18.3113(8) Å<br>c= 7.5966(3) Å<br>α= 90º<br>ß= 93.391(1)º<br>γ= 90º	a= 6.3035(4) Å<br>b= 15.4582(10) Å<br>c= 9.6706(7) Å<br>α= 90º<br>ß= 108.507(2)º<br>γ= 90º
Volume ($Å^3$)	1376.43(10)	1397.90(11)	893.58(10)
Z	4	4	2
D_{calc} (g/cm^3)	1.506	1.442	1.239
μ (mm^{-1})	0.471	0.481	0.365
F(000)	648	632.2	356
Crystal size (mm^3)	0.257 × 0.334 × 0.451	0.254 × 0.259 × 0.44	0.12 × 0.18 × 0.4
Θ range	2.5º to 27.49º	2.31º to 27.48º	2.64º to 27.5º
Index ranges	–12 ≤h ≤ 11,<br>–20≤ k ≤ 19,<br>–12 ≤ l ≤ 12	–13 ≤h ≤ 13,<br>–23 ≤ k ≤ 23,<br>–9 ≤ l ≤ 9	–8 ≤h ≤ 8,<br>–20 ≤ k ≤ 20,<br>–12 ≤ l ≤ 12
Refl. collected	27363	21114	25051
Refl. unique	3107	3173	4074
Absorption correction	Multi-scan	None	Multi-scan
Refinement method	Full-matrix least-squares on F^2	Full-matrix least-squares on F^2	Full-matrix least-squares on F^2
Data/restraints/parameters	3107/1/182	3173/106/221	4074/79/227
GooF on F2	1.194	1.008	0.885
Final R indices [I>2sigma(I)]	R1= 0.0349,<br>wR2= 0.0822	R1= 0.0964,<br>wR2= 0.2097	R1= 0.0265,<br>wR2= 0.0724
R indices (all data)	R1= 0.0418,<br>wR2= 0.0896	R1= 0.1247,<br>wR2= 0.2284	R1= 0.0275,<br>wR2= 0.0743
$\Delta\rho_{max}$, $\Delta\rho_{min}$ ($e \cdot Å^{-3}$)	0.37 and –0.277	0.949 and –0.568	0.268 and –0.204

a)

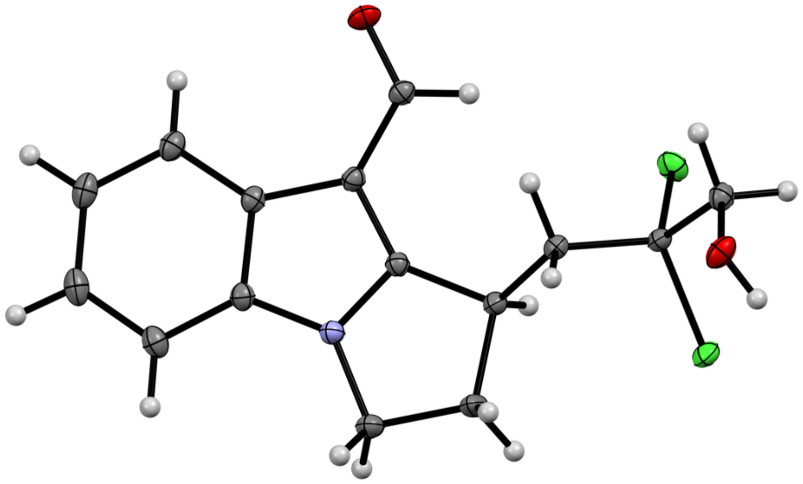

b)

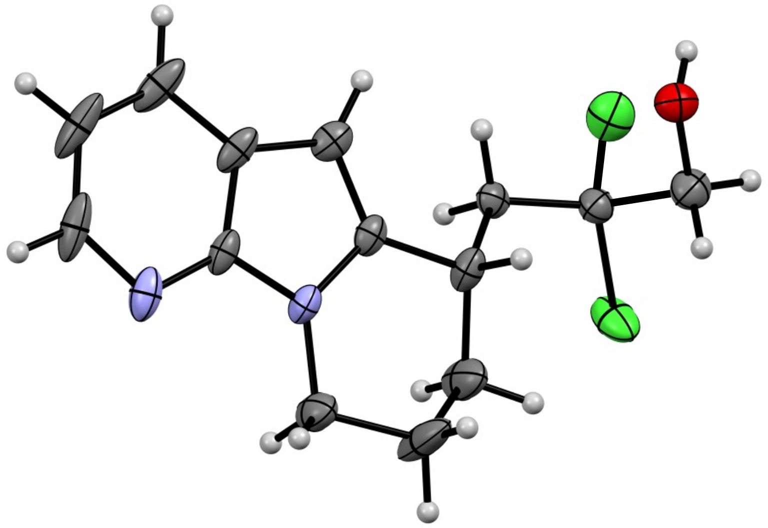

c)

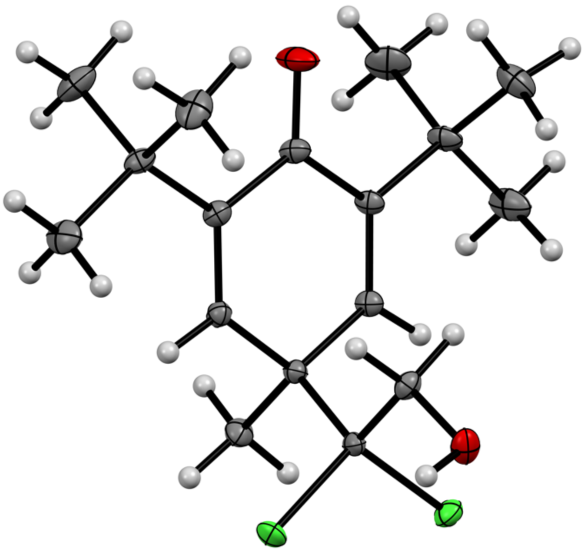

Figure S1. Molecular geometry observed in the crystal structure of compounds **3f** (a), **3h** (b), and **6** (c). Displacement ellipsoids of non-hydrogen atoms are drawn at the 30% probability level. Hydrogen atoms are presented as small spheres with an arbitrary radius.

Part III ^{1}H and $^{13}C\{^{1}H\}$ NMR spectra

^{1}H **NMR** (400 MHz, $CDCl_3$)

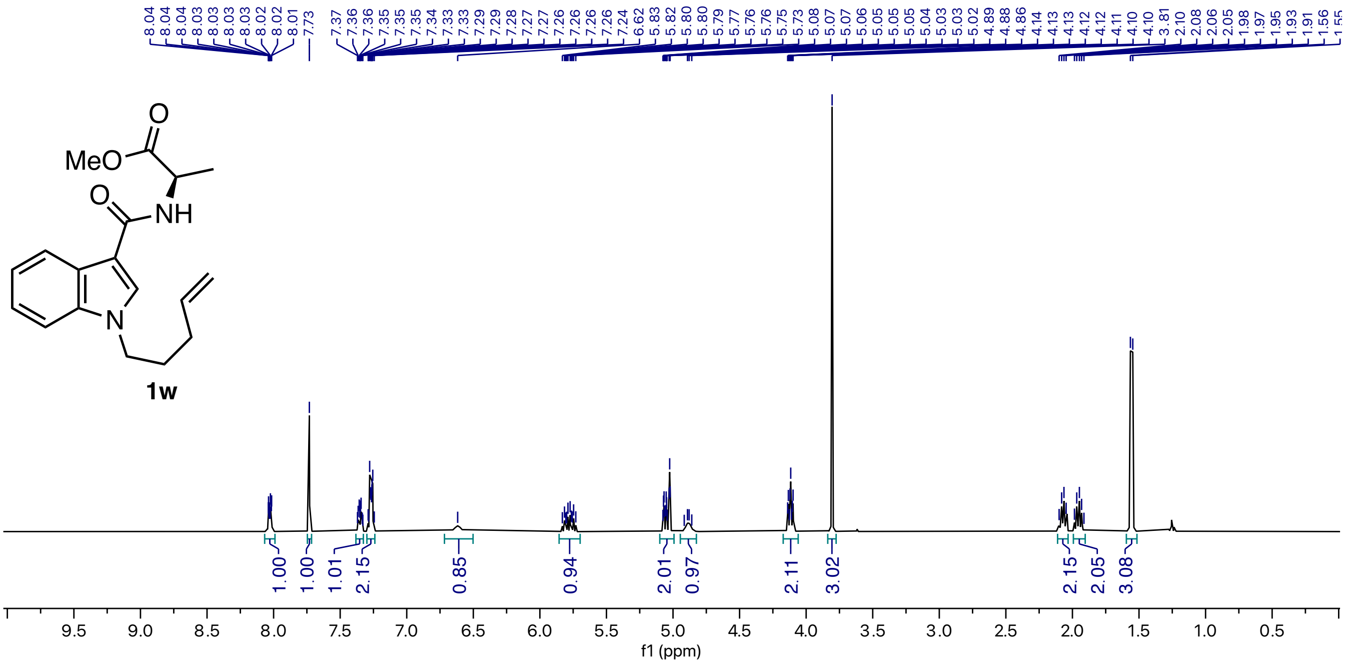

$^{13}C\{^{1}H\}$ **NMR** (100 MHz, $CDCl_3$)

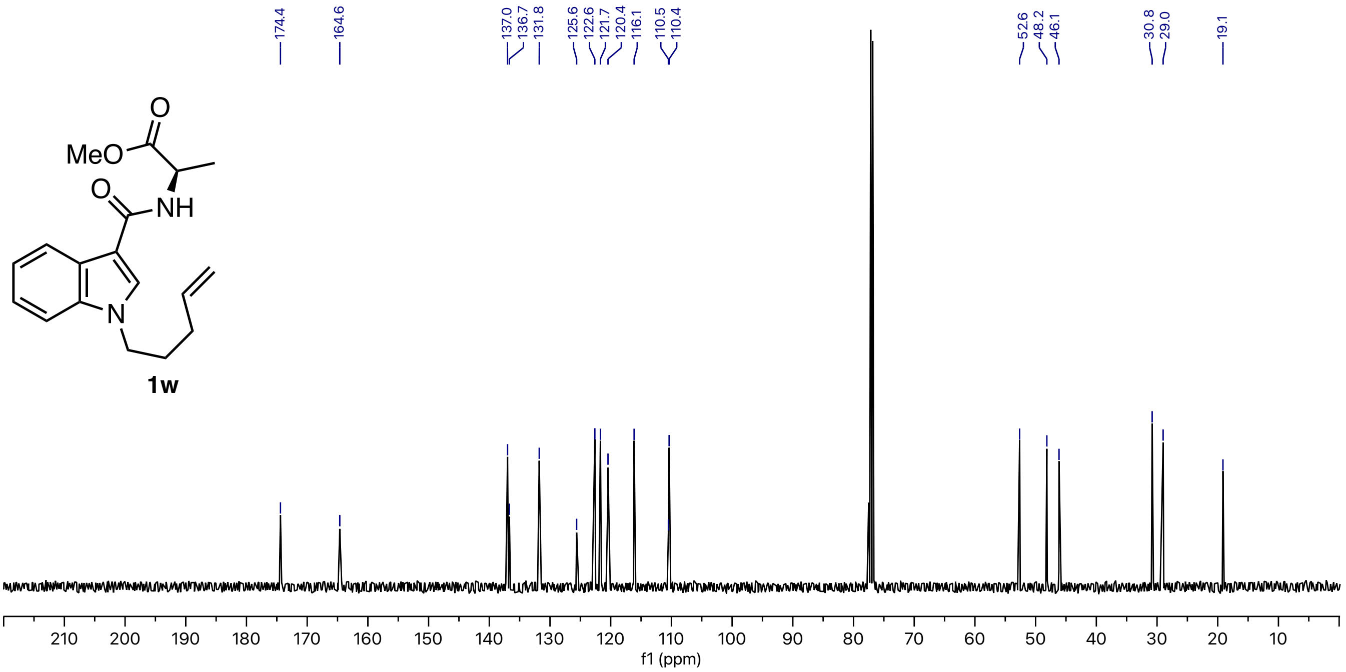

^{1}H NMR (400 MHz, $CDCl_3$)

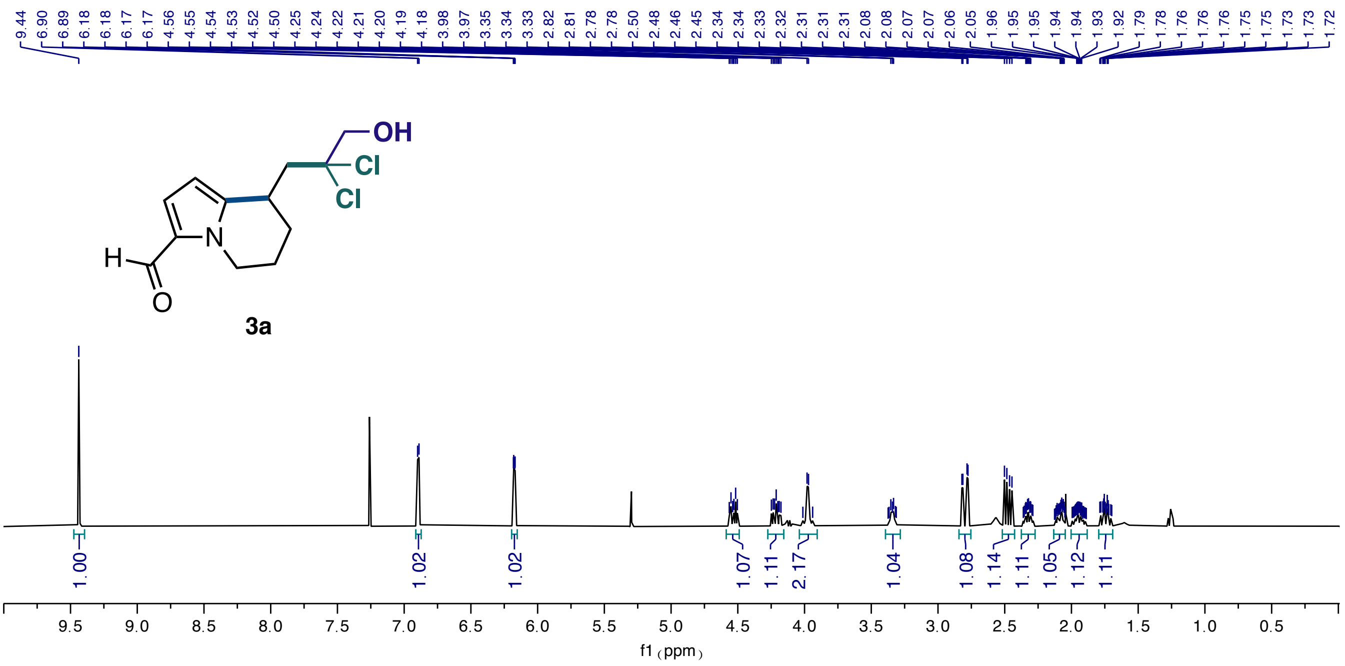

$^{13}C\{^1H\}$ NMR (100 MHz, $CDCl_3$)

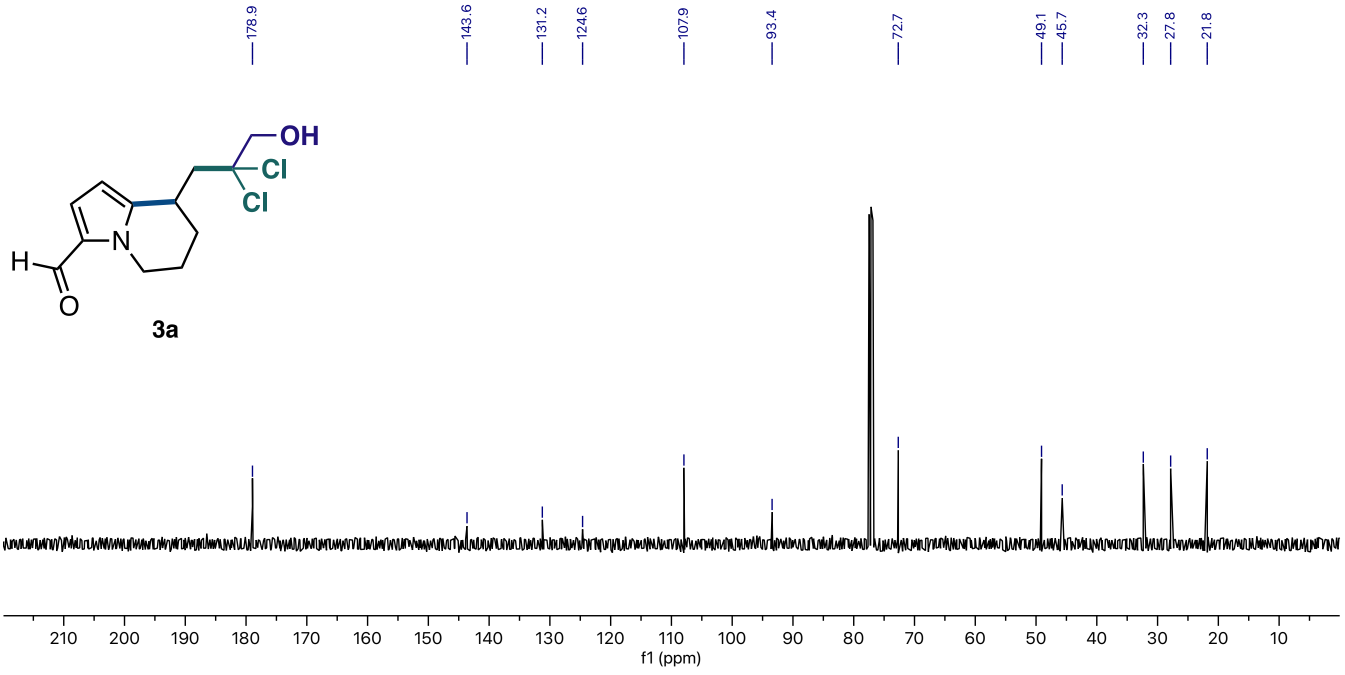

^{1}H NMR (400 MHz, $CDCl_3$)

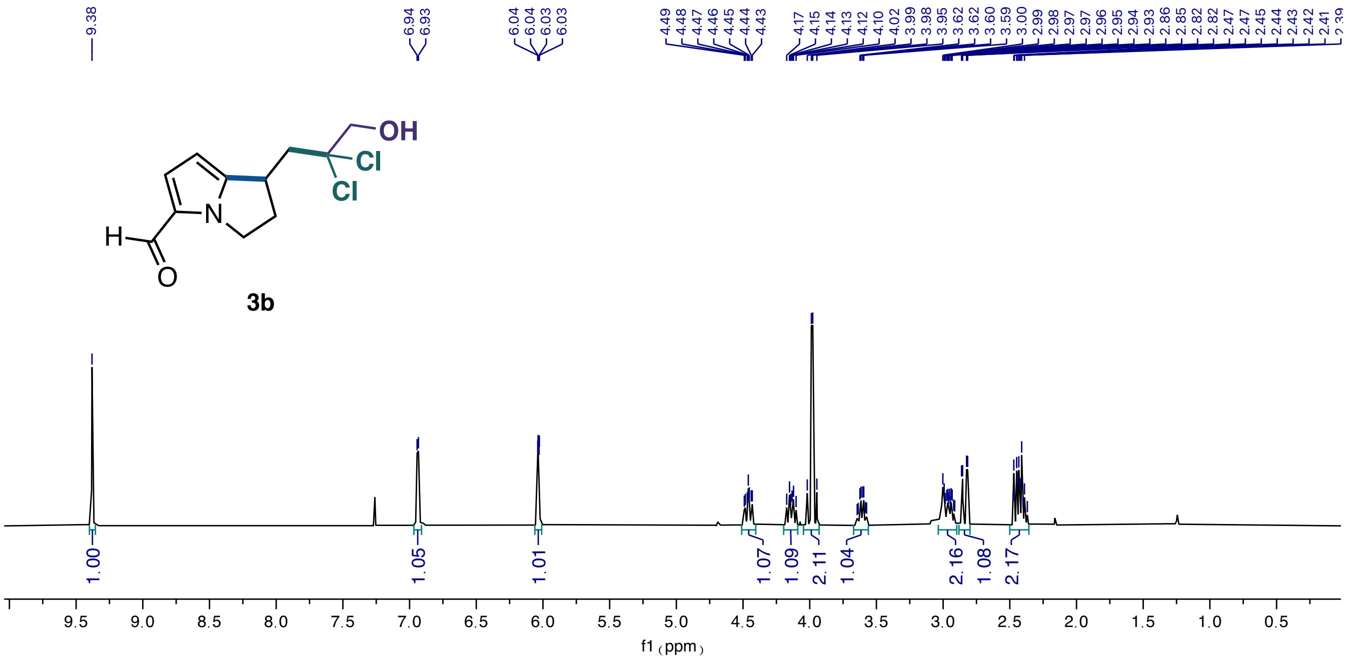

^{13}C{^{1}H} NMR (100 MHz, $CDCl_3$)

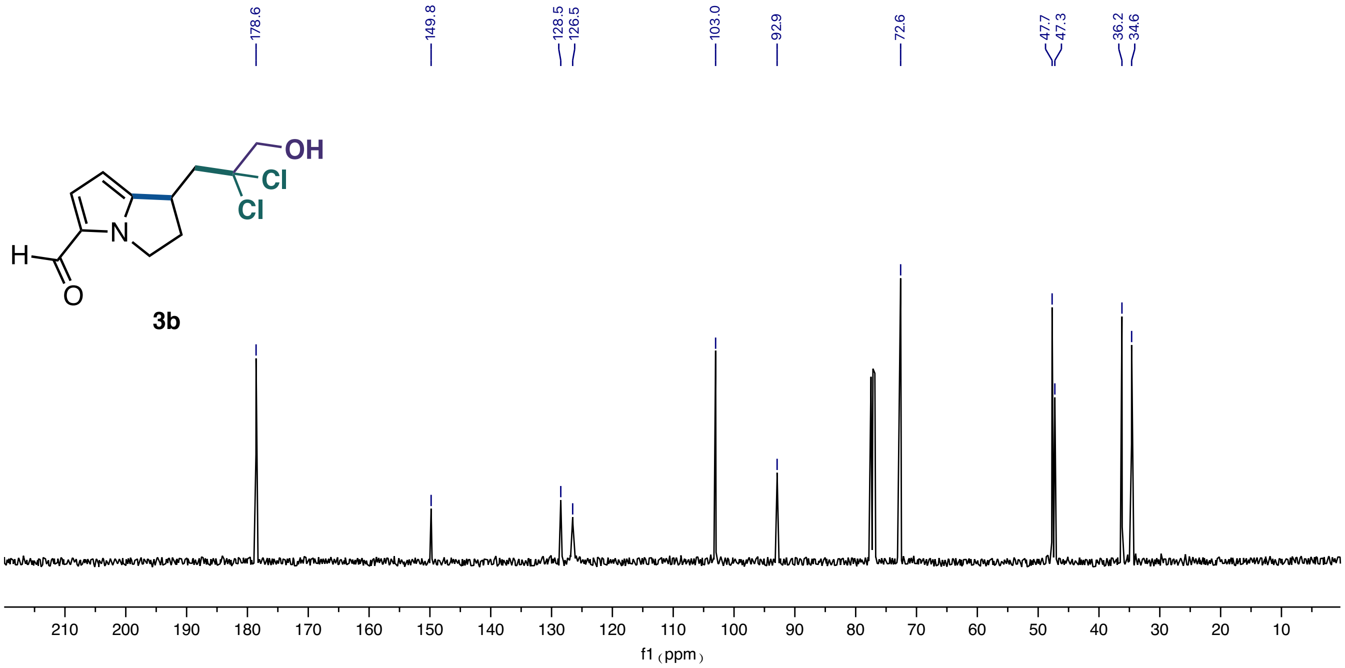

^{1}H NMR (400 MHz, $CDCl_3$)

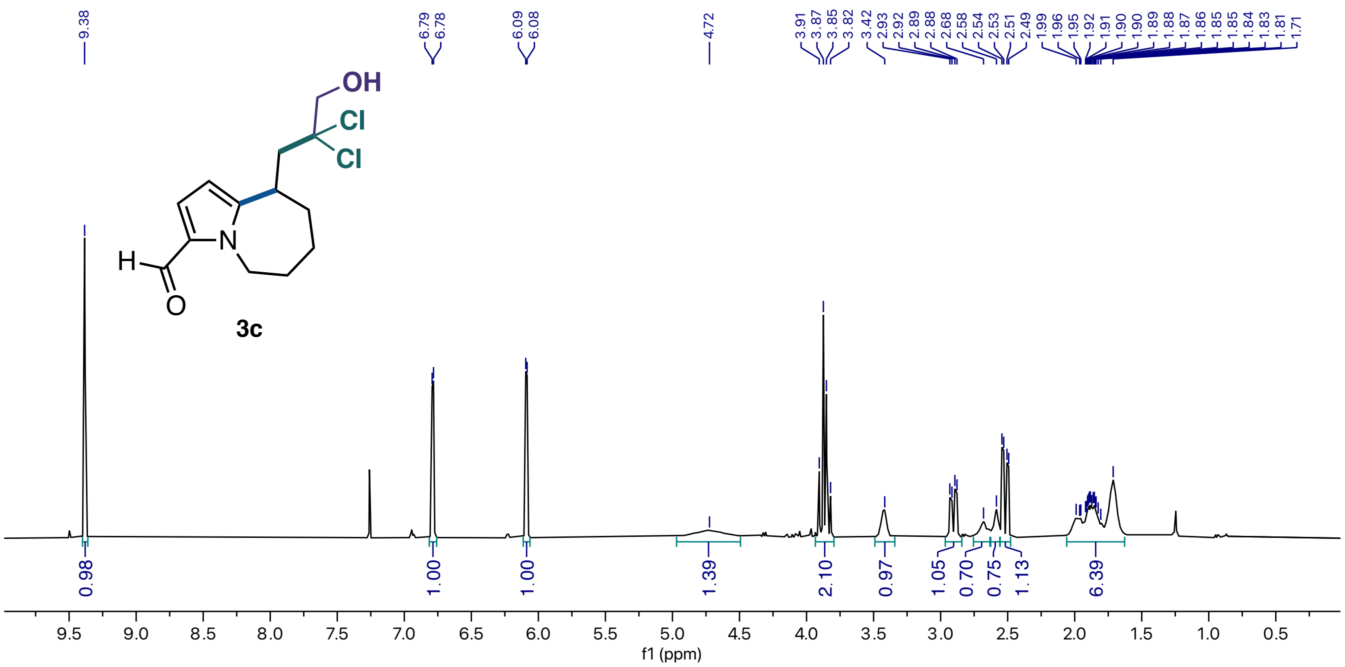

^{13}C{^{1}H} NMR (100 MHz, $CDCl_3$)

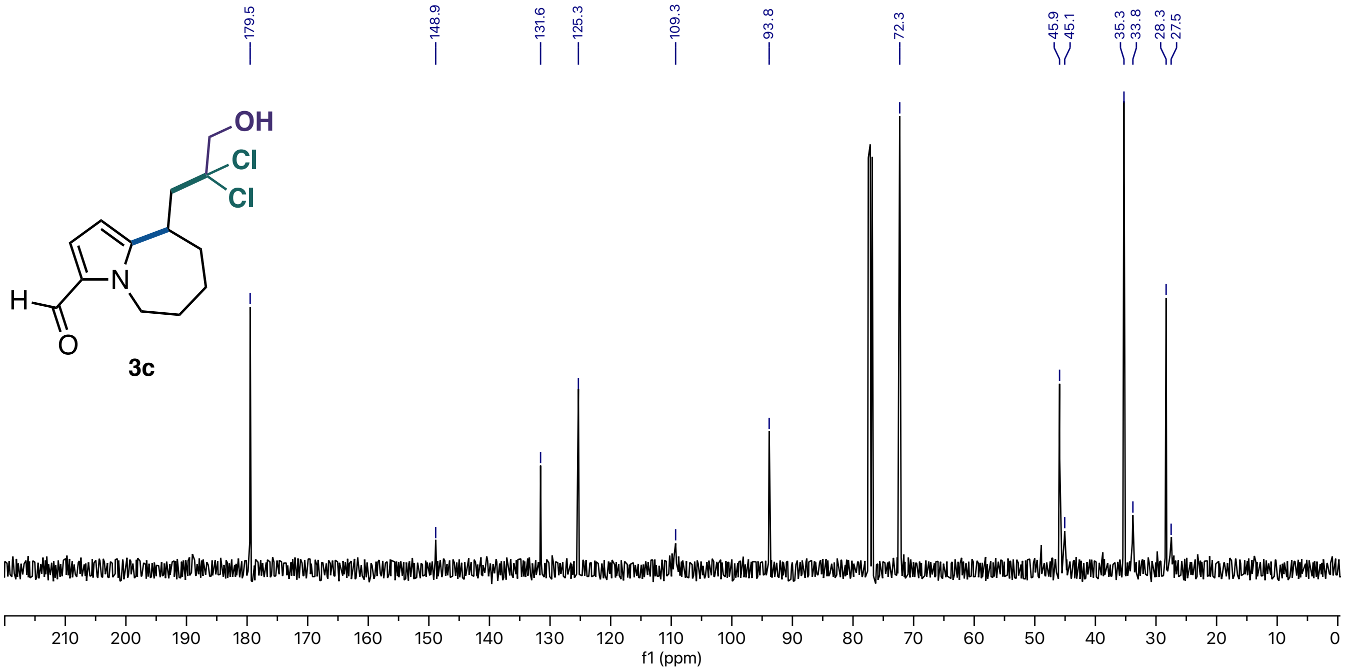

^{1}H NMR (400 MHz, $CDCl_3$)

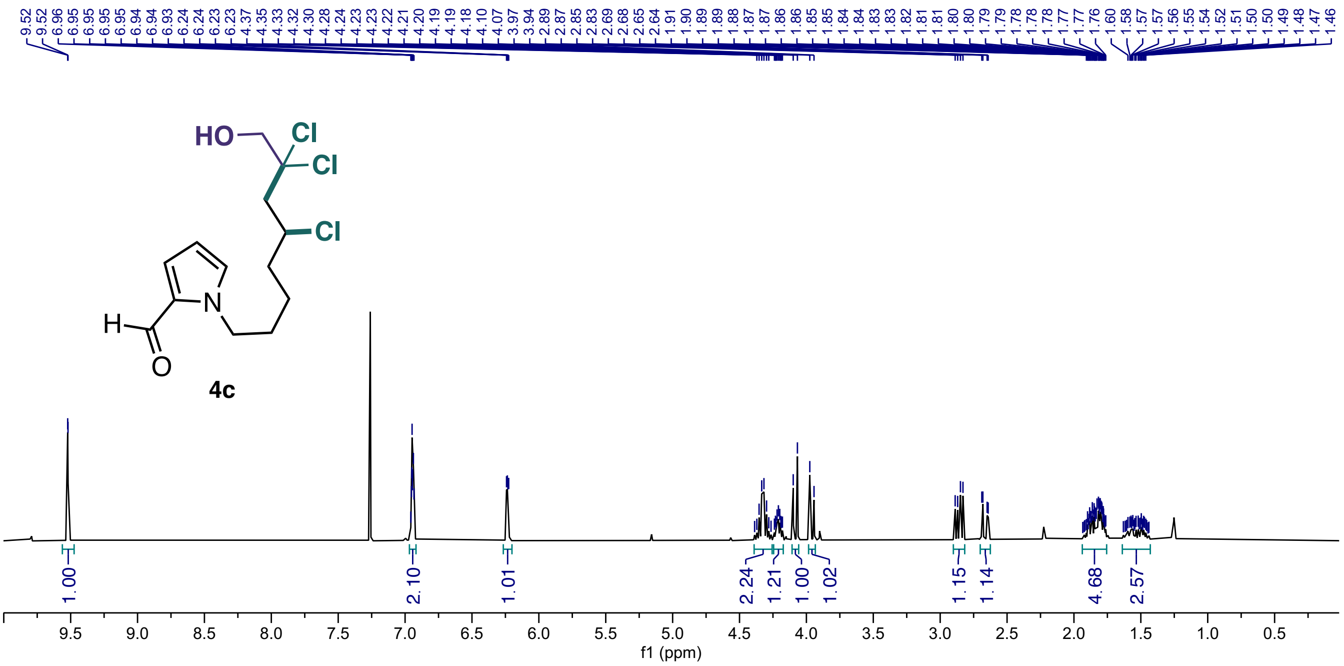

^{13}C{^{1}H} NMR (100 MHz, $CDCl_3$)

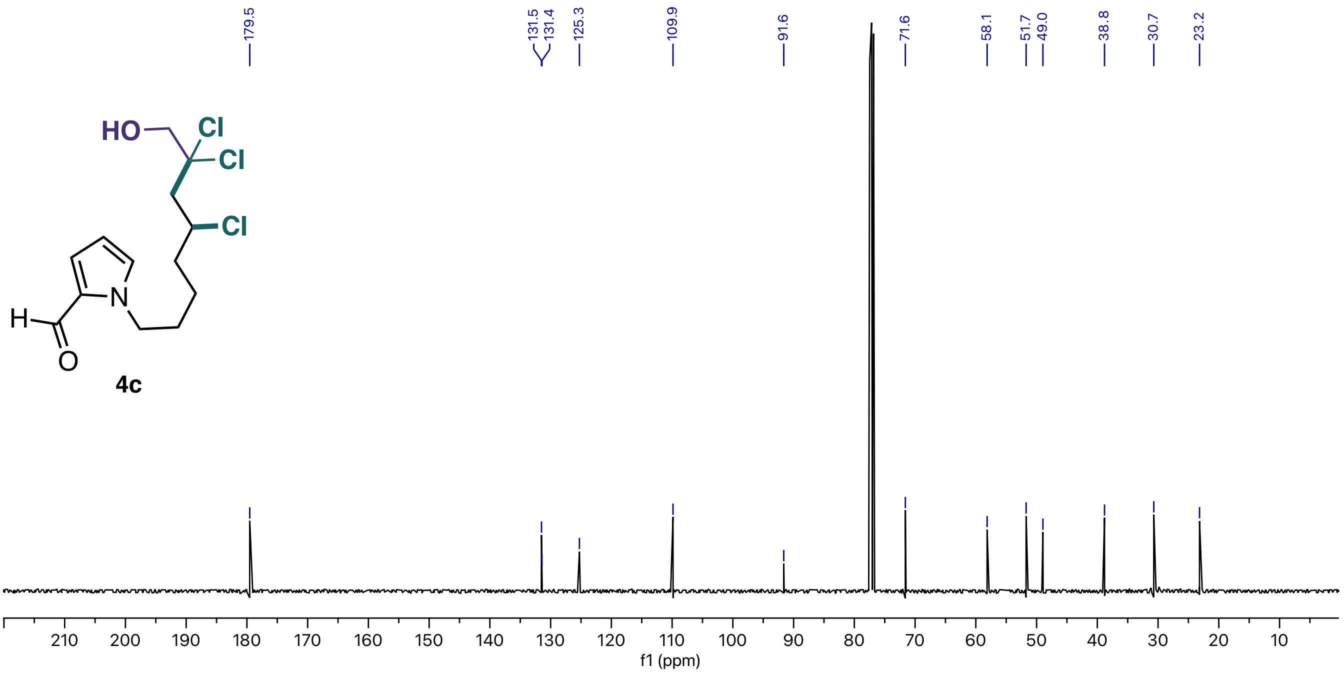

^{1}H NMR (300 MHz, $CDCl_3$)

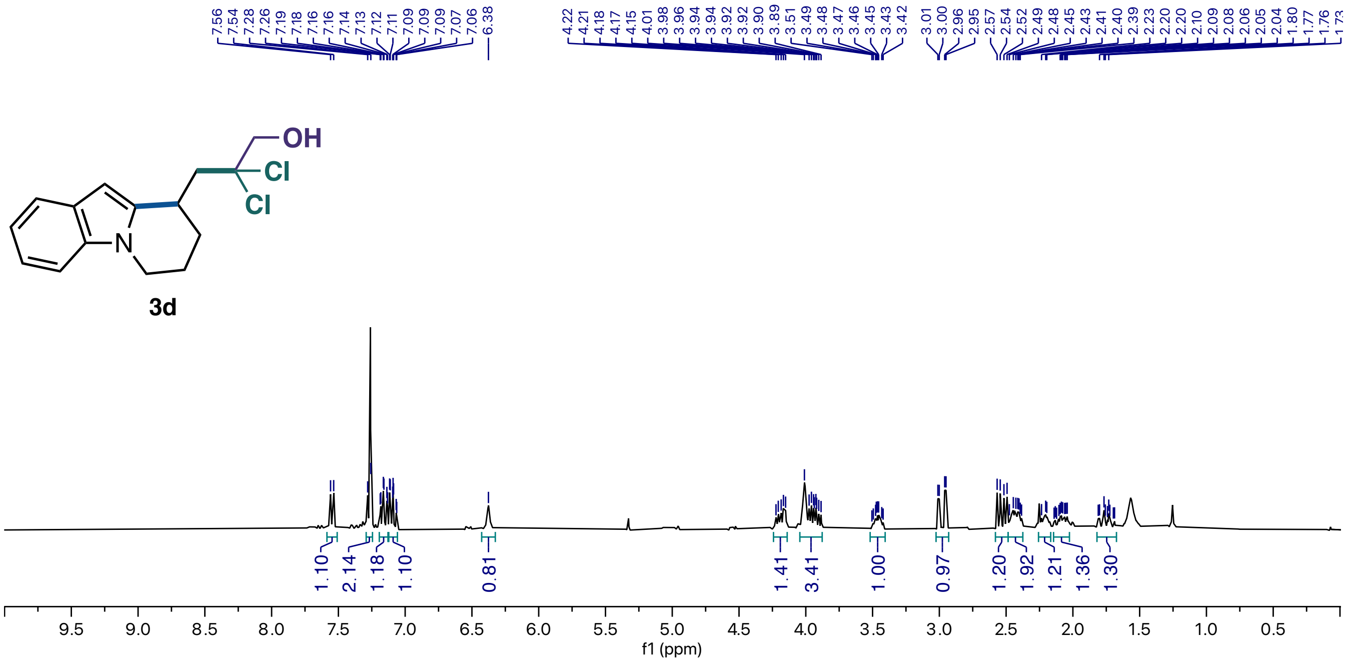

3d

$^{13}C\{^1H\}$ NMR (100 MHz, $CDCl_3$)

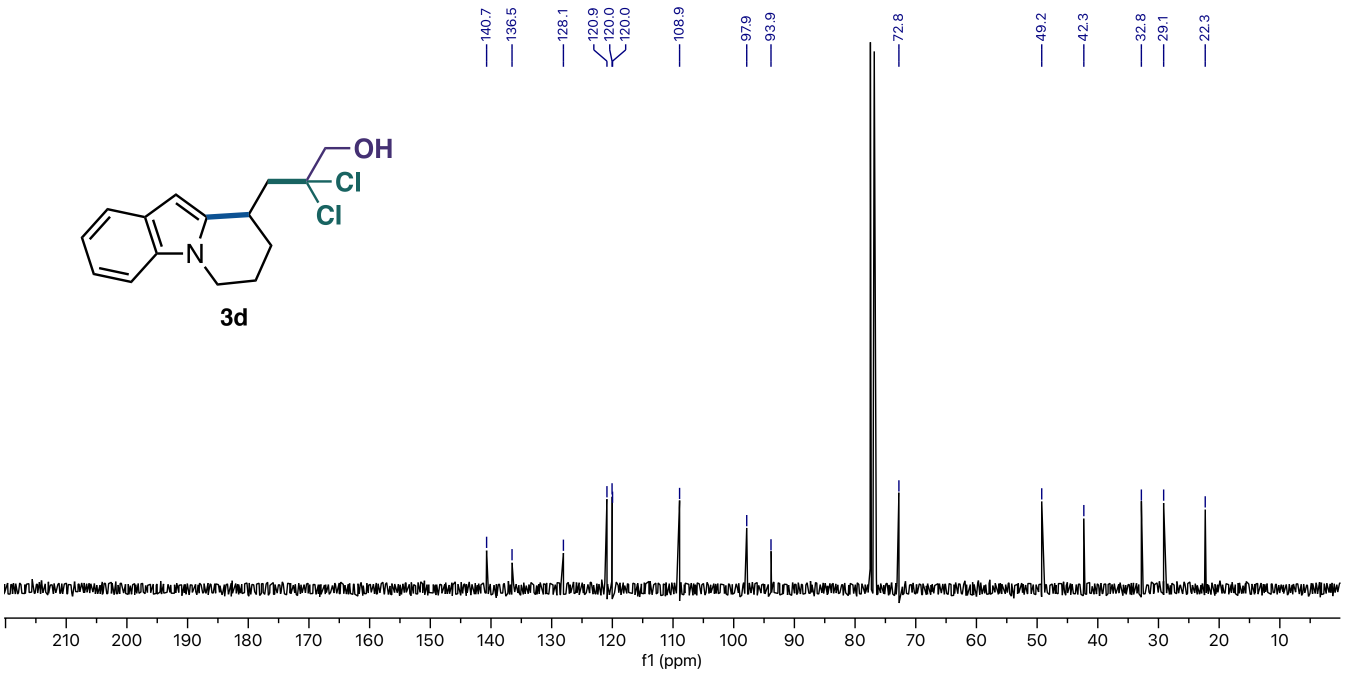

3d

^{1}H NMR (700 MHz, $CDCl_3$)

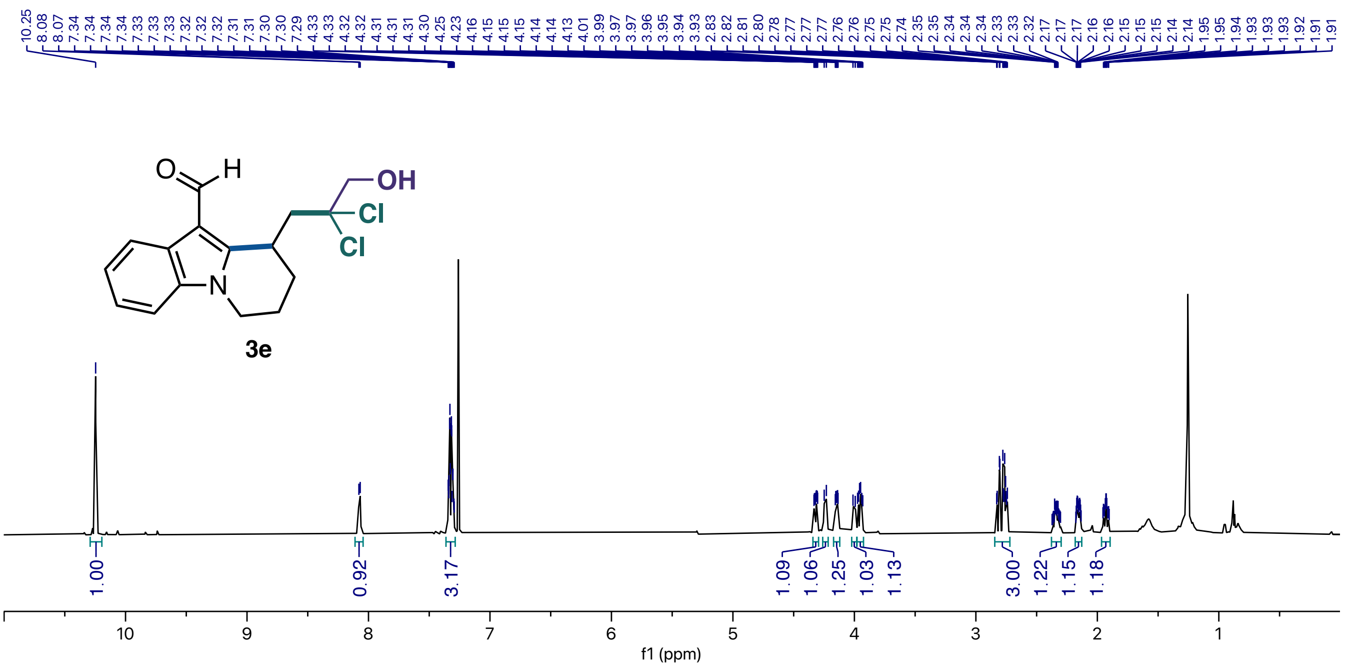

^{13}C{^{1}H} NMR (175 MHz, $CDCl_3$)

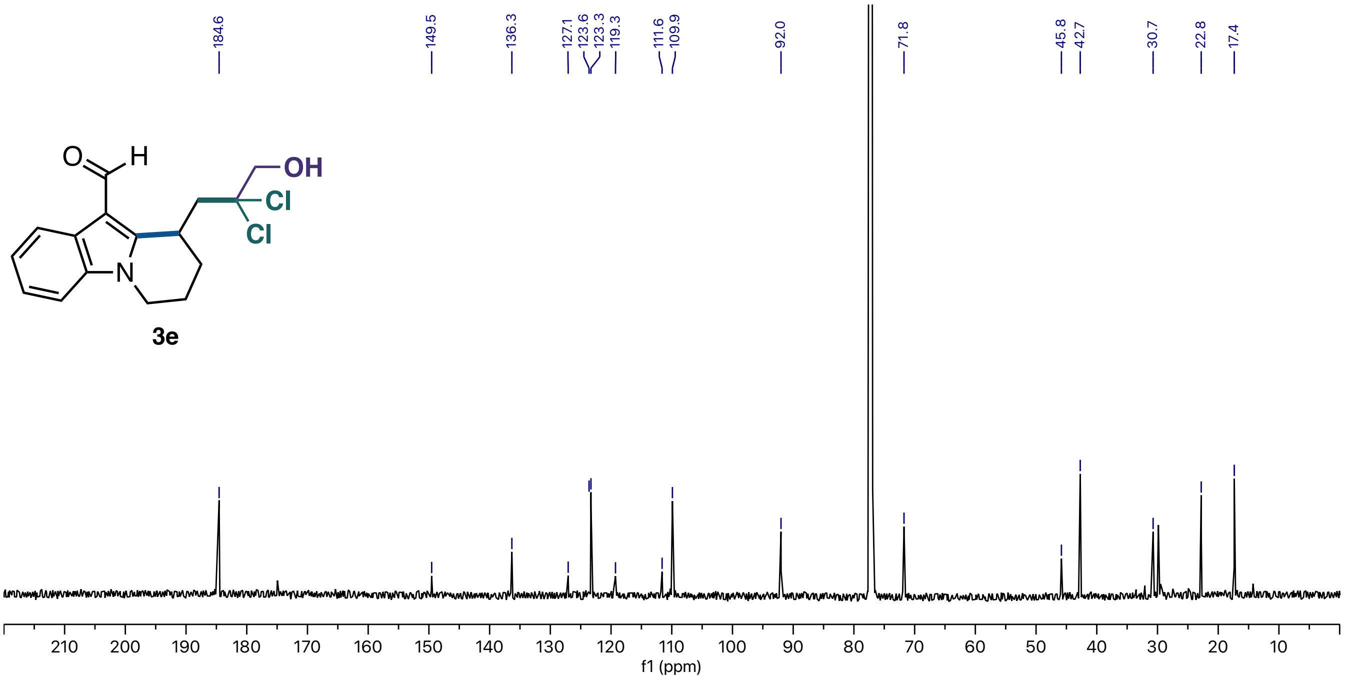

^{1}H NMR (400 MHz, $CDCl_3$)

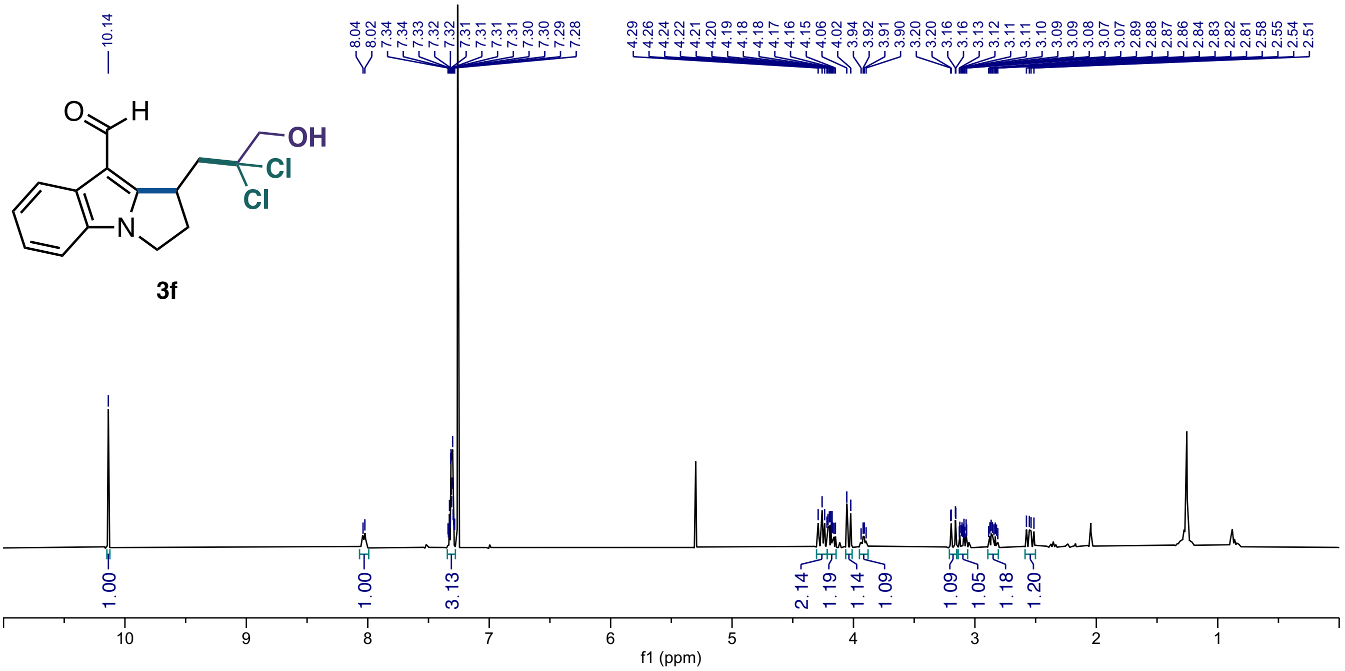

^{13}C{^{1}H} NMR (100 MHz, $CDCl_3$)

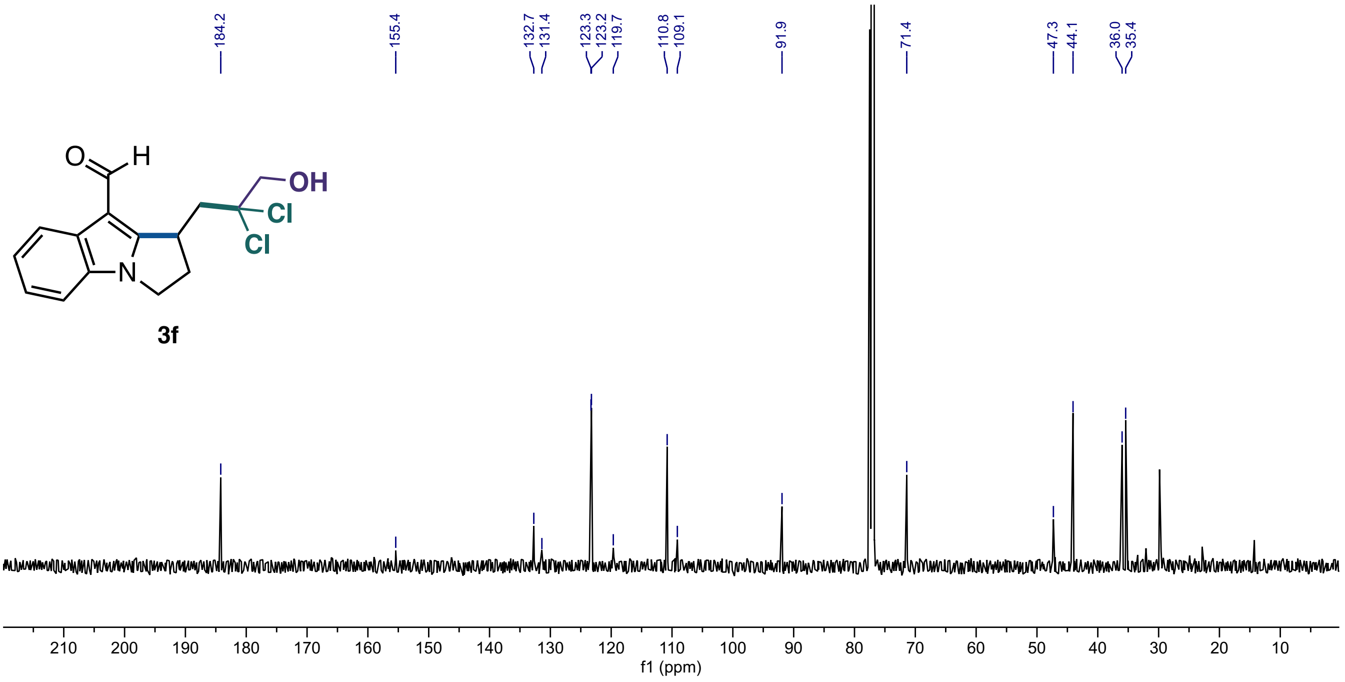

^{1}H NMR (400 MHz, $CDCl_3$)

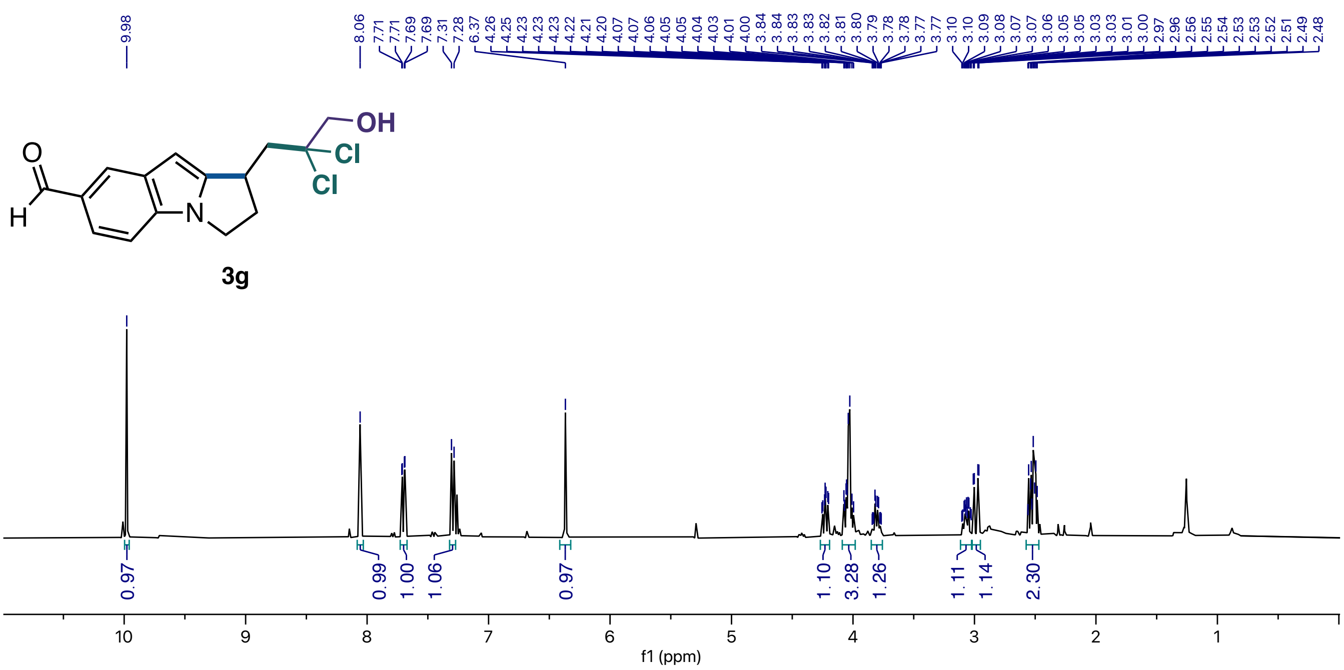

^{13}C{^{1}H} NMR (100 MHz, $CDCl_3$)

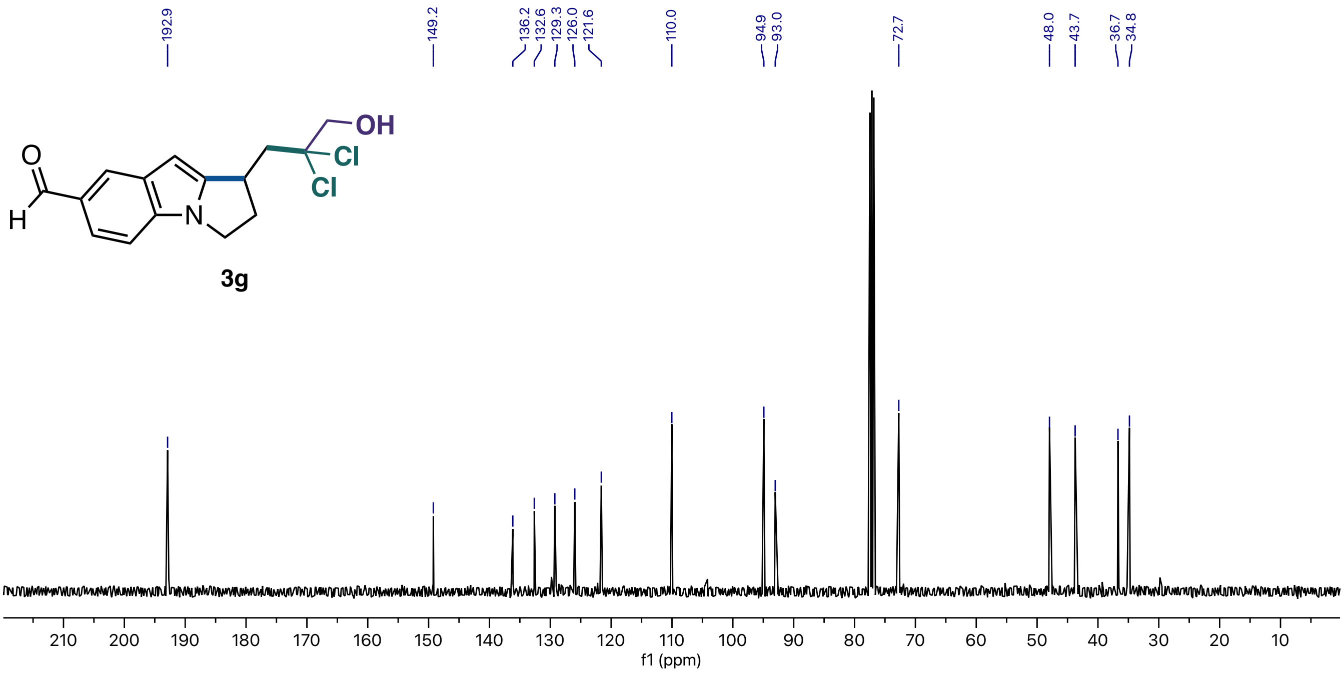

^{1}H NMR (400 MHz, $CDCl_3$)

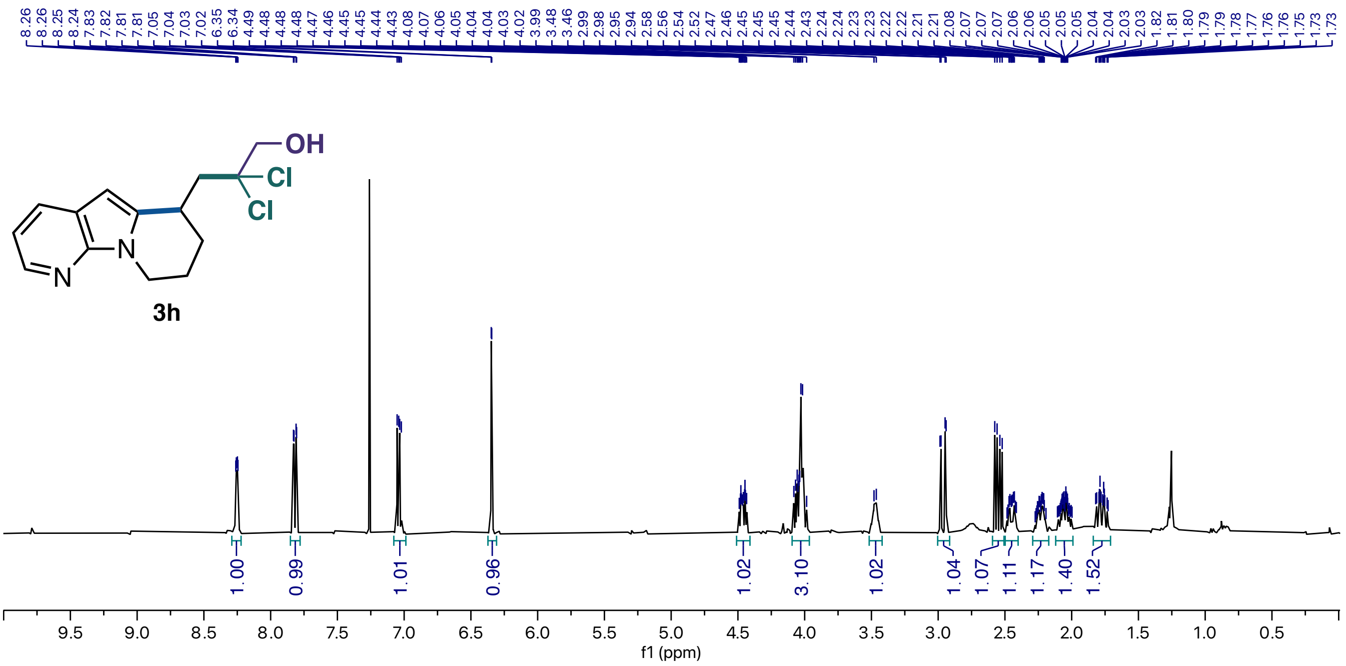

^{13}C{^{1}H} NMR (100 MHz, $CDCl_3$)

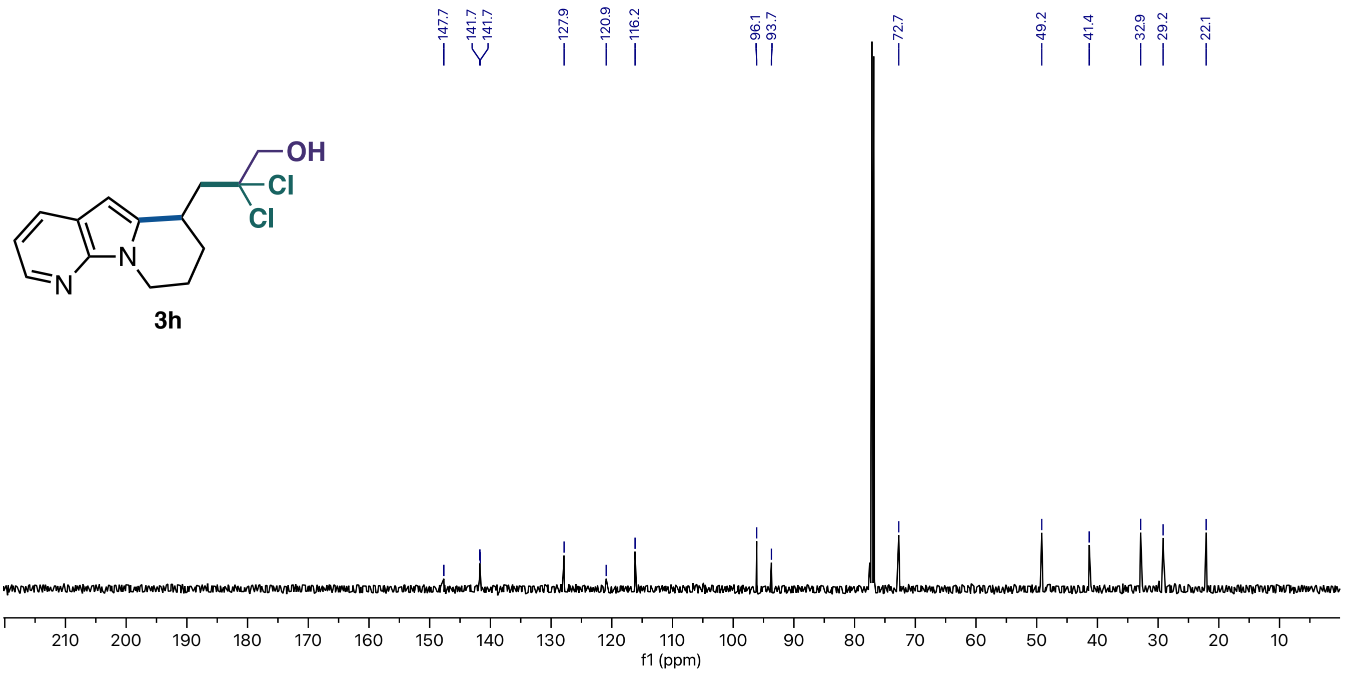

^{1}H NMR (400 MHz, $CDCl_3$)

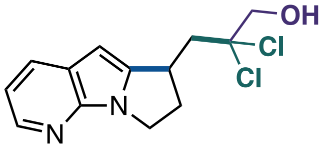

3i

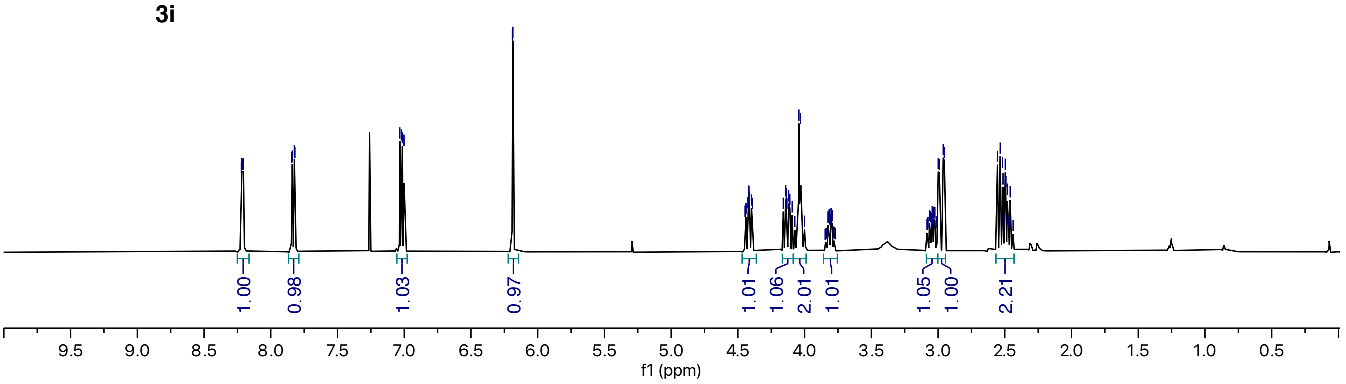

^{13}C{^{1}H} NMR (100 MHz, $CDCl_3$)

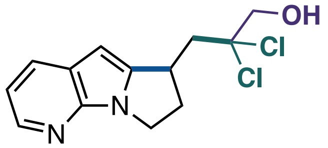

3i

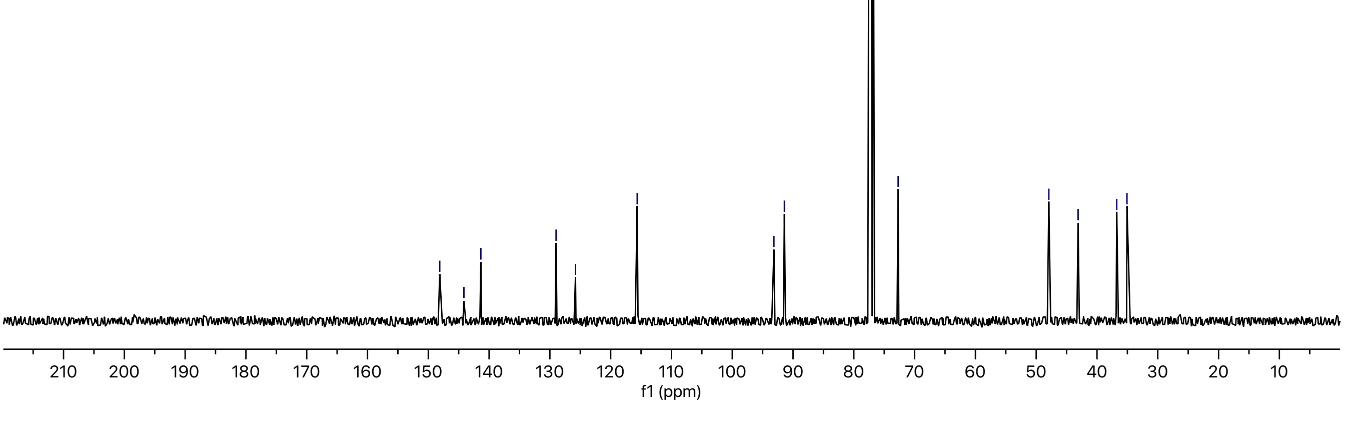

^{1}H NMR (400 MHz, $CDCl_3$)

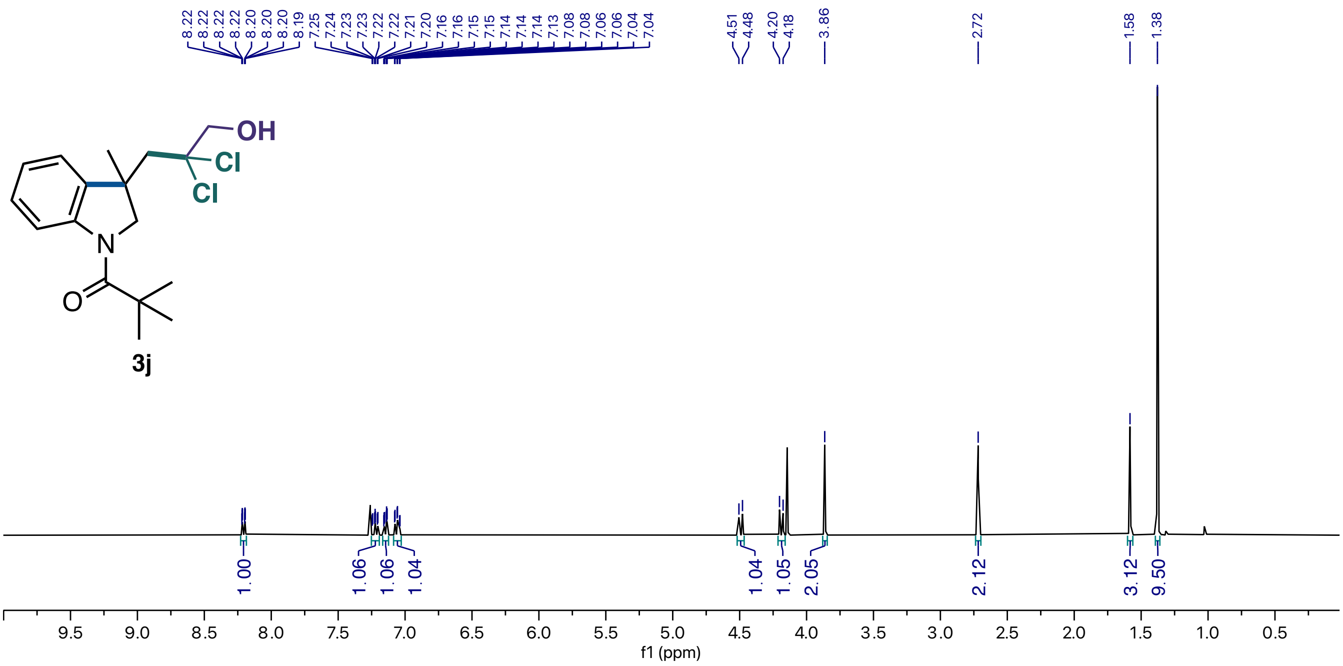

^{13}C{^{1}H} NMR (100 MHz, $CDCl_3$)

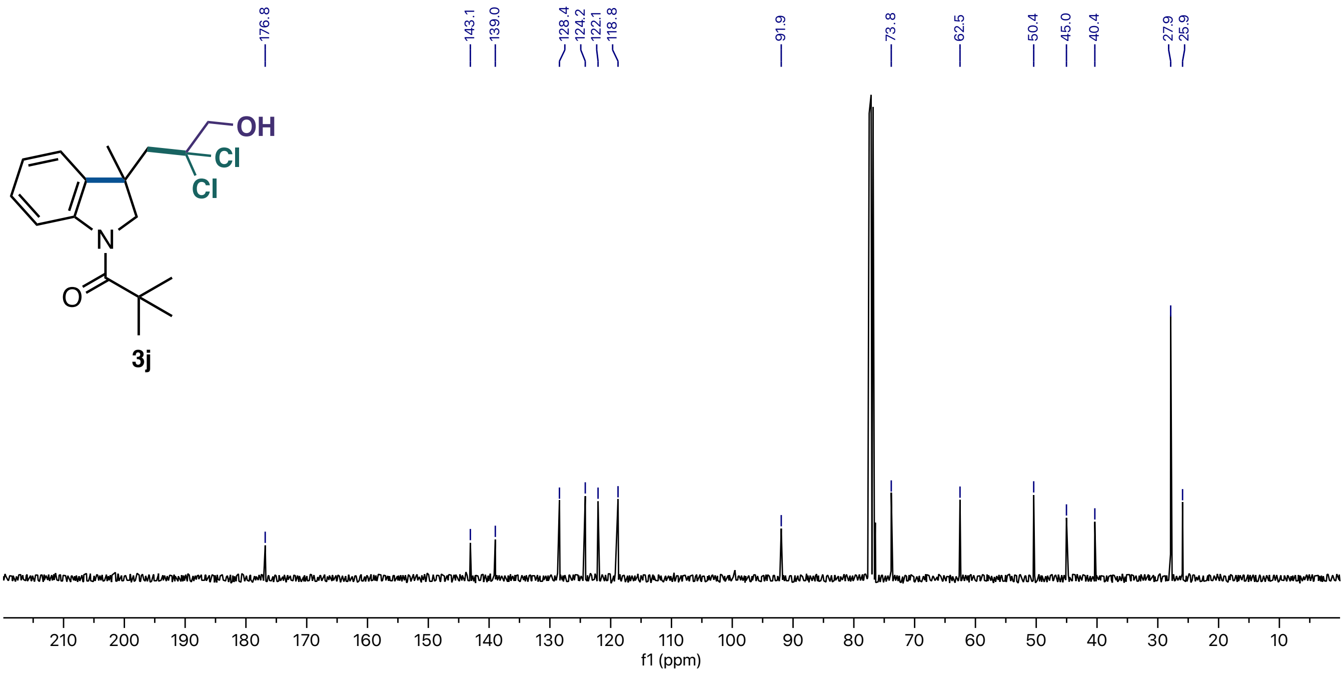

^{1}H NMR (400 MHz, $CDCl_3$)

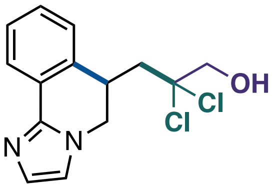

3k

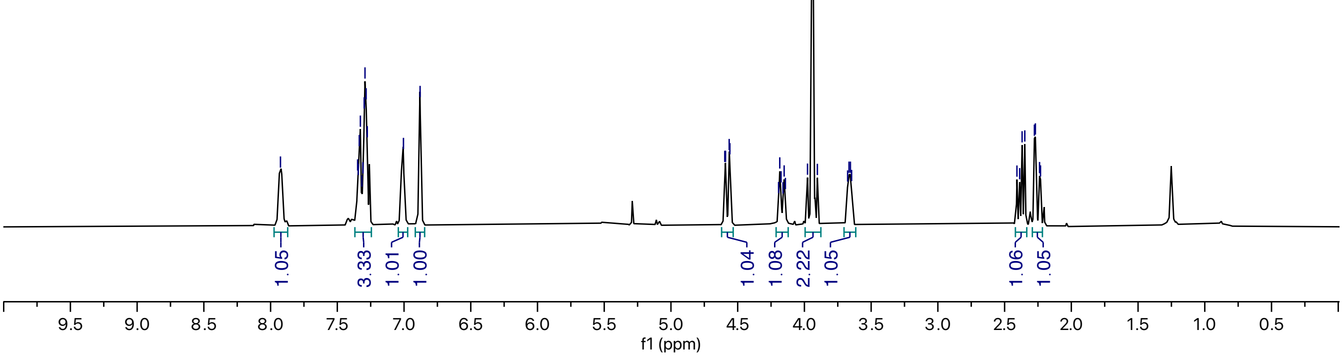

$^{13}C\{^1H\}$ NMR (100 MHz, $CDCl_3$)

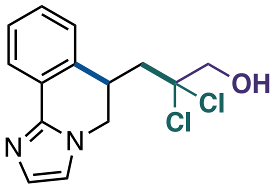

3k

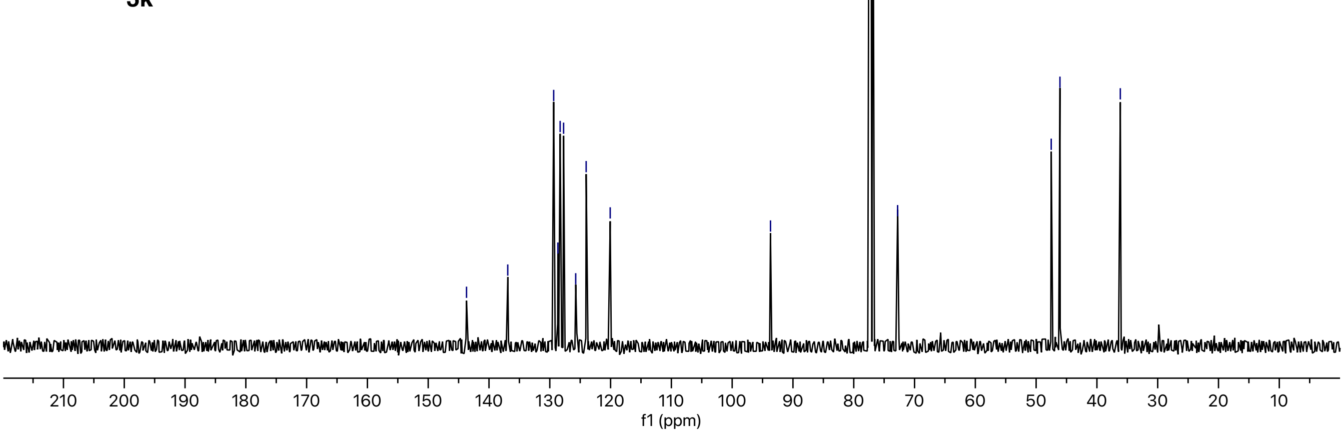

^{1}H NMR (400 MHz, $CDCl_3$)

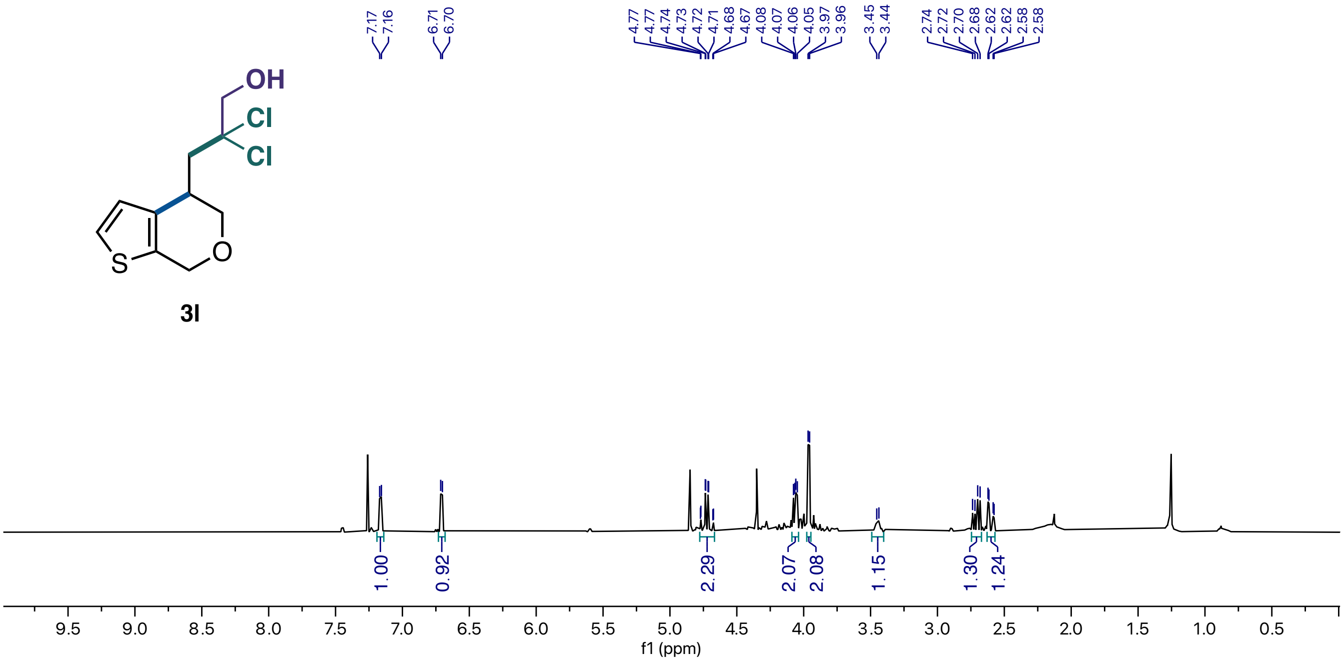

$^{13}C\{^{1}H\}$ NMR (100 MHz, $CDCl_3$)

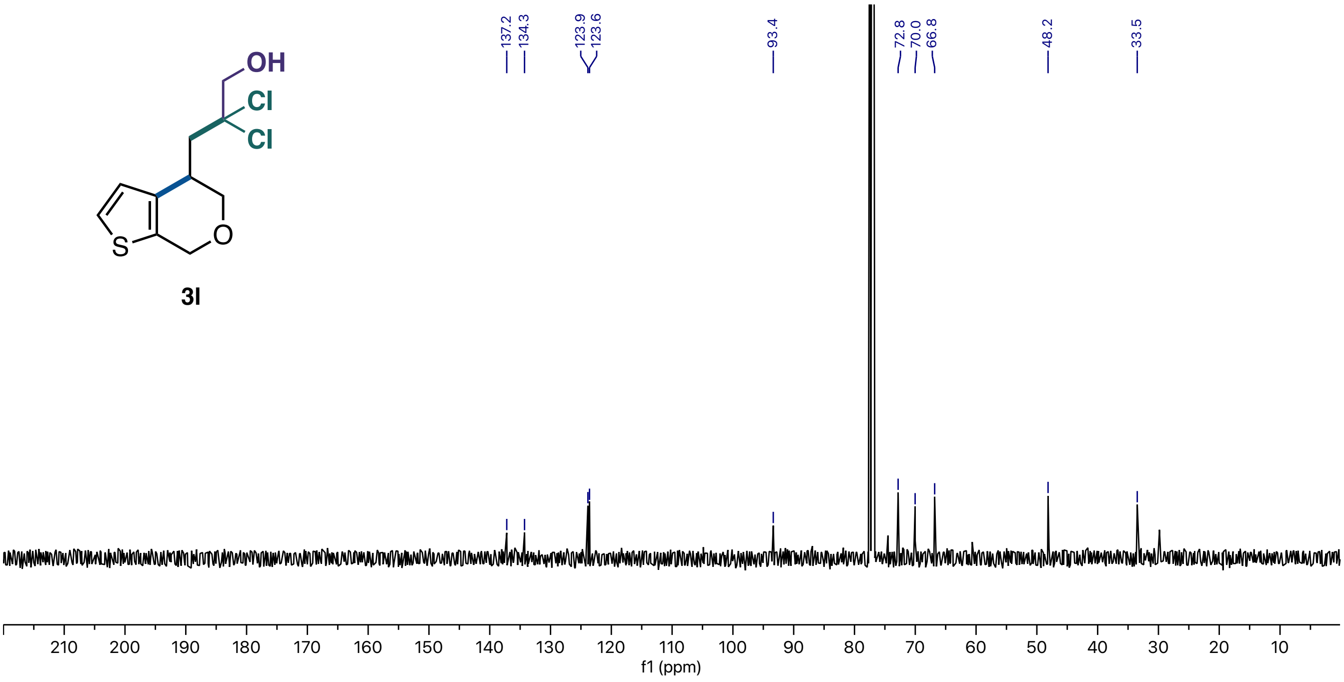

¹H NMR (300 MHz, $CDCl_3$)

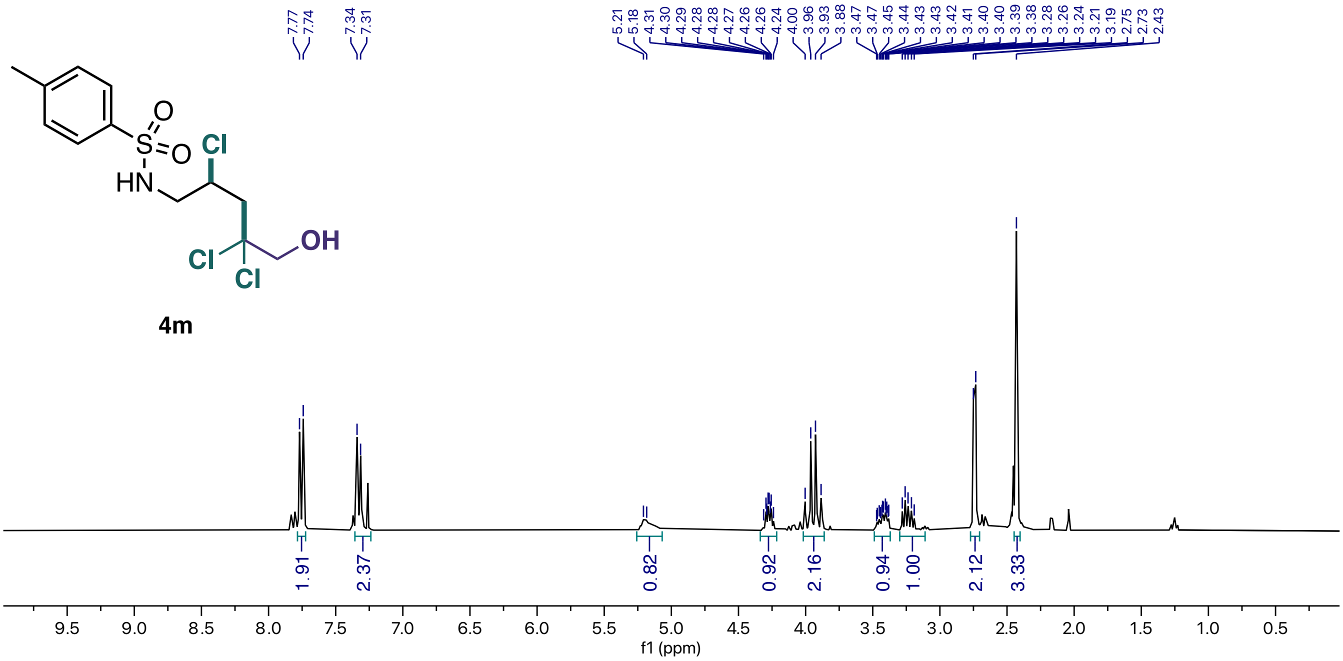

¹³C{¹H} NMR (75 MHz, $CDCl_3$)

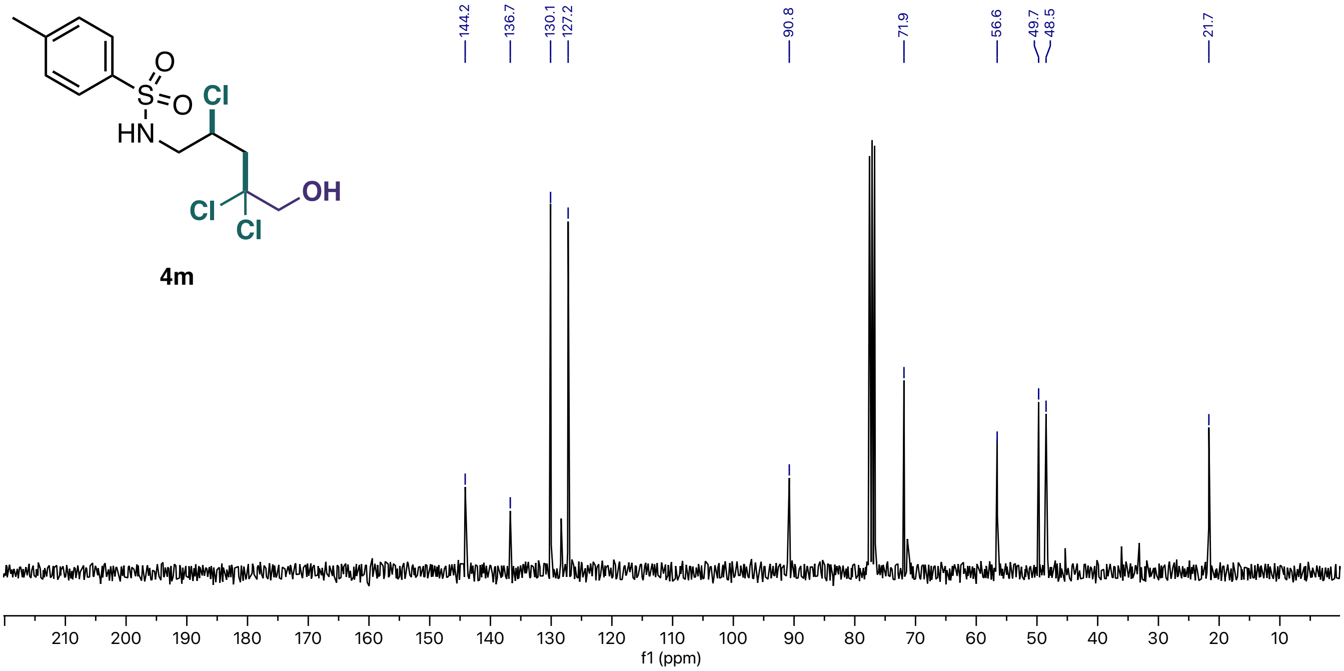

^{1}H NMR (400 MHz, $CDCl_3$)

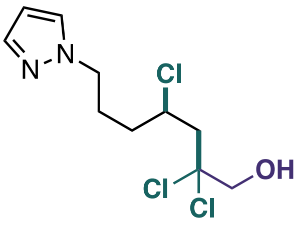

4n

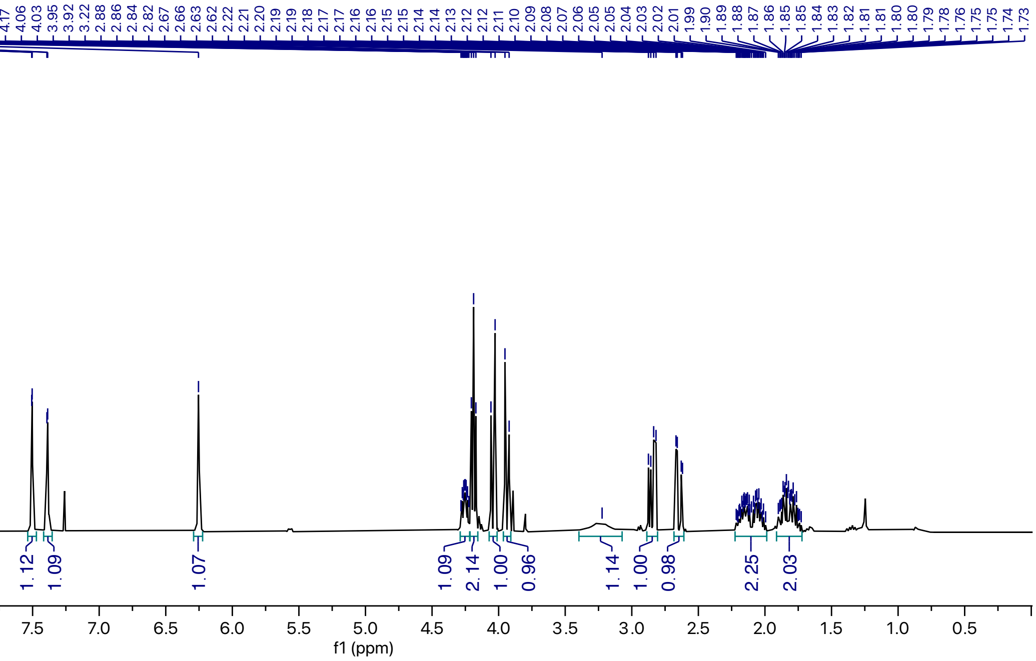

$^{13}C\{^{1}H\}$ NMR (100 MHz, $CDCl_3$)

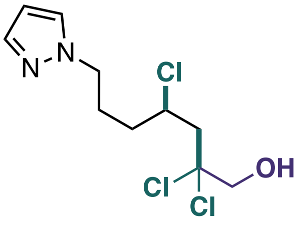

4n

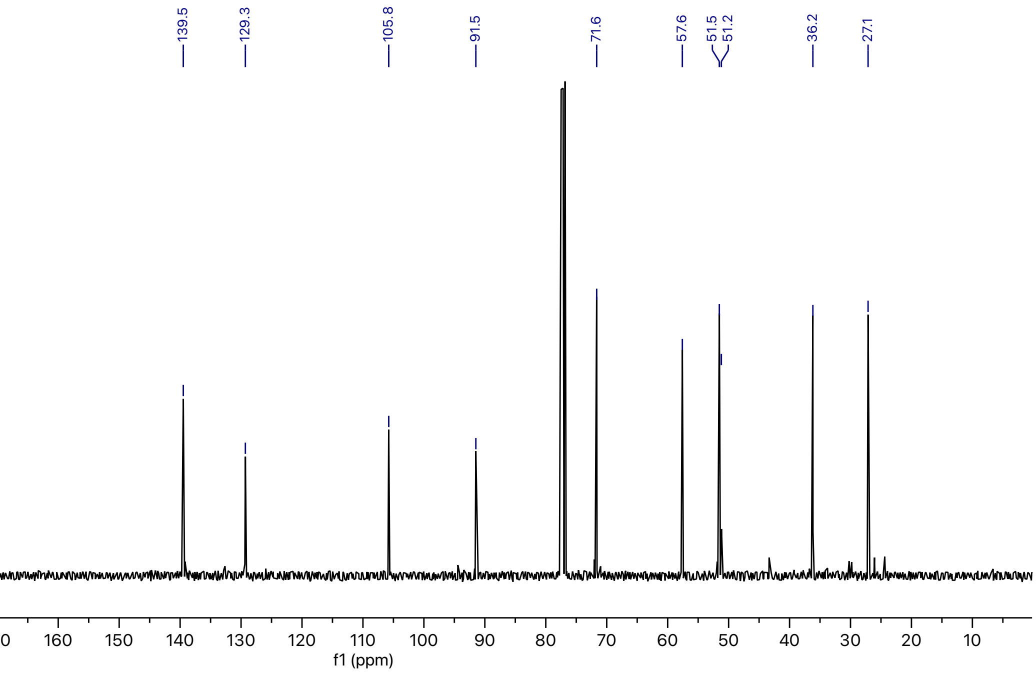

^{1}H NMR (400 MHz, $CDCl_3$)

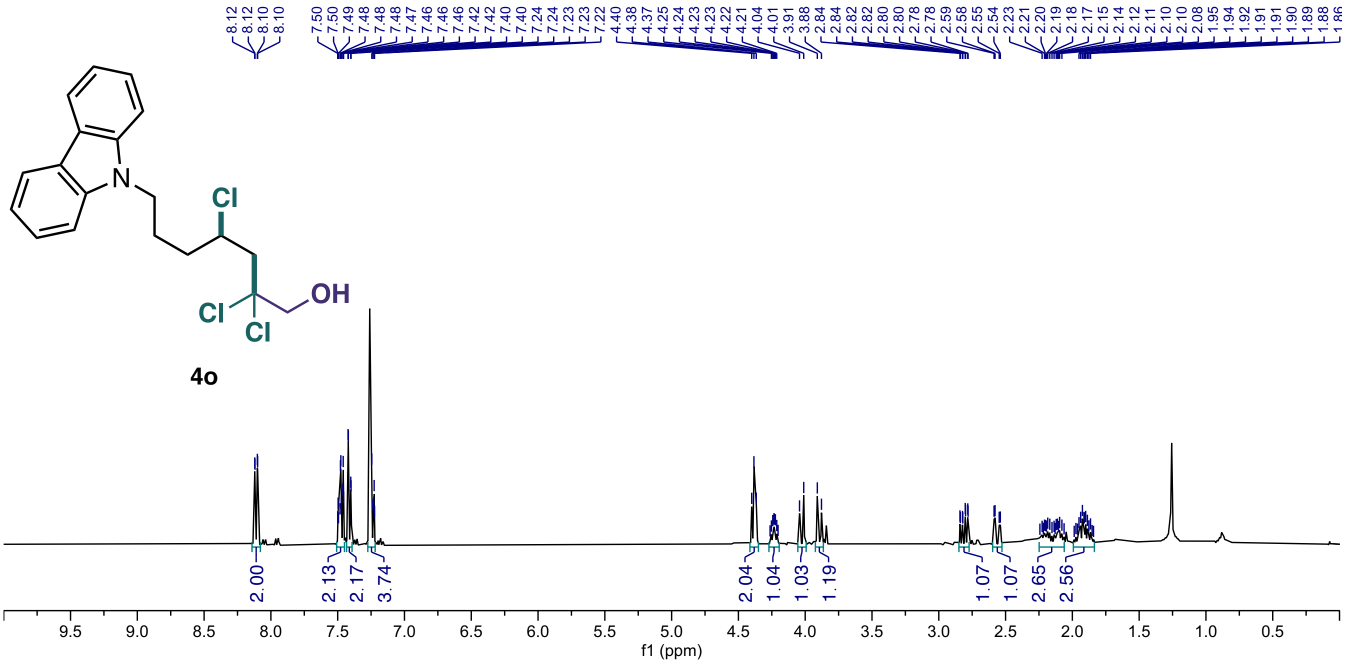

^{13}C{^{1}H} NMR (100 MHz, $CDCl_3$)

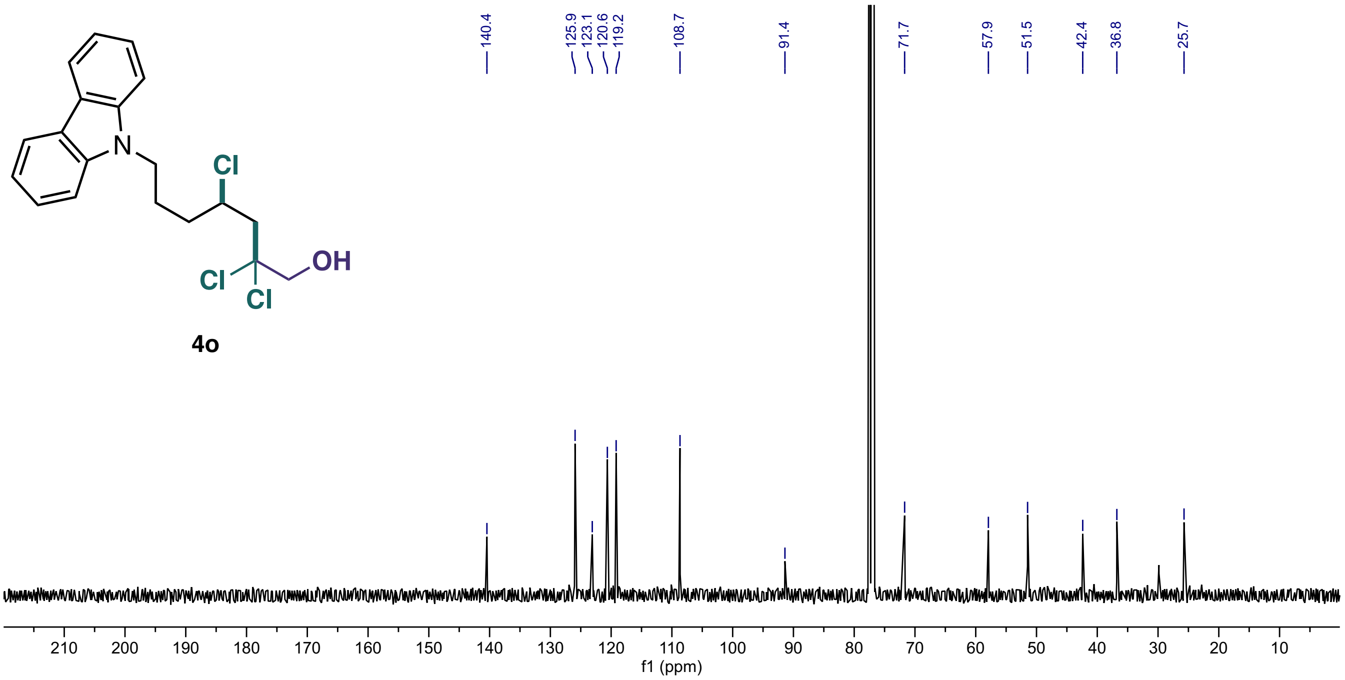

^{1}H NMR (400 MHz, $CDCl_3$)

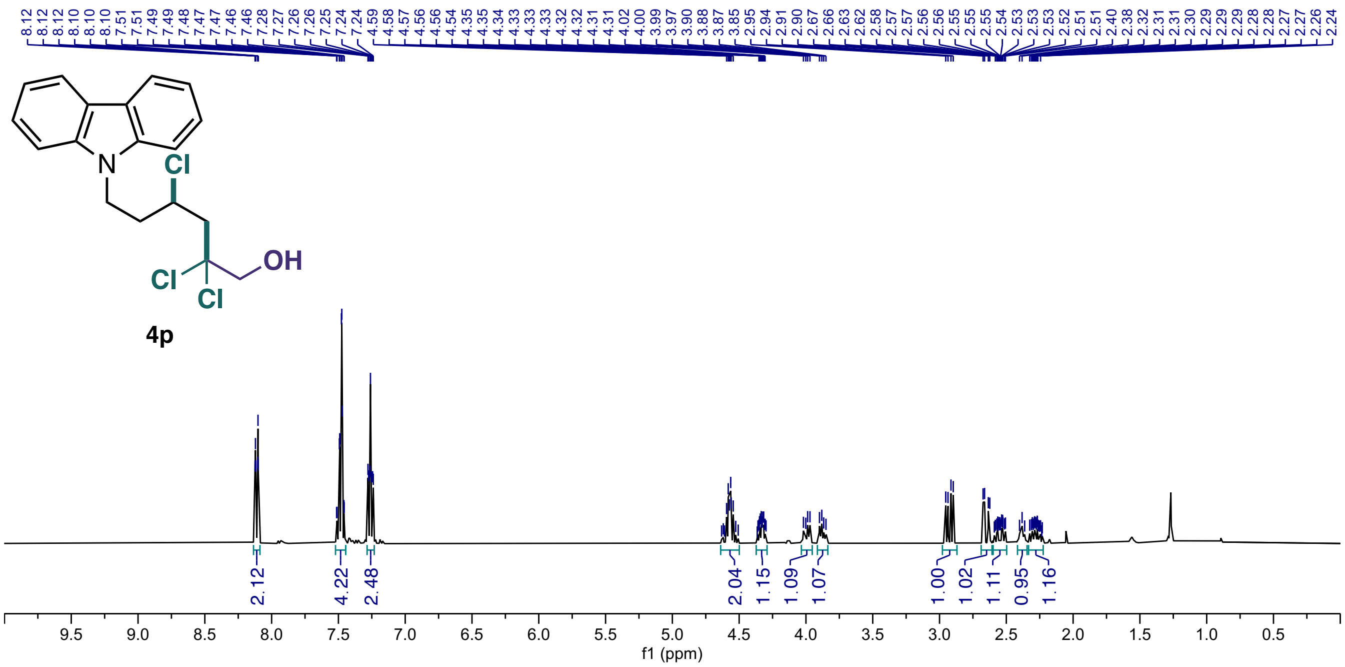

^{13}C{^{1}H} NMR (100 MHz, $CDCl_3$)

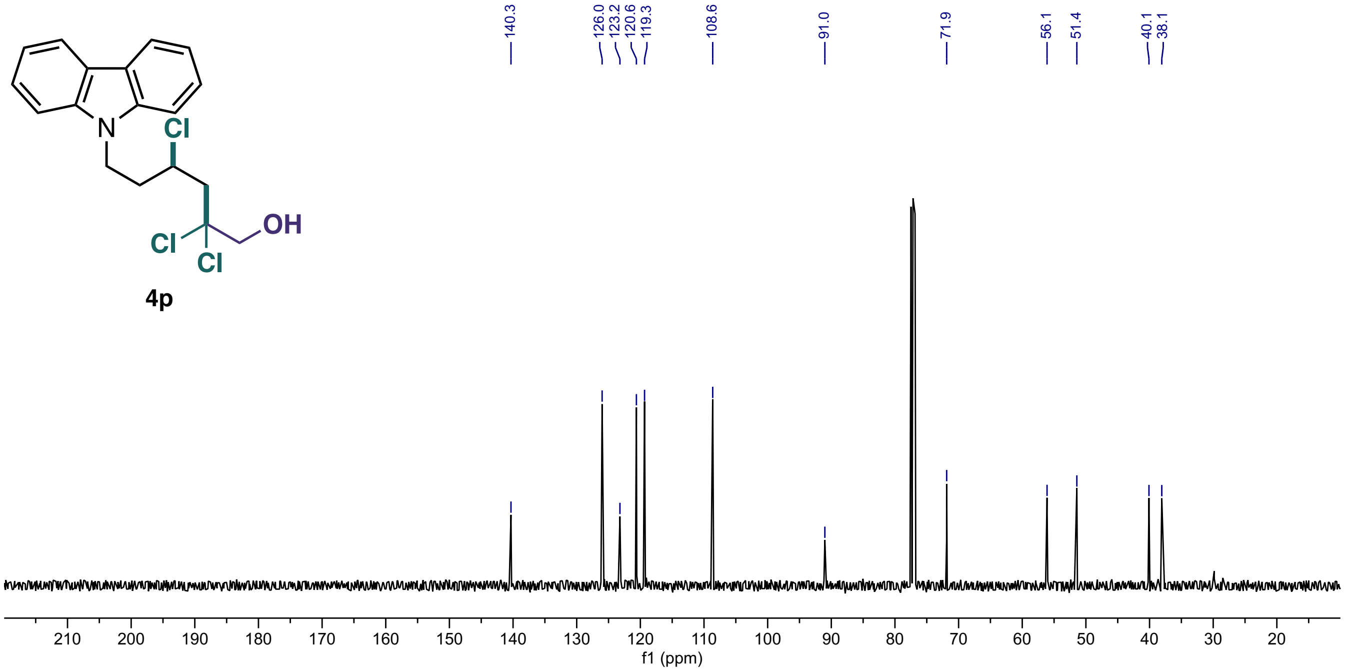

^{1}H NMR (400 MHz, $CDCl_3$)

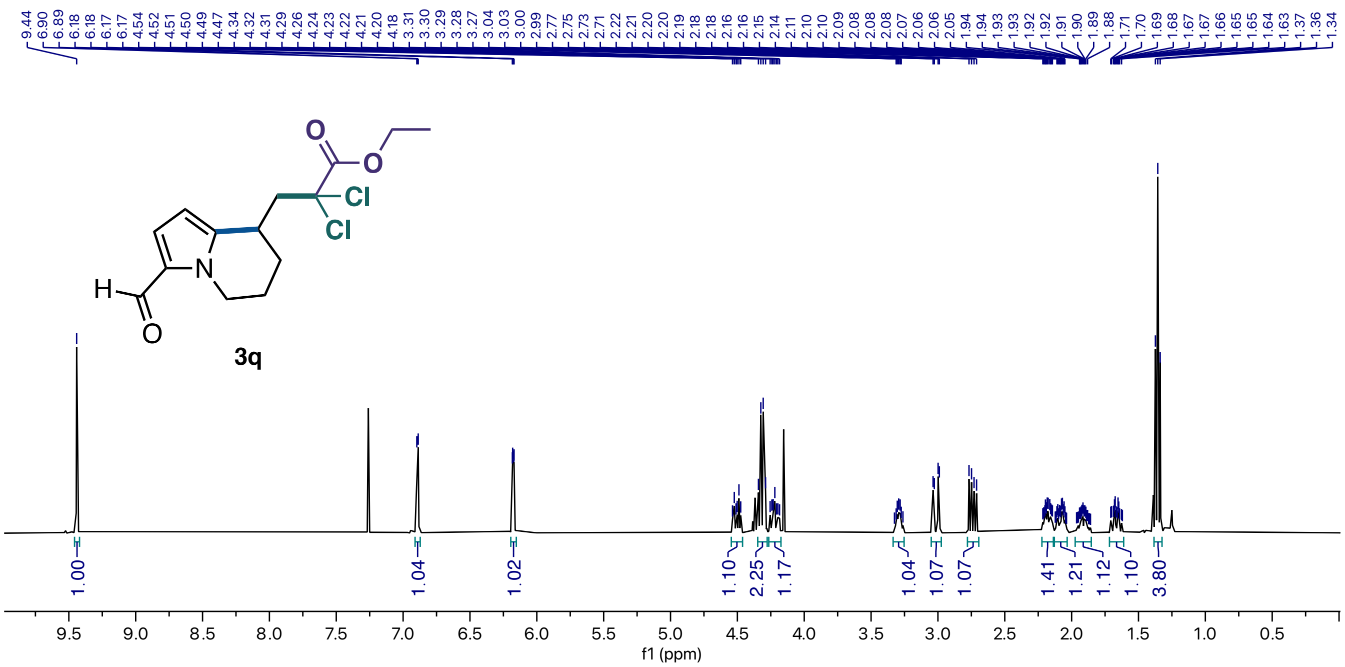

^{13}C{^{1}H} NMR (100 MHz, $CDCl_3$)

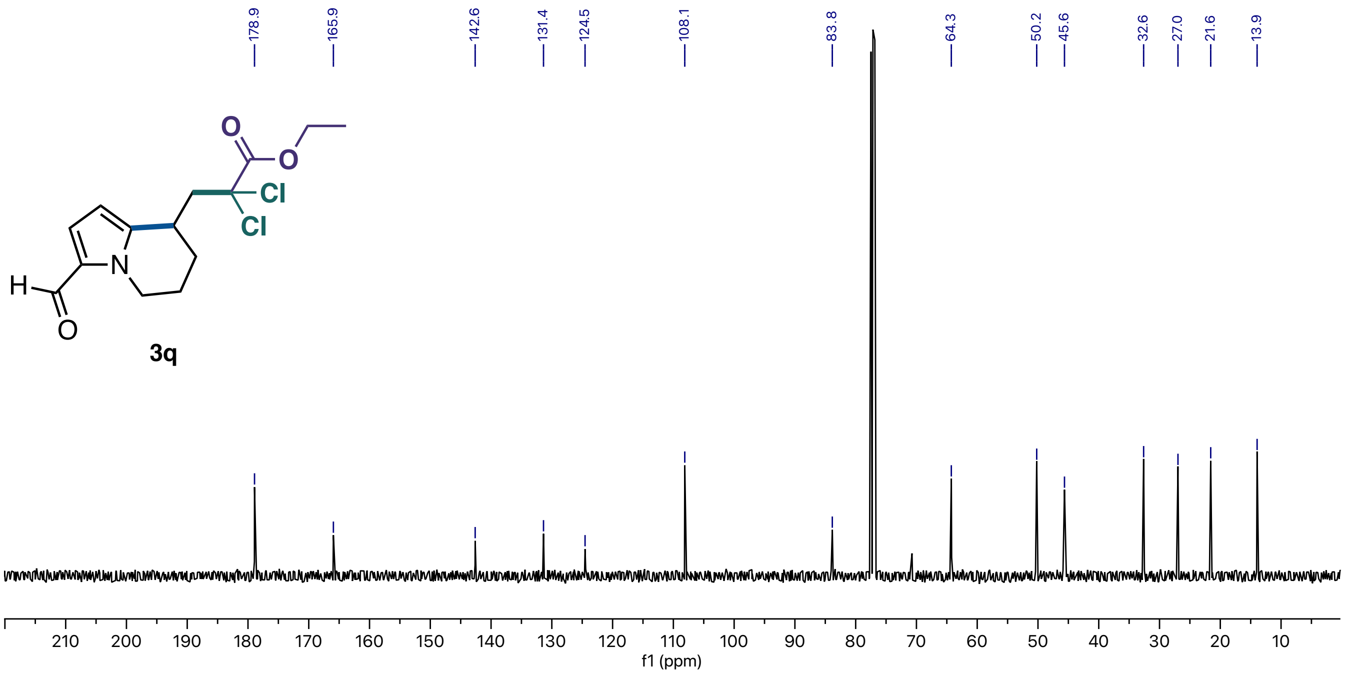

^{1}H NMR (400 MHz, $CDCl_3$)

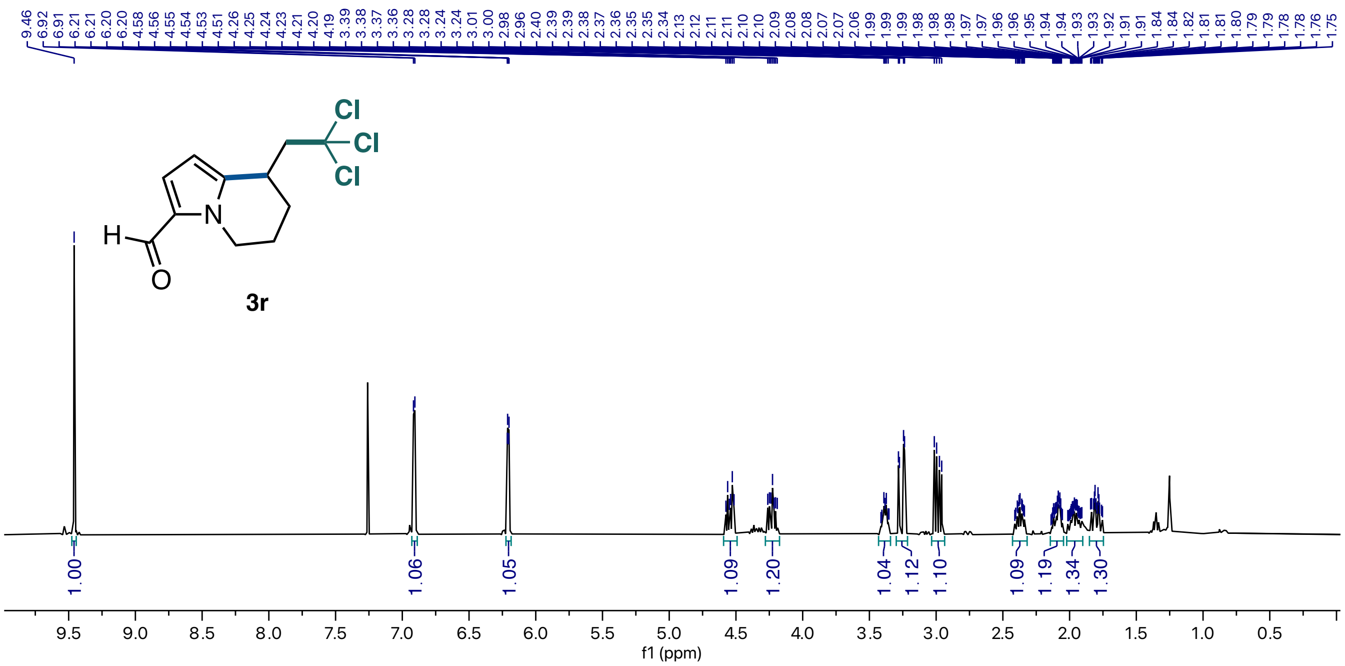

^{13}C{^{1}H} NMR (100 MHz, $CDCl_3$)

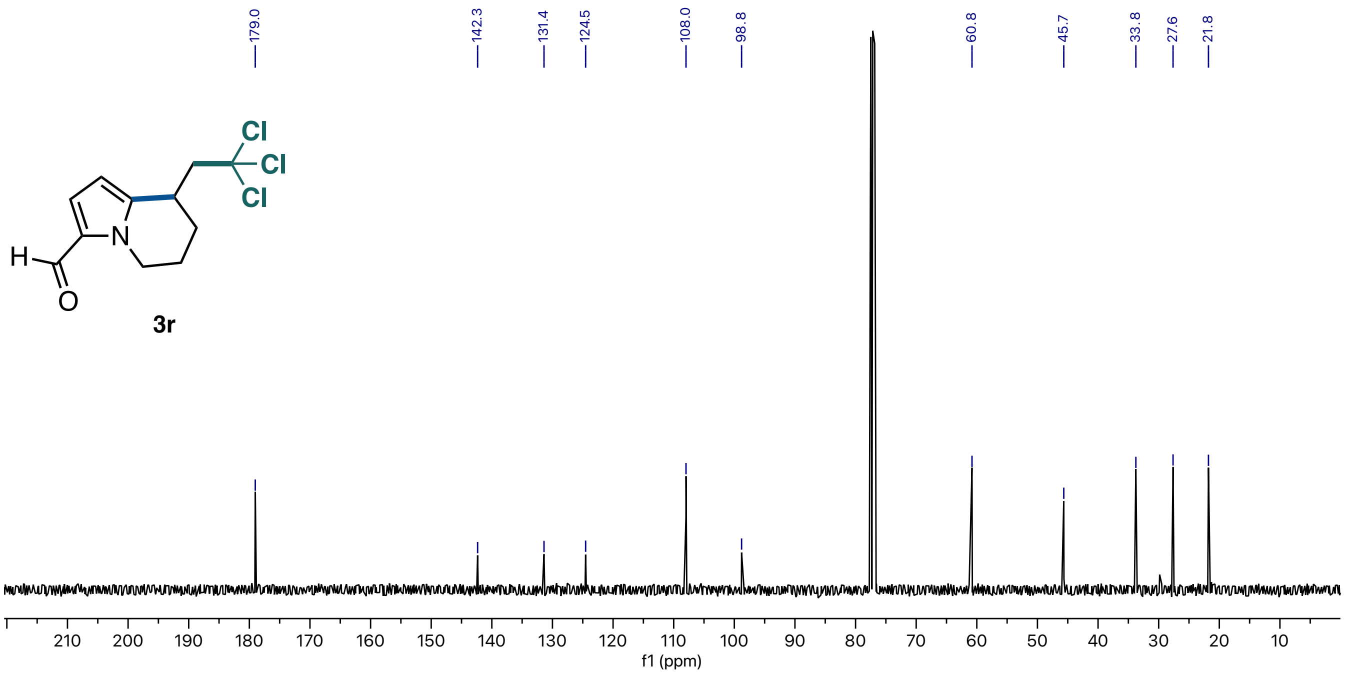

^{1}H NMR (400 MHz, $CDCl_3$)

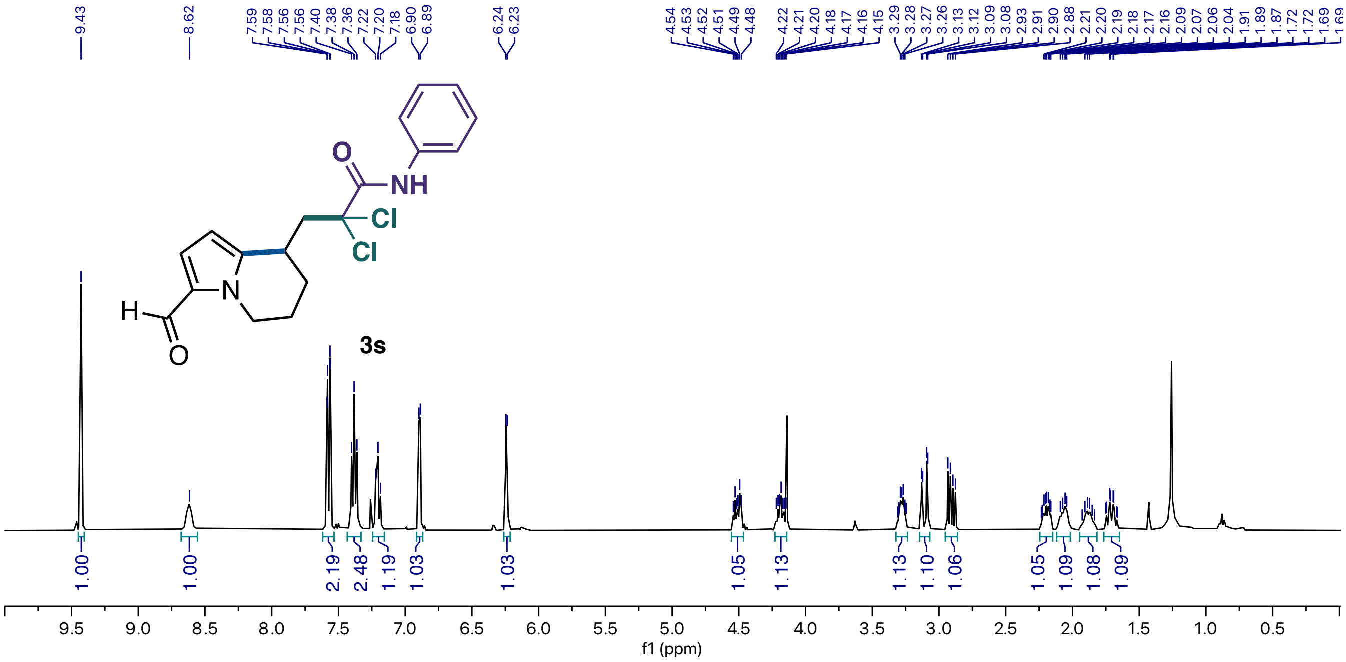

^{13}C{^{1}H} NMR (100 MHz, $CDCl_3$)

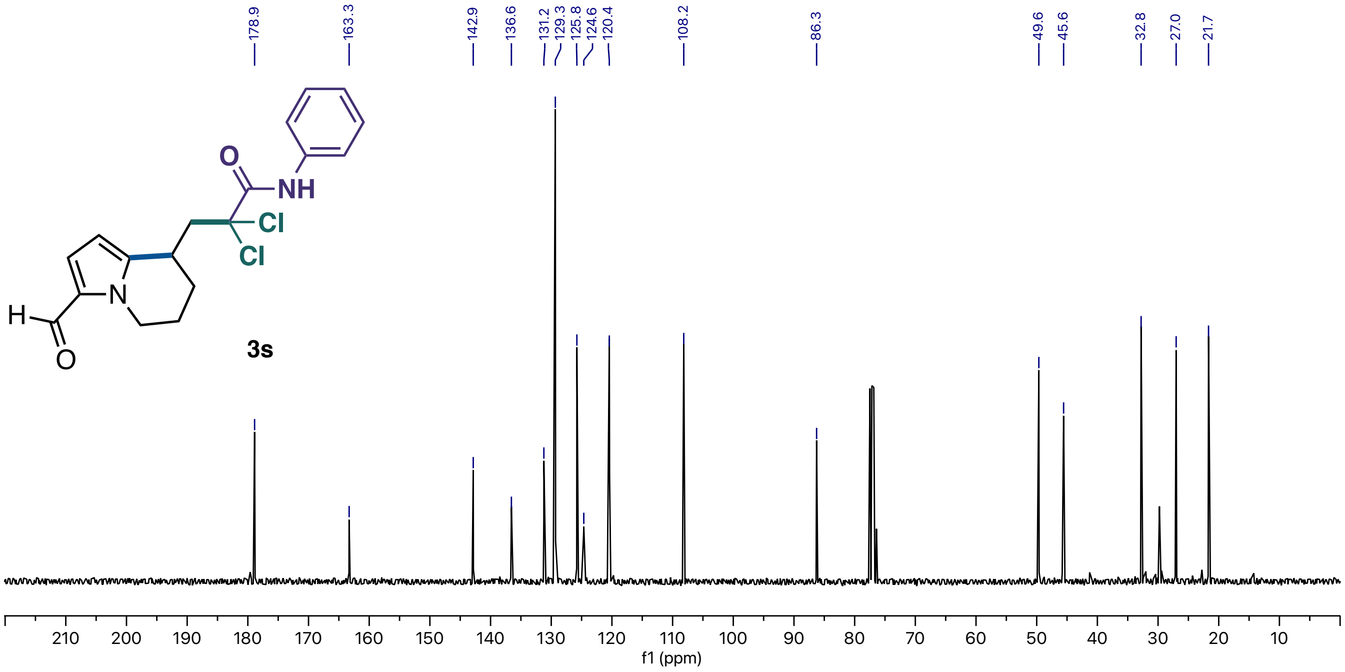

¹H NMR (400 MHz, CDCl$_3$)

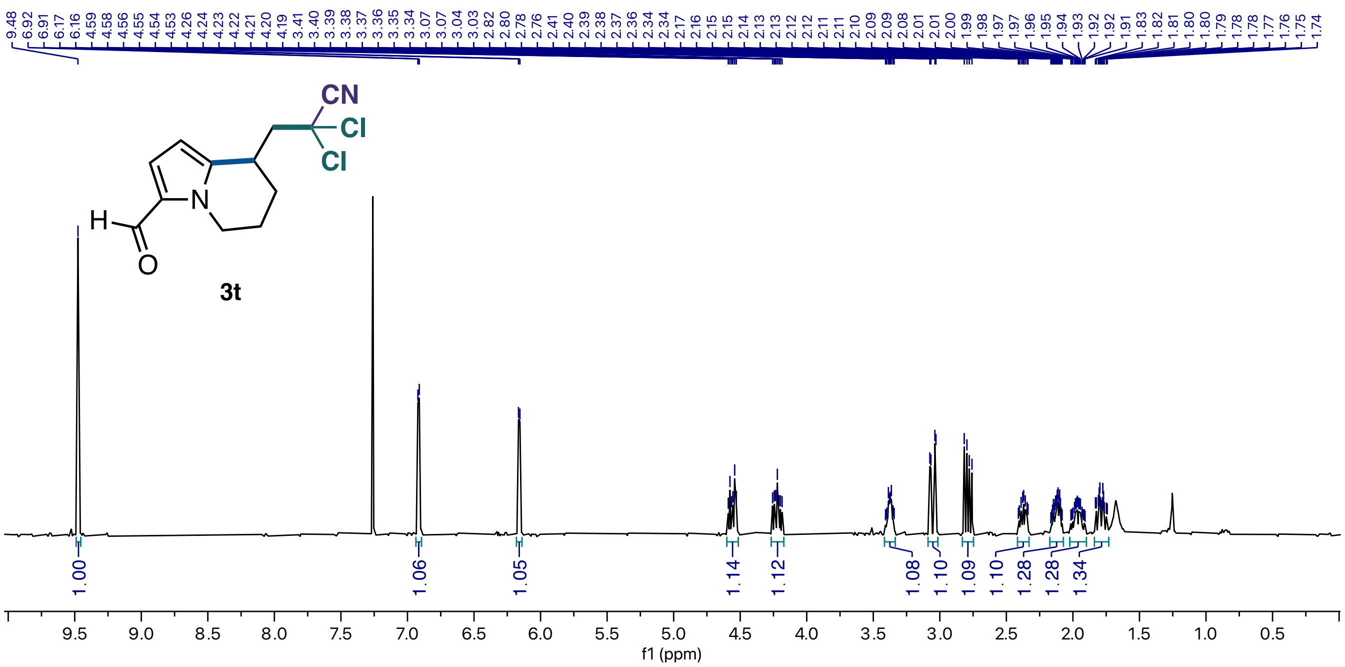

^{13}C{^{1}H} NMR (100 MHz, CDCl$_3$)

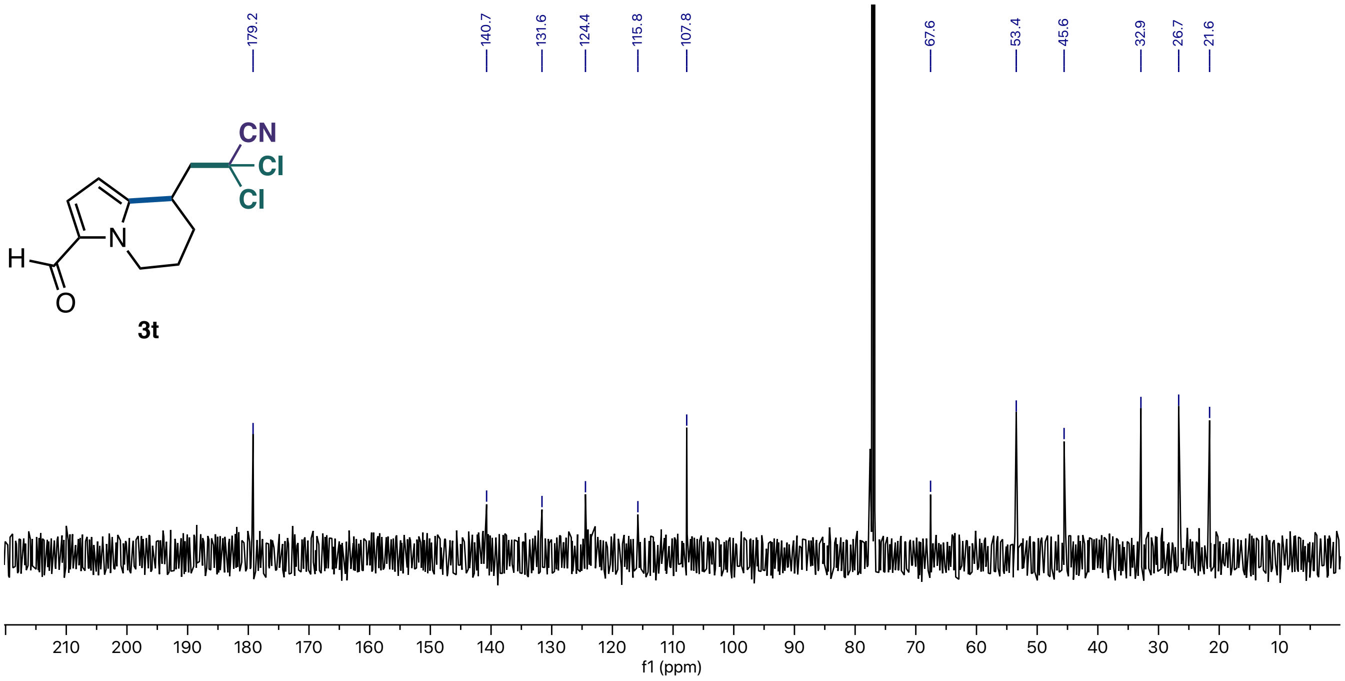

^{1}H NMR (400 MHz, $CDCl_3$)

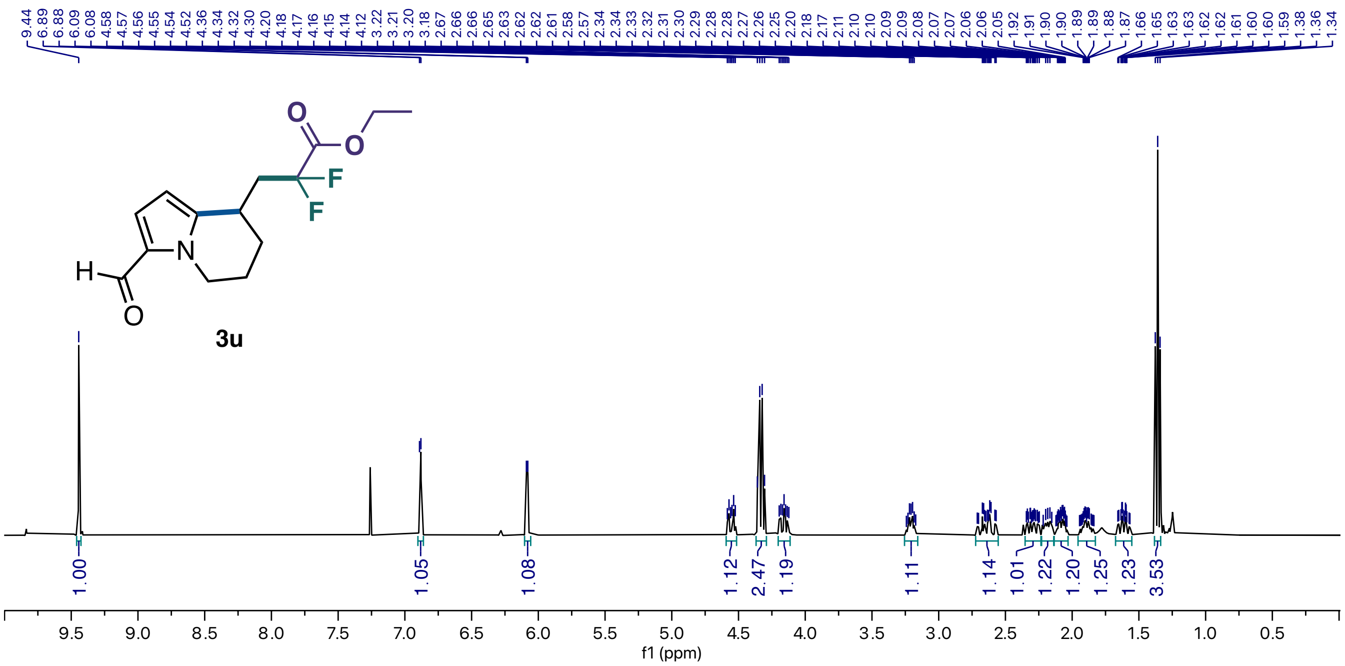

^{13}C{^{1}H} NMR (100 MHz, $CDCl_3$)

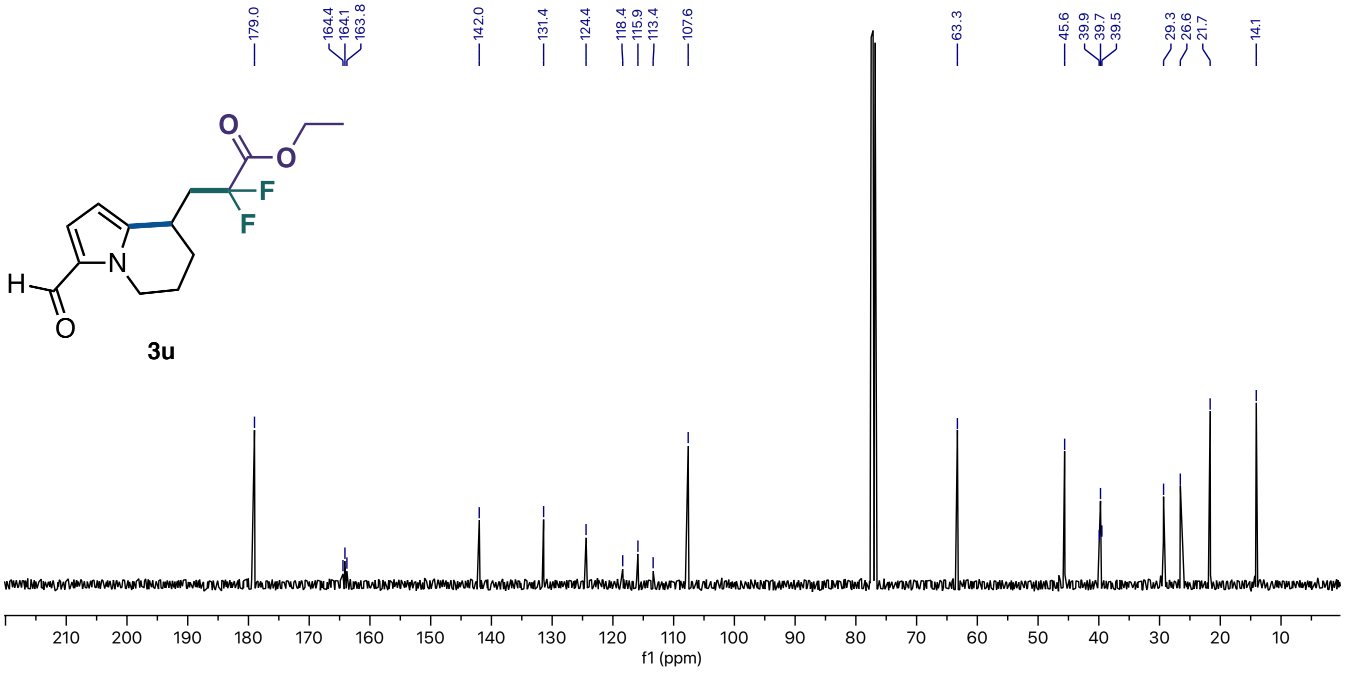

^{1}H NMR (400 MHz, $CDCl_3$)

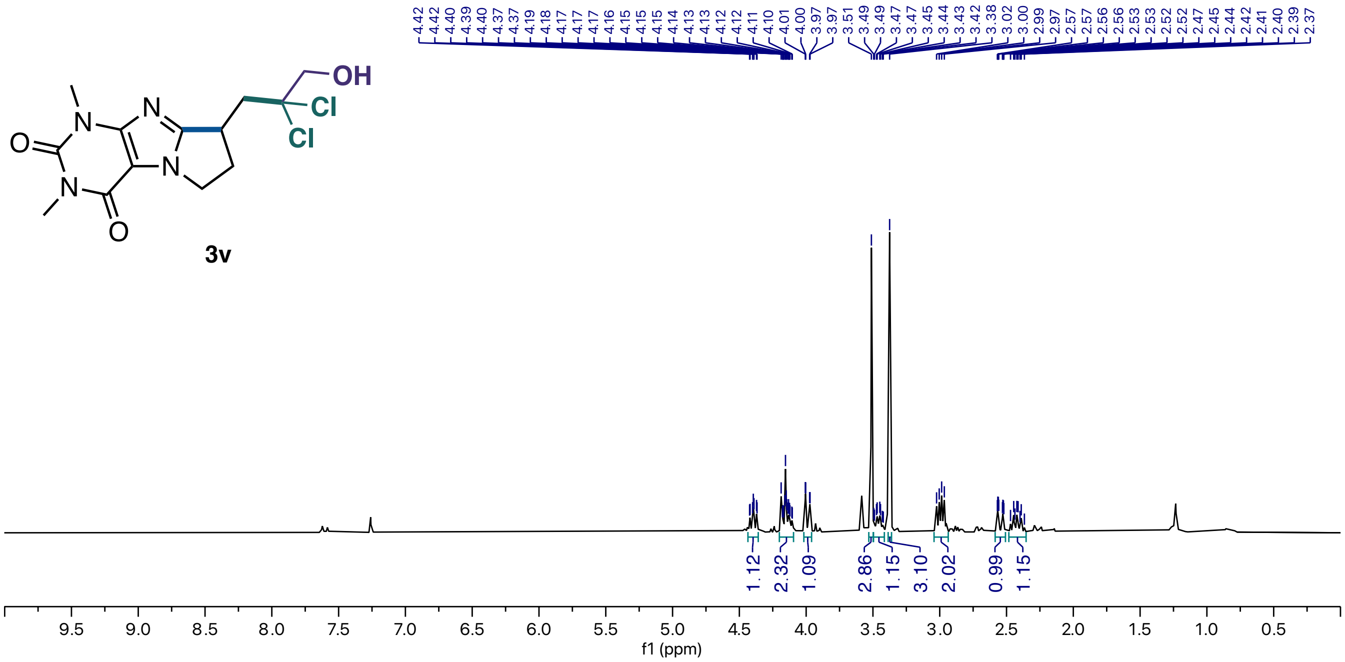

^{13}C{^{1}H} NMR (100 MHz, $CDCl_3$)

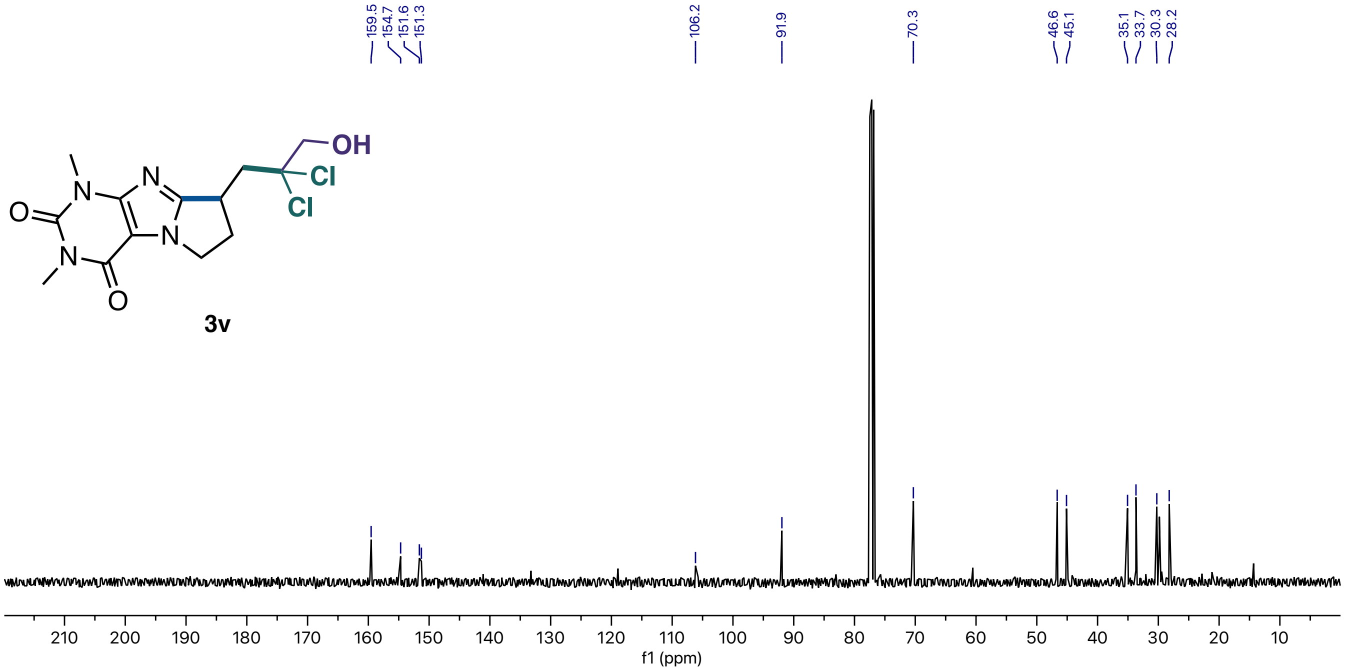

^{1}H NMR (400 MHz, $CDCl_3$)

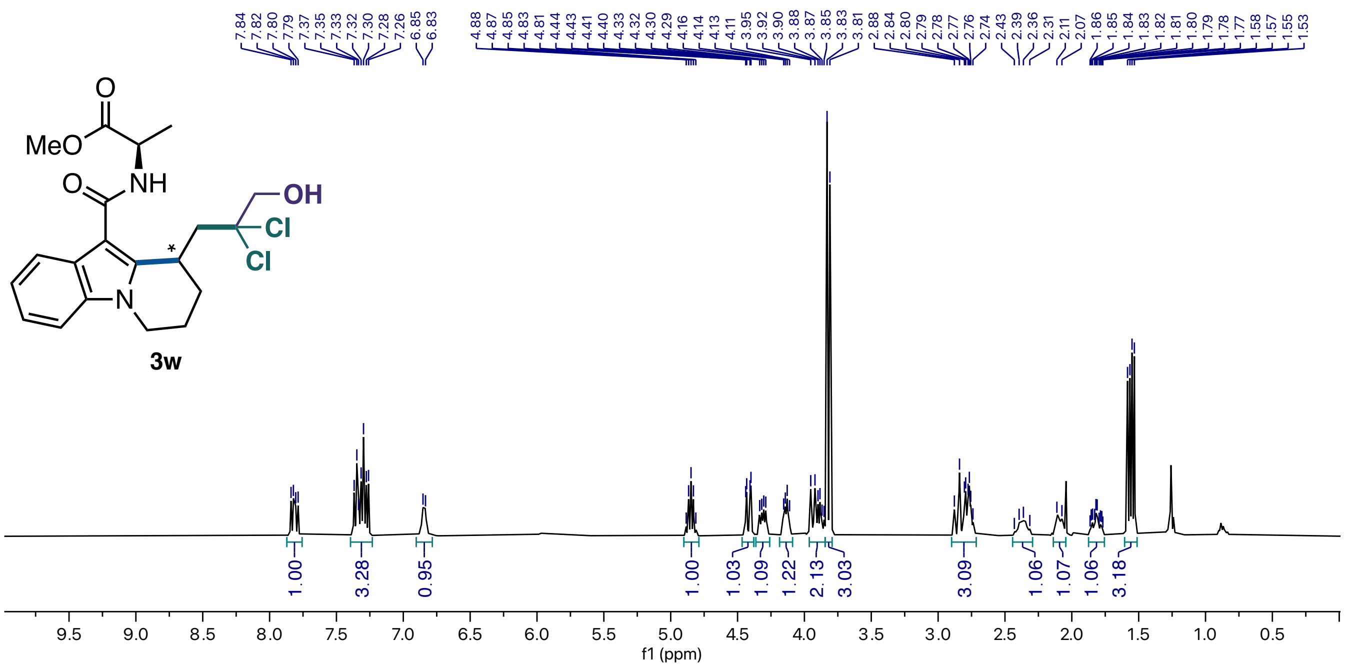

3w

^{13}C{^{1}H} NMR (100 MHz, $CDCl_3$)

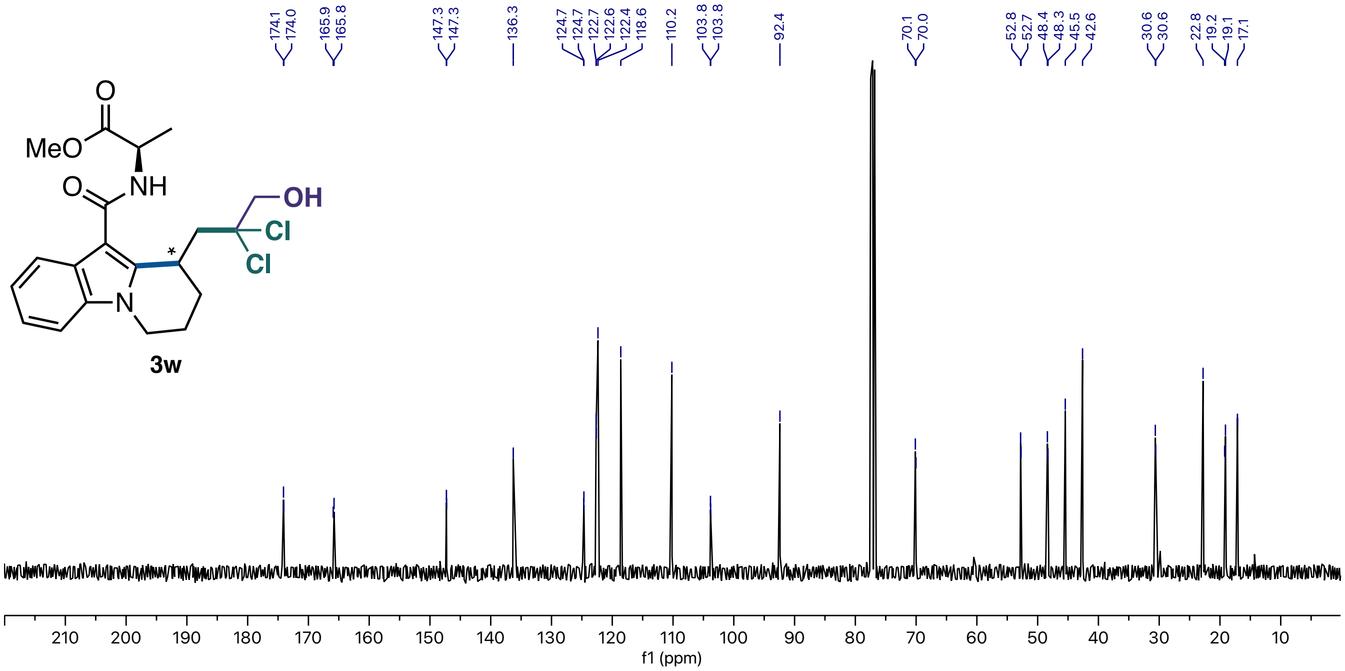

3w

^{1}H NMR (400 MHz, $CDCl_3$)

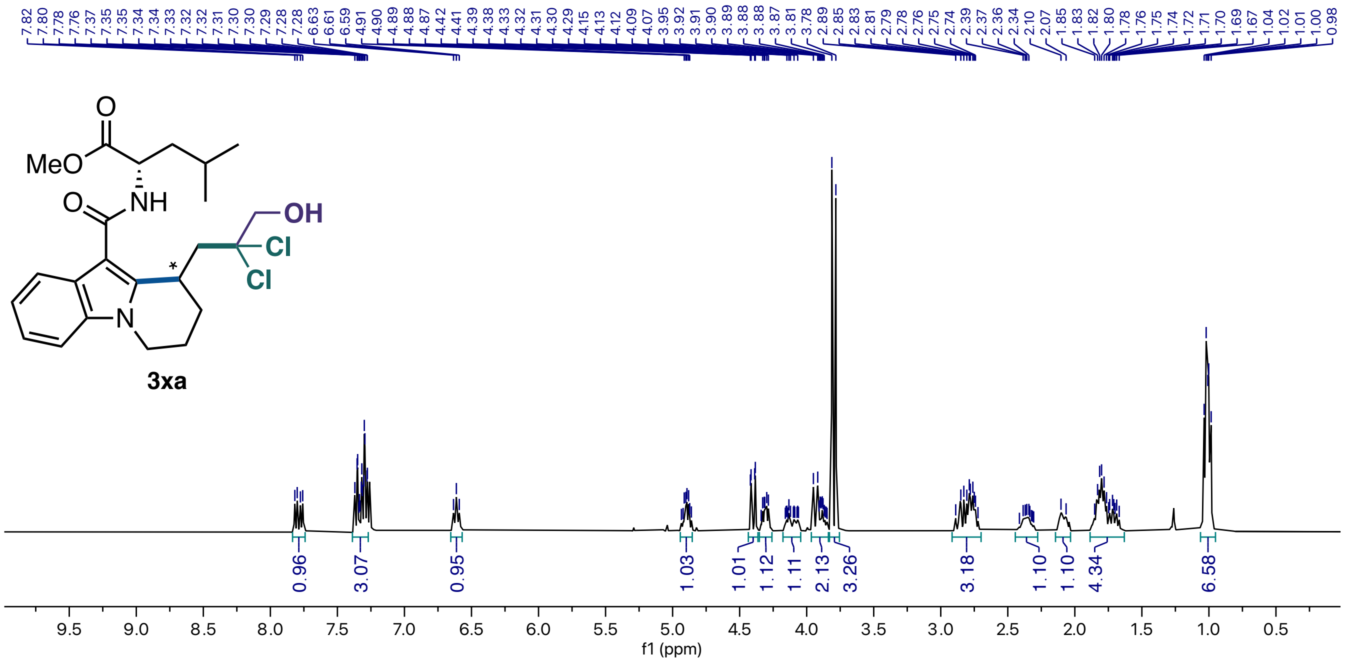

^{13}C{^{1}H} NMR (100 MHz, $CDCl_3$)

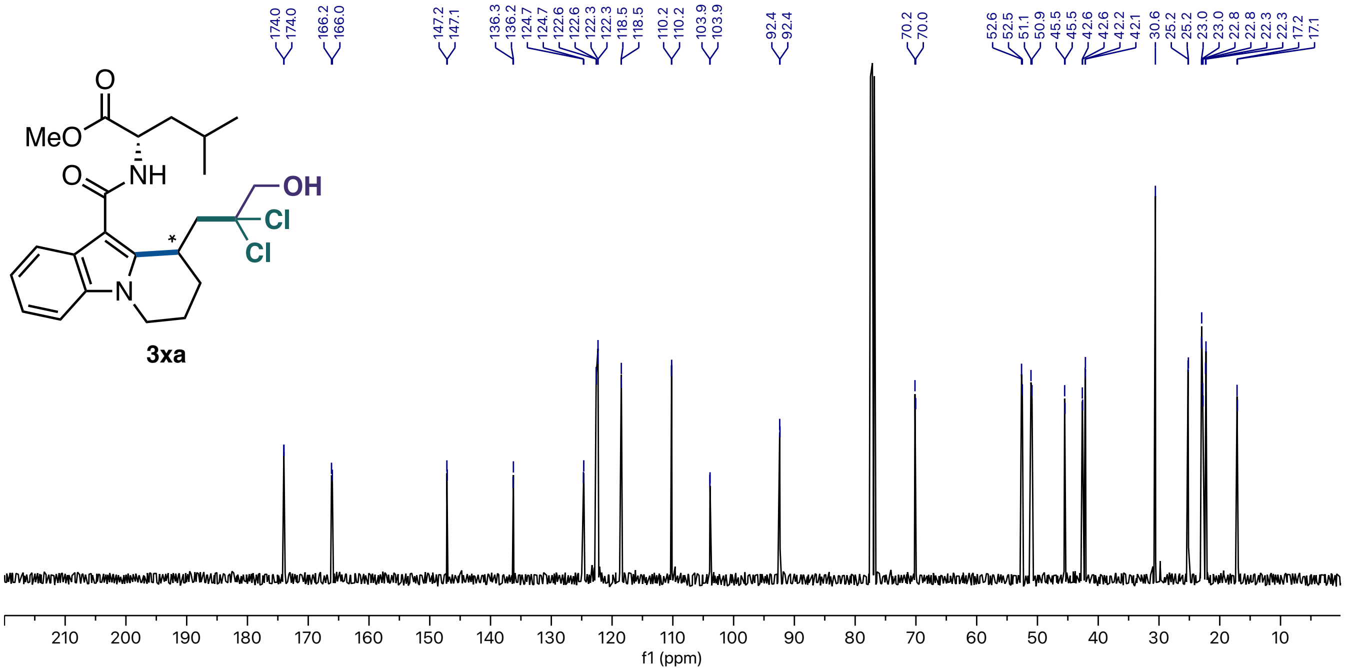

^{1}H NMR (400 MHz, $CDCl_3$)

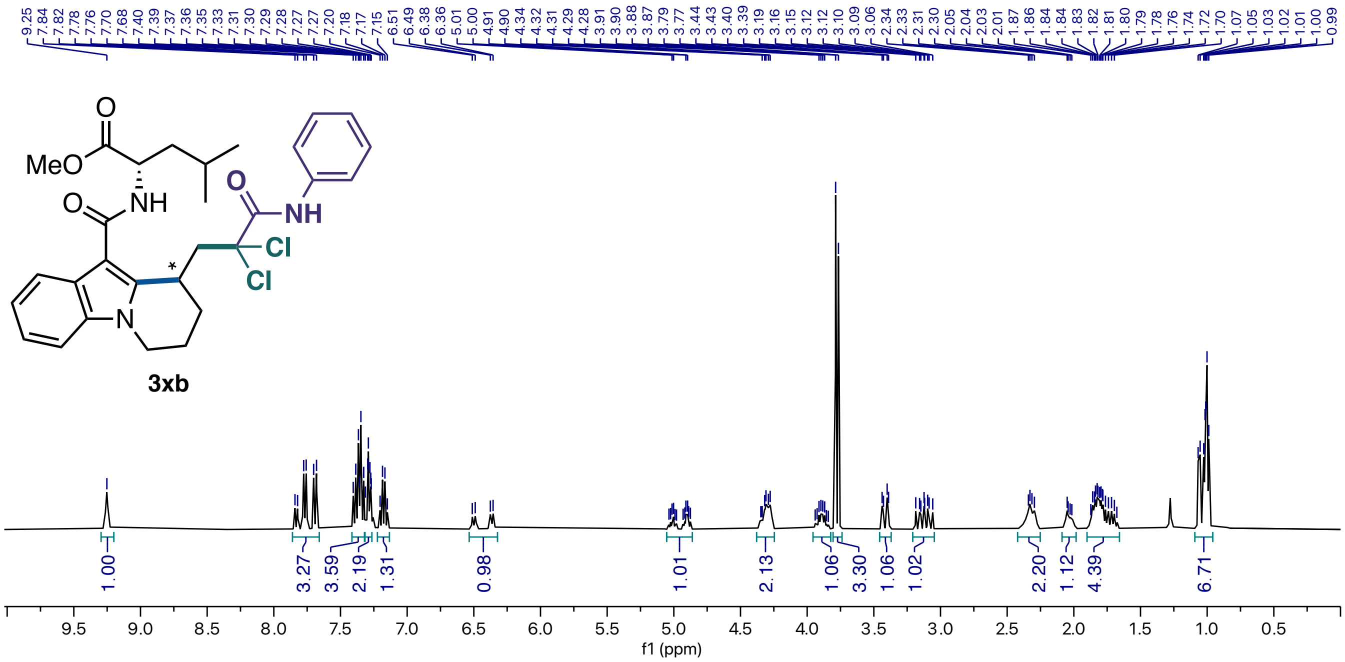

^{13}C{^{1}H} NMR (100 MHz, $CDCl_3$)

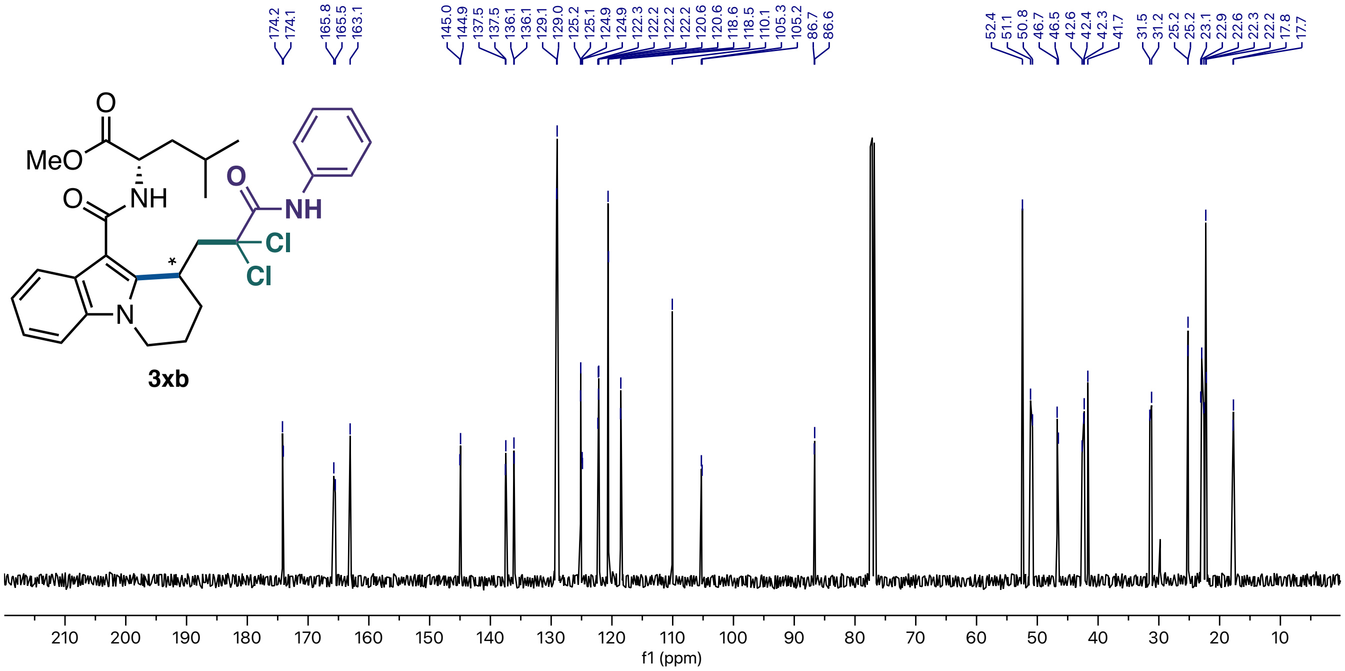

^{1}H NMR (400 MHz, $CDCl_3$)

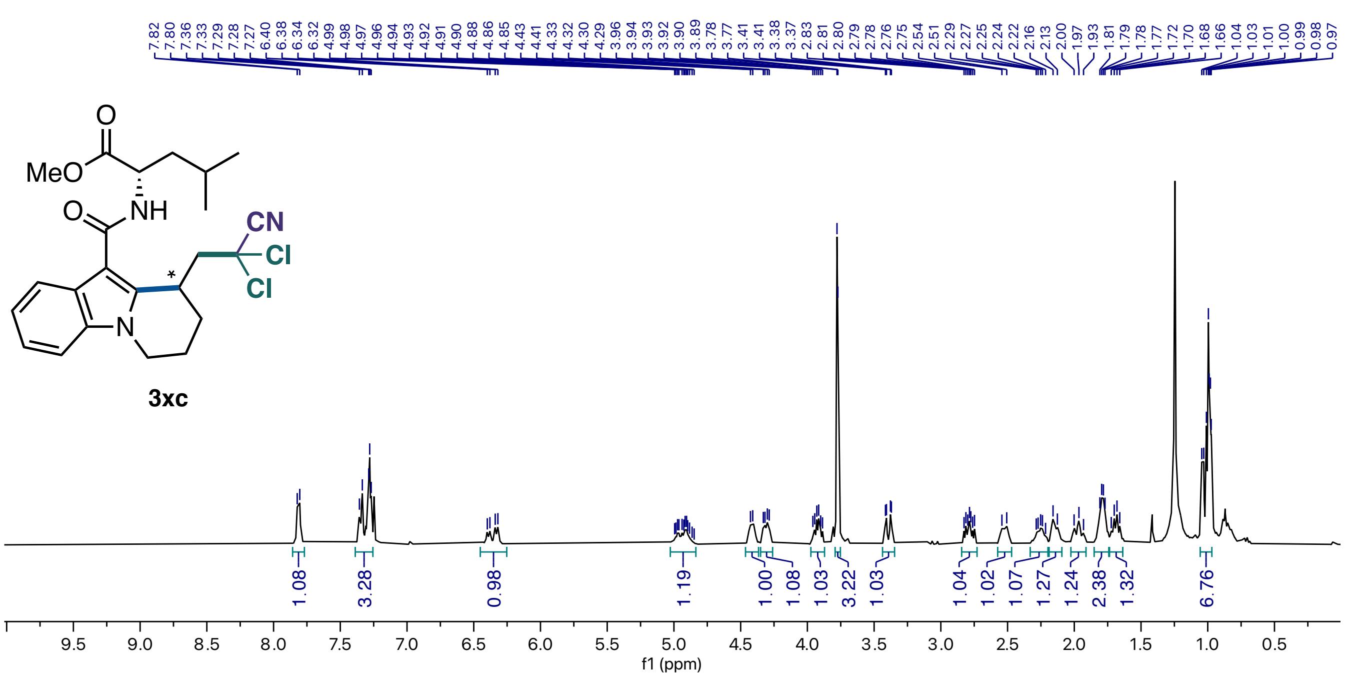

^{13}C{^{1}H} NMR (100 MHz, $CDCl_3$)

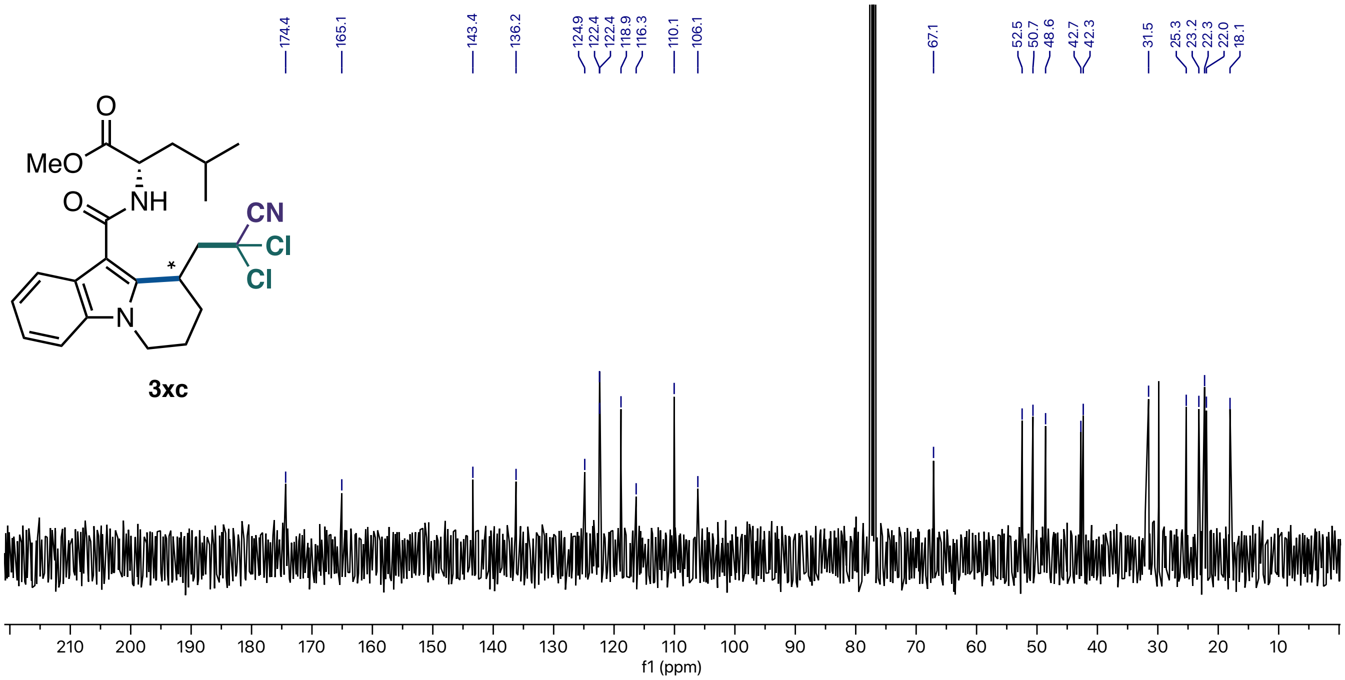

^{1}H NMR (400 MHz, $CDCl_3$)

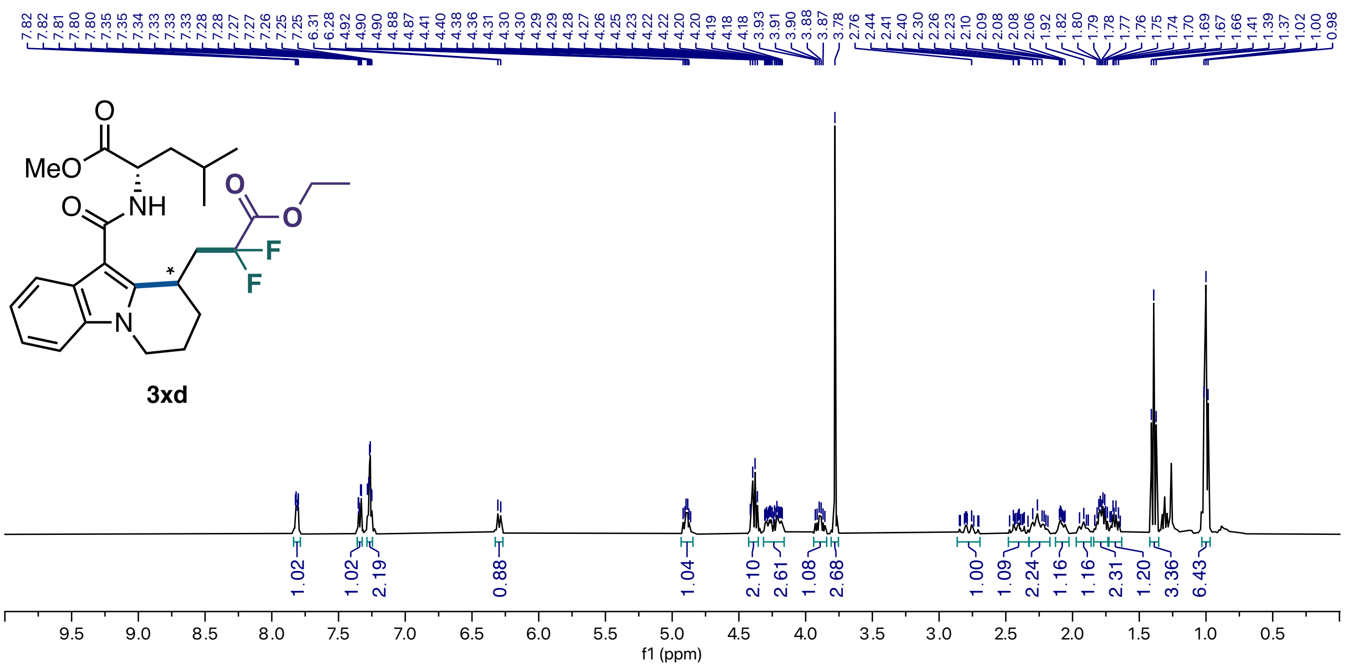

^{13}C{^{1}H} NMR (100 MHz, $CDCl_3$)

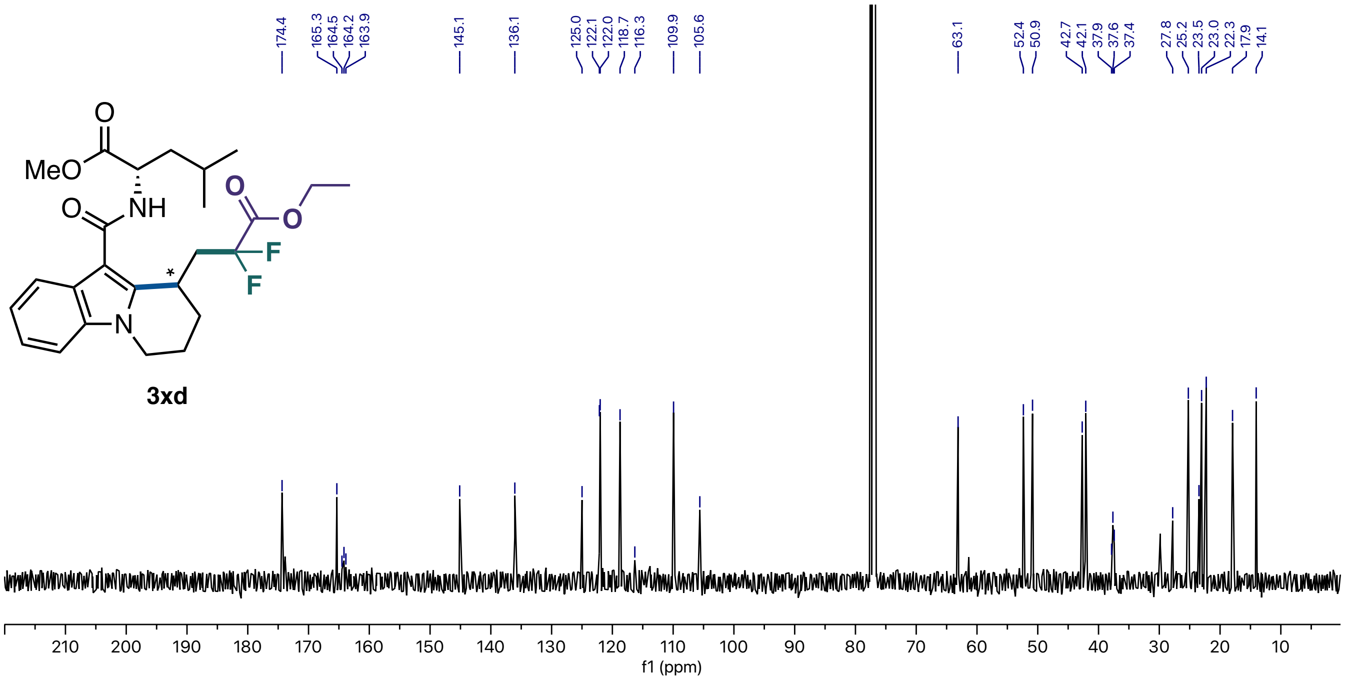

^{1}H NMR (400 MHz, $CDCl_3$)

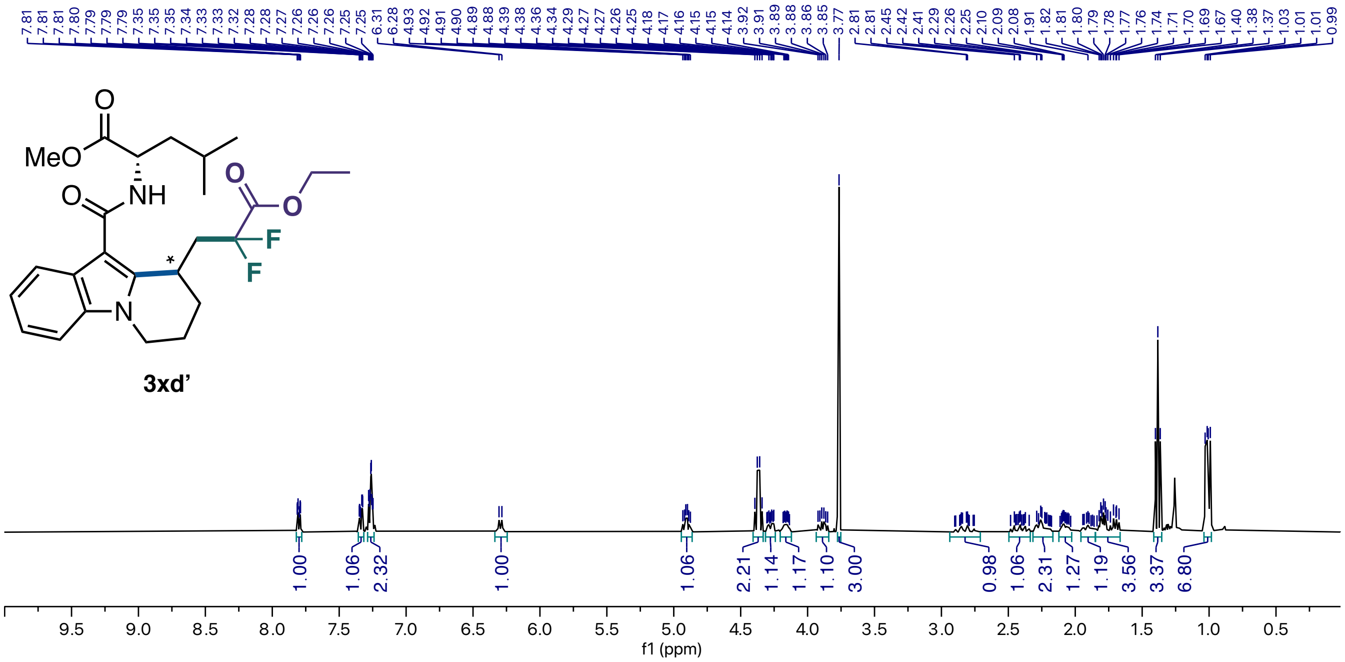

^{13}C{^{1}H} NMR (100 MHz, $CDCl_3$)

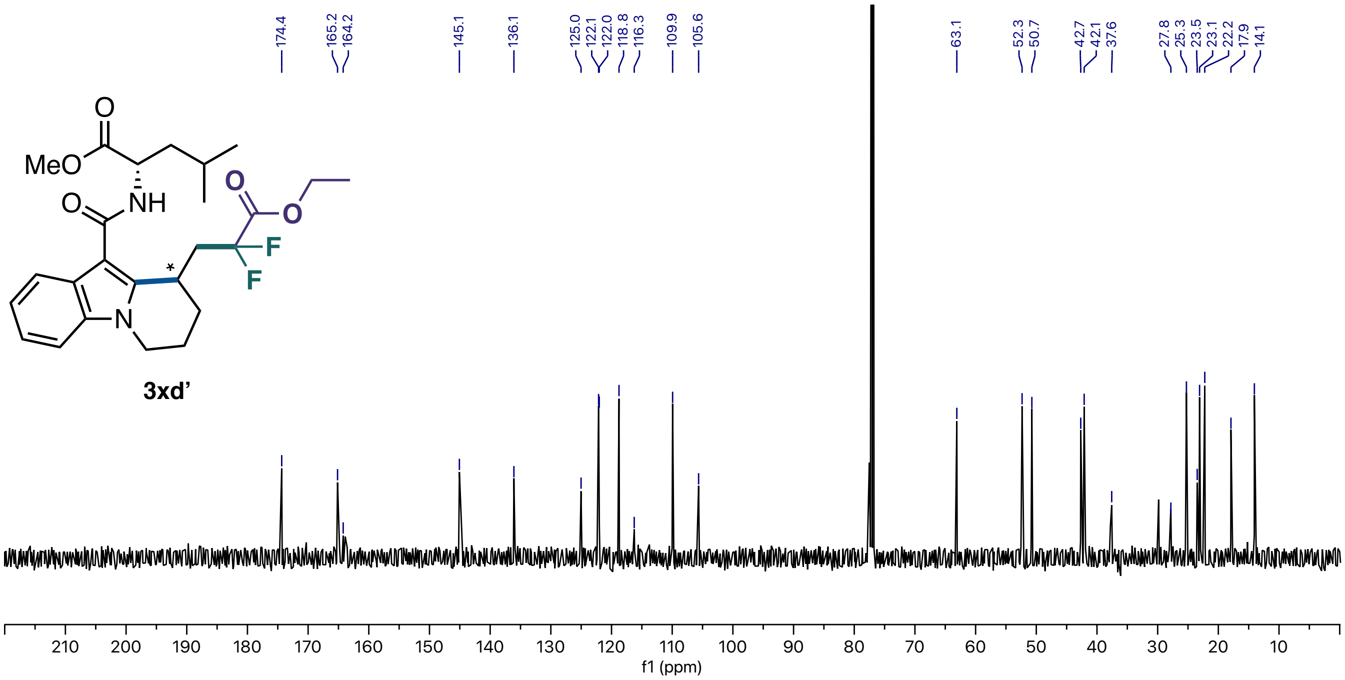

^{1}H NMR (400 MHz, $CDCl_3$)

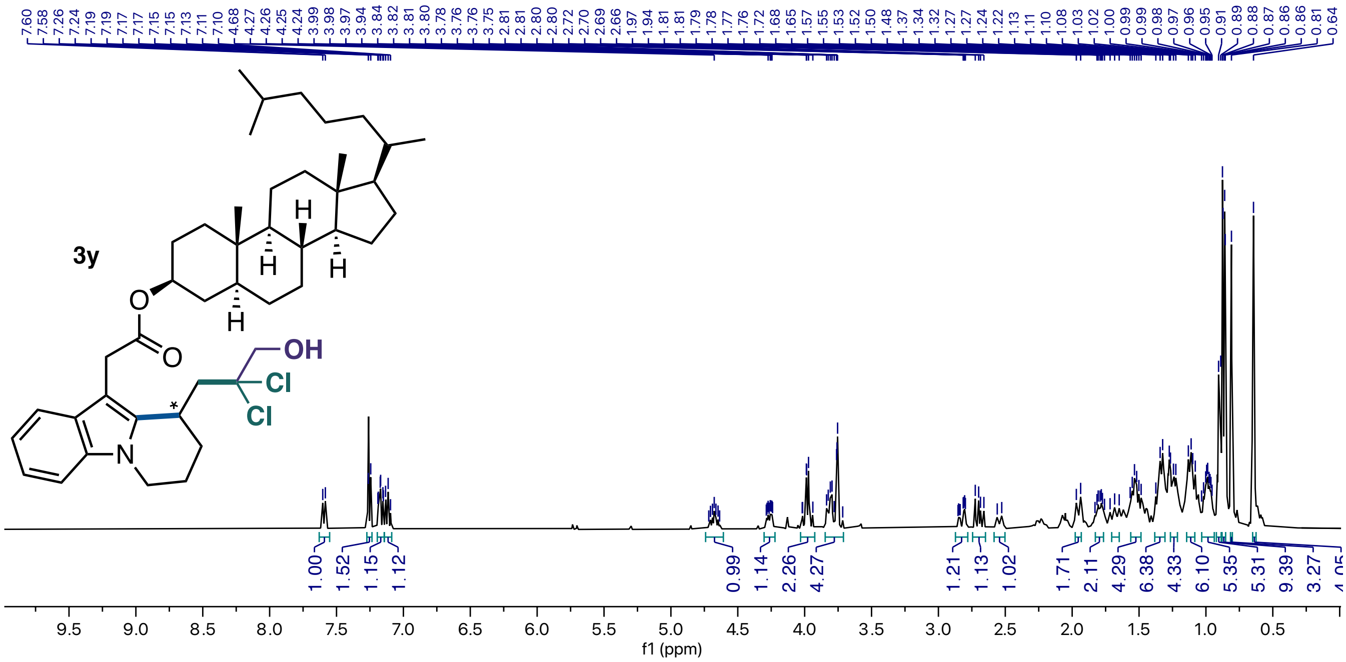

^{13}C{^{1}H} NMR (100 MHz, $CDCl_3$)

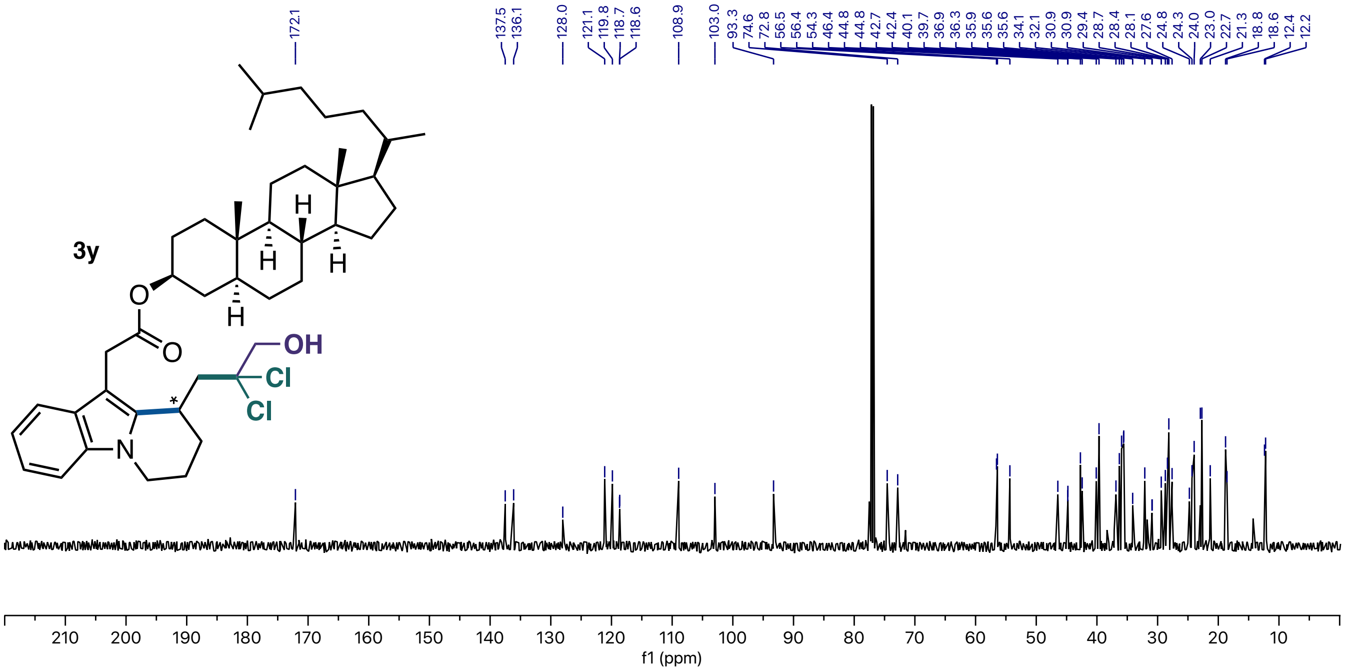

^{1}H NMR (400 MHz, $CDCl_3$)

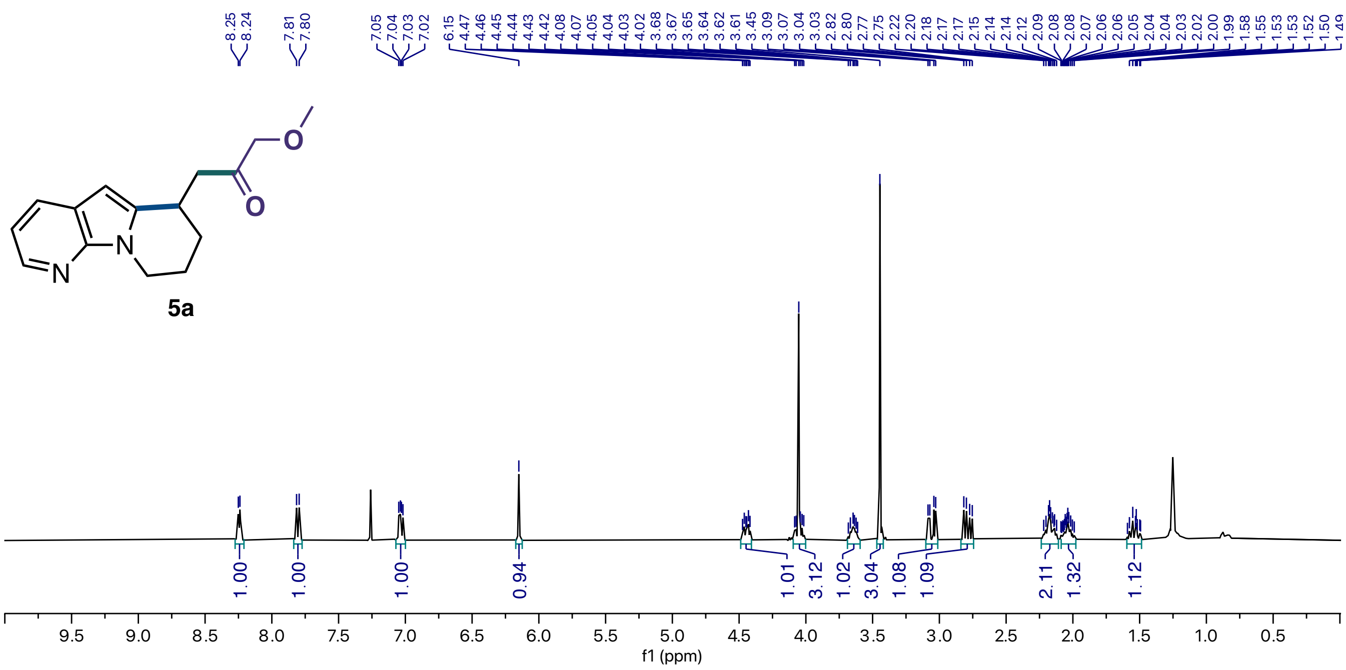

^{13}C{^{1}H} NMR (100 MHz, $CDCl_3$)

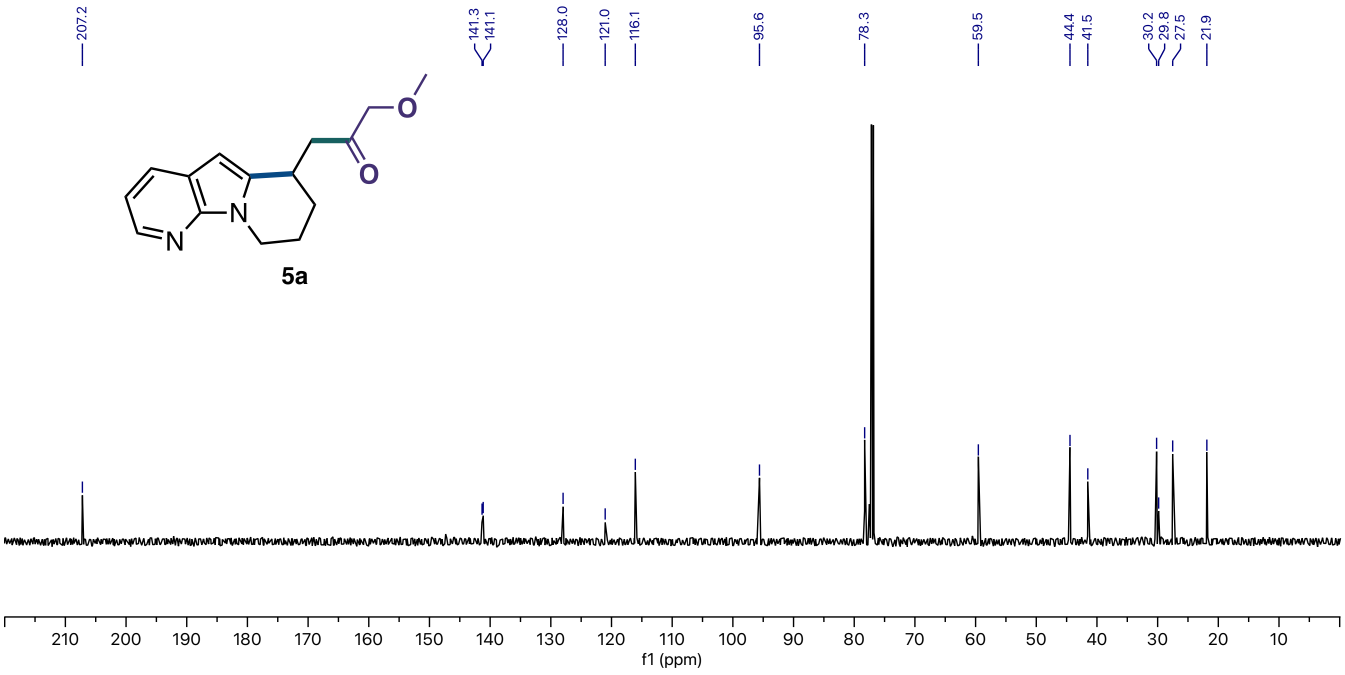

^{1}H NMR (400 MHz, $CDCl_3$)

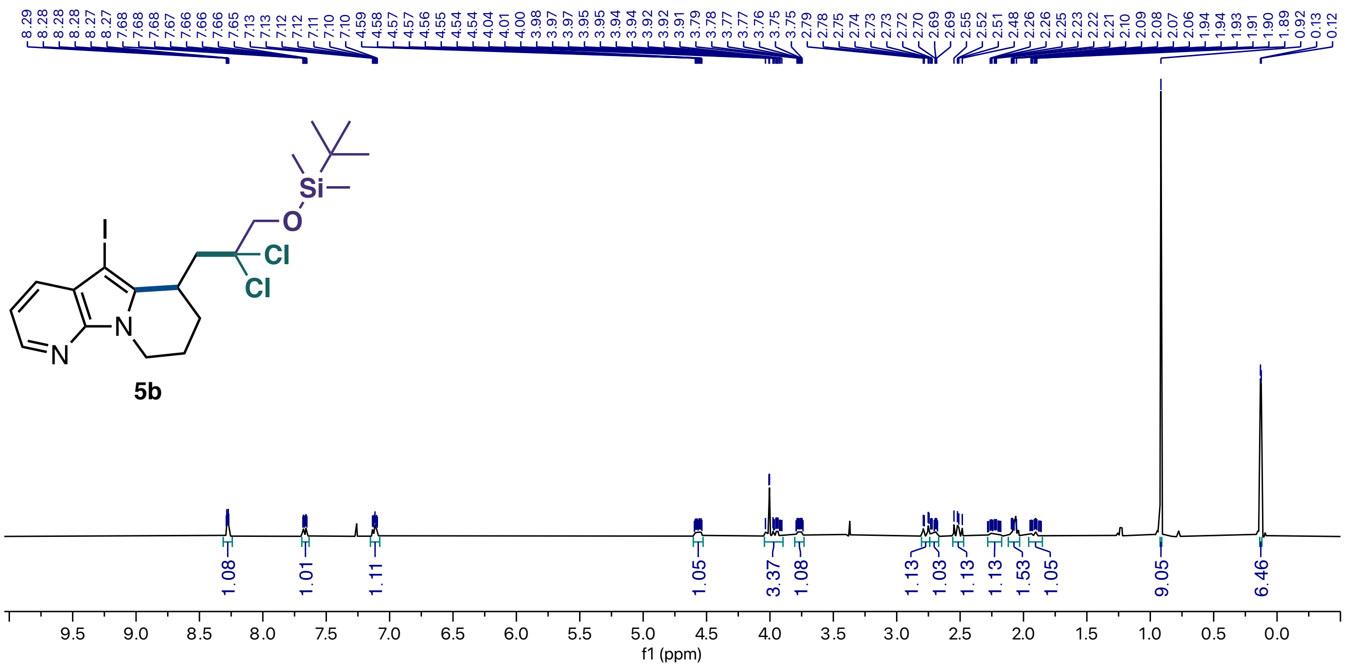

^{13}C{^{1}H} NMR (100 MHz, $CDCl_3$)

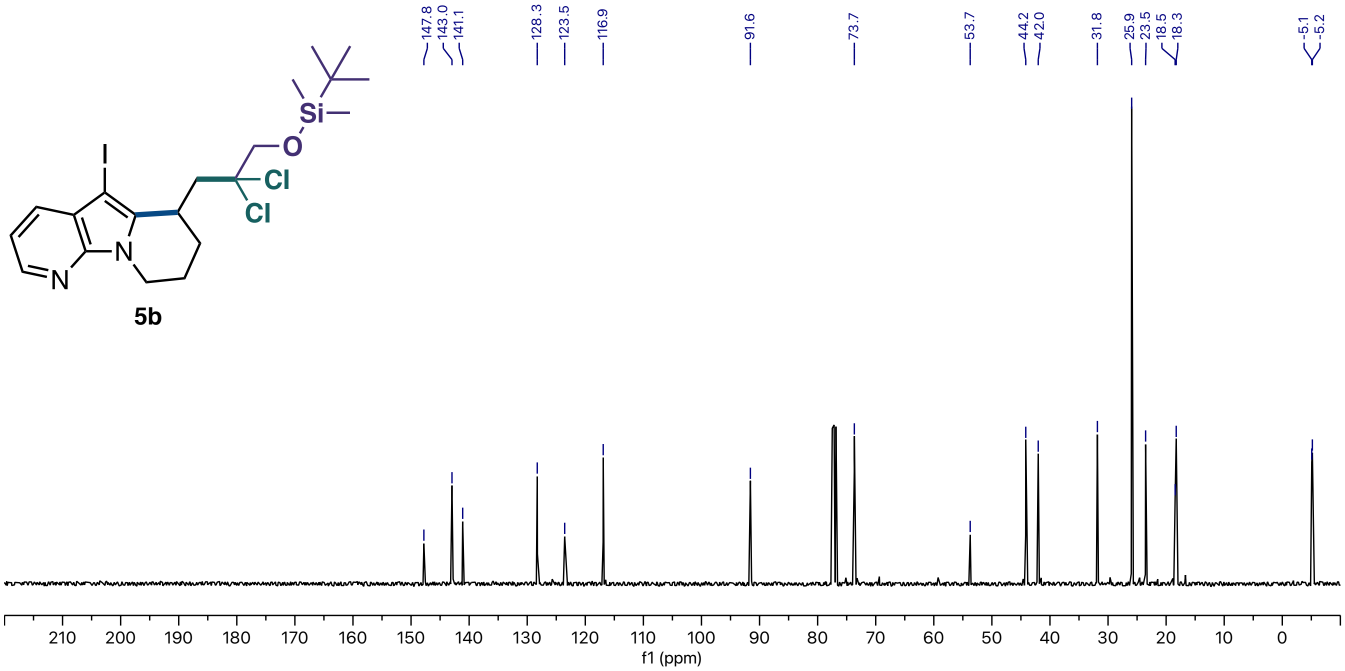

^{1}H NMR (400 MHz, $CDCl_3$)

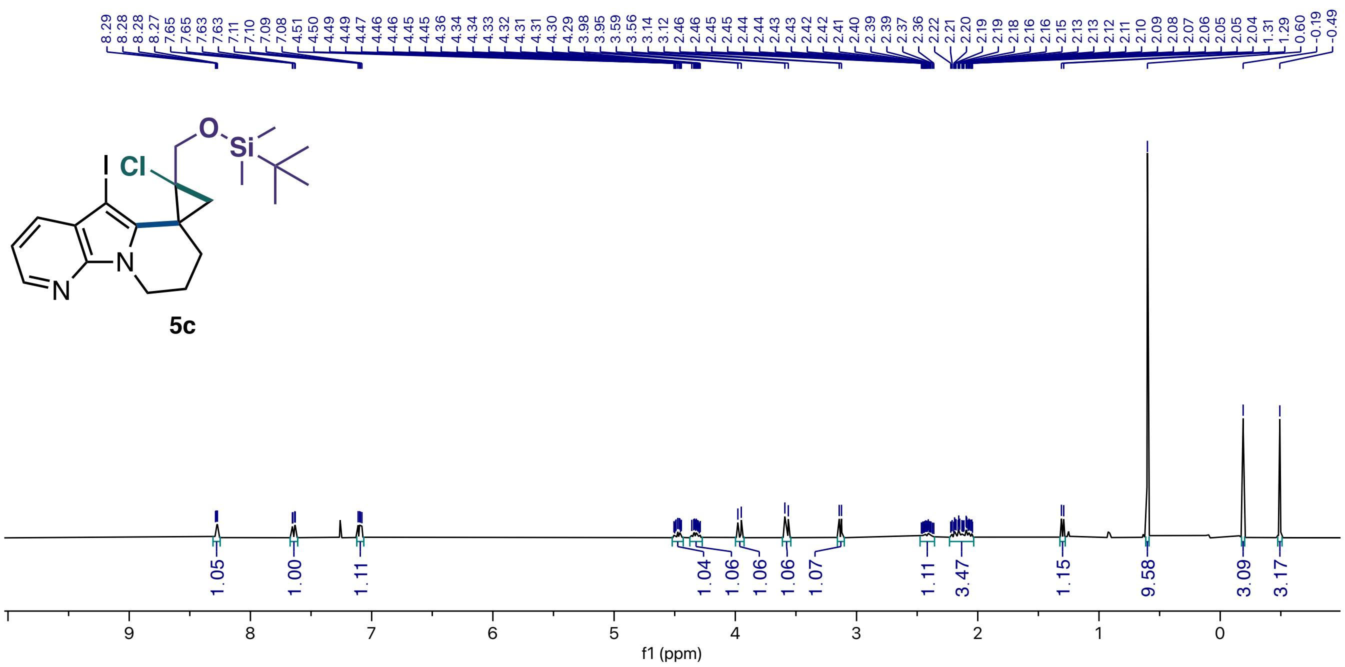

^{13}C{^{1}H} NMR (100 MHz, $CDCl_3$)

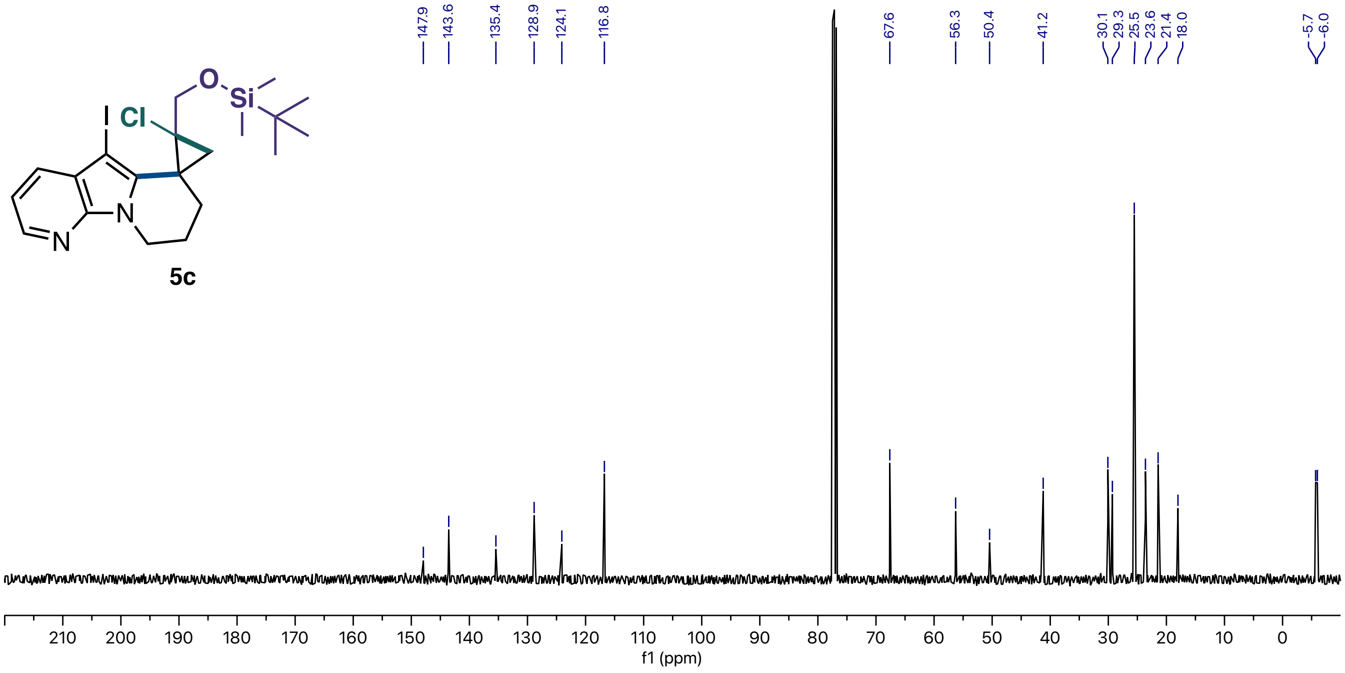

^{1}H NMR (400 MHz, $CDCl_3$)

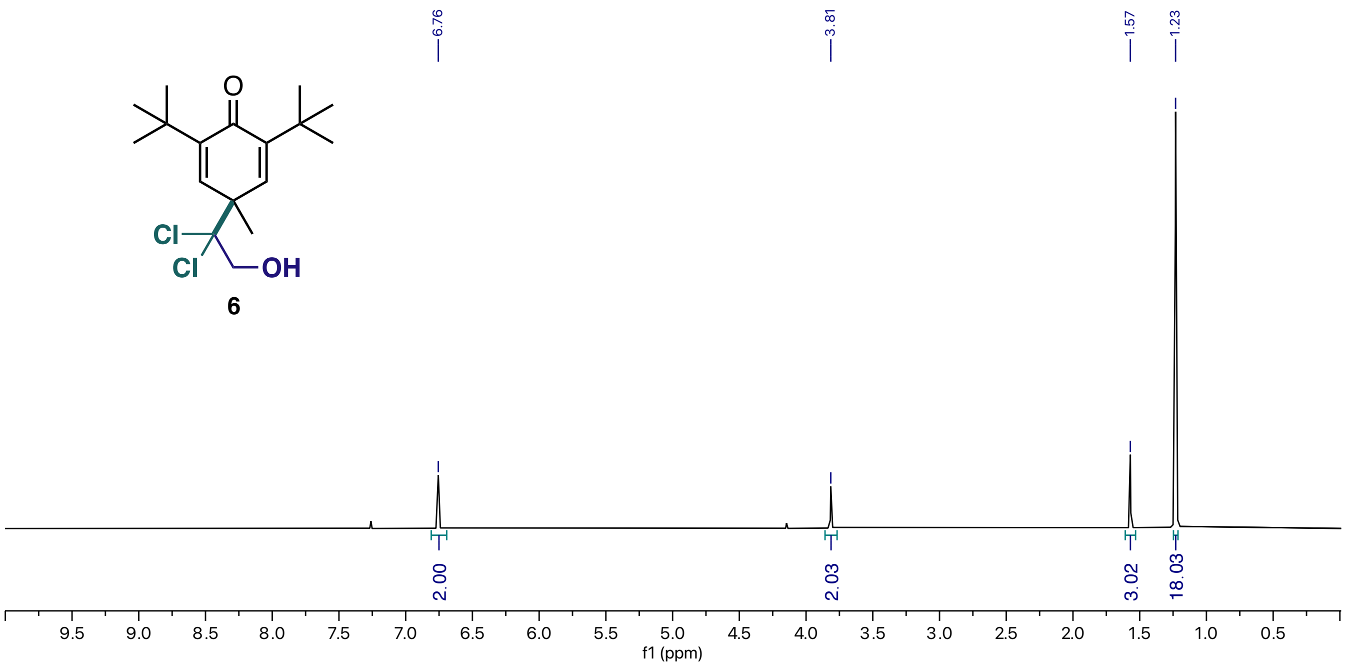

^{13}C{^{1}H} NMR (100 MHz, $CDCl_3$)

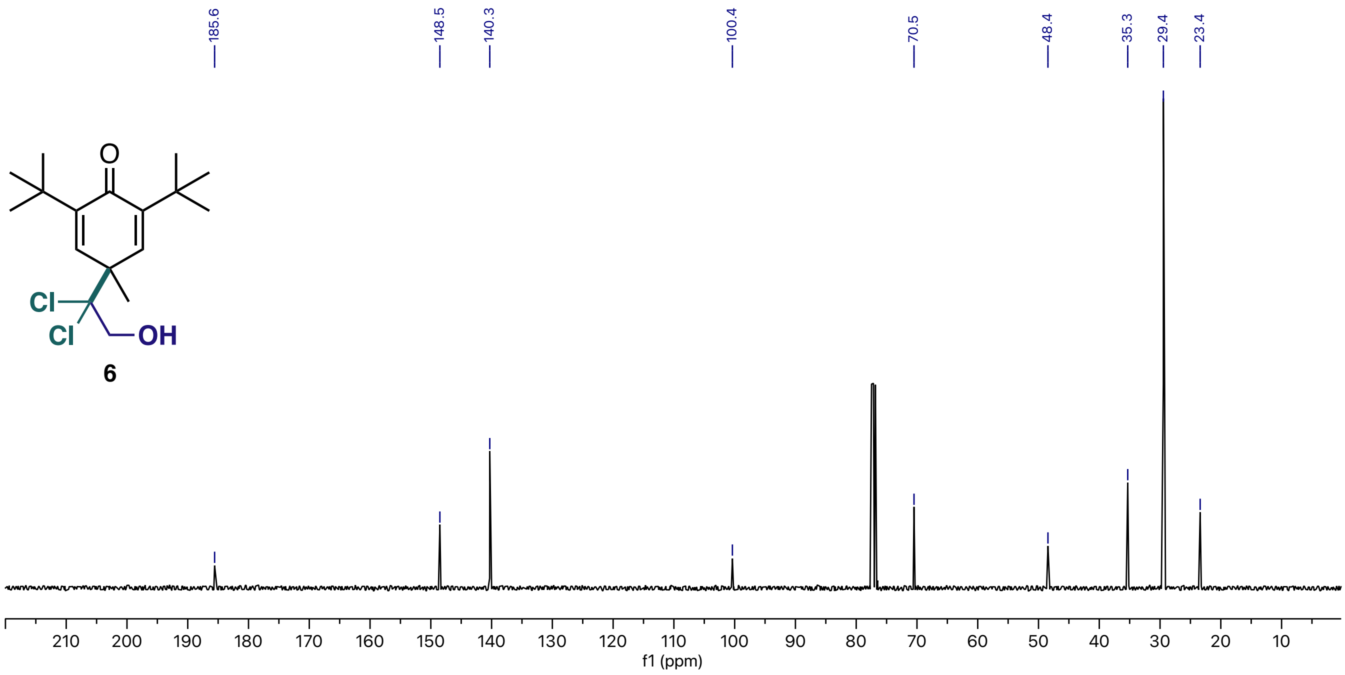

References

[1] E. Paleo, Y. M. Osornio, L. D. Miranda, *Org. Biomol. Chem.* **2011**, *9*, 361-362.
[2] A. Ghosh, J. A. Walker, Jr., A. Ellern, L. M. Stanley, *ACS Catal.* **2016**, *6*, 2673−2680.
[3] D. Bagnis, L. Beverina, H. Huang, F. Silvestri, Y. Yao, H. Yan, G. A. Pagani, T. J. Marks, A. Facchetti, *J. Am. Chem. Soc.* **2010**, *132*, 4074–4075.
[4] A. Banerjee, S. Sarkar, J. A. Shah, N. C. Frederiks, E. A. Bazan-Bergamino, C. J. Johnson, M.-Y. Ngai, *Angew. Chem. Int. Ed.* **2022**, *61*, e202113841.
[5] M. K. Eberle, M. J. Shapiro, R. Stucki, *J. Org. Chem.* **1987**, *52*, 4661–4665.
[6] Z Ding, N. Yoshikai, *Angew. Chem. Int. Ed.* **2013**, *52*, 8574 –8578.
[7] L. Sole Feu, I. Carranco Moruno, J. Aiguade Bosch, C. Puig Duran, S. Fonquerna Pou (Almirall, S.A.), patent WO 2014/095920 A1, 2014.
[8] Z. He, Z. Li, S. Lai, H. Li, *Org. Lett.* **2024**, *26*, 6652–6657.
[9] L. R. Chavada, P. Mishra, A. K. Pandey, *J. Org. Chem.* **2024**, *89*, 9233–9242.
[10] J. García-Ramírez, L. A. González-Cortés, L. D. Miranda, *Org. Lett.* **2022**, *24*, 8093–8097.
[11] Y. Zhao, S. Ge, *Angew. Chem. Int. Ed.* **2022**, *61*, e202116133.
[12] A. B. Kumar, J. M. Anderson, A. L. Melendez, R. Manetsch, *Bioorg. Med. Chem. Lett.* **2012**, *22*, 4740–4744.
[13] Q.-H. Zhou, J.-Y. Dai, W.-J. Zhao, X.-Y. Zhong, C.-Y. Liu, W.-W. Luo, Z.-W. Li, J.-S. Li, W.-D. Liu, *Org. Biomol. Chem.* **2023**, *21*, 3317–3322.
[14] M. D. Rathnayake, J. D. Weaver III, *Org. Lett.* **2019**, *21*, 9681–9687.
[15] X. Zhang, S. Zhang, S. Li, X. Feng, Y. Yamamoto, M. Bao, *Chem. Commun.* **2022**, *58*, 2670–2673.
[16] A. Bartoszewicz, M. Kalek, J. Nilsson, R. Hiresova, J. Stawinski, *Synlett*, **2008**, 37–40.
[17] M. Pan, H. Liao, J. Liu, L. Gao, Y. Liu, X. Liu, L. Rong, *Eur. J. Org. Chem.* **2024**, *27*, e202400928.
[18] Bruker (2018). APEX3. Bruker AXS Inc., Madison, Wisconsin, USA.
[19] G. M. Sheldrick, *Acta Cryst.* **2008**, *A64*, 112–122.
[20] G. M. Sheldrick, *Acta Cryst.* **2015**, *A71*, 3–8.
[21] G. M. Sheldrick, *Acta Cryst.* **2015**, *C71*, 3–8.
[22] L. J. Farrugia, *J. Appl. Cryst.* **2012**, *45*, 849–854.
